# Grubbs Metathesis Enabled by a Light‐Driven *gem*‐Hydrogenation of Internal Alkynes

**DOI:** 10.1002/anie.202007030

**Published:** 2020-08-20

**Authors:** Tobias Biberger, Raphael J. Zachmann, Alois Fürstner

**Affiliations:** ^1^ Max-Planck-Institut für Kohlenforschung 45470 Mülheim/Ruhr Germany

**Keywords:** gem-hydrogenation, Grubbs catalysts, metal carbenes, metathesis, ruthenium

## Abstract

[(NHC)(cymene)RuCl_2_] (NHC=N‐heterocyclic carbene) complexes instigate a light‐driven *gem*‐hydrogenation of internal alkynes with concomitant formation of discrete Grubbs‐type ruthenium carbene species. This unorthodox reactivity mode is harnessed in the form of a “hydrogenative metathesis” reaction, which converts an enyne substrate into a cyclic alkene. The intervention of ruthenium carbenes formed in the actual *gem*‐hydrogenation step was proven by the isolation and crystallographic characterization of a rather unusual representative of this series carrying an unconfined alkyl group on a disubstituted carbene center.

## Graphical abstract

The hydrogenation of enynes with [(NHC)(cymene)RuCl_2_] (NHC=N‐heterocyclic carbene) complexes under UV irradiation leads to the formation of cyclic alkenes through the intervention of Grubbs‐type ruthenium complexes. This unorthodox entry into the realm of metathesis chemistry represents the first light‐driven catalyst system that is able to instigate alkyne *gem*‐hydrogenation with concomitant formation of a discrete metal carbene species.

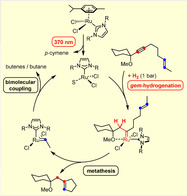

It is textbook knowledge that the hydrogenation of an unsaturated substrate over a transition metal catalyst proceeds in a suprafacial manner as long as it does not follow a radical path; *cis*‐addition of the H‐atoms to the π‐system is the necessary outcome.^1^ This stereochemical paradigm was only recently challenged when our group reported that [Cp*Ru]‐based catalysts engage internal alkynes into a *trans*‐hydrogenation reaction with *direct* 2 formation of *E*‐alkenes.^3^, ^4^, ^5^, ^6^ Combined experimental and computational investigations showed that this perplexing outcome can actually be reached by two different yet intertwined pathways (Scheme 1): once the substrate and H_2_ are uploaded onto the ruthenium fragment as shown in **A**, a first hydrogen delivery leads to a ruthenacyclopropene **B**.^4^, ^5^ It is at this stage that the route bifurcates because either end of this intermediate can participate in the subsequent hydrogen transfer step: reaction at C_α_ provides the *E*‐alkene **F** via **E** in a concerted fashion, whereas delivery at C_β_ generates a pianostool ruthenium carbene **C** in the first place (“*gem*‐hydrogenation”); this species then evolves into **F** by an associative mechanism, in which a second molecule of H_2_ has to ligate the metal center to lower the barriers.^4^, ^5^ For 2‐butyne, the two pathways have essentially identical barrier heights; substrates carrying propargylic heteroatom substituents able to coordinate the ruthenium center, in contrast, are (strongly) biased for *gem*‐hydrogention.^4^, ^5^, ^6^

**Scheme 1:**
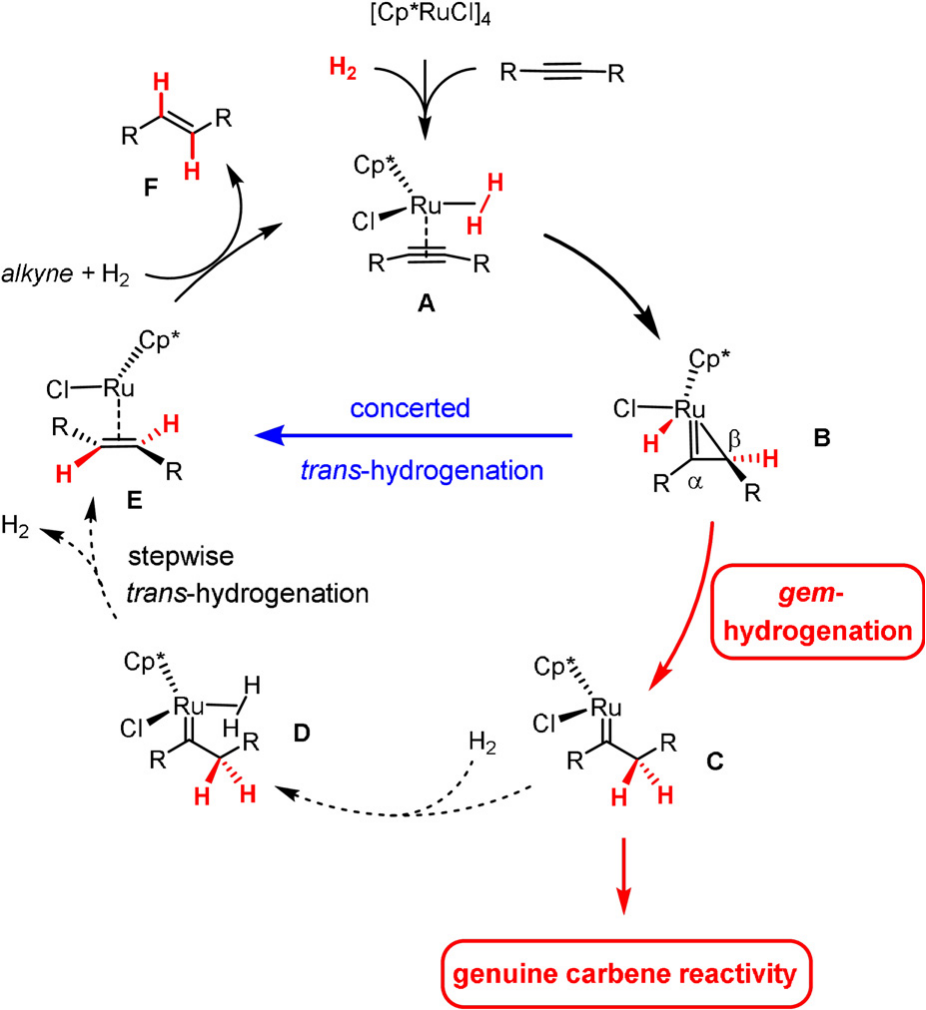
The interwoven pathways of *trans*‐ and *gem*‐hydrogenation of alkynes; the dotted lines indicate those steps downstream of *gem*‐hydrogenation that need to be outperformed in order to divert the pathway towards genuine carbene reactivity. Cp*=pentamethylcyclopentadienyl.

Prior to this discovery, the formation of discrete metal carbene complexes by *gem*‐hydrogenation was unknown.^6^ If one is able to outperform the (facile) downstream evolution of **C** via **D** into the *E*‐alkene **F**, this unorthodox reactivity mode opens intriguing new vistas for hydrogenation at large. For the time being, we managed to use *gem*‐hydrogenation as an entirely new gateway to genuine carbene chemistry in the form of hydrogenative ring expansion reactions,^4^ hydrogenative heterocycle syntheses,^4^, ^7^ and a counterintuitive hydrogenative cyclopropanation process.^4^, ^8^, ^9^ Moreover, it powers the “hydrogenative metathesis” of enynes into cycloalkenes by intervention of pianostool ruthenium carbenes of type **G** (Scheme 2);^8^ the secondary carbene **H** released in the actual metathesis step is then re‐converted into [Cp*RuCl] as the propagating species by (hydrogenolytic) cleavage of the organic ligand.^8^ As such, “hydrogenative metathesis” must be distinguished from traditional enyne metathesis which leads to a 1,3‐diene as the product;^10^ from the conceptual viewpoint, this transformation hence represents a novel reactivity mode in the realm of metathesis.^11^

**Scheme 2:**
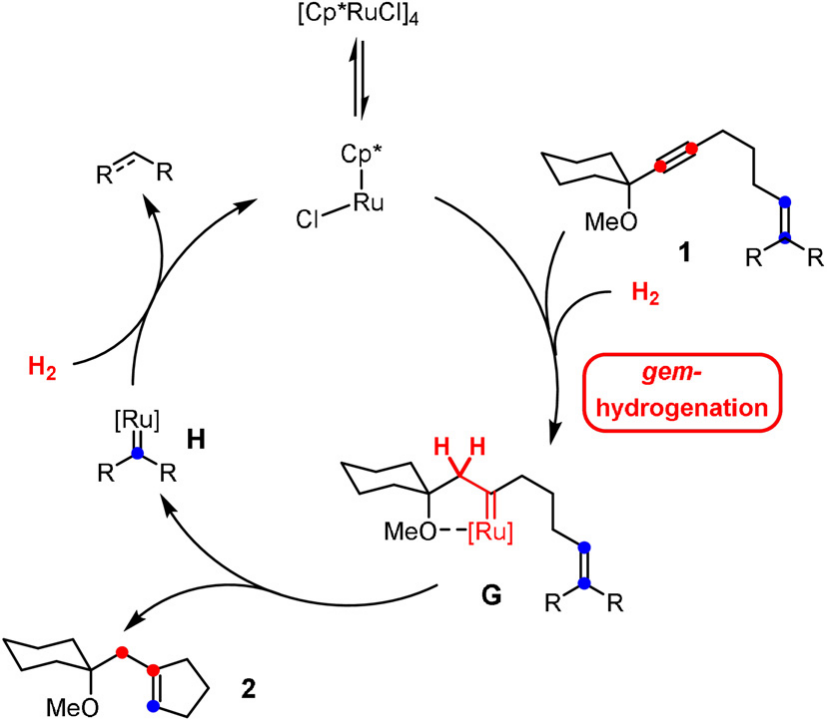
The prototype “hydrogenative metathesis” reaction involving pianostool ruthenium carbene intermediates. [Ru]=Cp*RuCl.

Proof‐of‐concept notwithstanding, a number of serious limitations remain to be addressed:^8^ (i) only enynes with a steering ‐OR substituent at the propargylic position were found to be adequate substrates; **1** (R=Me) is representative; (ii) even amongst this class of compounds, only tertiary propargyl alcohol derivatives led to good yields, whereas their secondary cousins reacted much less cleanly; (iii) the alkene site of the substrate must be trisubstituted; (iv) for the propensity of the [Cp*Ru] fragment to engage in η^6^‐binding with arenes, compounds containing (electron rich) aromatic rings basically failed to react under the standard conditions; (iv) because the metathetic ring closure needs to outperform the facile transformation of the carbene intermediate into the *E*‐alkene (see above), hydrogenative metathesis is essentially limited to the formation of five‐membered rings; even the kinetically only slightly less favorable closure of cyclohexene derivatives proved challenging and required ligand tuning.^8^

On top of this list comes the fundamental question whether or not *gem*‐hydrogenation by [Cp^X^Ru] complexes is a singularity. Although we were able to demonstrate that the reaction is responsive to changes of the electronic character of the Cp^X^ ring,^8^, ^12^ it is perplexing that the truly massive literature on catalytic hydrogenation gathered in over a century of intense research does not know of any other catalyst that instigates this type of transformation. From the preparative viewpoint, the possibility to change the ligand sphere and/or the central metal would arguably be of high significance. In a first foray into this uncharted territory, our search was focused on ancillary ligands other than Cp^X^ with the hope of gaining a better understanding for *gem*‐hydrogenation in general while overcoming some of the limitations of “hydrogenative metathesis” outlined above.

Our quest was guided by the perception that any alternative catalyst should provide—in analogy to the successful [Cp*Ru] fragment—(up to) three binding sites preferentially in a facial array for proper upload of substrate and H_2_ (as σ‐complex or, after oxidative insertion, in form of two hydride ligands).^4^, ^5^, ^13^ Careful balancing of the electron density at the central metal was deemed yet another critically important aspect: the catalyst must be notably carbophilic and π‐acidic to ensure the necessary activation of the triple bond;^14^ at the same time, however, the ligand sphere should push electron density into the hydrides to facilitate attack onto the co‐ligated π‐system. It was hoped that such a local electronic asymmetry about the central atom might be crafted by an appropriate combination of ligands with different *trans*‐influence.

With these notions in mind, a small set of precatalysts was screened (Table 1) and a first hit was obtained with complex **3 a** (L=SIMes);^15^ however, it required authentic **2** ^8^ to prove that the crude mixture formed upon hydrogenation of **1 a** (R=H) with **3 a** (10 mol %) under concomitant UV irradiation ≈8 % (NMR) of the desired metathesis product. These conditions were chosen because (photochemical) de‐coordination of *p*‐cymene from a complex of type **3** vacates three binding sites and the released 12‐electron fragment [(SIMes)RuCl_2_] comprises two ligands of largely different donor character.^16^

**Table 1:** Hit finding and reaction optimization. 
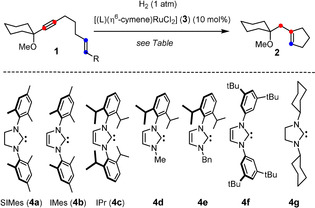

Entry	R	L	Solvent	*T* [°C]	Conditions^[a]^	Yield (%)^[b]^
1	H	SIMes (**4 a**)	1,2‐DCE	ca. 50^[c]^	UV‐A	8 (n.d.)
2	H	IMes (**4 b**)	1,2‐DCE	ca. 50^[c]^	UV‐A	25 (n.d.)
3	H	IMes (**4 b**)	toluene	ca. 50^[c]^	UV‐A	66 (56)
4	Me	IMes (**4 b**)	toluene	ca. 50^[c]^	UV‐A	85 (79)
5	Me	IPr (**4 c**)	toluene	ca. 50^[c]^	UV‐A	quant. (95)
6	Me	IPr (**4 c**)	toluene	60	dark	traces
7	Me	IPr (**4 c**)	toluene	110	dark	traces
8	Me	**4 d**	toluene	ca. 50^[c]^	UV‐A	traces
9	Me	**4 e**	toluene	ca. 50^[c]^	UV‐A	traces
10	Me	**4 f**	toluene	ca. 50^[c]^	UV‐A	traces
11	Me	**4 g**	toluene	ca. 50^[c]^	UV‐A	traces
12	Me	PCy_3_	toluene	ca. 50^[c]^	UV‐A	traces

[a] UV‐A refers to irradiation with a lamp with an emission maximum at 370 nm; for details, see the Supporting Information. [b] NMR yield (isolated yield of analytically pure product). [c] Circa 50 °C is the temperature reached upon irradiation of the mixture; no external heating bath was used. 1,2‐DCE=1,2‐dichloroethane, n.d.=not determined.

Subsequent optimization revealed the following facts and trends: (i) NHC's with an unsaturated backbone gave better results than their saturated cousins;^17^, ^18^ (ii) the nature of the N‐substituent is also important even though its exact role remains to be elucidated:^19^ for the time being, [(IPr)(η^6^‐cymene)RuCl_2_] (**3 c**) works best, followed by [(IMes)(η^6^‐cymene)RuCl_2_] (**3 b**); the use of related NHC's (**4 d**–**g**) as ancillary ligands resulted in failure;^20^ (iii) likewise, replacement of the NHC by R_3_P (R=Cy, Ph) brings hydrogenative metathesis to a hold; (iv) constant irradiation of the reaction mixture with a UV lamp is necessary;^21^ attempts to drive the reaction by thermal de‐coordination of the *p*‐cymene ligand led to mixtures that contain only minute amounts of the desired product;^22^ (v) while the [Cp*Ru] catalysts previously used operate best in chlorinated media,^8^ the switch from 1,2‐dichloroethane to toluene resulted in a significant increase in yield; (vi) just like in our previous study on *gem*‐hydrogenation,^8^ the substitution of the alkene strongly impacts on the outcome; enyne **1 b** (R=Me) bearing a methyl cap on the olefinic site proved to be optimal. When the reaction of this substrate is carried out with **3 c** (10 mol %) in toluene under H_2_ atmosphere (balloon) and irradiation with a UV lamp (*λ*=370±40 nm), the metathesis reaction is remarkably clean and essentially quantitative (see the SI), affording analytically pure **2** in 95 % yield after flash chromatography.

The products compiled in Scheme 3 were formed under these standard conditions.^23^ As expected, enynes carrying a propargylic ‐OR substituent gave good to excellent results, even if the steering group was a secondary rather than tertiary propargyl alcohol derivative. Importantly, several enynes devoid of any such propargylic substituent reacted equally well; such substrates had been beyond the scope of the original [Cp*Ru]‐based system.^8^ In case of **11**, hyperconjugation between the silicon substituent and the emerging electrophilic carbene is thought to dictate the regiochemical course of the *gem*‐hydrogenation and hence the site of metathetic ring closure, even though steric effects definitely play a role too.^24^ This notion is supported by the fact that the adamantyl derivative **12** was equally formed as a single regioisomer in essentially quantitative yield: note that the transient carbene must have resided distal to the bulky caged substituent. Likewise, an electron rich and bulky ferrocene moiety entails regioselective ring closure to give **13**, again via transient carbene formation at the alkyne's distal site. In contrast, the triple bond of (substituted) phenyl acetylenes is not sufficiently biased on electronic and/or steric grounds: as a result, *gem*‐hydrogenation occurs at either end, thus leading to product mixtures, independent of whether the phenyl group carries an electron‐donating or ‐withdrawing substituent (**14 a**–**d**). Importantly, however, these and several other examples shown in Scheme 3 demonstrate that the new catalyst system works well in the presence of aromatic groups, even if they are electron rich. The fact that six‐membered rings can also be closed with appreciable yields marks yet another notable step forward. This progress notwithstanding, attempted formation of larger carbo‐ and heterocycles has so far basically met with failure.

**Scheme 3:**
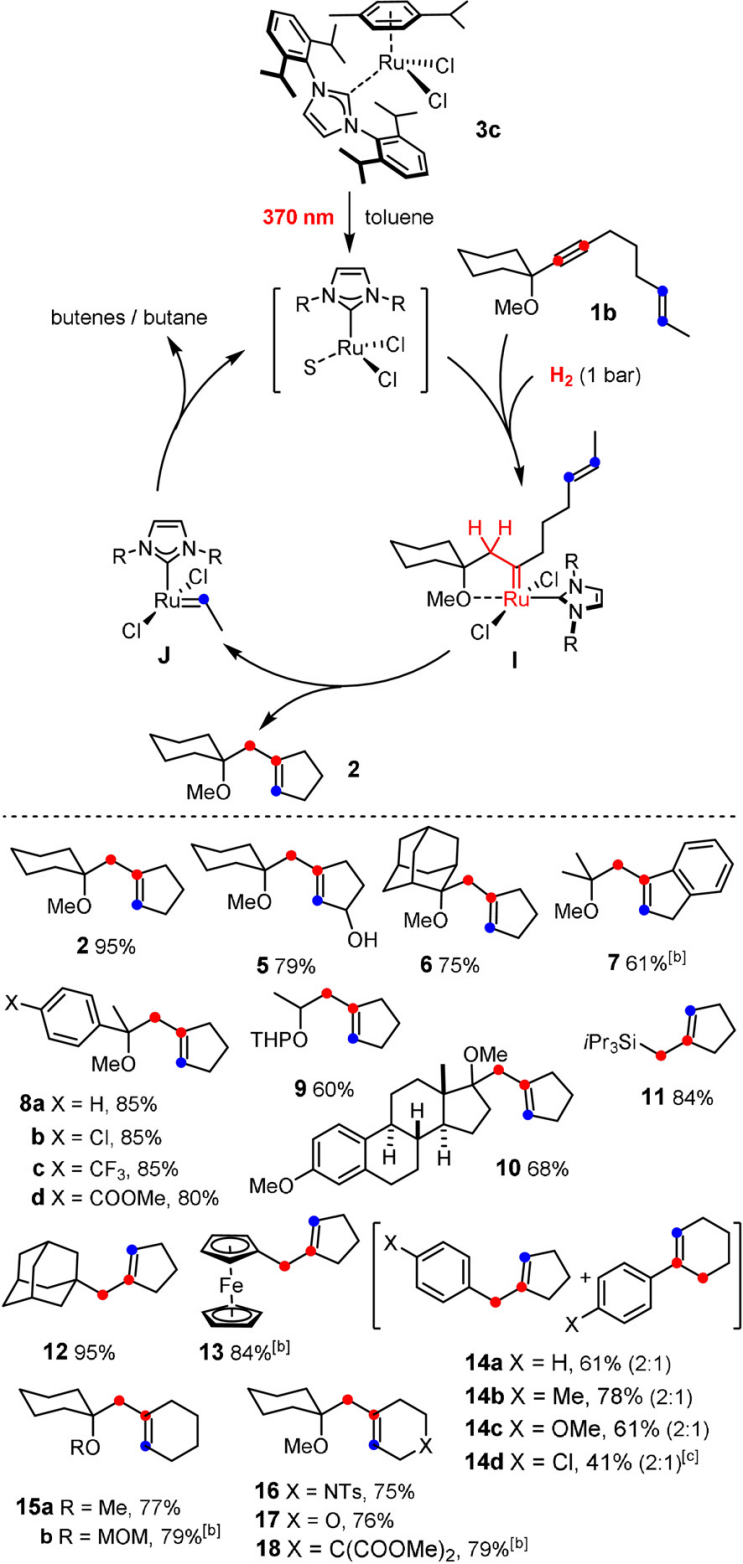
Light‐driven hydrogenative metathesis of enyne substrates: proposed catalytic cycle and an assortment of cycloalkene products. [a] All reactions were carried out under H_2_ atmosphere with [(IPr)(η^6^‐cymene)RuCl_2_] (**3 c**, 10 mol %) in toluene under constant UV irradiation. Unless stated otherwise, the yields refer to analytically pure products. [b] Using 20 mol % of the catalyst. [c] NMR yield. S=solvent, MOM=methoxymethyl, THP=tetrahydropyranyl, Ts=*p*‐toluenesulfonyl (tosyl).

Although these data suggest that Grubbs‐type carbenes of type **I** are generated in situ by alkyne *gem*‐hydrogenation as the actual reactive intermediates,^11^, ^25^, ^26^ additional experimental support was sought to corroborate this claim. Since the use of *para*‐H_2_, which had proven very useful for the study of the reactivity of [Cp*Ru]‐based catalysts, is to no avail in the present case,^4^, ^5^, ^27^ other control experiments were carried out to confirm or disprove the intervention of discrete ruthenium carbene intermediates in the new reaction set‐up. To this end, alkyne **19** was hydrogenated under UV‐irradiation with a stoichiometric amount of **3 b** (Scheme 4): NMR inspection of the crude mixture showed a diagnostic signal at δ_C_=314.5 ppm; the expected carbene complex **20** could indeed be isolated in analytically pure form. The modest yield of 31 % is mainly due to competing dimerization of **19** with formation of diene **21**, most likely by oxidative cyclization and subsequent hydrogenolytic cleavage of the resulting metallacycle.^28^ The formation of **20** does prove that a net *gem*‐hydrogenation of the triple bond of the alkyne must have taken place that leads to the formation of a genuine five‐coordinate “second generation” Grubbs‐type carbene complex.^29^ Actually, **20** seems to be the first example of a fully characterized Grubbs‐type catalyst carrying two substituents on the carbene unit, one of which is an *unconfined alkyl* group.^30^, ^31^, ^32^ Although the transient formation of such species can be inferred from the literature,^11^ **20** shows that such complexes do not degrade by the common decomposition pathways such as 1,2‐hydride or 1,2‐alkyl shift which plague non‐stabilized electrophilic metal carbenes otherwise.^33^ Complex **20** is stable under the photochemical conditions under which it is formed and decomposition by bimolecular coupling of the carbene was not observed to any noticeable extent; once isolated, it can be stored on the bench for extended periods of time and can be used in lieu of the commercial “second generation” Grubbs or Grubbs‐Hoveyda catalysts^34^ as proven by the RCM reaction leading to product **23**.

**Scheme 4:**
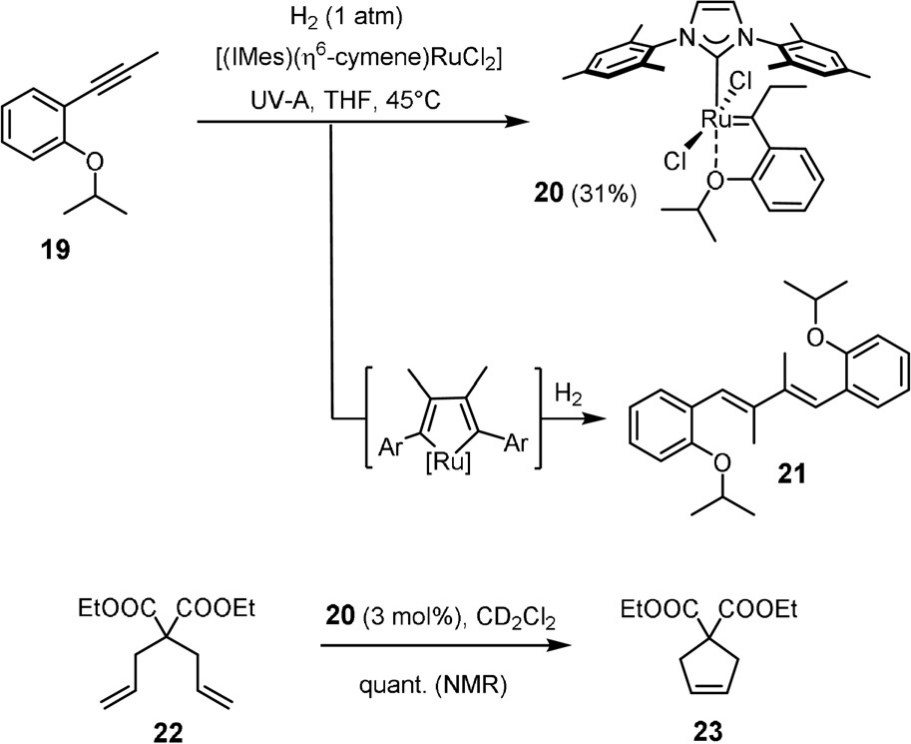
Light‐driven *gem*‐hydrogenation with formation of a Grubbs–Hoveyda‐type catalyst.

The structure of **20** in the solid state deserves particular attention (Figure 1).^35^ Since coordination of the tethered ether‐O‐atom onto the Ru center locks the carbene in place, the ethyl substituent is forced to reside directly under the umbrella of one of the two mesityl groups. Even though this *N*‐substituent turns about the N1‐C25 bond in order to provide more space (C1‐N1‐C25‐C30 97.9(1)°, C1‐N1‐C25‐C26 89.0(1)°) and the entire NHC ligand tilts away from linearity (N1‐C1‐Ru1 136.0(1)°, N2‐C1‐Ru1 119.6(1)°),^36^ the clash between the ethyl group and the ancillary ligand is massive: the distances between H11a and C25, C26, C31 and H31a are (well) below the sum of the van‐der‐Waals radii of these atoms. The strong puckering of the oxa‐ruthenacycle is yet another consequence of the crowded situation (see the SI). The strain manifested in these structural attributes is thought to be a major reason why the formation of this particular complex by *gem*‐hydrogenation is rather low‐yielding; future ligand design must take these issues into consideration.

**Figure 1:**
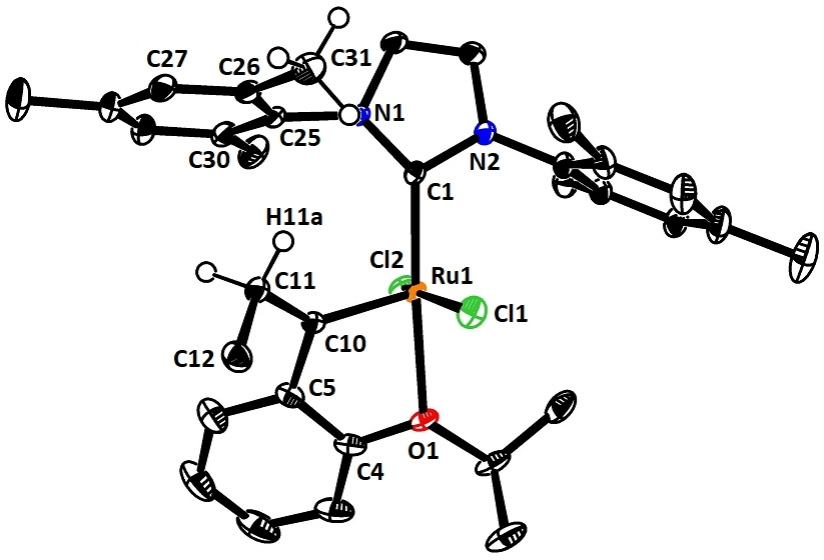
Structure of complex **20** in the solid state; arbitrary numbering scheme.^35^

As mentioned above, “hydrogenative metathesis” of an enyne involves two different metal carbenes (Scheme 3): the first such species formed by *gem*‐hydrogenation of the alkyne unit reacts with the alkene to give the metathesis product with concomitant release of a secondary carbene. For the catalytic cycle to be closed, this secondary carbene must be reconverted into the propagating ruthenium species at a rate that prevents side reactions from occurring: if it accumulates, the process transmutes into an ordinary enyne metathesis furnishing a 1,3‐diene as the product. In case of the [Cp*Ru]‐based system originally disclosed by our group (Scheme 2), hydrogenolytic cleavage of the organic ligand is responsible for the regeneration of the active catalyst.^8^ For the new light‐driven method, however, all evidence suggests that bimolecular coupling rather than hydrogenolytic cleavage of the carbene moiety is the major pathway (Scheme 5). Specifically, the secondary carbene **J** derived from **1 a** is a ruthenium methylidene, but head‐space analysis showed that ≈82 % of the released gas consists of ethene and ethane; in analogy, the internal alkene **1 b** leads to a ruthenium ethylidene, but C_4_ compounds dominate amongst the decomposition products.^38^ The fact that the amount of C_3_ products in the headspace increased significantly (at the expense of the C_2_ and C_4_ products) when a 1:1 mixture of **1 a** and **1 b** was reacted under standard conditions is particularly indicative. Regeneration of the active catalyst by this bimolecular process may also explain why the degree of substitution of the olefin in the substrate is linked to the overall efficiency of the hydrogenative metathesis reaction: with one methyl group, the secondary carbene **J** is sufficiently stabilized to render the dimerization clean in order not to lose too much catalyst per turn‐over,^39^ but it is neither over‐stabilized nor overcrowded to impede the reaction. In any case, the results of the headspace analysis are in excellent accord with studies into the decomposition pathways of Grubbs catalysts which unveiled the ease of bimolecular methylidene coupling of intermediates of type [(SIMes)(Cl_2_)Ru=CH_2_].^39^, ^40^, ^41^ Computational data corroborate the experimental results in that the corresponding transition state was found to be remarkably low‐lying.^42^ At the *meta*‐level, it is therefore interesting to note that the role of bimolecular coupling is fundamentally different in the present context: rather than being the chief destructive element,^39^, ^40^, ^41^, ^42^ this process has become the key enabling feature of the catalytic cycle, without which the new light‐driven hydrogenative metathesis reaction would not be able to proceed.

**Scheme 5:**
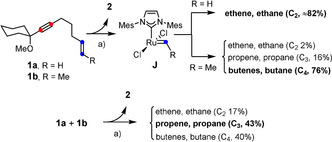
Headspace analysis showing the fate of the secondary carbene formed in the actual metathesis step: a) **3 b** (10 mol %), H_2_ (1 atm), UV‐A, toluene.

In line with the notion that bimolecular coupling is quintessential for maintaining catalytic turnover, the Grubbs‐Hoveyda‐type complex **20** itself—though formed by a light‐driven *gem*‐hydrogenation event—is not a competent catalyst for hydrogenative enyne metathesis because the disubstitued carbene moiety is simply too reluctant.^43^ In contrast, the slim Grubbs ethylidene complex **24** (20 mol %)—though ligated to two additional pyridine ligands—is able to convert enyne **1 b** into product **2** in 64 % yield under the standard reaction conditions (H_2_, toluene, UV‐A light, ca. 50 °C) (Scheme 6). As this control experiment is based on an orthogonal entry point into the catalytic cycle, it provides independent support for the proposed mechanism.

**Scheme 6:**
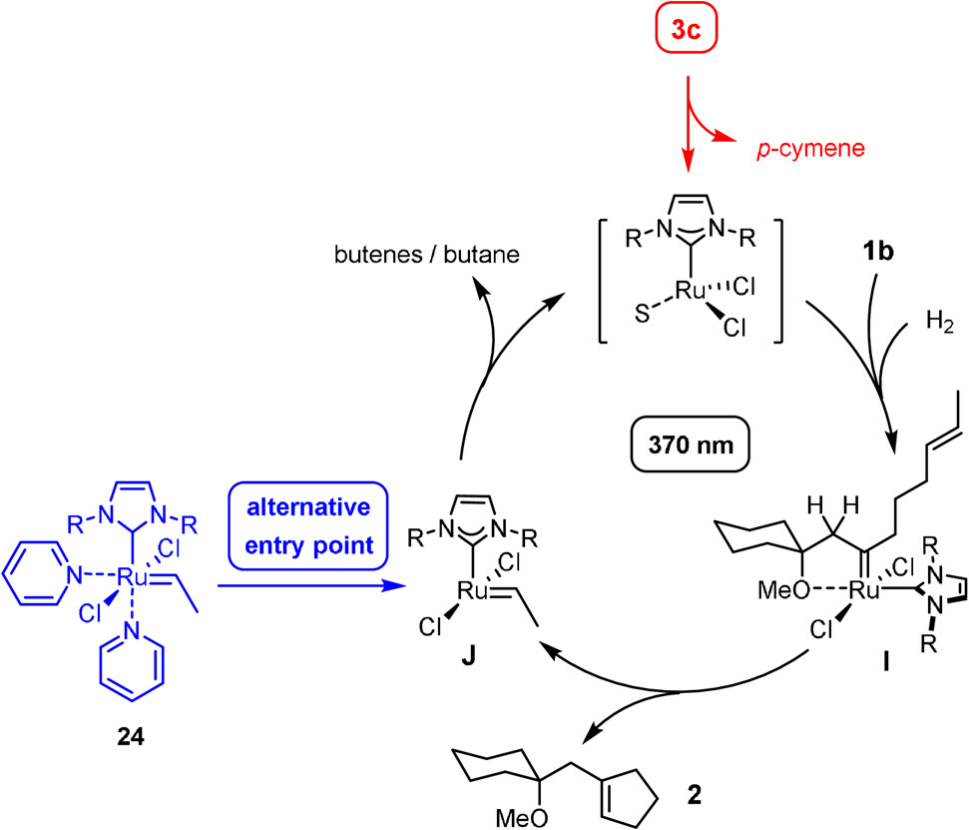
A Grubbs‐type ruthenium ethylidene complex as an alternative entry point. *R*=2,6‐diisopropylphenyl.

The reasons why the reaction mixture must be permanently irradiated with UV light in order to achieve full and clean conversion are by no means clear at this point. In accord with literature data,^16^ our control experiments confirmed that cymene decomplexation from the precatalyst **3** proceeds without problem at elevated temperatures in the dark (see the SI); hence, the role of the light is more intricate.^44^, ^45^ Recent studies, which draw an integral picture of metathesis including the fate of the catalyst might provide hints:^42^ specifically, DFT analysis showed that thermal decomplexation of the *p*‐cymene ligand from [(SIMes)(*p*‐cymene)RuCl_2_] delivers the 12‐electron fragment [(SIMes)RuCl_2_] as *spin‐singlet*; this species is extremely electron‐deficient and readily inserts into the C−H bonds of an *N*‐mesityl group of the SIMes ligand. In contrast, decomposition of a Hoveyda‐type catalyst generates the analogous *spin‐triplet* ^3^[(SIMes)RuCl_2_] which shows a notably different chemical behavior in that it preferentially engages with alkenes.^42^ It is tempting to speculate that light is needed in the new hydrogenative metathesis reaction to connect the two spin surfaces.^46^ In any case, it will be interesting to study the reactivity of the two spin‐isomers vis‐à‐vis H_2_ and alkynes. The question whether or not the current reaction is another incarnation of two‐state reactivity^47^ and other open mechanistic aspects deserve detailed scrutiny by a combined experimental and computational approach.

In summary, we document herein the only second example of a catalyst that is capable of effecting the net *geminal* delivery of the two H‐atoms of H_2_ to one and the same C‐atom of an alkyne substrate. In contrast to the only literature precedent,^4^, ^5^, ^6^, ^7^, ^8^ the new *gem*‐hydrogenation manifold is light‐driven and leads to the formation of second‐generation Grubbs‐type complexes as the key reactive intermediates. Although numerous aspects remain to be addressed and the maturity and scope of the transformation to be improved, it is clear that catalytic hydrogenation opens a non‐canonical and orthogonal entry into the cosmos of Grubbs catalysis.^11^ Further studies intending to harness the power of this and related *gem*‐hydrogenation reactions are underway in our laboratory.

## Conflict of interest

The authors declare no conflict of interest.

## Supporting information

As a service to our authors and readers, this journal provides supporting information supplied by the authors. Such materials are peer reviewed and may be re‐organized for online delivery, but are not copy‐edited or typeset. Technical support issues arising from supporting information (other than missing files) should be addressed to the authors.

Supplementary
