# Supplementary material for: Grubbs Metathesis Enabled by a Light‐Driven gem‐Hydrogenation of Internal Alkynes
Source: Angew Chem Int Ed Engl. 2020 Aug 20;59(42):18423–9. doi: 10.1002/anie.202007030 (PMC7589215; doi:10.1002/anie.202007030)
Supplement: Supplementary file 1 — Supplementary [file ANIE-59-18423-s001.pdf]

Angewandte Chemie
Eine Zeitschrift der Gesellschaft Deutscher Chemiker

Supporting Information

Grubbs Metathesis Enabled by a Light-Driven *gem*-Hydrogenation of Internal Alkynes

*Tobias Biberger, Raphael J. Zachmann, and Alois Fürstner**

anie_202007030_sm_miscellaneous_information.pdf

Contents

1. General Remarks 2
2. Mechanistic Studies 4
   2.1 Synthesis of a Hoveyda-Grubbs Carbene by *gem*-Hydrogenation 4
   2.2 Kinetic Profiling of Complex 20 16
   2.3 Hydrogenative Metathesis with H_2/D_2 mixtures 17
   2.4 Headspace Analysis 19
   2.6 The Impact of the UV Irradiation on the Reaction Outcome 31
   2.7 Control Experiment: Orthogonal Entry via a Grubbs Ethylidene Complex 32
3. Synthesis of Substrates and Precatalysts 34
   3.1 Synthesis of the Substrates 34
   3.2 Synthesis of the Precatalysts 46
4. Hydrogenative Metathesis Reactions 47
5. Copies of Spectra 53
6. References 104

1. General Remarks

All reactions were carried out in flame-dried glassware under argon, ensuring rigorously inert conditions. The solvents were purified by distillation over the indicated drying agents and were stored and handled under argon: CH_2Cl_2 (CaH_2), MeCN (CaH_2), pentane (Na/K alloy), THF (Na/K alloy).

NMR spectra were recorded on Bruker AV400 or AV500 spectrometers at 298 K unless otherwise indicated, with the chemical shift (*δ*) given in ppm relative to TMS and the coupling constants (*J*) in Hz. The solvent signals were used as references[1] and the chemical shifts converted to the TMS scale (CD_2Cl_2: δ_H = 5.32 ppm, δ_C = 53.8 ppm; [D_3]-acetonitrile: δ_H = 1.94 ppm, δ_C = 118.26 ppm; [D_6]-acetone: δ_H = 2.05 ppm, δ_C = 29.8 ppm; C_6D_6: δ_H = 7.16 ppm, δ_C = 128.06 ppm).

Unless stated otherwise, all commercially available compounds (abcr, Acros, TCI, Aldrich, Alfa Aesar) were used without further purification.

Hydrogen gas (N50, ≥99.999 Vol.%) was purchased from AirLiquide and was used without further purification. Deuterium gas (99.8 atom% D, 99.995% purity) was purchased from SigmaAldrich. Both hydrogen and deuterium were handled with standard balloon techniques.

IR spectra were recorded on Alpha Platinum ATR spectrometer (Bruker) at room temperature, wavenumbers ($\tilde{\nu}$) are given in cm^{-1}.

Mass spectrometric samples were analyzed using the following instruments: MS (EI): Finnigan MAT 8200 (70 eV), ESI-MS: Bruker ESQ3000, accurate mass determinations: Bruker APEX III FT-MS (7 T magnet) or MAT 95 (Finnigan).

GC-MS was performed with a Shimadzu GCMS-QP2010 Ultra instrument.

Headspace GC-FID samples were measured on an Agilent Technology 6890 or 7890 instrument with a 30 m HP-plot Al_2O_3 column (0.25 mm ∅, 5 µm film) using H_2 as the carrier gas. Headspace GC/MS samples were analyzed on an Agilent Technology 7890A instrument with AT 5975C MSD detection.

Photolysis experiments were performed in a self-made apparatus (Figure S-1), consisting of an aluminum box with a circular arrangement of 8 UV-A lamps (Philips Fluorescent lamps TUV PL-S 9W/2P, λ = 340-380 nm (Figure S-2) at 6 cm distance to the quartz-Schlenk tube. The temperature in the apparatus typically rises to 45 °C over the course of 1 h.

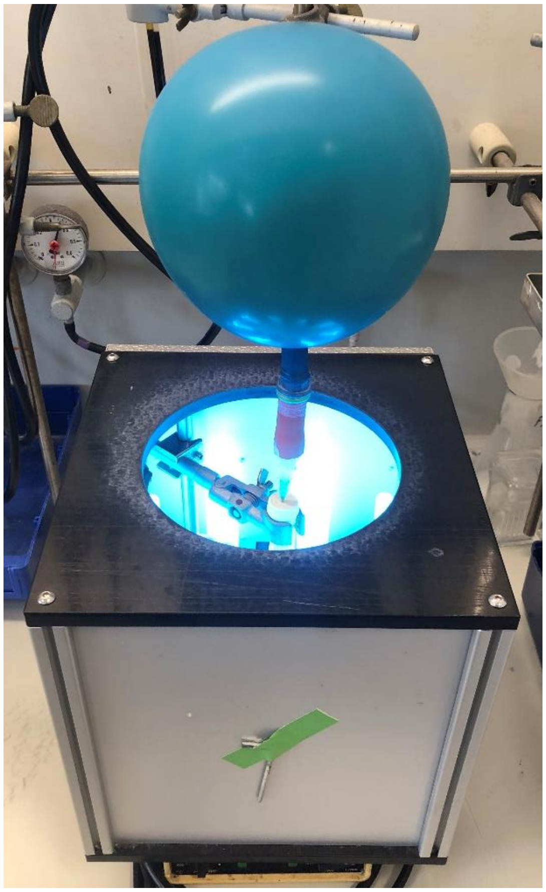

Figure S-1. Reaction set-up

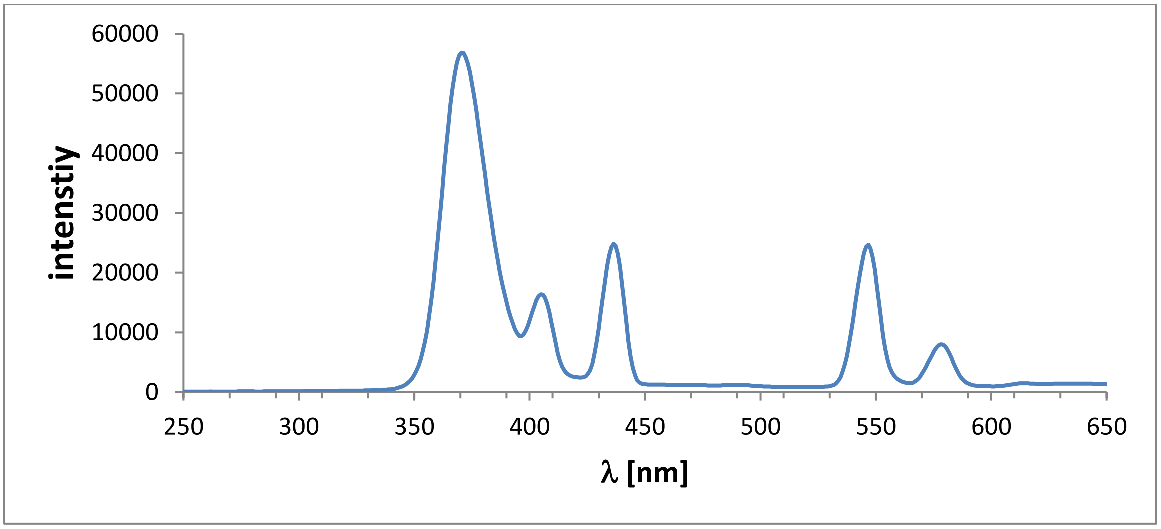

Figure S-2. Emission spectrum of the UV lamps

2. Mechanistic Studies

2.1 Synthesis of a Hoveyda-Grubbs Carbene by *gem*-Hydrogenation

1-Isopropoxy-2-(prop-1-yn-1-yl)benzene (19)

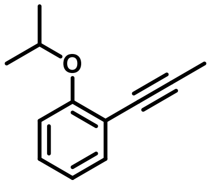

1-Ethynyl-2-isopropoxybenzene (250 mg, 1.56 mmol)[2] was dissolved in THF (5 mL) and the resulting solution cooled to –78 °C. *n*-BuLi (1.6 M in hexanes, 1.07 mL, 1.71 mmol) was added dropwise over 5 min and the mixture was stirred for 30 min. Methyl iodide (147 µL, 2.35 mmol) was added and stirring was continued at room temperature for 15 h. Sat. aq. NH_4Cl (4 mL), *tert*-butyl methyl ether (10 mL) and water (2 mL) were added and the layers were separated. The aqueous layer was extracted with *tert*-butyl methyl ether (3 × 20 mL) and the combined organic layers were washed with brine and dried over Na_2SO_4. The solvent was removed under reduced pressure and the residue was purified by flash chromatography (silica, pentane/*tert*-butyl methyl ether 100:1) to provide the title compound as a colorless oil (200 mg, 74%). ^{1}H NMR (400 MHz, $CDCl_3$) δ 7.36 (dd, *J* = 7.9, 1.8 Hz, 1H), 7.22 – 7.12 (m, 1H), 6.93 – 6.83 (m, 2H), 4.55 (hept, *J* = 6.1 Hz, 1H), 2.09 (s, 3H), 1.37 (d, *J* = 6.1 Hz, 6H). ^{13}C NMR (101 MHz, $CDCl_3$) δ 158.8, 133.8, 128.8, 120.9, 115.5, 89.7, 76.3, 72.1, 22.3, 4.8. IR (film) $\tilde{\nu}$ 2977, 1594, 1482, 1444, 1383, 1372, 1262, 1102, 974, 748 cm^{-1}. HRMS (ESI$^+$) for $C_{12}H_{14}O$ [M]$^+$: calcd. 174.10388; found: 174.10392.

Complex 20.

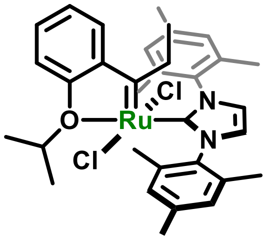

A flame-dried quartz Schlenk tube was charged under argon with [(IMes)(*p*-cymene)$RuCl_2$] (102 mg, 167 µmol), alkyne **19** (28.2 mg, 167 µmol) and THF (6 mL). The Schlenk tube was closed with a septum and then transferred into the photolysis apparatus (Figure S-1). A hydrogen-filled balloon was connected to a needle which was pierced through the septum. The Schlenk tube was flushed with hydrogen for 2 min through an outlet cannula (the cannula did not reach into the solution to ensure that only the head space of the tube was flushed). After the first 10 seconds of flushing with hydrogen, the light source was switched on and the reaction mixture was stirred for 30 min under hydrogen atmosphere. After cooling to room temperature, all volatile components were removed under high vacuum. The remaining crude material was suspended in pentane (10 mL) and the suspension was vigorously stirred for

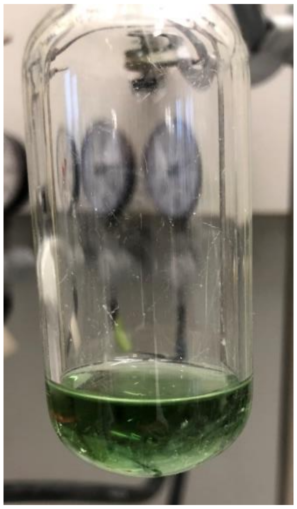

5 min. The supernatant was removed via a filter canula and the dark green filtrate was collected. This trituration procedure was repeated twice and the combined filtrates were evaporated. The remaining material was purified by flash chromatography (silica, CH_2Cl_2→ CH_2Cl_2/MeOH 25:1). The green band was collected and the solvent evaporated to provide the title compound (34.1 mg, 31%) as a dark green oil. Dark green needle-shaped crystals suitable for X-ray diffraction were grown by slow evaporation of CH_2Cl_2/pentane solutions in a constant stream of argon.

^{1}H NMR (600 MHz, C_6D_6) δ 7.35 (dd, *J* = 7.7, 1.6 Hz, 1H), 7.10 (ddd, *J* = 8.5, 7.3, 1.6 Hz, 1H), 6.88 (s, 4H), 6.60 (td, *J* = 7.7, 7.3, 0.9 Hz, 1H), 6.43 (d, *J* = 8.3 Hz, 1H), 6.17 (s, 2H), 4.57 (hept, *J* = 6.2 Hz, 1H), 2.62 (q, *J* = 7.5 Hz, 2H), 2.40 (s, 12H), 2.20 (s, 6H), 1.12 (d, *J* = 6.2 Hz, 6H), 0.97 (t, *J* = 7.5 Hz, 3H). ^{13}C NMR (151 MHz, C_6D_6) δ 314.5, 174.9, 154.6, 146.7, 138.9, 138.5, 136.0, 129.0, 128.5, 124.6, 121.0, 114.3, 112.4, 74.3, 51.5, 21.2, 20.8, 19.5, 6.2. IR (film) $\tilde{\nu}$ 2961, 2921, 2865, 1573, 1483, 1450, 1395, 1304, 1259, 1224, 1160, 1078, 1012, 936, 922, 850, 791, 751, 698, 665, 614, 593, 579 cm^{-1}. HRMS (ESI$^+$) for $C_{33}H_{40}Cl_2N_2ORu$ $[M]^+$: calcd. 652.15553; found: 652.15557.

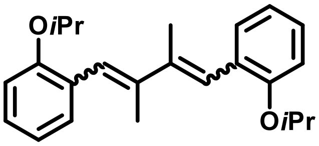

A second fraction collected during flash chromatography consisted of compound **21** (colorless oil, 16.1 mg, 55%). ^{1}H NMR (600 MHz, $CDCl_3$) δ 7.30 (dd, *J* = 7.5, 1.8 Hz, 1H), 7.21 (td, *J* = 7.7, 1.8 Hz, 1H), 6.97 (td, *J* = 7.5, 1.1 Hz, 1H), 6.93 (dd, *J* = 8.2, 1.2 Hz, 1H), 6.86 (s, 1H), 4.50 (hept, *J* = 6.0 Hz, 1H), 2.14 (s, 2H), 1.36 (d, *J* = 6.1 Hz, 5H). ^{13}C NMR (151 MHz, $CDCl_3$) δ 156.1, 138.3, 131.0, 129.4, 127.6, 123.2, 120.3, 114.8, 71.3, 22.3, 15.9. IR (film) $\tilde{\nu}$ 2975, 2931, 1594, 1481, 1449, 1383, 1372, 1287, 1238, 1120, 957, 749 cm^{-1}. HRMS (ESI$^+$) for $C_{24}H_{30}O_2Na$ $[M]^+$: calcd. 373.21349; found: 373.21380.

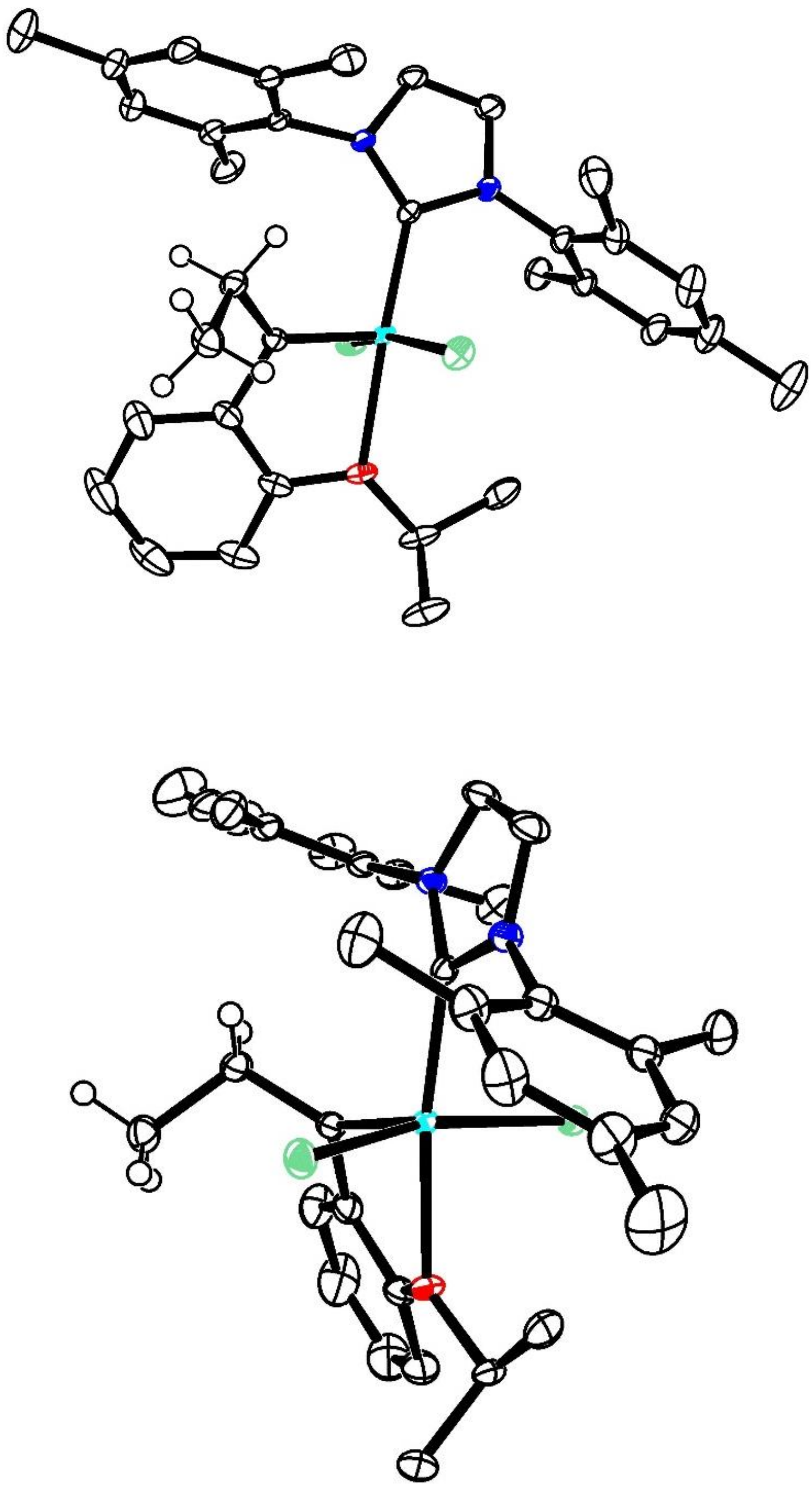

Figure S-3. Structure of complex **20** in the solid state in two different orientation; hydrogen atoms (except on the ethyl substituent) are omitted for clarity

X-ray Crystal Structure Analysis of Complex 20: $C_{33} H_{40} Cl_2 N_2 O$ Ru, M_r = 652.64 g · mol^{-1}, green plate, crystal size 0.127 x 0.067 x 0.013 mm^3, triclinic, space group $P\bar{1}$ [2], a = 8.5880(5) Å, b = 11.2933(6) Å, c = 17.1714(9) Å, α = 99.455(2)°, β = 92.749(2)°, γ = 107.076(2)°, V = 1562.16(15) Å^3, T = 100(2) K, Z = 2, D_{calc} = 1.387 g · cm^3, λ = 0.71073 Å, μ(*Mo-K*$_\alpha$) = 0.701 mm^{-1}, analytical absorption correction ($T_{\min}$ = 0.95, $T_{\max}$ = 0.99), Bruker-AXS Kappa Mach3 diffractometer with APEX-II detector and IµS micro focus X-ray source, 1.208 < θ < 33.141°, 67324 measured reflections, 11891 independent reflections, 10420 reflections with $I > 2\sigma(I)$, R_{int} = 0.0411. S = 1.046, 361 parameters, residual electron density +1.5 (0.83 Å from Si3) / –0.7 (0.72 Å from Si3) e · Å^{-3}. The structure was solved by *SHELXT* and refined by full-matrix least-squares (*SHELXL*) against F^2 to R_1 = 0.032 [$I > 2\sigma(I)$], wR_2 = 0.073. **CCDC-2004044**.

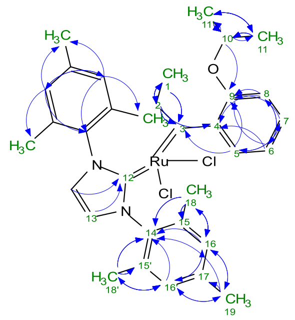

ROESY cross correlations
HMBC cross peaks

Atom	δ (ppm)	J	COSY	HSQC	HMBC	NOESY
1 C	6.18			1	2	
H3	0.97	7.50(2)	2	1	2, 3	2, 5
2 C	51.54			2	1	
H2	2.62	7.50(1)	1	2	1, 3, 4	1, 5
3 C	314.51				1, 2, 5	
4 C	146.73				2, 6, 8	
5 C	114.26			5	7	
H	7.35	1.60(7), 7.70(6)	6	5	3, 7, 9	1, 2, 6
6 C	121.02			6	8	
H	6.60	7.70(5), 7.30(7)	5, 7	6	4, 8, 9	5, 7
7 C	128.50			7	5	
H	7.10	1.60(5), 8.20(8), 7.30(6)	6, 8	7	5, 9	6, 8
8 C	112.36			8	6	
H	6.43	8.20(7)	7	8	4, 6, 9	7, 10, 11, 11'
9 C	154.58				5, 6, 7, 8, 10	
10 C	74.33			10	11, 11'	
H	4.57	6.20(11'C), 6.20(11)	11, 11'	10	9, 11, 11'	8, 11, 11'
11 C	21.16			11	10, 11'	
H3	1.12	6.20(10)	10	11	10, 11'	8, 10

Atom	δ (ppm)	J	COSY	HSQC	HMBC	NOESY
11' C	21.16	6.20(10H)		11'	10, 11	
H3	1.12		10	11'	10, 11	8, 10
12 C	174.84				4, 13	
13 C	124.57			13	13	
H	6.17			13	12, 13	
14 C	136.01				16, 16', 18, 18', 19'	
15 C	138.45				18	
15' C	138.45				18'	
16 C	129.02			16	16', 18, 19'	
H	6.88			16	14, 16', 18, 19	18, 19', 19'', 19'''
16' C	129.02			16'	16, 18', 19'	
H	6.88			16'	14, 16, 18', 19	18', 19'
17 C	138.90				19'	
18 C	19.50			18	16	
H3	2.40			18	14, 15, 16	16
18' C	19.50			18'	16'	
H3	2.40			18'	14, 15', 16'	16'
19 C	20.76			19', 19'', 19'''	16, 16'	
H3	2.20			19	14, 16, 16', 17	16, 16'

P-ID: ML0000000
Measured on: 16/01/2020
CHIFFRE: BIU-BB-560-01
ELNA#: 3477
Client: Tobias Biberger
Group: Fürstner
Spectroscopist: Leutzsch
Analysed on: 16/01/2020
Analysed by: Leutzsch
Amount: 10 mg
Solvent: C6D6
Reference: solvent
Temperature: 298 K
Spectrometer: AV600neo
Probe: Z159656_0010 (CP2.1 BBO 600S3 BB-H&F-D-05 Z XT)
Experiments: 1H-zg30, 13C-zgpg30, [13C, 1H]-hsqcedetgpsisp2.3, [1H, 1H]-clipcosy, [1H, 1H]-roesyadjsph, [13C, 1H]-hmbcetgpl3nd

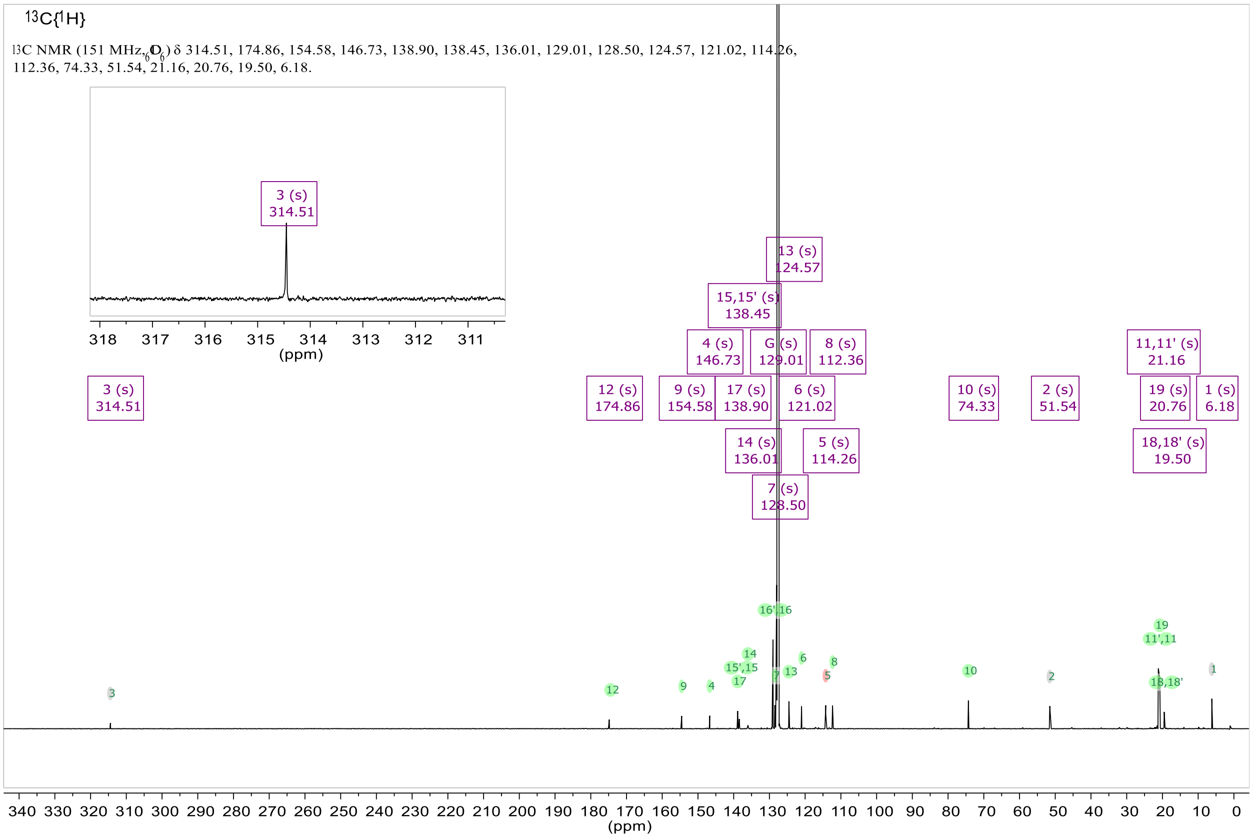
13C{1H}
13C NMR (151 MHz, C6D6) δ 314.51, 174.86, 154.58, 146.73, 138.90, 138.45, 136.01, 129.01, 128.50, 124.57, 121.02, 114.26, 112.36, 74.33, 51.54, 21.16, 20.76, 19.50, 6.18.
(ppm)

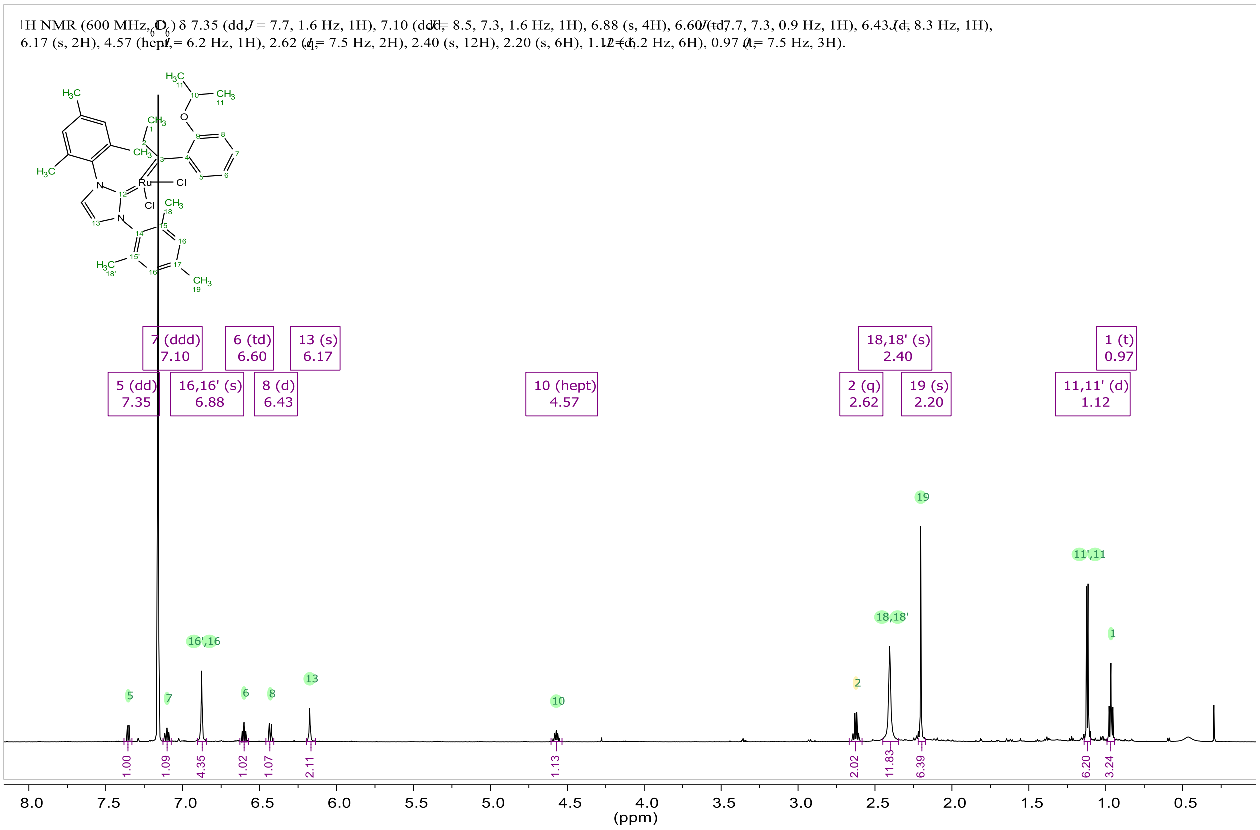
1H NMR (600 MHz, C6D6) δ 7.35 (dd, J = 7.7, 1.6 Hz, 1H), 7.10 (ddd, J = 8.5, 7.3, 1.6 Hz, 1H), 6.88 (s, 4H), 6.60 (td, J = 7.7, 7.3, 0.9 Hz, 1H), 6.43 (d, J = 8.3 Hz, 1H), 6.17 (s, 2H), 4.57 (hept, J = 6.2 Hz, 1H), 2.62 (q, J = 7.5 Hz, 2H), 2.40 (s, 12H), 2.20 (s, 6H), 1.12 (d, J = 6.2 Hz, 6H), 0.97 (t, J = 7.5 Hz, 3H).
(ppm)

^{13}C HSQC

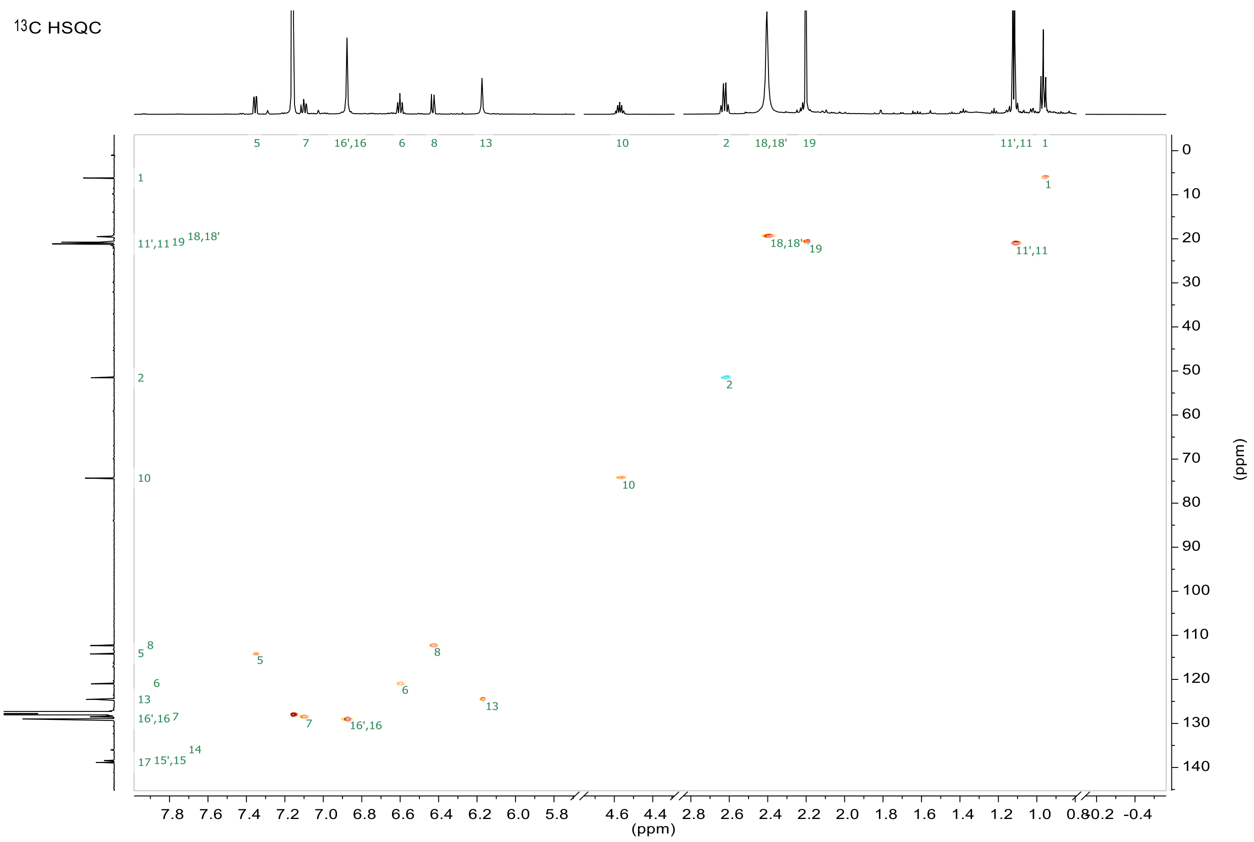

CLIP COSY

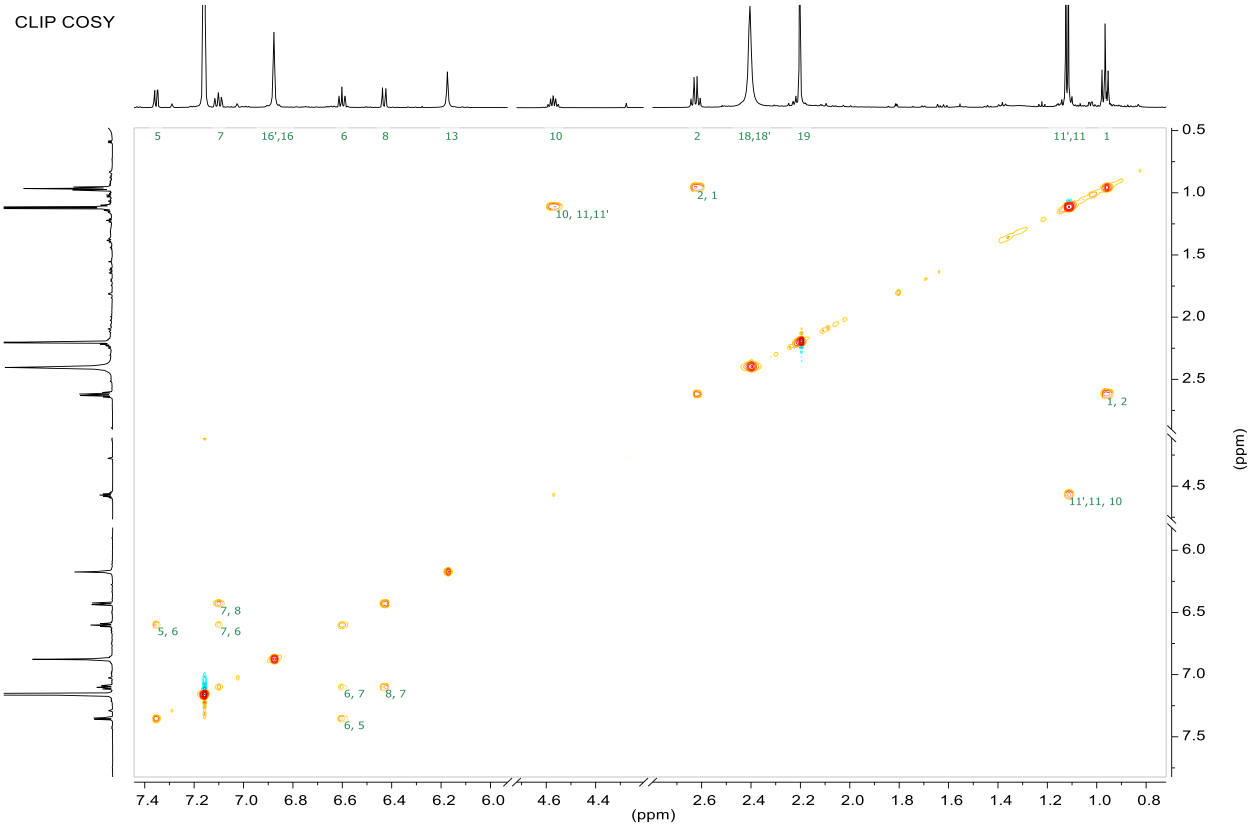

^{13}C HSQC

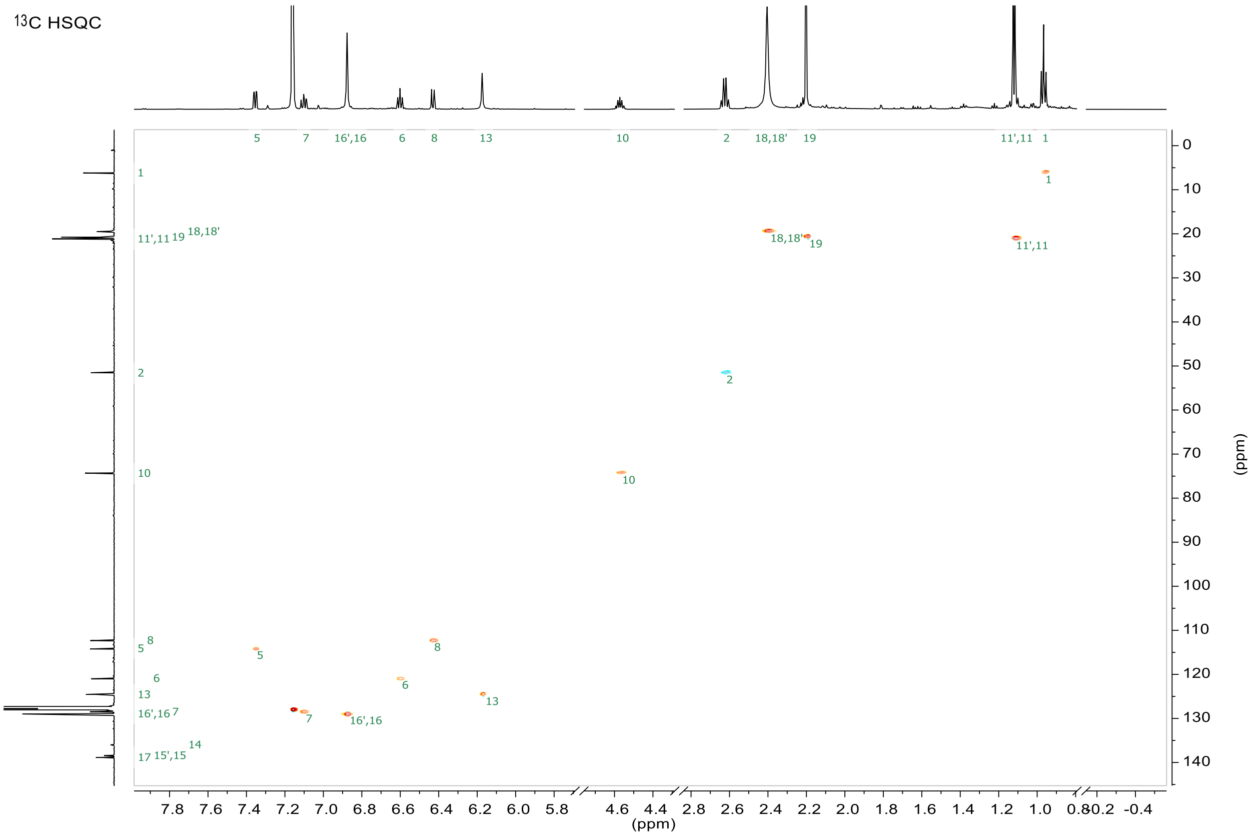

2D ROESY

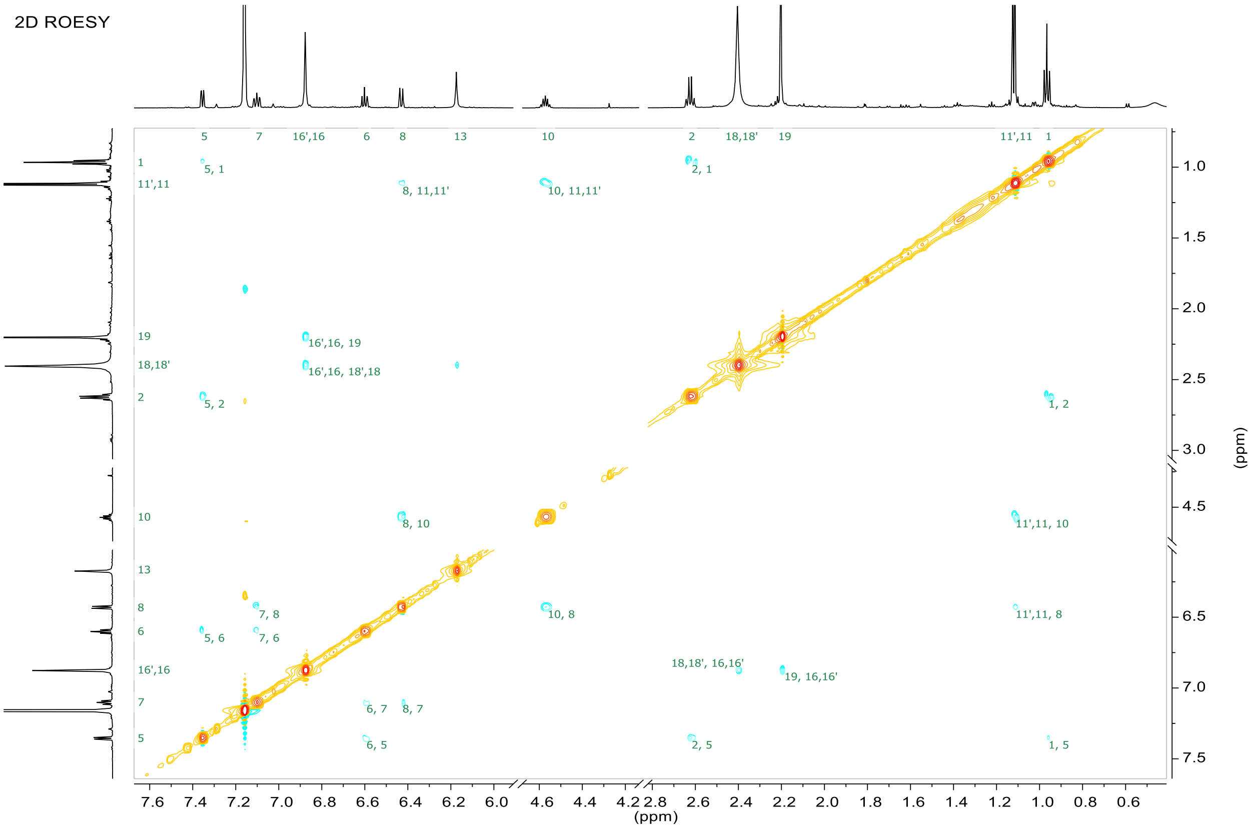

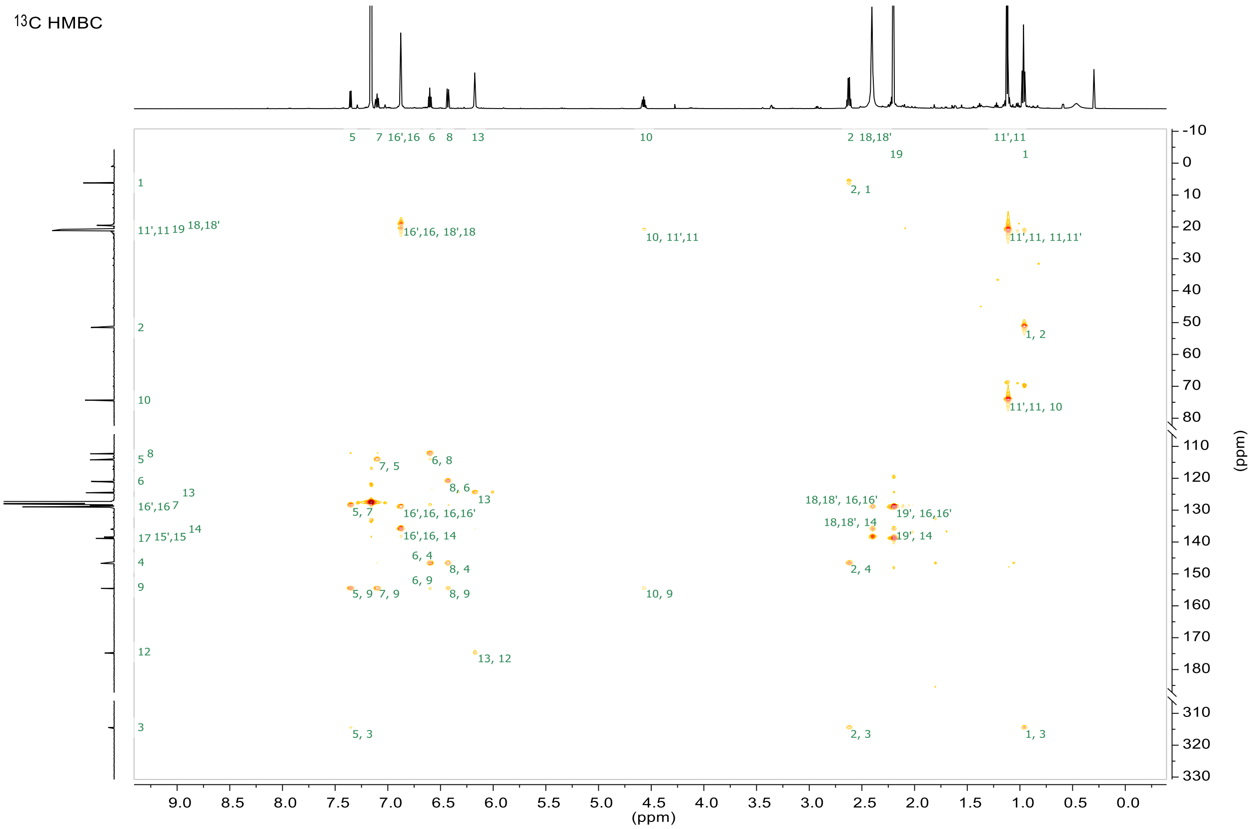

¹³C HMBC
5 7 16',16 6 8 13 10 2 18,18' 19 11',11 1
1
11',11 19 18,18'
2
10
5 8
6
13
16',16 7
17 15',15 14
4
9
12
3
2, 1
16',16, 18',18
10, 11',11
11',11, 11,11'
1, 2
11',11, 10
7, 5
6, 8
8, 6
13
5, 7
16',16, 16,16'
18,18', 16,16'
19', 16,16'
16',16, 14
18,18', 14
19', 14
6, 4
8, 4
2, 4
6, 9
5, 9 7, 9
8, 9
10, 9
13, 12
5, 3
2, 3
1, 3
9.0 8.5 8.0 7.5 7.0 6.5 6.0 5.5 5.0 4.5 4.0 3.5 3.0 2.5 2.0 1.5 1.0 0.5 0.0
(ppm)
-10 0 10 20 30 40 50 60 70 80 110 120 130 140 150 160 170 180 310 320 330
(ppm)

Side product 21

After analysis the NMR data supports the following structure:

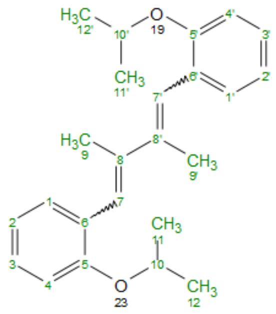

Due to the high symmetry and flexibility of the molecule, no information could be obtained about the double bond geometry. It is either all-*cis* or all-*trans*.
A Xray structure could solve that problem. Maybe EI-MS can also help to solve that question.

User Report

Assignments					
**Atom**	**Chemical Shift**	**COSY**	**HSQC**	**HMBC**	**NOESY**
1 C	130.985		1	3	
H	7.296	2	1	3, 5, 7	2, 9
1' C	130.985		1'	3', 7'	
H	7.296	2'	1'	3', 5', 7'	2', 9'
2 C	120.270		2	4	
H	6.973	1, 3	2	4, 6	1, 3
2' C	120.270		2'	4'	
H	6.973	1', 3'	2'	4', 6'	1', 3'
3 C	127.587		3	1	
H	7.211	2, 4	3	1, 5	2, 4
3' C	127.587		3'	1'	
H	7.211	2', 3', 4'	3'	1', 5'	2', 4'
4 C	114.751		4	2	
H	6.932	3	4	2, 5, 6	3, 10, 11, 12
4' C	114.751		4'	2'	
H	6.932	3'	4'	2', 5', 6'	3', 10', 11', 12'
5 C	156.048			1, 3, 4	
5' C	156.048			1', 3', 4', 7', 10'	
6 C	129.409			2, 4	
6' C	129.409			2', 4'	
7 C	123.234		7	1	
H	6.859		7	8'	9, 11, 12

Assignments					
**Atom**	**Chemical Shift**	**COSY**	**HSQC**	**HMBC**	**NOESY**
7' C	123.234		7'	1', 9, 9'	
H	6.859		7'	1', 5', 8'	9', 11', 12'
8 C	138.266				
8' C	138.266			7, 7', 9, 9'	
9 C	15.846		9		
H3	2.143		9	7', 8'	1, 7
9' C	15.846		9'		
H3	2.143		9'	7', 8'	1', 7'
10 C	71.224		10	11	
H	4.505	11, 12	10	11, 12	4
10' C	71.224		10'	11', 12'	
H	4.505	11', 12'	10'	5', 11', 12'	4'
11 C	22.231		11	10, 12	
H3	1.357	10	11	10, 12	4, 7
11' C	22.231		11'	10', 12'	
H3	1.357	10'	11'	10', 12'	4', 7'
12 C	22.231		12	10, 11	
H3	1.357	10	12	11	4, 7
12' C	22.231		12'	10', 11'	
H3	1.357	10'	12'	10', 11'	4', 7'
19 O					
23 O					

P-ID: ML00000
Measured on: 16/10/2019
CHIFFRE: BIU-BB-459-01
ELNA#: 3314
Client: Tobias Biberger
Group: Fürstner
Spectroscopist: Leutzsch
Analysed on: 16/10/2019
Analysed by: Leutzsch
Amount: 15 mg
Solvent: CDCl3
Reference: solvent
Temperature: 298 K
Spectrometer: AV600a
Probe: Z44896_0147 (CP TCI 600S3 H-C/N-D-05 Z)
Experiments: 1H-zg30, 13C-zgdc30, [13C, 1H]-ASAP_hsqcedetgpsp, [13C, 1H]-hmbcetgpl3nd, [1H, 1H]-noesygpph, [1H, 1H]-clipcosysp.cf

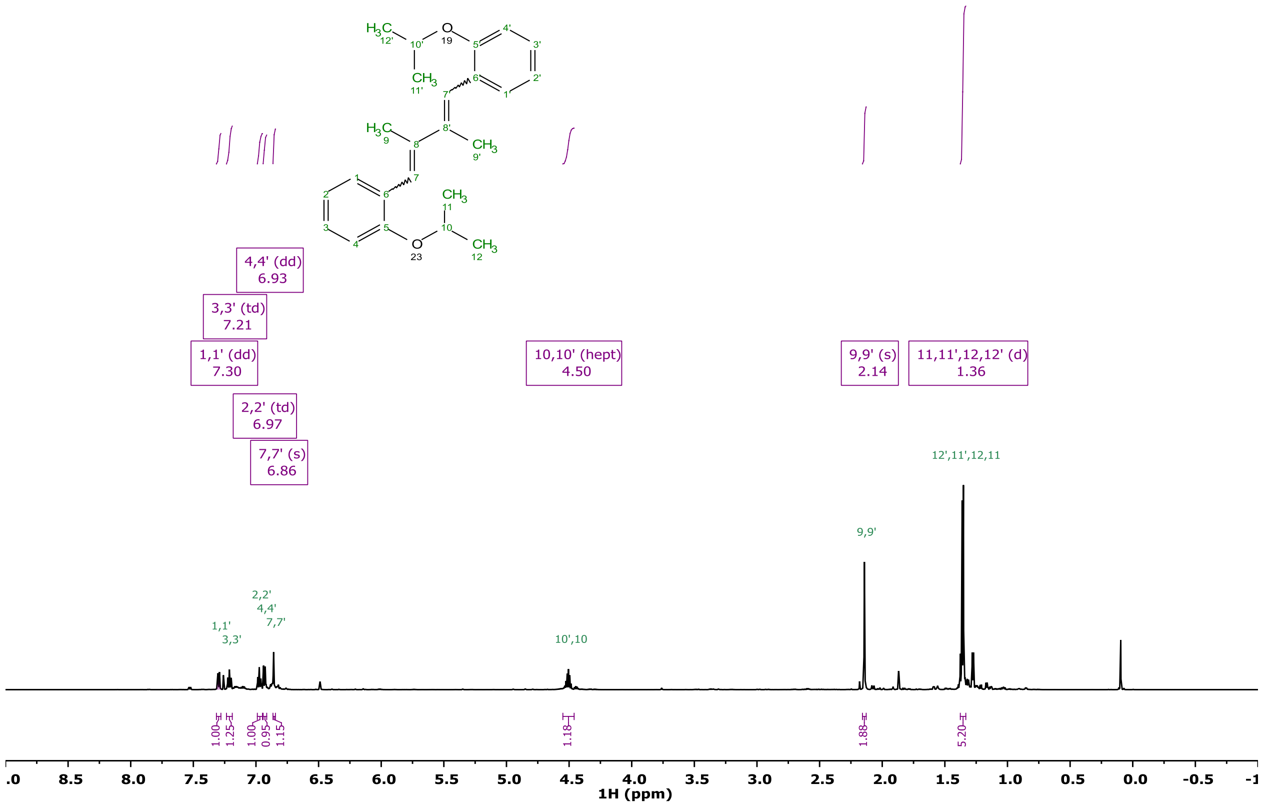
4,4' (dd)
6.93
3,3' (td)
7.21
1,1' (dd)
7.30
2,2' (td)
6.97
7,7' (s)
6.86
10,10' (hept)
4.50
9,9' (s)
2.14
11,11',12,12' (d)
1.36
12',11',12,11
9,9'
2,2'
4,4'
7,7'
1,1'
3,3'
10',10
1.00
1.25
1.00
0.95
1.15
1.18
1.88
5.20
1H (ppm)

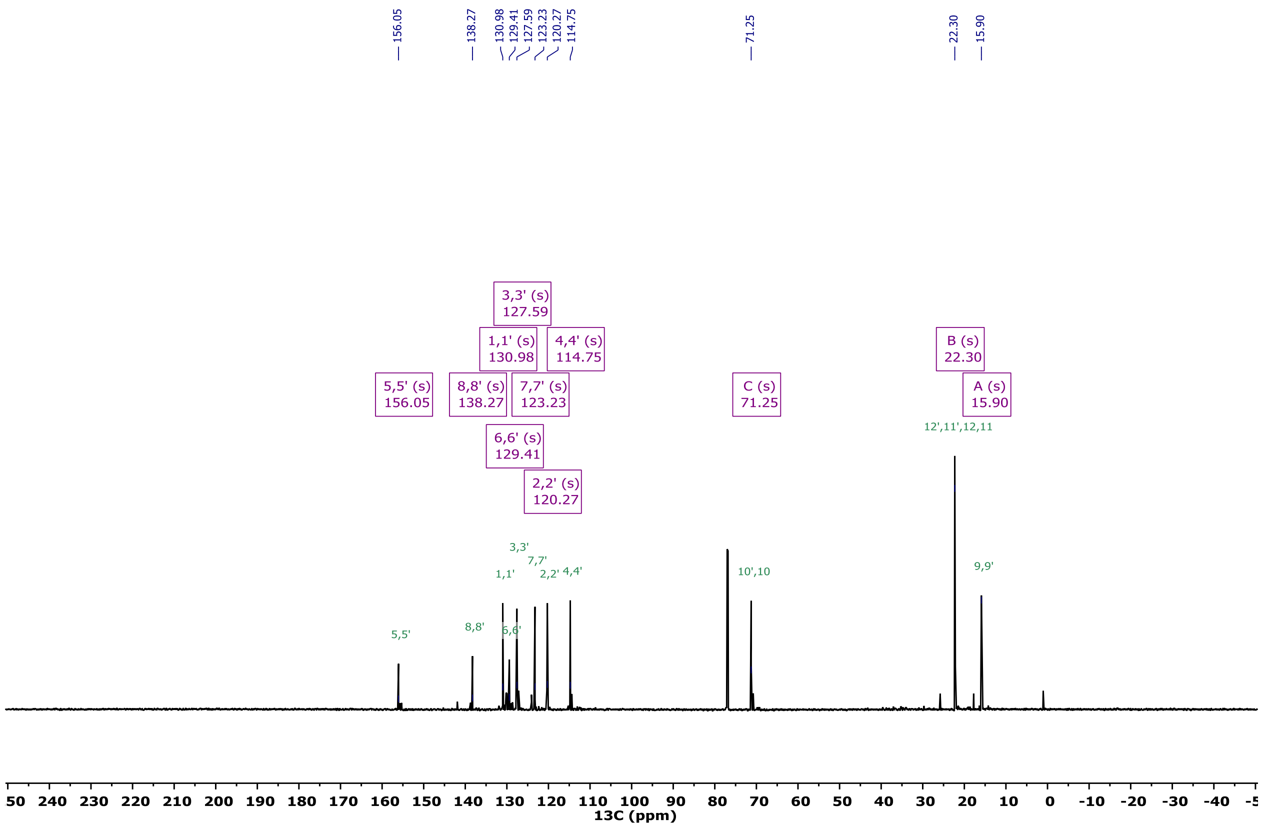
156.05
138.27
130.98
129.41
127.59
123.23
120.27
114.75
71.25
22.30
15.90
3,3' (s)
127.59
1,1' (s)
130.98
4,4' (s)
114.75
B (s)
22.30
5,5' (s)
156.05
8,8' (s)
138.27
7,7' (s)
123.23
C (s)
71.25
A (s)
15.90
6,6' (s)
129.41
2,2' (s)
120.27
12',11',12,11
3,3'
7,7'
1,1'
2,2'
4,4'
10',10
9,9'
5,5'
8,8'
6,6'
13C (ppm)

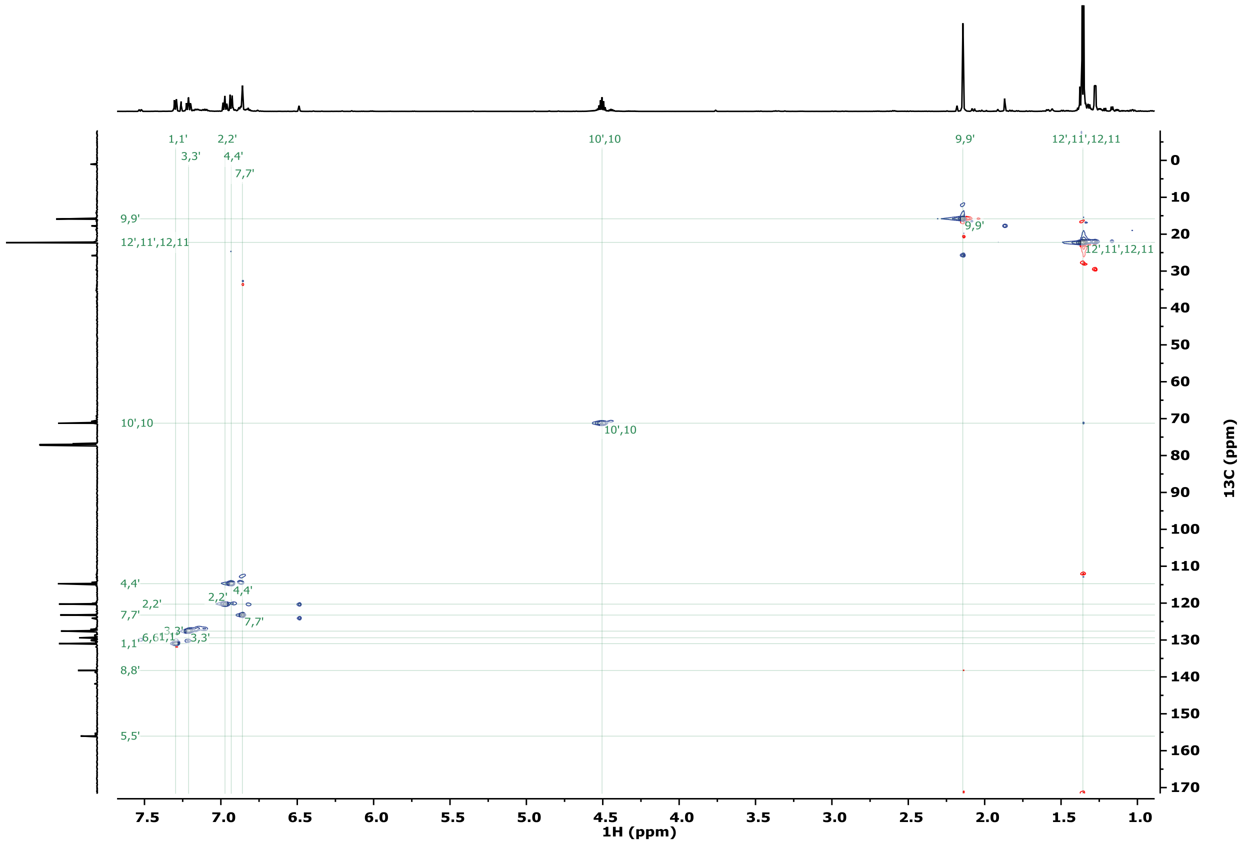
1,1'
3,3'
2,2'
4,4'
7,7'
10',10
9,9'
12',11',12,11
9,9'
12',11',12,11
10',10
4,4'
2,2'
7,7'
1,1'
8,8'
5,5'
1H (ppm)
13C (ppm)

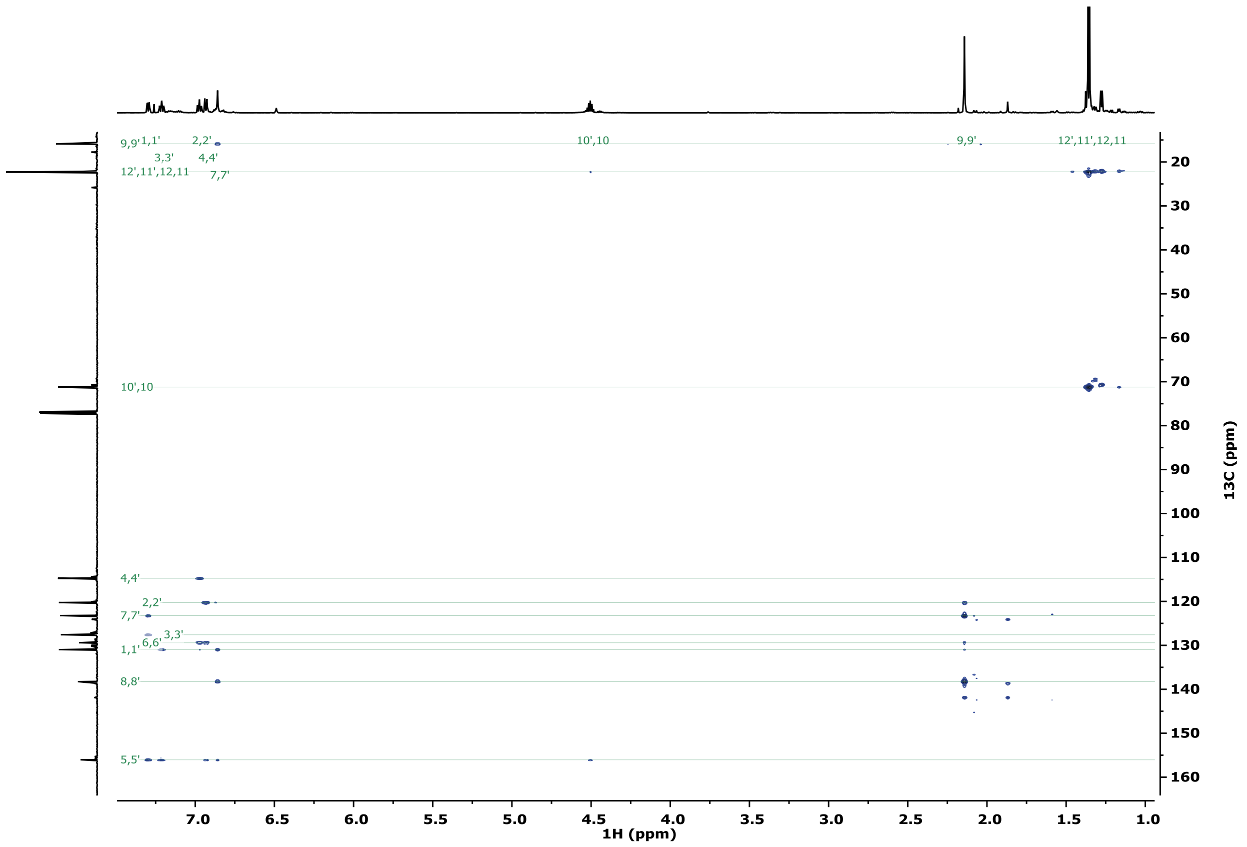
9,9'
1,1'
2,2'
3,3'
4,4'
12',11',12,11
7,7'
10',10
9,9'
12',11',12,11
10',10
4,4'
2,2'
7,7'
3,3'
1,1'
6,6'
8,8'
5,5'
1H (ppm)
13C (ppm)

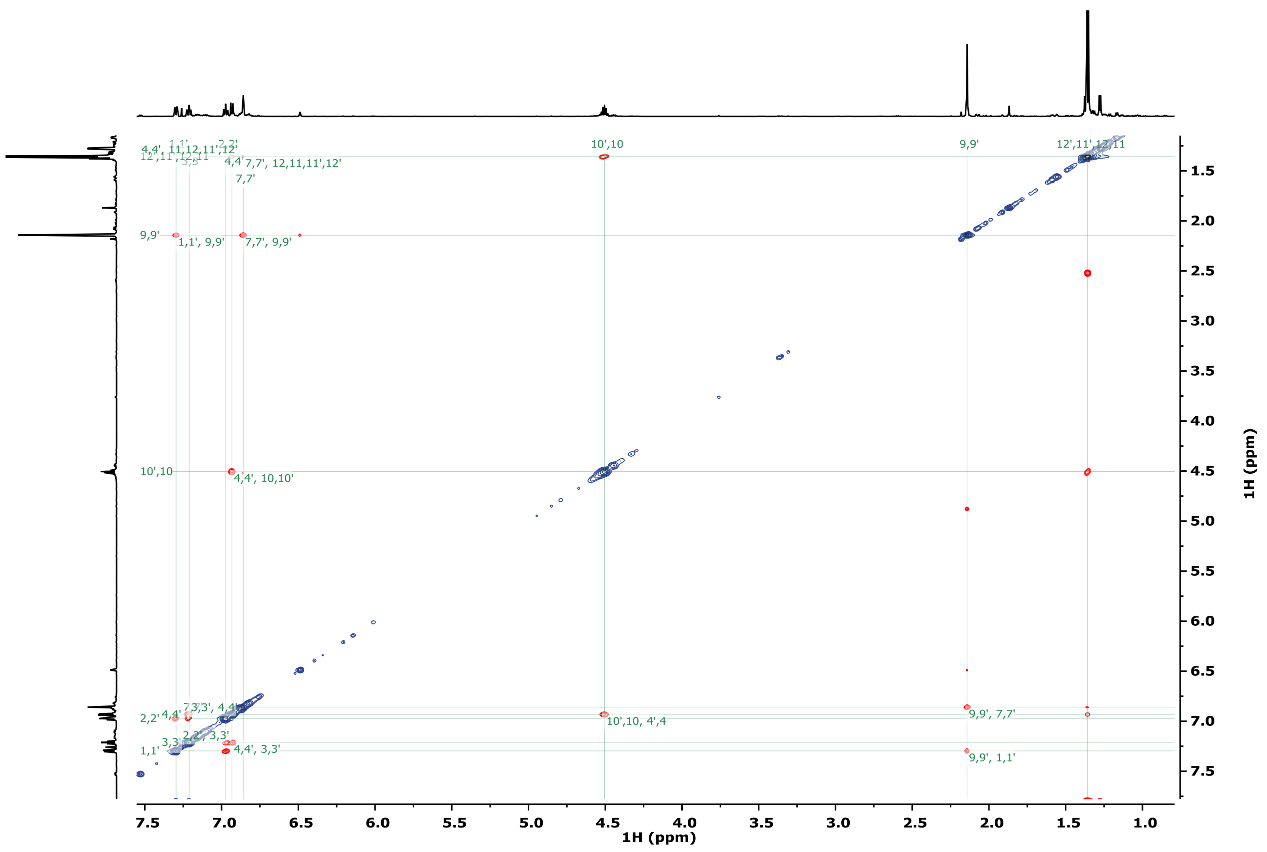
1H (ppm)
1H (ppm)
10',10
9,9'
12',11',12,11
4,4'
1,1'
2,2'
7,7'
10',10, 4',4
4,4', 10,10'
1,1', 9,9'
7,7', 9,9'
9,9', 7,7'
9,9', 1,1'
4,4', 3,3'
7,7', 12,11,11',12'

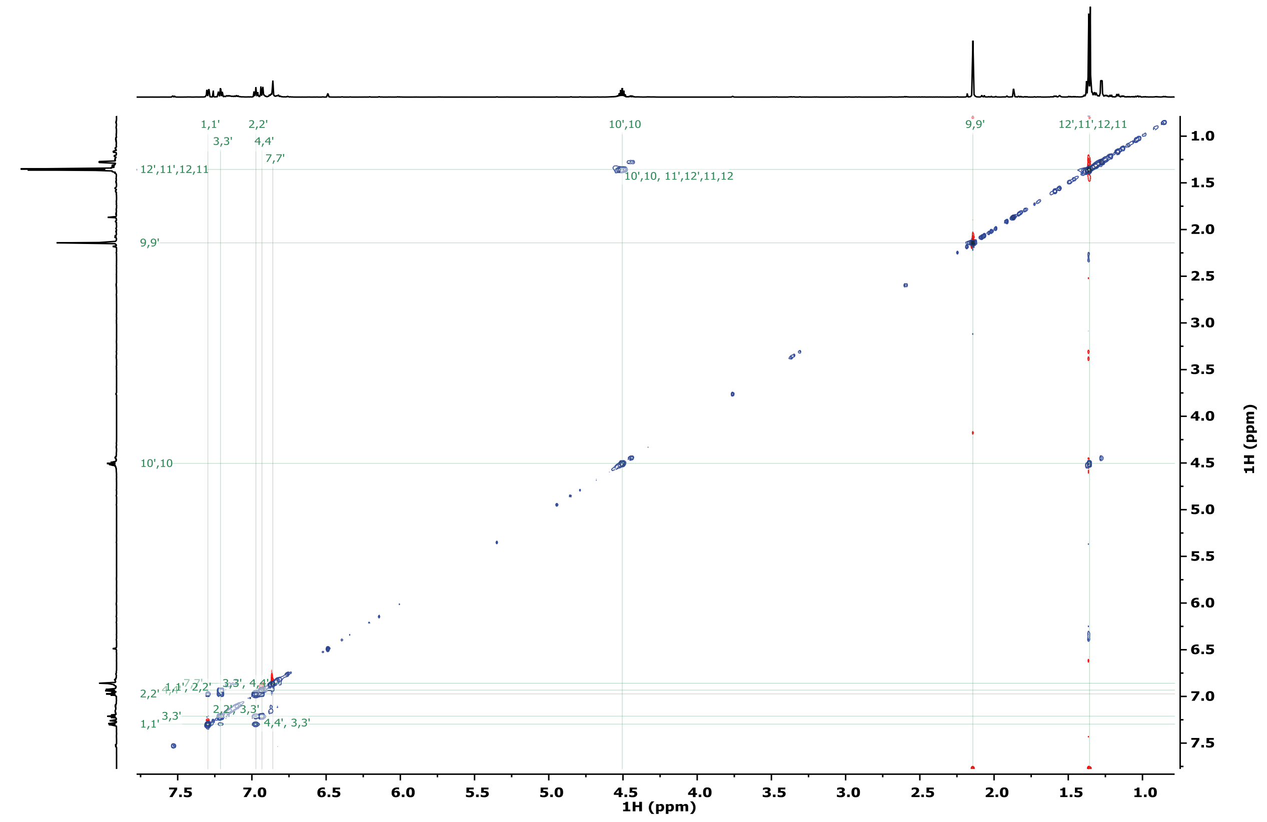
1H (ppm)
1H (ppm)
1,1'
3,3'
2,2'
4,4'
7,7'
10',10
9,9'
12',11',12,11
10',10, 11',12',11,12
4,4', 3,3'

2.2 Kinetic Profiling of Complex 20

In order to characterize complex **20** further, we made use of a standard method to assess the catalytic performance of olefin metathesis catalyst developed by Grubbs et al.[3] To this end, the ring closing metathesis of diallylmalonate (**22**) was carried out with complex **20** and the commercially available Grubbs II and Hoveyda-Grubbs II catalysts for comparison.

In an argon-filled glovebox, an NMR tube with a screw cap septum top was charged with a stock solution of the corresponding complex in CD_2Cl_2 (1 mol%, 0.5 mL). The sample was brought out of the glovebox and equilibrated at 30°C in the NMR machine before diallylmalonate **22** (12.8 µL, 53.5 µmol) was added via a Hamilton syringe. The NMR tube was quickly shaken and inserted in the NMR probe. Data points were collected over 20 mins and the acquired raw data were processed in MestReNova with the ReactionMonitoring-plugin (Figure S-4).

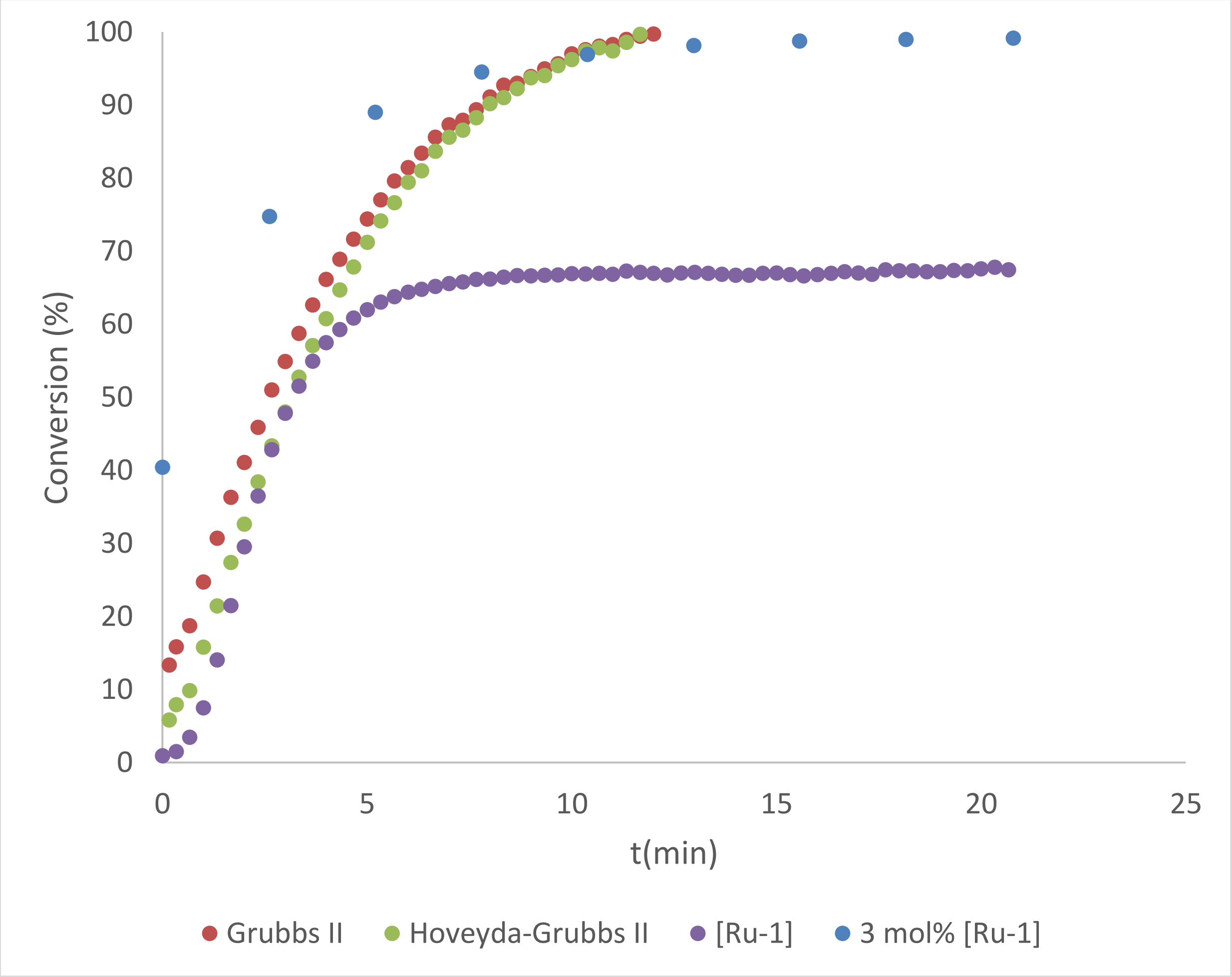

Figure S-4. RCM of diallylmalonate **22** with formation of product **23** ([Ru-1] = complex **20**)

All tested catalysts exhibit similar rates at 1 mol% catalyst loading up to 60% conversion. Figure S-4 shows that after this point the Grubbs II and the Hoveyda-Grubbs II catalyst convert the substrate quantitatively into the product, whereas the reaction catalyzed by **20** stalled at ≈60% conversion. However, this catalysts fully converts the substrate into product without losing activity at a loading of 3 mol%.

2.3 Hydrogenative Metathesis with H_2/D_2 mixtures

A 1:1 mixture of H_2 and D_2 was prepared by using a gas-tight 10 mL Hamilton syringe: a gas volume of 5 mL was taken out of a balloon filled with H_2 and of a second balloon filled with D_2: the Hamilton syringe was then shaken to ensure mixing of the two gases.

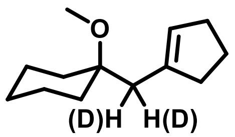

A flame-dried quartz Schlenk tube was charged with [(IPr)(p-cymene)$RuCl_2$] (13.8 mg, 200 µmol, 10 mol%), enyne **1b** (44.1 mg, 0.2 mmol) and toluene (2.0 mL, 0.1 M). The Schlenk tube was closed with a septum and then transferred into the photolysis apparatus. The total volume of 10 mL H_2/D_2 was then flushed through the quartz Schlenk tube using an outlet canula. This procedure was repeated nine times to fully exchange the argon atmosphere for a H_2/D_2 atmosphere. At this point, the light source was switched on and the mixture was stirred for 60 min. For work-up, the mixture was diluted with pentane (5 mL) and filtered through a short silica pad. The filtrate was evaporated and the remaining crude material was purified by flash chromatography (silica, pentane/*tert*-butyl methyl ether 50:1) to provide product **2** (28.1 mg, 72%) as a colorless oil. The identity and composition of the different isotopomers was established by ^{1}H and ^{13}C NMR spectroscopy.

The isotopomer ratio was determined to be [D_0]:[D_1]:[D_2] = 42:34:24. The formation of significant quantities of an [D_1] isotopomer is suggestive of a non-concerted hydrogen transfer mechanism.

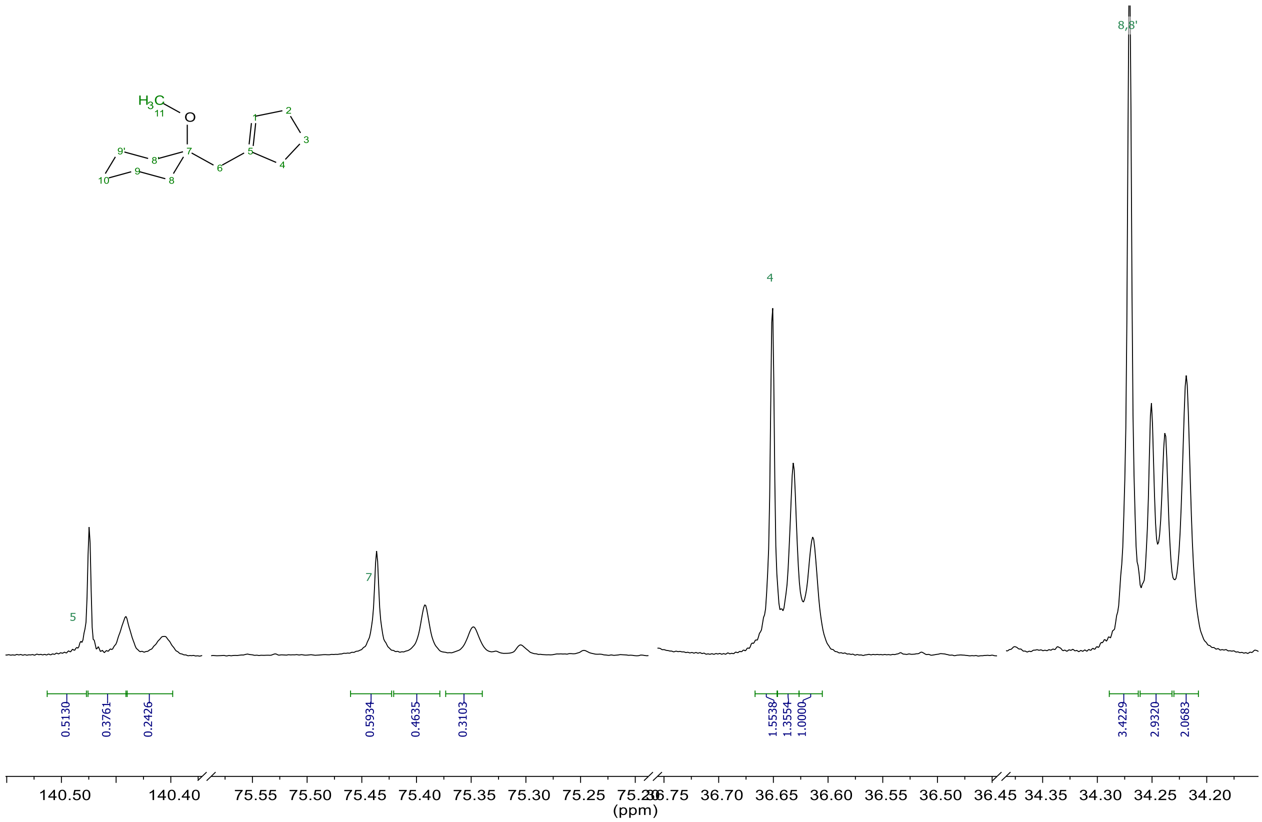

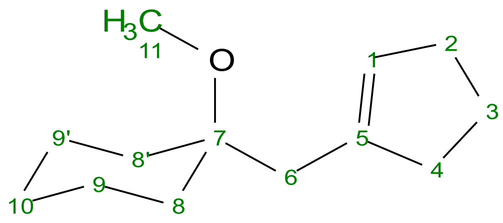

From ^{13}C	Integral			%Content		
Atom	H2	HD	D2			
1	1.8878	1.4529	1	43%	33%	23%
7	1.8184	1.4542	1	43%	34%	23%
8.8'	1.6476	1.4036	1	41%	35%	25%
4	1.5538	1.3524	1	40%	35%	26%
		Average		41.6%	34.2%	24.2%
		error		1.7%	0.6%	1.2%

2.4 Headspace Analysis

A flame-dried quartz Schlenk tube was charged with [(IPr)(p-cymene)RuCl$_{2]}$ (13.8 mg, 0.02 mmol, 10 mol%), the corresponding enyne **1a** or **1b** (200 µmol) *[for the crossover experiment, enyne **1a** and **1b** (100 µmol, each)]* and toluene (2 mL, 0.1 M). The Schlenk tube was closed with a septum and then transferred into the photolysis apparatus. A hydrogen-filled balloon was equipped with a needle which was pierced through the septum and the Schlenk tube was flushed with hydrogen for 2 min through an outlet cannula (the cannula did not reach into the solution to ensure that only the head space of the tube was flushed). After the first 10 seconds of flushing with hydrogen, the light source was switched on and the mixture was stirred for 60 min under hydrogen atmosphere. At this point, a gas-tight 1 mL Hamilton Syringe was used to take 500 µL aliquots of the gas in the headspace of the Schlenk tube and the samples were analyzed by GC. Retention times and fragmentation patterns of authentic samples of various gases (Phillips No.40 + methane, ethane and ethylene) were used to unambiguously assign the structure of the volatile compounds formed during the hydrogenative metathesis reaction.

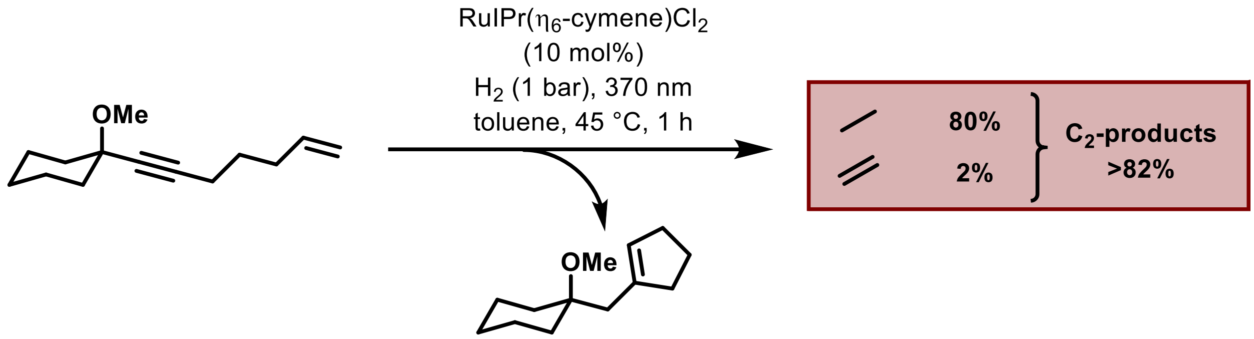

.

The presence of alkanes in the headspace is explained by reduction of the alkenes primarily formed by the same catalyst system. Terminal alkenes tend to be reduced (much) faster than more hindered internal alkenes, which likely explains why ethane is by far the major component of the C_2 fraction.

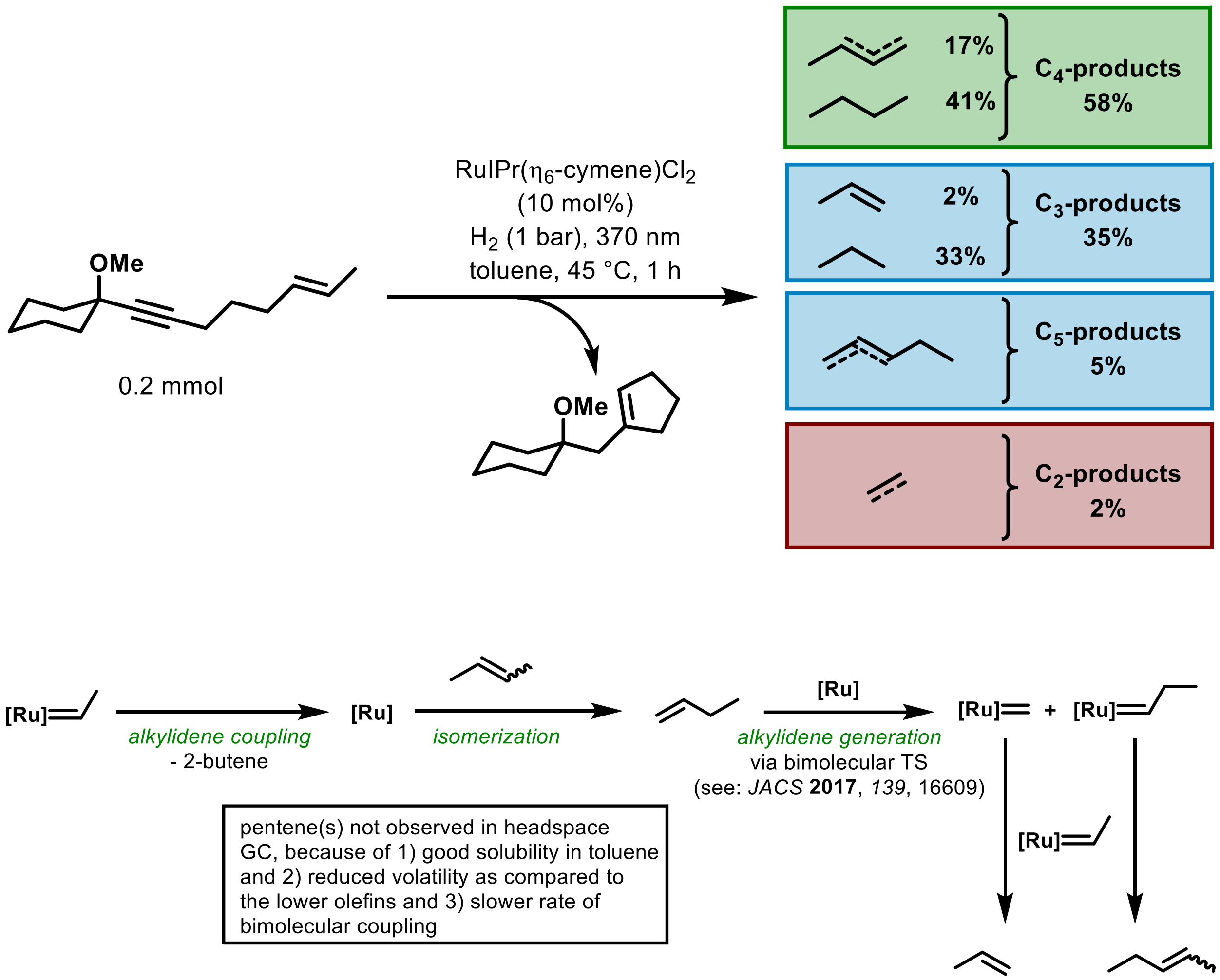

Figure S-5. Possible explanation for the formation of products with odd carbon numbers in the headspace

Hydrogenative metathesis of enyne **1b** yields 95% (NMR) of cyclopentene **2**. Analysis of the headspace by GC shows that the major species are C_4-products, namely butenes and butane. Interestingly, significant amounts of C_3- and C_5-products are detected as well: their formation is attributed to an isomerization/carbene formation/cross dimerization sequence (see Figure S-5). It has previously been shown that the 12e-fragment [(IPr)RuCl$_2$] generated under the reaction conditions is an effective catalyst for alkene isomerization.[4] Moreover, DFT calculations suggested that [(IPr)RuCl$_2$] is able to activate a terminal alkene to generate, via a bimolecular transition state, two new ruthenium carbenes.[4] Cross-dimerization of these "tertiary" ruthenium carbenes with the "secondary" ruthenium carbene explains the observation of C_3- and C_5-products.

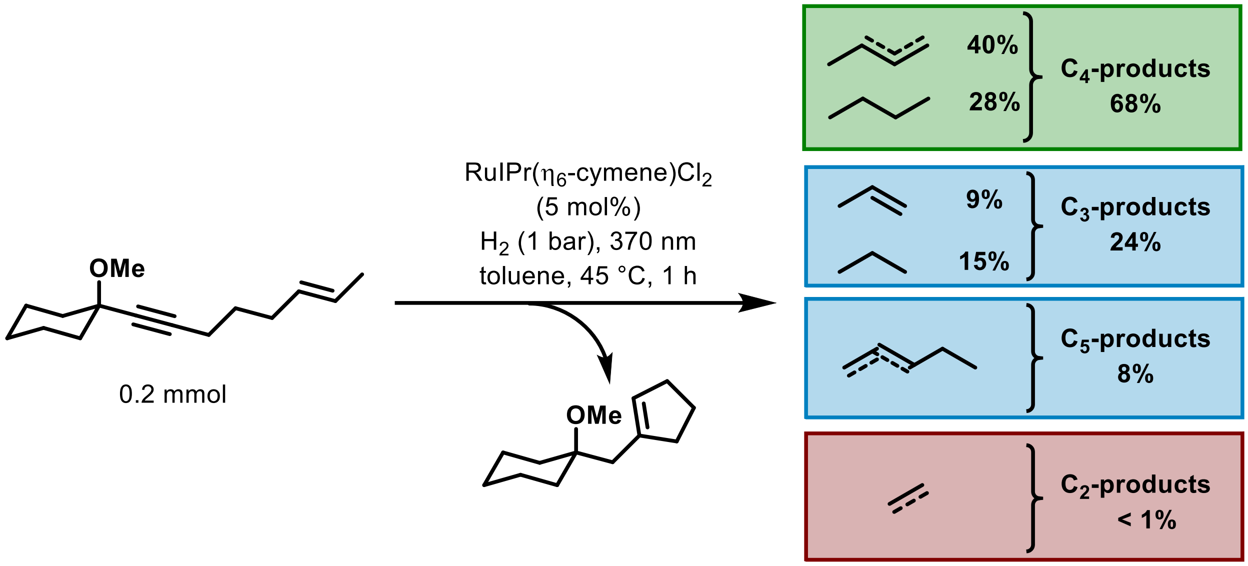

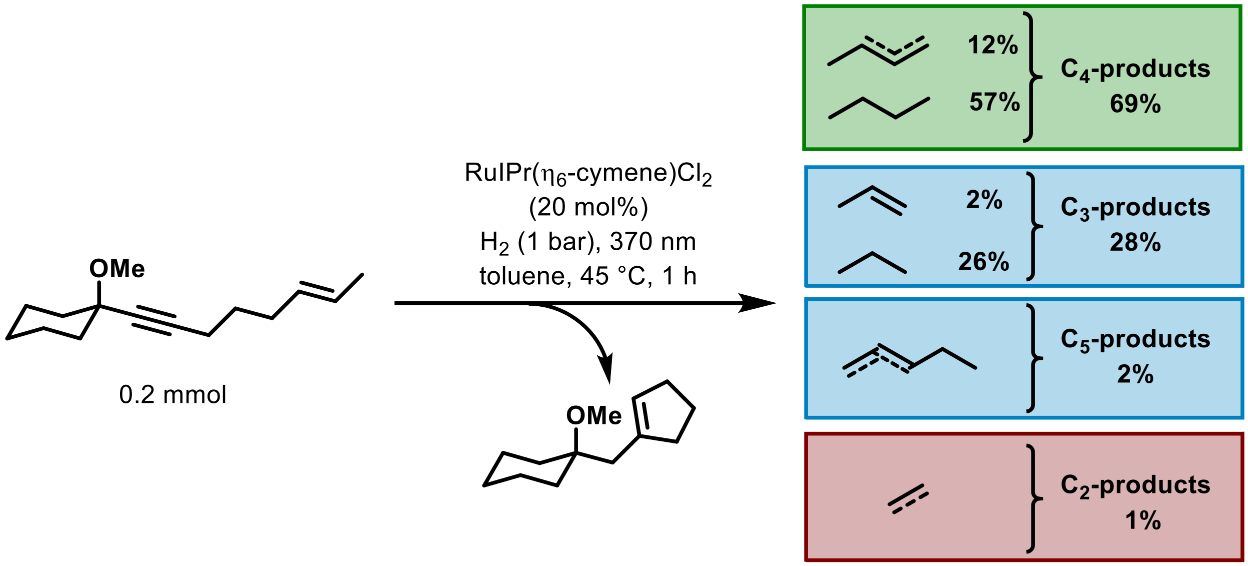

The model reaction was also run with 5 mol% and 20 mol% catalyst loading. Headspace GC analysis showed that the proportion of C_4 and C_3 compounds is essentially independent of the loading. However, the ratio between unsaturated and saturated compounds does change in that the proportion of alkanes is significantly increased at higher loading; this finding is again in line with a secondary hydrogenation process.

It is of note that Fogg, Jensen and coworkers have recently studied a pyridine-stabilized ruthenium ethylidene complex, which decomposes via bimolecular coupling to give a mixture of butene,

pentene and propene in a 2:1:1 ratio.[5] Whilst this observation is qualitatively similar to our observations, we note that pentenes and pentane are underrepresented (relative to propane/propene) in our study. The difference might be explained by the fact that different analytical tools were used (GC/MS versus NMR); since pentenes/pentane are less volatile than propene/propane, they might be underrepresented in the gas phase. Efforts to quantify the pentenes by NMR spectroscopy in the liquid phase were unsuccessful due to severe signal overlap with product **2**. Moreover, one might conceive that dimerization of a propylidene complex with an ethylidene complex is (somewhat) slower than the dimerization of a propylidene complex with a methylidene complex.

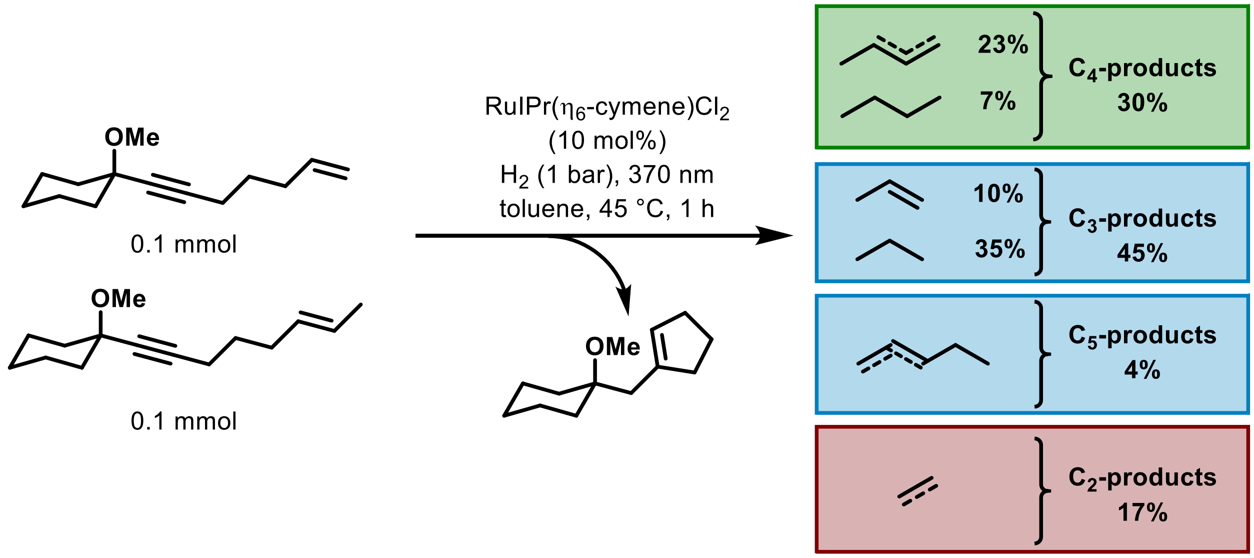

Hydrogenative metathesis with a 1:1 mixture of enynes **1a**[6] and **1b** confirms the bimolecular decomposition of the secondary carbene complex. The *increased* amount of C_3-products and concomitant *decrease* of C_4- and C_2-products strongly supports the notion that bimolecular alkylidene coupling is operative. The fact that the C_2:C_3:C_4 ratio is not 1:2:1, as statistically expected from such a crossover experiment, is ascribed to different rates of alkylidene coupling and to the necessary isomerization events discussed above.

Headspace analysis (GC) @ 10 mol% catalyst loading

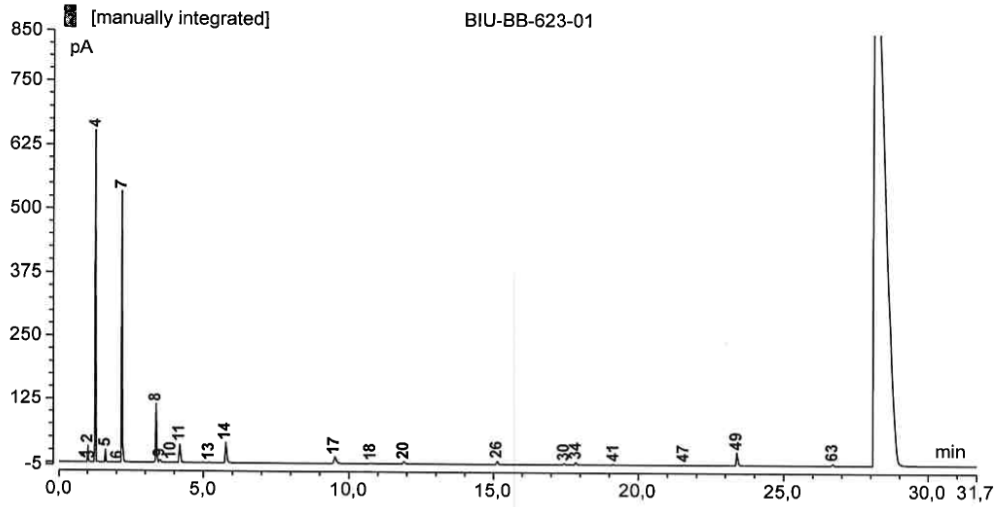

Sample:	**BIU-BB-623-01**	Instrument:	**GC_212**
Sequenz:	**7200 BIU-BB VD**	Measured:	**25.02.20 10:06**
Sequenz date:	**25.02.20**	Processing M.:	**Übersicht BIU-BB**
		Report-File:	**623-01**

Auswertung ohne Lösungsmittel (Toluol-d8)
Zuordnung mit Vergleichssubstanzen (Gasmischung Phillips No.40 und Methan, Ethan, Ethen)

No.	Ret.Time min	area-% %	Peak Name
1	0,94	0,09	Methan
2	1,01	1,31	Ethan
4	1,26	29,19	Propan
5	1,62	1,22	Propen
7	2,18	35,52	n-Butan
8	3,37	10,50	trans-2-Buten
9	3,51	0,51	1-Buten
10	3,88	0,02	iso-Buten
11	4,19	3,82	cis-2-Buten
12	4,98	0,07	
13	5,21	0,01	iso-Pentan
14	5,77	6,04	
16	8,57	0,02	
17	9,51	3,28	trans-2-Penten
18	10,76	0,20	1-Penten
19	11,26	0,01	
20	11,91	0,81	cis-2-Penten
25	14,09	0,01	
26	15,13	0,96	
28	16,45	0,01	
30	17,45	0,24	
31	17,62	0,07	
32	17,73	0,01	
33	17,76	0,01	
34	17,86	0,64	
36	18,17	0,04	
38	18,57	0,01	

Page 2-2

No.	Ret.Time min	area-% %	Peak Name
39	18,67	0,01	
40	18,75	0,04	
41	19,17	0,18	
44	20,00	0,01	
47	21,57	0,14	
48	23,13	0,09	
49	23,39	4,20	
50	24,14	0,02	
51	24,55	0,03	
52	24,67	0,01	
53	24,80	0,01	
54	24,91	0,01	
57	25,32	0,01	
58	25,55	0,03	
59	25,68	0,02	
60	25,89	0,03	
61	26,01	0,01	
63	26,69	0,44	
64	26,99	0,01	
66	27,98	0,02	

19 peaks out of 66 (total area percentage= 0,06 %) are below threshold.

Instrument parameters:

Column:	29,5 m	HP-Plot/Al2O3 0,25/5,0df G/425
Temperature:	220/ 80, 10 min iso 6/min 180, 5 min iso/ 350	
Gas:	0,80 bar	H2
Sample size:	450,0 µL	

V.Diehl

Page 1-2

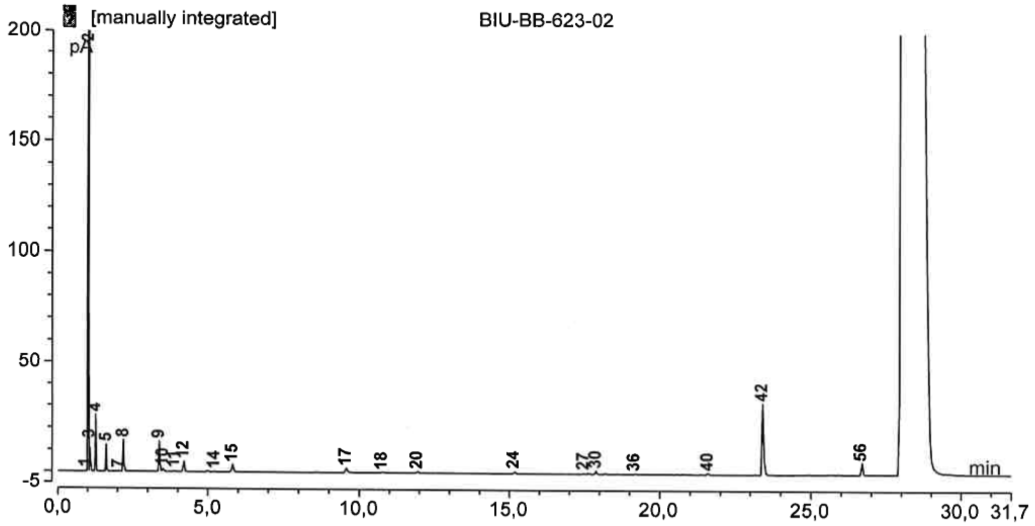

Sample: **BIU-BB-623-02**
Sequenz: **7200 BIU-BB VD**
Sequenz date: **25.02.20**

Instrument: **GC_212**
Measured: **25.02.20 14:35**
Processing M.: **Übersicht BIU-BB**
Report-File: **623-02**

Auswertung ohne Lösungsmittel (Toluol-d8)
Zuordnung mit Vergleichssubstanzen (Gasmischung Phillips No.40 und Methan, Ethan, Ethen)

No.	Ret.Time min	area-% %	Peak Name
1	0,94	0,08	Methan
2	1,01	63,30	Ethan
3	1,07	1,77	Ethen
4	1,26	3,27	Propan
5	1,62	1,71	Propen
7	2,04	0,02	iso-Butan
8	2,20	2,32	n-Butan
9	3,38	3,08	trans-2-Buten
10	3,51	0,26	1-Buten
11	3,89	0,08	iso-Buten
12	4,20	1,27	cis-2-Buten
13	4,99	0,10	
14	5,22	0,01	iso-Pentan
15	5,82	1,26	
16	8,60	0,05	
17	9,57	1,17	trans-2-Penten
18	10,77	0,16	1-Penten
19	11,28	0,02	
20	11,94	0,32	cis-2-Penten
21	13,09	0,01	
22	13,45	0,01	
23	14,13	0,02	
24	15,16	0,50	
25	15,96	0,01	
26	16,48	0,01	
27	17,48	0,17	
28	17,65	0,23	

Page 2-2

No.	Ret.Time min	area-% %	Peak Name
29	17,79	0,03	
30	17,88	0,45	
31	18,19	0,13	
32	18,47	0,02	
33	18,60	0,04	
34	18,69	0,02	
35	18,77	0,03	
36	19,19	0,13	
37	19,75	0,02	
38	19,83	0,03	
39	20,03	0,05	
40	21,59	0,17	
41	23,15	0,11	
42	23,40	15,14	
43	24,15	0,02	
44	24,57	0,04	
45	24,69	0,02	
46	24,84	0,04	
47	24,93	0,04	
48	25,12	0,02	
49	25,17	0,01	
50	25,35	0,05	
51	25,58	0,14	
52	25,70	0,05	
54	25,91	0,09	
55	26,02	0,04	
56	26,70	1,81	
57	27,00	0,02	
58	27,62	0,04	

2 peaks out of 58 (total area percentage= 0,00 %) are below threshold.

Instrument parameters:

Column:	29,5 m	HP-Plot/Al2O3 0,25/5,0df G/425
Temperature:	220/ 80, 10 min iso	6/min 180, 5 min iso/ 350
Gas:	0,80 bar	H2
Sample size:	450,0 µL	

V. Diehl

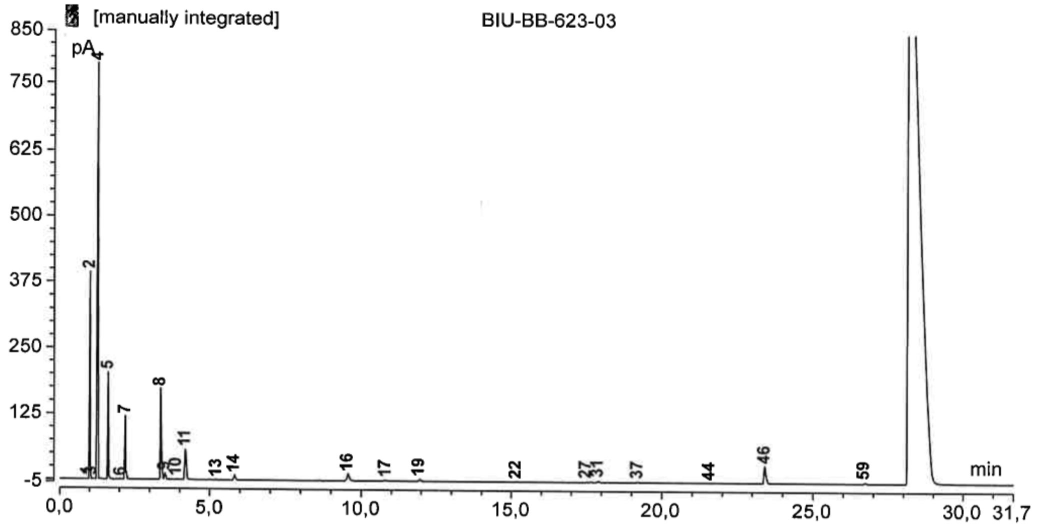

Sample:	**BIU-BB-623-03**	Instrument:	**GC_212**
Sequenz:	**7200 BIU-BB VD**	Measured:	**25.02.20 16:26**
Sequenz date:	**25.02.20**	Processing M.:	**Übersicht BIU-BB**
		Report-File:	**623-03**

Auswertung ohne Lösungsmittel (Toluol-d8)
Zuordnung mit Vergleichssubstanzen (Gasmischung Phillips No.40 und Methan, Ethan, Ethen)

**No.**	**Ret.Time min**	**area-% %**	**Peak Name**
1	0,94	0,06	Methan
2	1,00	17,01	Ethan
3	1,06	0,05	Ethen
4	1,25	34,99	Propan
5	1,62	9,83	Propen
6	2,04	0,01	iso-Butan
7	2,19	6,68	n-Butan
8	3,36	14,29	trans-2-Buten
9	3,51	0,98	1-Buten
10	3,89	0,06	iso-Buten
11	4,19	5,43	cis-2-Buten
12	4,99	0,04	
13	5,23	0,01	iso-Pentan
14	5,82	1,03	.
15	8,61	0,08	
16	9,56	2,55	trans-2-Penten
17	10,81	0,24	1-Penten
18	11,31	0,02	
19	11,95	0,69	cis-2-Penten
21	14,13	0,01	
22	15,17	0,10	.
23	15,97	0,01	
25	16,48	0,01	
27	17,49	0,13	.
28	17,65	0,11	
29	17,74	0,01	
30	17,79	0,01	

Page 2-2

No.	Ret.Time min	area-% %	Peak Name
31	17,89	0,37	
32	18,20	0,05	
34	18,60	0,02	
35	18,70	0,02	
36	18,78	0,02	
37	19,20	0,11	
40	20,03	0,01	
44	21,60	0,02	
45	23,16	0,04	
46	23,41	4,40	
47	24,17	0,01	
48	24,58	0,01	
50	24,94	0,01	
51	25,12	0,01	
52	25,36	0,01	
54	25,58	0,02	
55	25,71	0,01	
56	25,92	0,02	
57	26,04	0,01	
59	26,72	0,35	
61	27,65	0,01	
62	28,01	0,01	

13 peaks out of 62 (total area percentage= 0,04 %) are below threshold.

Instrument parameters:

Column:	29,5 m	HP-Plot/Al2O3 0,25/5,0df G/425
Temperature:	220/ 80, 10 min iso 6/min 180, 5 min iso/ 350	
Gas:	0,80 bar	H2
Sample size:	450,0 µL	

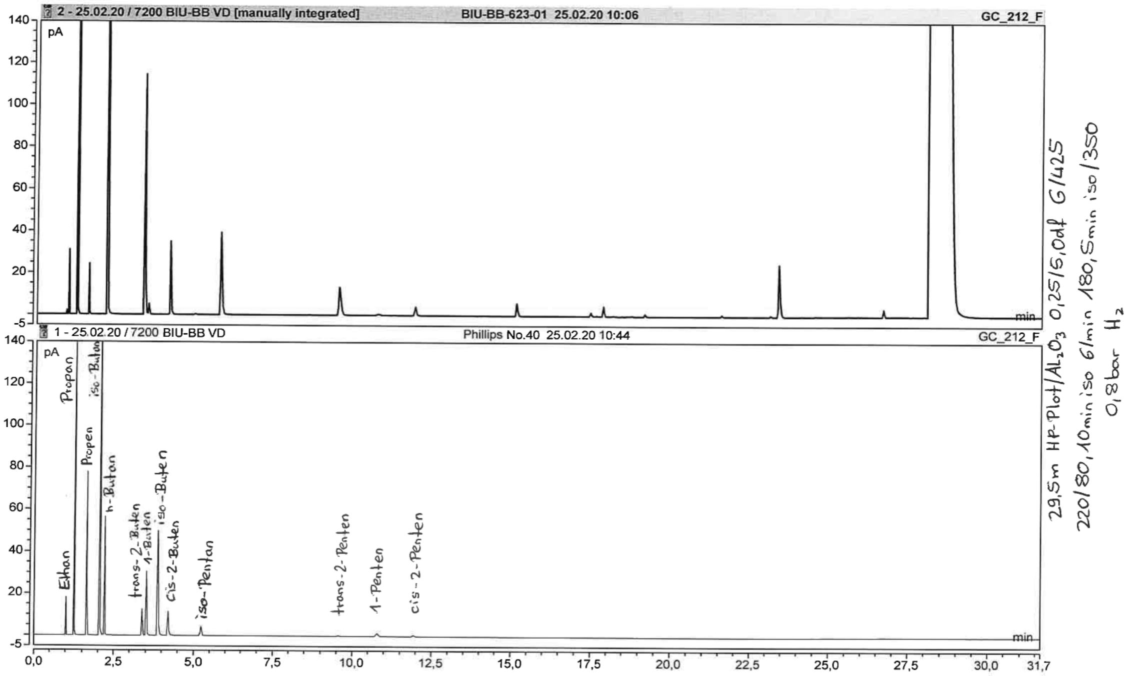

2.5 VT NMR Studies of [(NHC)(*p*-cymene)$RuCl_2$] Complexes

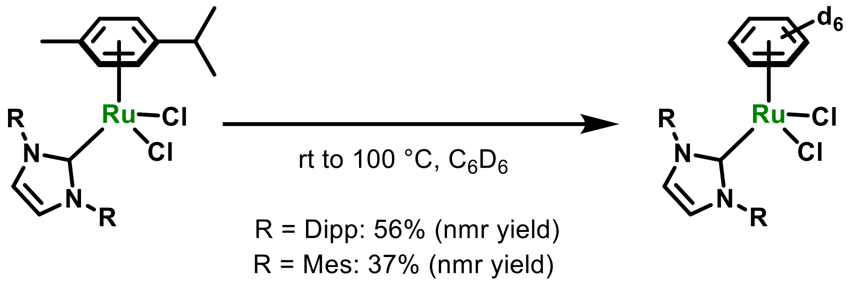

A flame-dried pressure NMR tube was charged with a solution of the corresponding complex [(NHC)(*p*-cymene)$RuCl_2$] (15 µmol) in [D_6]-benzene (500 µL). The NMR tube was inserted into the NMR probe head and spectra were acquired in a temperature range from 25 °C to 100 °C (10 °C increments).

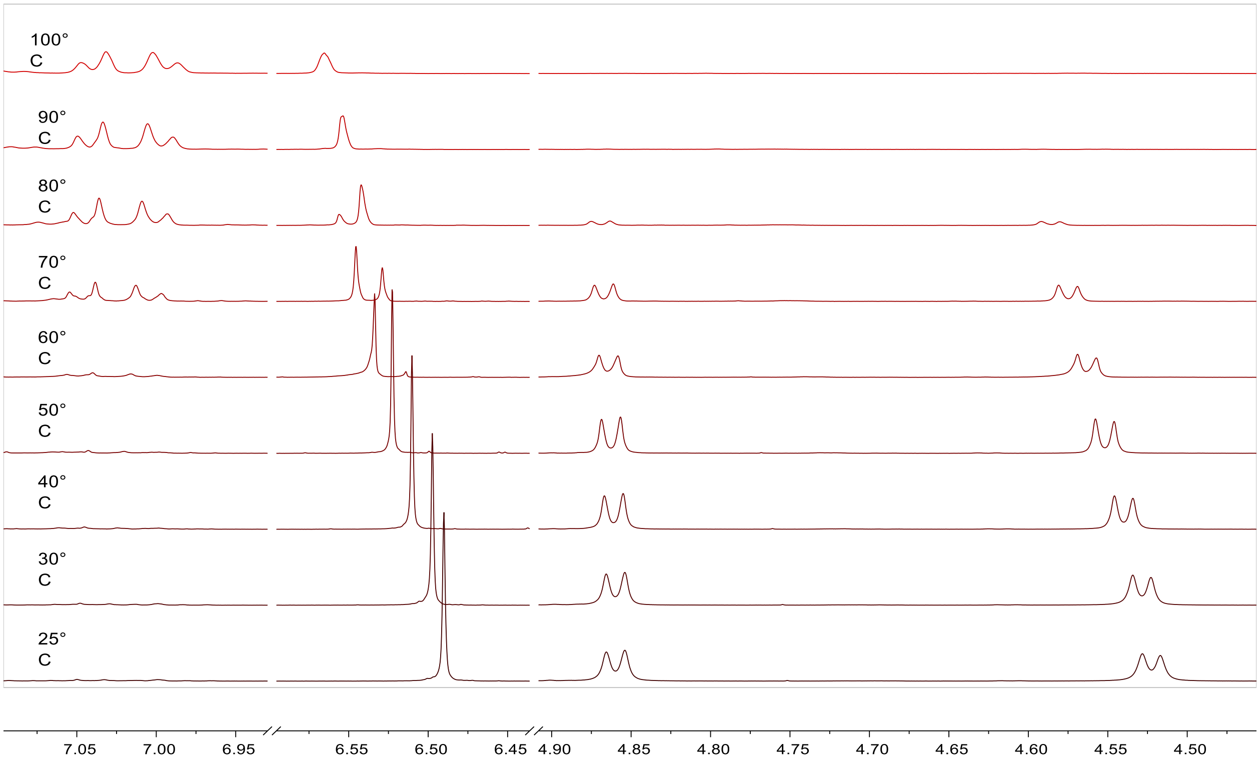

Figure S-6. Exchange of the *p*-cymene ligand in [(IPr)(η^6-cymene)$RuCl_2$] by C_6D_6 under themal conditions in the dark

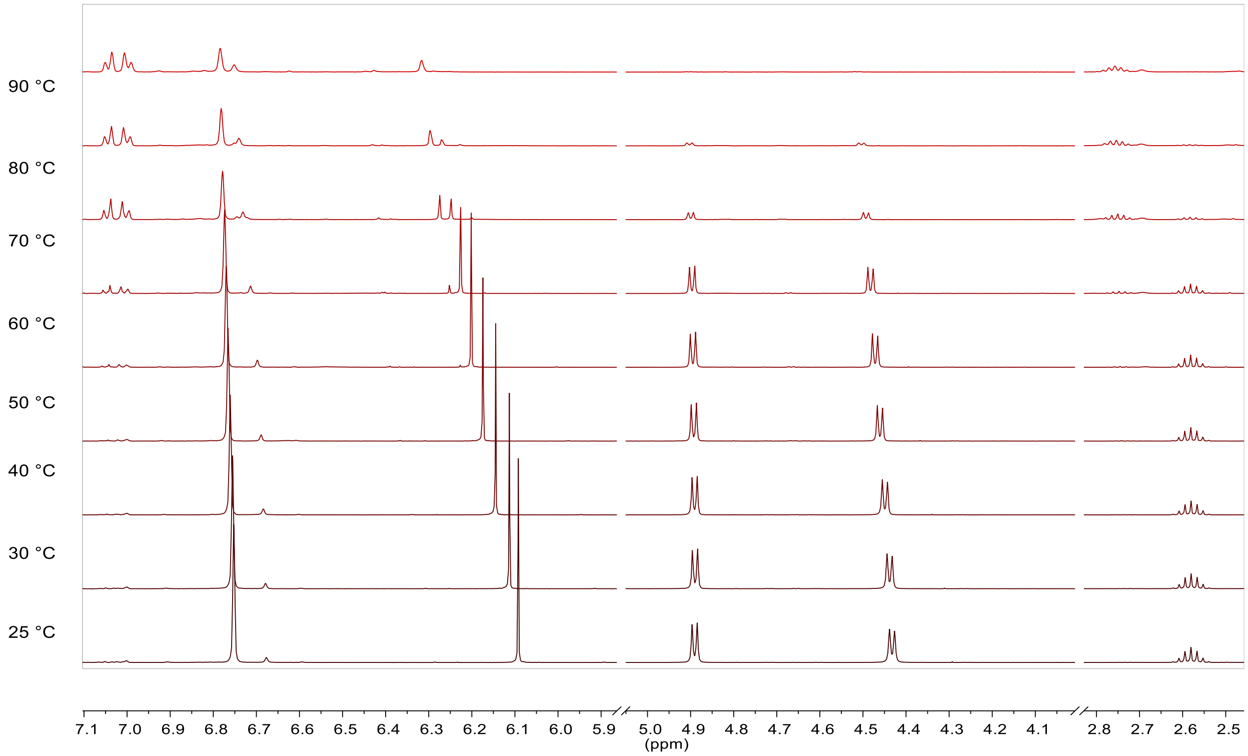

Figure S-7. Exchange of the *p*-cymene ligand in [(IMes)(η^6-cymene)$RuCl_2$] by C_6D_6 under themal conditions in the dark

We infer that ligand exchange with [(IPr)(*p*-cymene)$RuCl_2$] is more efficient compared to [(IMes)(*p*-cymene)$RuCl_2$] as evidenced by the higher NMR yield. In both cases, the ligand displacement starts at 60-70 °C.

2.6 The Impact of the UV Irradiation on the Reaction Outcome

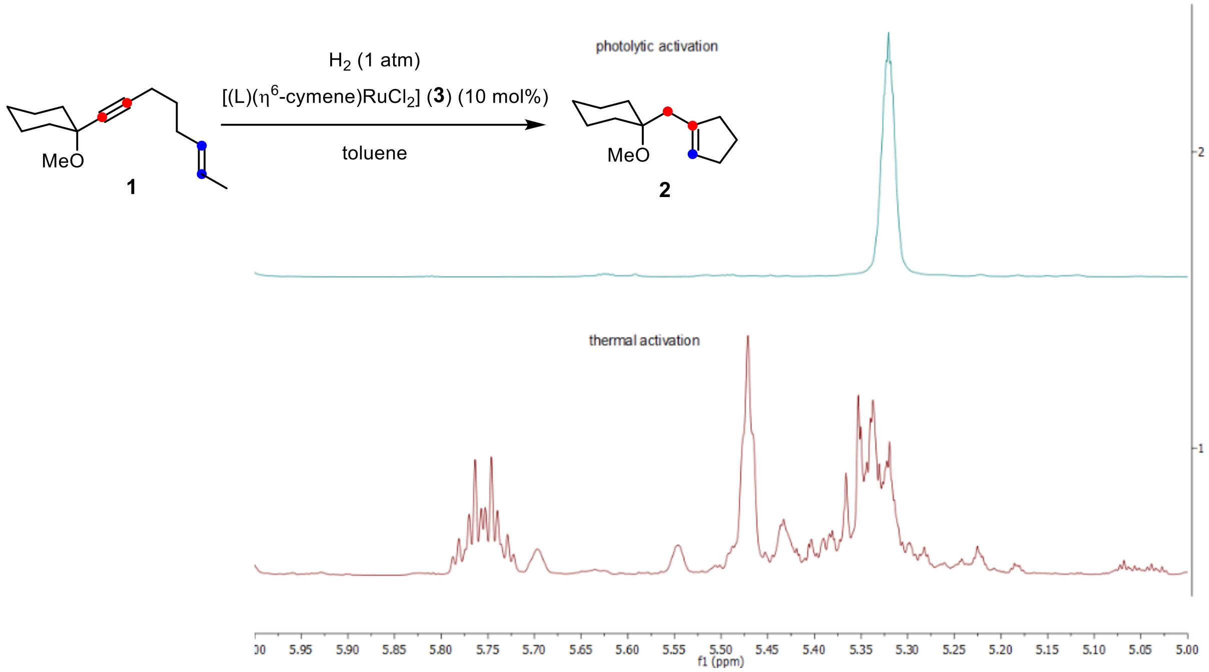

Figure S-8. Comparison of the ^{1}H NMR spectra of the crude reaction mixture formed with photochemical (top) or themal (100°C, bottom) activation: region of the olefinic signals

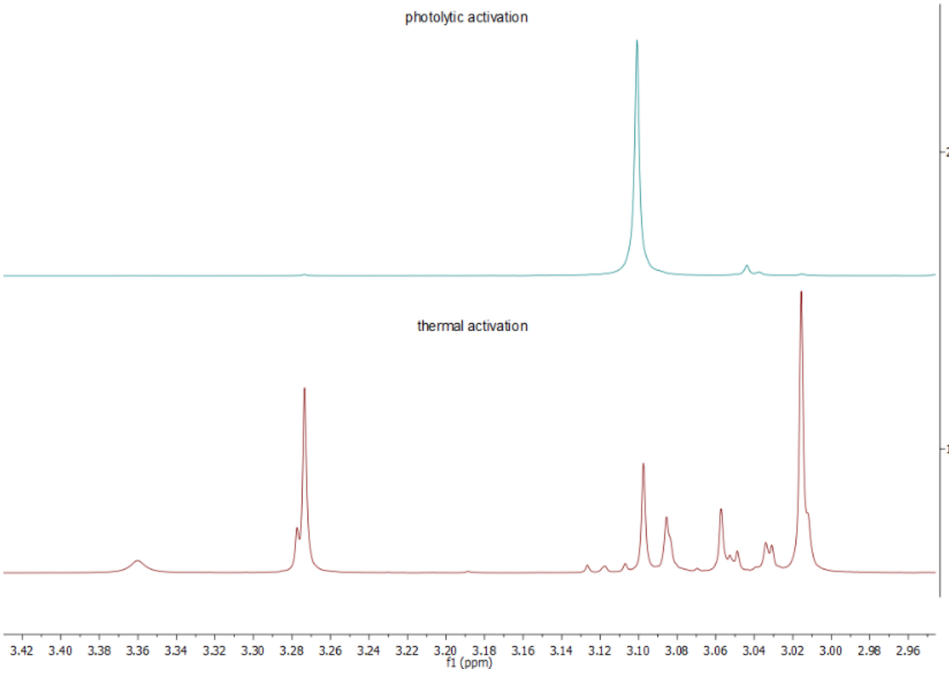

Figure S-9. Comparison of the ^{1}H NMR spectra of the crude reaction mixture formed with photochemical (top) or themal (100°C, bottom) activation: region of the –OMe signal

2.7 Control Experiment: Orthogonal Entry via a Grubbs Ethylidene Complex

[1,3-Bis(2,6-diisopropyl-phenyl)-2-imidazolidinylidene]dichloro(benzylidene)bis(pyridine)-ruthenium

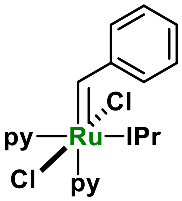

(1,3-Bis(2,6-diisopropylphenyl)-2-imidazolidinylidene)dichloro(phenylmethylene)-(tricyclohexylphosphine)ruthenium (1.00 g, 1.93 mmol) was weighed into a Schlenk tube and neat pyridine (3.90 mL, 48.3 mmol) was added at room temperature, causing an immediate color change from purple to dark green. After 15 min, pentane (20 mL) were carefully layered onto the solution and the Schlenk tube was sealed and stored in a fridge for 18 h. The precipitated light green crystals were filtered off with a filter canula, washed with pentane (3 x 15 mL) and dried under high vacuum to provide the compound as a light green powder (830 mg, 95%). ^{1}H NMR (400 MHz, C_6D_6) δ 19.82 (s, 1H), 8.70 (d, *J* = 4.9 Hz, 2H), 8.36 (d, *J* = 5.4 Hz, 2H), 7.99 – 7.86 (m, 2H), 7.33 – 7.22 (m, 2H), 7.21 – 7.10 (m, 5H), 6.93 – 6.82 (m, 3H), 6.73 (s, 2H), 6.55 (t, *J* = 6.5 Hz, 2H), 6.38 (t, *J* = 7.7 Hz, 1H), 6.05 (t, *J* = 6.7 Hz, 2H), 3.66 (s, 4H), 1.46 (s, 12H), 1.10 (d, *J* = 6.8 Hz, 12H). ^{13}C NMR (101 MHz, C_6D_6) δ 314.0, 186.9, 153.2, 152.7, 150.6, 147.9, 137.7, 135.5, 135.0, 130.4, 130.2, 129.6, 126.2, 124.3, 123.2, 122.9, 28.8, 26.5, 23.3.

[1,3-Bis(2,6-diisopropyl-phenyl)-2-imidazolidinylidene]dichloro(ethylidene)bis(pyridine)-ruthenium (24).[7]

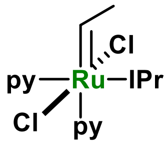

[1,3-Bis(2,6-diisopropyl-phenyl)-2-imidazolidinylidene]dichloro(benzylidene)bis(3-bromopyridine)ruthenium (50 mg, 61.7 µmol) was dissolved in C_6D_6 (1.5 mL) and the resulting solution was cooled to 5 °C (just above the melting point of benzene). (2*Z*)-Butene was bubbled through the mixture for 1 min, causing a color change from dark green to dark yellow within 3 min. The solution was then cooled to 0 °C to freeze the benzene, which was then sublimed off under high vacuum. The residue was then redissolved in cold pentane and the pentane was again removed under high vacuum to azeotropically remove traces of residual methyl styrene that was formed during the reaction. Drying under high vacuum afforded the compound as a yellow-brown solid (42 mg, 93%). The complex decomposes readily at ambient temperature. ^{1}H NMR (400 MHz, C_6D_6) δ 19.70 (q, *J* = 6.0 Hz, 1H), 8.75 (d, *J* = 5.1 Hz, 4H), 7.28 – 7.06 (m, 12H), 6.72 (s, 2H), 3.89 – 3.49 (m, 4H), 1.89 (d, *J* = 6.0 Hz, 3H), 1.58 (d, *J* = 6.5 Hz, 12H), 1.08 (d, *J* = 7.0 Hz, 12H). *Due to the thermal instability of the sample, a ^{13}C NMR spectrum could not be recorded.*

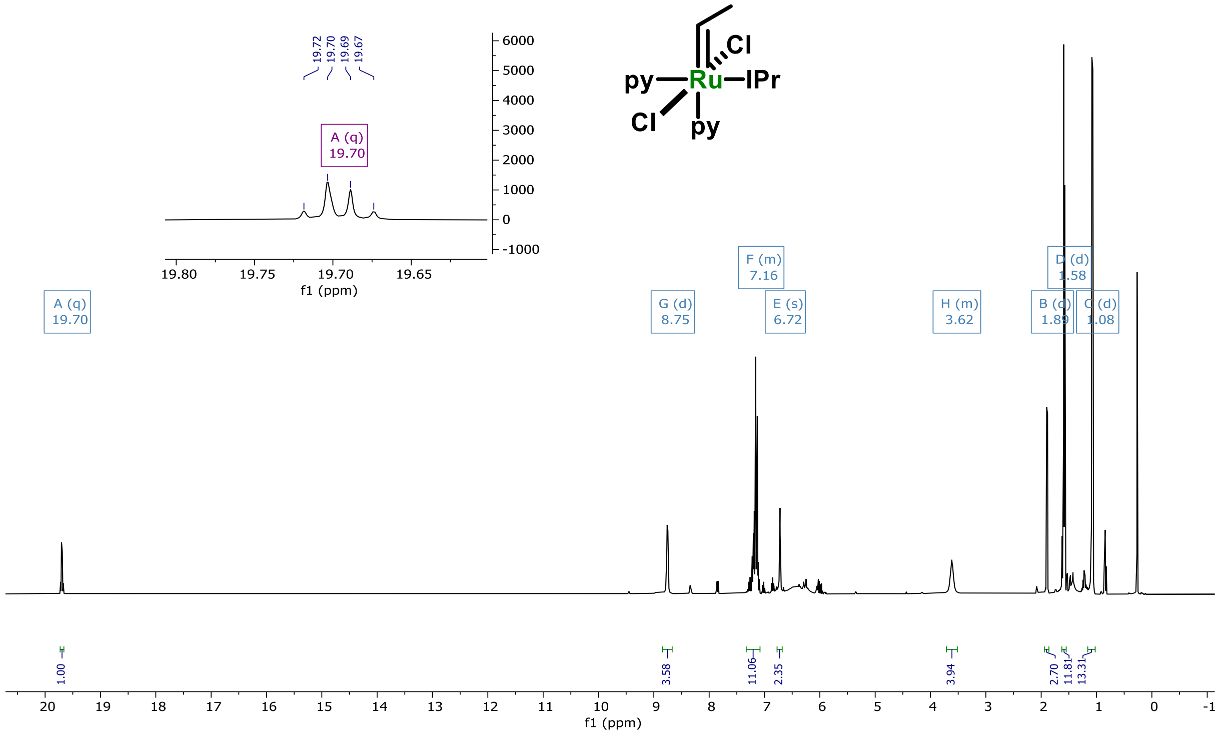

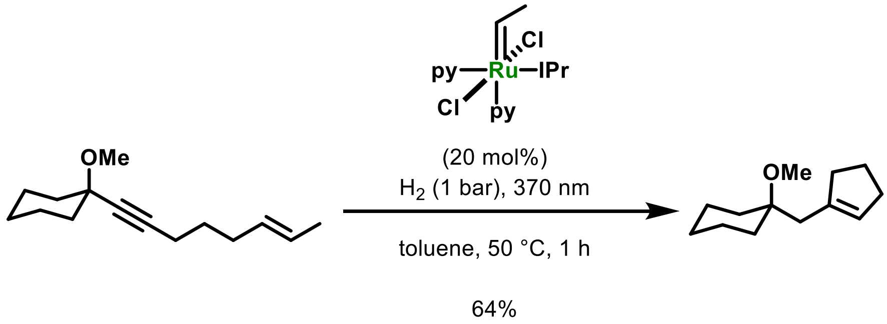

A flame-dried quartz Schlenk tube was charged with enyne **1a** (66.0 mg, 0.31 mmol) and toluene (1 mL). The Schlenk tube was closed with a septum and then transferred into the photolysis apparatus. A hydrogen-filled balloon was connected to a needle which was pierced through the septum and the Schlenk tube was flushed with hydrogen for 2 min through an outlet cannula (the cannula did not reach into the solution to make sure that only the head space of the tube was flushed). The ethylidene complex **24** (42.0 mg, 20 mol%) in toluene (2 mL) was added via syringe and the resulting mixture was stirred for 60 min under H_2 atmosphere. The mixture was diluted with pentane (5 mL) and then filtered through a short pad of silica. The filtrate was evaporated and the residue was analyzed by ^{1}H NMR spectroscopy, which showed the formation of product **2** in 64% NMR yield; the analytical data of this compound are compiled below.

3. Synthesis of Substrates and Precatalysts

3.1 Synthesis of the Substrates

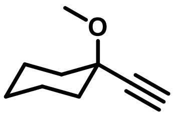

S1

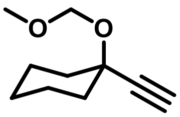

S2

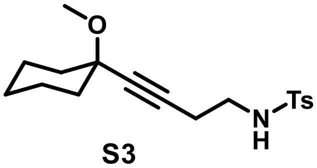

S3

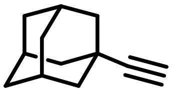

S4

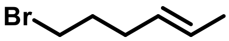

S5

The alkynes **S1**,[13] **S2**,[13] **S3**,[13] **S4**[8] and (*E*)-6-bromohex-2-ene (**S5**)[9] were prepared according to literature procedures.

(*E*)-7-Bromohept-2-ene (S6)

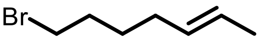

A solution of (*E*)-hept-5-en-1-ol (498 mg, 4.36 mmol)[10] and CBr_4 (1.60 g, 4.82 mmol) in CH_2Cl_2 (25 mL) was cooled to 0 °C before PPh_3 (1.26 g, 4.82 mmol) was added in portions over a period of 30 min under vigorous stirring. Once the addition was complete, stirring was continued for another 2.5 h at ambient temperature. The mixture was concentrated to half of the volume under reduced pressure and then slowly added to hexanes (250 mL) with vigorous stirring. After 5 min, the precipitate was filtered off and the filtrate was concentrated under reduced pressure. Purification of the residue by flash chromatography (silica, pentane) provided the title compound as colorless oil (328 mg, 42%). ^{1}H NMR (400 MHz, $CDCl_3$) δ 5.71 – 5.20 (m, 2H), 3.41 (t, *J* = 6.8 Hz, 2H), 2.05 – 1.96 (m, 2H), 1.90 – 1.81 (m, 2H), 1.64 (dq, *J* = 4.3, 1.2 Hz, 3H), 1.56 – 1.44 (m, 2H). ^{13}C NMR (101 MHz, $CDCl_3$) δ 130.7, 125.6, 34.0, 32.4, 31. 8, 28.2, 18.1. IR (film) $\tilde{\nu}$ 2961, 2933, 2855, 1452, 1438, 1250, 1226, 964, 645, 562 cm^{-1}. HRMS (EI) for $C_7H_{13}Br$ $[M]^+$: calcd. 176.01958; found: 176.01953.

1-Methoxy-1-(prop-1-yn-1-yl)cyclohexane (S7)

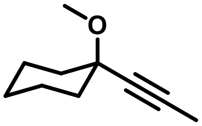

Sodium hydride (381 mg, 15.9 mmol) was added in portions over 5 min to a solution of 1-(1-propynyl)cyclohexanol (2.00 g, 14.5 mmol) in THF (72 mL) at 0 °C. The suspension was stirred for 30 min before dimethyl sulfate (1.53 mL, 16.2 mmol) was added dropwise and stirring was continued overnight at room temperature. Water (100 mL) was carefully added and the mixture was extracted with pentane (3 x 50 mL). The combined organic fractions were washed with brine, dried over Na_2SO_4 and concentrated under reduced

pressure (40 °C, 100 mbar). The residue was dissolved in pentane/diethyl ether (50:1) and the turbid solution filtered through a short silica plug. After evaporation of all volatile materials, the compound was purified by vacuum distillation (34 mbar, bath temperature: 135 °C, head temperature 100 °C) which yields the title compound as a colorless liquid (2.10 g, 95%). ^{1}H NMR (400 MHz, $CDCl_3$) δ 3.34 (s, 3H), 1.87 (s, 3H), 1.84 – 1.78 (m, 2H), 1.67 – 1.41 (m, 8H). ^{13}C NMR (101 MHz, $CDCl_3$) δ 81.6, 80.3, 74.0, 50.5, 37.0, 25.6, 22.9, 3.6. IR (film) $\tilde{\nu}$ 2933, 2856, 1447, 1294, 1185, 1172, 1145, 1091, 1086, 925 cm^{-1}. HRMS (ESI^{+}) for $C_{10}H_{16}ONa$ $[M+Na]^{+}$: calcd. 175.10935; found: 175.10935.

1-Bromo-2-(3-methoxy-3-methylbut-1-yn-1-yl)benzene (S8)

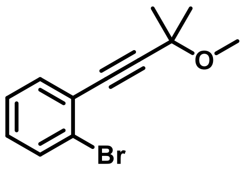

Sodium hydride (141 mg, 5.89 mmol) was added to a solution of 4-(2-bromophenyl)-2-methylbut-3-yn-2-ol (880 mg, 3.68 mmol)[11] in DMF (15 mL) at 0 °C and the resulting mixture was stirred for 30 min at this temperature. Methyl iodide (791 mg, 0.34 mL, 5.52 mmol) was added and stirring was continued for 15 h at ambient temperature. Water (20 mL) was introduced and the mixture was extracted with *tert*-butyl methyl ether (3 x 20 mL). The combined organic layers were washed with brine, dried over Na_2SO_4 and concentrated under reduced pressure. Purification of the residue was achieved by flash chromatography (silica, pentane/*tert*-butyl methyl ether 50:1) to yield the title compound (727 mg, 78%) as a colorless oil. ^{1}H NMR (400 MHz, $CDCl_3$) δ 7.61 – 7.55 (m, 1H), 7.47 – 7.43 (m, 1H), 7.28 – 7.22 (m, 1H), 7.16 (ddd, *J* = 8.0, 7.5, 1.7 Hz, 1H), 3.48 (s, 3H), 1.57 (s, 6H). ^{13}C NMR (101 MHz, $CDCl_3$) δ 133.4, 132.5, 129.5, 127.0, 125.8, 125.1, 96.0, 83.0, 71.2, 52.1, 28.4. IR (film) $\tilde{\nu}$ 2983, 1467, 1433, 1360, 1289, 1266, 1186, 1171, 1146, 1072, 870, 794, 750, 679, 520 cm^{-1}. HRMS (GC-EI) for $C_{12}H_{13}OBr$ $[M]^{+}$: calcd. 252.01453; found: 252.01444.

2-Ethynyl-2-methoxyadamantane (S9)

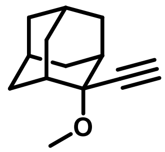

Prepared analogously from 2-ethynyladamantan-2-ol[12] as a colorless oil (169 mg, 72%). ^{1}H NMR (400 MHz, $CDCl_3$) δ 3.37 (s, 3H), 2.52 (s, 1H), 2.16 – 2.09 (m, 2H), 2.08 – 2.02 (m, 4H), 1.88 – 1.75 (m, 4H), 1.71 – 1.67 (m, 2H), 1.57 – 1.48 (m, 2H). ^{13}C NMR (101 MHz, $CDCl_3$) δ 85.4, 77.9, 74.4, 50.1, 37.7, 35.5, 35.1, 31.5, 27.2, 26.7. IR (film) $\tilde{\nu}$ 3304, 2900, 2848, 2104, 1450, 1228, 616, 511 cm^{-1}. HRMS (GC-EI) for $C_{13}H_{18}O$ $[M]^{+}$: calcd. 190.13516; found: 190.13522.

Mestranol methyl ether (S10)

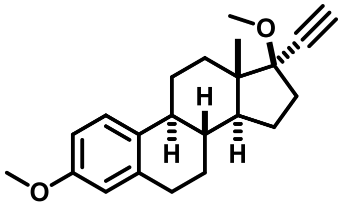

Prepared analogously from commercially available mestranol as a colorless oil (266 mg, 55%). ^{1}H NMR (400 MHz, $CDCl_3$) δ 7.21 (dd, *J* = 8.7, 1.1 Hz, 1H), 6.71 (dd, *J* = 8.6, 2.8 Hz, 1H), 6.63 (d, *J* = 2.5 Hz, 1H), 3.78 (s, 3H), 3.42 (s, 3H), 2.95 – 2.77 (m, 2H), 2.61 (s, 1H), 2.44 – 2.17 (m, 3H), 2.01 (tt, *J* = 13.6, 4.9 Hz, 2H), 1.90 – 1.66 (m, 3H), 1.55 (s, 2H), 1.51 – 1.27 (m, 3H), 0.88 (d, *J* = 0.7 Hz, 3H). ^{13}C NMR (101 MHz, $CDCl_3$) δ 157.5, 138.0, 132.7, 126.5, 113.9, 111.6, 85.8, 84.8, 75.8, 55.3, 53.4, 49.6, 47.6, 43.6, 39.3, 36.7, 34.2, 30.0, 27.4, 26.7, 22.8, 12.8. IR (film) $\tilde{\nu}$ 2933, 1612, 1504, 1465, 1447, 1290, 1252, 1240, 1134, 1109, 1021, 904, 833 cm^{-1}. HRMS (GC-EI) for $C_{22}H_{28}O_2$ $[M]^+$: calcd. 324.20838; found: 324.20849.

(*E*)-1-Methoxy-1-(oct-6-en-1-yn-1-yl)cyclohexane (1b)

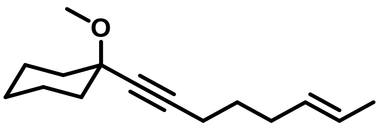

Prepared according to a literature procedure.[13] The analytical data matched those previously reported.

(*E*)-1-(But-2-en-1-yl)-2-(3-methoxy-3-methylbut-1-yn-1-yl)benzene (S11)

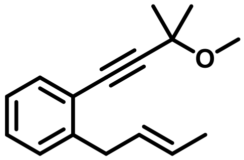

n-BuLi (1.55 M in hexanes, 1.91 mL, 2.96 mmol) was added to a solution of **S8** (500 mg, 1.97 mmol) in THF (10 mL) at –78 °C. The mixture stirred for 20 min before TMEDA (0.29 mL, 1.97 mmol) was added. After stirring at –78 °C for another 20 min, crotyl bromide (mixture of (*E/Z*)-isomers, 0.30 mL, 2.96 mmol) was introduced and stirring was continued for 15 h. Water (20 mL) was added and the mixture was extracted with *tert*-butyl methyl ether (3 x 20 mL). The combined organic fractions were dried over Na_2SO_4 and concentrated under reduced pressure. The purification of the residue was achieved by flash chromatography (silica, pentane/*tert*-butyl methyl ether 100:1) to yield the title compound as a colorless oil (mixture of isomers, 420 mg, 93%). ^{1}H NMR (400 MHz, $CDCl_3$) δ 7.41 (m, 1H), 7.28 – 7.22 (m, 1H), 7.20 (m, 1H), 7.14 (m, 1H), 5.68 – 5.44 (m, 2H), 3.58 – 3.47 (m, 2H), 3.45 (m, 3H), 1.75 – 1.66 (m, 3H), 1.56 (m, 6H). ^{13}C NMR (101 MHz, $CDCl_3$) δ 143.0, 132.4, 129.1, 128.7, 128.5, 126.7, 125.9, 122.3, 83.1, 71.2, 51.9, 37.7, 28.6, 18.0. IR (film) $\tilde{\nu}$ 2983, 2934, 1483, 1447, 1377, 1359, 1279, 1171, 1146, 1074, 966, 755 cm^{-1}. HRMS (GC-CI) for $C_{16}H_{21}O$ $[M+H]^+$: calcd. 229.15847; found: 229.15869.

(*E*)-Oct-6-en-1-yn-1-ylbenzene (S12a)

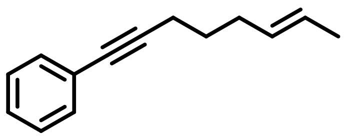

Prepared analogously from commercially available phenyl acetylene and bromide **S5** as a colorless oil (266 mg, 74%). ^{1}H NMR (400 MHz, $CDCl_3$) δ 7.40 (dt, *J* = 6.9, 1.8 Hz, 2H), 7.27 (ddd, *J* = 6.3, 2.6, 1.0 Hz, 3H), 5.57 – 5.37 (m, 2H), 2.40 (td, *J* = 7.1, 1.1 Hz, 2H), 2.15 (dtd, *J* = 7.6, 5.8, 1.4 Hz, 2H), 1.72 – 1.62 (m, 5H). ^{13}C NMR (101 MHz, $CDCl_3$) δ 131.6, 130.4, 128.3, 127.6, 125.8, 124.2, 90.3, 80.8, 31.8, 28.7, 18.9, 18.1. IR (film) $\tilde{\nu}$ 2934, 1598, 1490, 1441, 1330, 1070, 1027, 965, 912, 754, 690, 525 cm^{-1}. HRMS (GC-EI) for $C_{14}H_{16}$ $[M]^+$: calcd. 184.12461; found: 184.12465.

(*E*)-1-methyl-4-(oct-6-en-1-yn-1-yl)benzene (S12b)

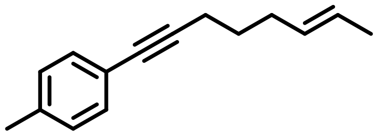

Prepared analogously from commercially available 1-ethynyl-4-methylbenzene and bromide **S5** as a slightly yellow oil (318 mg, 80%). ^{1}H NMR (400 MHz, $CDCl_3$) δ 7.31 – 7.27 (m, 2H), 7.11 – 7.06 (m, 2H), 5.55 – 5.38 (m, 2H), 2.39 (t, *J* = 7.2 Hz, 2H), 2.33 (s, 3H), 2.18 – 2.10 (m, 2H), 1.70 – 1.62 (m, 5H). ^{13}C NMR (101 MHz, $CDCl_3$) δ 137.6, 131.5, 130.5, 129.1, 121.1, 89.5, 80.9, 31.8, 28.8, 21.5, 19.0, 18.1. IR (film) 2932, 2857, 1509, 1452, 1437, 965, 815, 526, 412 cm^{-1}.

(*E*)-1-methoxy-4-(oct-6-en-1-yn-1-yl)benzene (S12c)

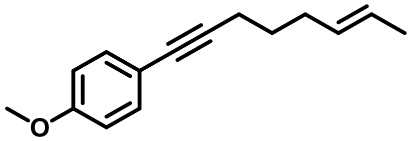

Prepared analogously from commercially available 1-ethynyl-4-methoxybenzene and bromide **S5** as a slightly yellow oil (468 mg, 96%). ^{1}H NMR (400 MHz, $CDCl_3$) δ 7.36 – 7.30 (m, 2H), 6.84 – 6.78 (m, 2H), 5.55 – 5.38 (m, 2H), 3.80 (s, 3H), 2.38 (t, *J* = 7.2 Hz, 2H), 2.19 – 2.09 (m, 2H), 1.72 – 1.61 (m, 5H). ^{13}C NMR (101 MHz, $CDCl_3$) δ 159.1, 133.0, 130.6, 125.8, 116.4, 113.9, 88.7, 80.6, 55.4, 31.8, 28.8, 18.9, 18.1. IR (film) 2933, 2836, 1606, 1508, 1288, 1243, 1171, 1034, 965, 829, 534 cm^{-1}. HRMS (ESI$^+$) for $C_{15}H_{18}O$ $[M+H]^+$: calcd. 215.14304, found: 215.14288.

(*Z*)-1-Chloro-4-(oct-6-en-1-yn-1-yl)benzene (S12d)

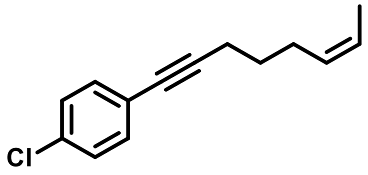

Prepared analogously from commercially available 1-ethynyl-4-methylbenzene and (*Z*)-6-bromohex-2-ene[14] as a slightly yellow oil (297 mg, 63%). ^{1}H NMR (400 MHz, $CDCl_3$) δ 7.35 – 7.28 (m, 2H), 7.27 – 7.22 (m, 2H), 5.51 (ddddd, *J* = 9.4, 7.9, 6.5, 4.7, 3.3 Hz, 1H), 5.44 – 5.35 (m, 1H), 2.41 (t, *J* = 7.1 Hz, 2H), 2.21 (qt, *J* = 7.2, 1.2 Hz, 2H), 1.71 – 1.62 (m, 5H). ^{13}C NMR (101 MHz, $CDCl_3$) δ 133.6, 132.9, 129.6, 128.6, 125.1, 122.7, 91.4, 79.8, 28.6, 26.1,

19.0, 13.0. IR (film) 3013, 2933, 2861, 1488, 1454, 1397, 1345, 1090, 1014, 825, 699, 524 cm^{-1}. HRMS (GC-EI) for $C_{14}H_{15}Cl$ $[M]^+$: calcd. 218.08568, found: 218.08575.

(*E*)-Triisopropyl(oct-6-en-1-yn-1-yl)silane (S13)

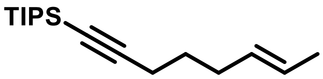

Prepared analogously from commercially available TIPS-acetylene and bromide **S5** as a colorless oil (351 mg, 81%). ^{1}H NMR (400 MHz, $CDCl_3$) δ 5.51 – 5.35 (m, 2H), 2.24 (t, *J* = 7.0 Hz, 2H), 2.15 – 2.07 (m, 2H), 1.66 – 1.62 (m, 3H), 1.61 – 1.53 (m, 2H), 1.13 – 1.01 (m, 21H). ^{13}C NMR (101 MHz, $CDCl_3$) δ 130.5, 125.8, 94.8, 80.3, 31.6, 28.9, 19.4, 18.7, 18.6, 11.4. IR (film) $\tilde{\nu}$ 2941, 2892, 2864, 2171, 1462, 1017, 995, 964, 924, 882, 674, 619, 492 cm^{-1}. HRMS (GC-CI) for $C_{17}H_{33}Si$ $[M]^+$: calcd. 265.23436; found: 265.23460.

1-((*E*)-Oct-6-en-1-yn-1-yl)adamantine (S14)

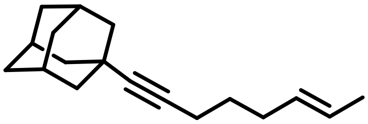

Prepared analogously from compounds **S4** and **S5** as a colorless oil (185 mg, 62%). ^{1}H NMR (400 MHz, $CDCl_3$) δ 5.51 – 5.34 (m, 2H), 2.14 (t, *J* = 7.1 Hz, 2H), 2.10 – 2.02 (m, 2H), 1.96 – 1.89 (m, 3H), 1.83 (d, *J* = 2.9 Hz, 6H), 1.71 – 1.62 (m, 8H), 1.56 – 1.47 (m, 3H). ^{13}C NMR (101 MHz, $CDCl_3$) δ 130.6, 125.3, 89.3, 78.6, 43.4, 36.4, 31.5, 29.4, 29.1, 28.1, 25.8, 18.1, 17.9. IR (film) $\tilde{\nu}$ 2902, 2850, 1451, 1437, 1344, 1100, 964, 507 cm^{-1}. HRMS (GC-EI) for $C_{18}H_{26}$ $[M]^+$: calcd. 242.20276; found: 242.20290.

2-Methoxy-2-((*E*)-oct-6-en-1-yn-1-yl)adamantine (S15)

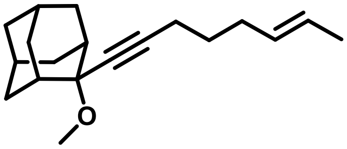

Prepared analogously from alkyne **S9** and bromide **S5** as a colorless oil (231 mg, 96%). ^{1}H NMR (400 MHz, $CDCl_3$) δ 5.54 – 5.44 (m, 1H), 5.37 (tddd, *J* = 9.0, 7.3, 3.3, 1.6 Hz, 1H), 3.34 (s, 3H), 2.27 (t, *J* = 7.0 Hz, 2H), 2.22 – 1.98 (m, 8H), 1.81 (p, *J* = 3.2 Hz, 1H), 1.75 (dddd, *J* = 11.4, 3.9, 2.6, 1.4 Hz, 3H), 1.68 (ddd, *J* = 4.3, 2.6, 1.6 Hz, 2H), 1.64 – 1.55 (m, 5H), 1.53 – 1.46 (m, 2H). ^{13}C NMR (101 MHz, $CDCl_3$) δ 129.7, 124.8, 86.8, 81.8, 78.0, 49.8, 37.8, 35.9, 35.3, 31.7, 29.0, 27.4, 26.8, 26.1, 18.4, 12.9. IR (film) $\tilde{\nu}$ 294, 2854, 1448, 1164, 1101, 1085, 877, 923, 699 cm^{-1}. HRMS (GC-EI) for $C_{19}H_{29}O$ $[M+H]^+$: calcd. 273.22129; found: 273.22129.

Mestranol derived enyne (S16)

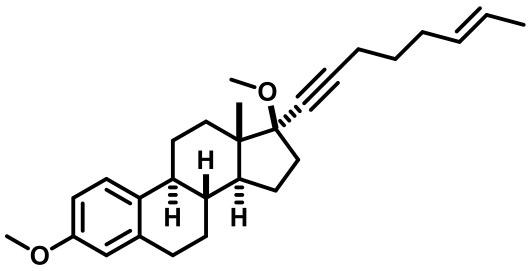

Prepared analogously from alkyne **S10** and bromide **S5** as a colorless oil (212 mg, 83%). ^{1}H NMR (400 MHz, $CDCl_3$) δ 7.21 (dd, *J* = 8.6, 1.0 Hz, 1H), 6.71 (dd, *J* = 8.6, 2.8 Hz, 1H), 6.63 (d, *J* = 2.7 Hz, 1H), 5.54 – 5.44 (m, 1H), 5.39 (dddd, *J* = 10.8, 7.2, 4.3, 1.6 Hz, 1H), 3.78 (s, 3H), 3.40 (s, 3H), 2.92 – 2.80 (m, 2H), 2.31 (t, *J* = 7.0 Hz, 3H), 2.26 – 2.10 (m, 4H), 2.07 – 1.93 (m, 2H), 1.91 – 1.82 (m, 1H), 1.81 – 1.71 (m, 3H), 1.68 – 1.57 (m, 5H), 1.52 – 1.27 (m, 4H), 0.87 (d, *J* = 0.7 Hz, 3H). ^{13}C NMR (101 MHz, $CDCl_3$) δ 157.5, 138.1, 132.8, 129.7, 126.5, 124.8, 113.9, 111.6, 88.1, 81.0, 75.8, 55.3, 53.4, 53.2, 49.7, 47.6, 43.7, 39.3, 37.0, 34.4, 30.0, 29.0, 27.4, 26.7, 26.1, 22.8, 18.5, 12.9. IR (film) $\tilde{\nu}$ 2933, 1496, 1453, 1255, 1238, 1102, 1086, 1039, 726, 694, 464 cm^{-1}. HRMS (GC-EI) for $C_{28}H_{38}O_2$ $[M]^+$: calcd. 406.28702; found: 406.28663.

(*E*)-2-(Dec-8-en-3-yn-2-yloxy)tetrahydro-2H-pyran (S17)

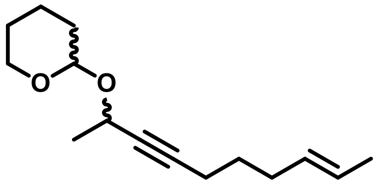

Prepared analogously from 2-(but-3-yn-2-yloxy)tetrahydro-2*H*-pyran (mixture of diastereoisomers)[15] and bromide **S5** as a colorless oil (mixture of diastereoisomers, 119 mg, 39%). ^{1}H NMR (400 MHz, $CDCl_3$) δ 5.52 – 5.32 (m, 2H), 5.00 – 4.72 (m, 1H), 4.59 – 4.40 (m, 1H), 4.08 – 3.76 (m, 1H), 3.60 – 3.39 (m, 1H), 2.25 – 2.13 (m, 2H), 2.10 – 2.00 (m, 2H), 1.92 – 1.68 (m, 2H), 1.66 – 1.63 (m, 3H), 1.62 – 1.49 (m, 5H), 1.45 – 1.37 (m, 3H). ^{13}C NMR (101 MHz, $CDCl_3$) δ 130.4, 130.3, 125.7, 125.6, 96.8, 95.8, 85.0, 81.0, 80.0, 62.6, 62.5, 62.3, 61.1, 31.6, 31.6, 30.6, 28.5, 28.5, 25.5, 25.5, 22.6, 22.2, 19.6, 19.3, 18.2, 18.1, 17.9. IR (film) $\tilde{\nu}$ 2937, 1440, 1335, 1116, 1073, 1019, 967, 873, 814 cm^{-1}. HRMS (GC-CI) for $C_{15}H_{24}O_2$ $[M+H]^+$: calcd. 237.18491; found: 237.18459.

(*E*)-1-(1-Methoxycyclohexyl)oct-6-en-1-yn-3-ol (S18)

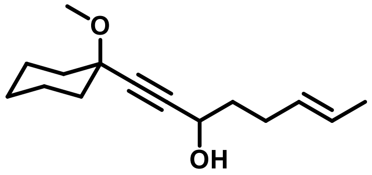

n-BuLi (1.55 M in hexanes, 4.67 mL, 7.24 mmol) was added dropwise to a solution of alkyne **S1** (1.00 g, 7.24 mmol) in THF (20 mL) at 0 °C. After stirring for 30 min, the mixture was cooled to –78 °C before a solution of (*E*)-hex-4-enal (923 mg, 9.41 mmol)[16] in THF (10 mL) was added dropwise. After warming to room temperature, the solution was stirred overnight before the reaction was quenched with sat. aq. NH_4Cl (10 mL), EtOAc (50 mL) and water (30 mL). The aqueous layer was extracted with EtOAc (3 × 50 mL) and the combined organic phases were washed with brine and dried over Na_2SO_4. The solvent was removed under reduced pressure and the residue was purified by flash chromatography (silica, pentane/*tert*-butyl methyl

ether 3:1) to provide the title compound as colorless oil (1.01 g, 59%). ^{1}H NMR (400 MHz, $CDCl_3$) δ 5.56 – 5.35 (m, 2H), 4.44 (t, *J* = 6.5 Hz, 1H), 3.35 (s, 3H), 2.17 (tq, *J* = 7.5, 3.8, 3.1 Hz, 2H), 1.92 – 1.83 (m, 2H), 1.83 – 1.61 (m, 8H), 1.52 (dtd, *J* = 19.7, 12.3, 9.6 Hz, 5H), 1.38 – 1.20 (m, 1H). ^{13}C NMR (101 MHz, $CDCl_3$) δ 130.2, 126.1, 87.1, 86.1, 74.0, 62.2, 50.8, 37.9, 36.9, 36.8, 28.5, 25.6, 22.9, 18.1. IR (film) $\tilde{\nu}$ 2934, 2875, 1444, 1291, 1184, 1162, 1079, 1055, 1030, 965, 923, 734, 522 cm^{-1}. HRMS (ESI^+) for $C_{15}H_{24}O_2$ $[M+Na]^+$: calcd. 259.16685; found: 259.16677.

(*E*)-Oct-6-en-1-yne (S19)

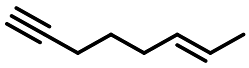

Trimethylsilylacetylene (1.61 mL, 11.4 mmol) was dissolved in THF (50 mL) and the solution cooled to 0°C before *n*-BuLi (1.59 M in hexanes, 8.60 mL, 13.7 mmol) was added dropwise. After stirring for 30 min, a solution of bromide **S5** (3.00 g, 14.7 mmol) in DMPU (6.89 mL, 57.0 mmol) and THF (3 mL) was added dropwise. The mixture was stirred at ambient temperature overnight. Sat. aq. NH_4Cl (30 mL), *tert*-butyl methyl ether (100 mL) and water (50 mL) were introduced, the aqueous layer was extracted with *tert*-butyl methyl ether (3 × 75 mL) and the combined organic phases were washed with brine and dried over Na_2SO_4. The residue was filtered through a short plug of silica, eluting with pentane/*tert*-butyl methyl ether (20:1, 100 mL). All volatile compounds were removed under reduced pressure and the residue was directly used in the next step without further purification.

K_2CO_3 (764 mg, 5.47 mmol) was added in one portion to a solution of the crude silyl alkyne (900 mg, 4.99 mmol) in MeOH (50 mL). After stirring overnight, water (20 mL) was added and the mixture was extracted with pentane (3 × 20 mL). Purification by distillation of the combined organic phases at atmospheric pressure provided the title compound as colorless oil (369 mg, 68%). ^{1}H NMR (400 MHz, $CDCl_3$) δ 5.52 – 5.34 (m, 2H), 2.18 (td, *J* = 7.2, 2.7 Hz, 2H), 2.08 (dtt, *J* = 7.6, 6.5, 1.2 Hz, 2H), 1.94 (t, *J* = 2.7 Hz, 1H), 1.67 – 1.62 (m, 3H), 1.58 (dt, *J* = 14.3, 7.2 Hz, 2H). ^{13}C NMR (101 MHz, $CDCl_3$) δ 130.3, 126.0, 84.7, 68.4, 31.6, 28.5, 18.1, 17.9. IR (film) $\tilde{\nu}$ 2956, 2748, 2379, 1439, 1355, 921, 802 cm^{-1}. HRMS (ESI^+) for C_8H_{13} $[M+H]^+$: calcd. 109.10110; found: 109.10118.

(*E*)-(2-Methoxydec-8-en-3-yn-2-yl)benzene (S20)

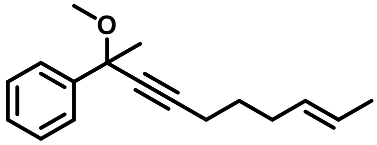

n-BuLi (1.59 M in hexanes, 330 µL, 525 µmol) was added dropwise to a solution of **S19** (73.3 mg, 573 µmol) in THF (2 mL) at 0°C. After stirring for 30 min, the mixture was cooled to –78 °C and a solution of acetophenone (43.4 µL, 372 µmol) in THF (0.5 mL) was added. After stirring at –78 °C for 90 min, the mixture was stirred overnight at ambient

temperature. Water (10 mL) was introduced and the mixture was extracted with EtOAc (3 × 50 mL). The combined organic layers were washed with brine and dried over Na_2SO_4. The solvent was removed under reduced pressure and the residue purified by flash chromatography (silica, pentane/*tert*-butyl methyl ether 3:1) to provide an inseparable mixture of the desired alcohol and remaining starting material.

A solution of this crude material (42.3 mg, 185 µmol) in THF (0.5 mL) was added dropwise to a suspension of NaH (13.3 mg, 556 µmol) in THF (1 mL) at 0 °C. After warming to ambient temperature, the mixture was stirred for additional 15 min, before it was cooled to 0 °C and methyl iodide (60 µL, 964 µmol) was added. The mixture was stirred overnight at ambient temperature before the reaction was quenched with water (10 mL) and *tert*-butyl methyl ether (20 mL). The aqueous layer was extracted with *tert*-butyl methyl ether (3 × 20 mL), the combined organic phases were washed with brine and dried over Na_2SO_4, the solvent was removed under reduced pressure and the residue was purified by flash chromatography (silica, pentane/*tert*-butyl methyl ether 15:1) to provide the title compound as colorless oil (37 mg, 82%). ^{1}H NMR (400 MHz, $CDCl_3$) δ 7.63 – 7.57 (m, 2H), 7.39 – 7.32 (m, 2H), 7.31 – 7.25 (m, 1H), 5.57 – 5.35 (m, 2H), 3.19 (s, 3H), 2.34 (t, *J* = 7.1 Hz, 2H), 2.14 (dtd, *J* = 7.7, 6.2, 1.4 Hz, 2H), 1.69 (s, 3H), 1.68 – 1.62 (m, 5H). ^{13}C NMR (101 MHz, $CDCl_3$) δ 143.4, 130.4, 128.3, 127.7, 126.3, 126.0, 88.1, 80.3, 76.7, 52.4, 33.1, 31.9, 28.8, 18.3, 18.1. IR (film) $\tilde{\nu}$ 2985, 2933, 1446, 1366, 1234, 1178, 1097, 975, 763, 699, 608 cm^{-1}. HRMS (ESI$^+$) for $C_{17}H_{22}O$ [M+Na]$^+$: calcd. 265.15628; found: 265.15624.

(*E*)-1-Chloro-4-(2-methoxydec-8-en-3-yn-2-yl)benzene (S21)

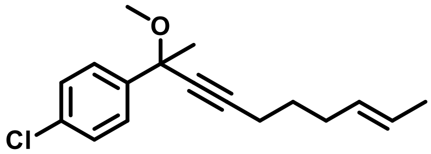

Prepared analogously from 1-(4-chlorophenyl)ethan-1-one as a colorless oil (205 mg, 79%). ^{1}H NMR (400 MHz, $CDCl_3$) δ 7.56 – 7.49 (m, 2H), 7.34 – 7.29 (m, 2H), 5.61 – 5.32 (m, 2H), 3.18 (s, 3H), 2.33 (t, *J* = 7.1 Hz, 2H), 2.18 – 2.09 (m, 2H), 1.69 – 1.60 (m, 8H). ^{13}C NMR (101 MHz, $CDCl_3$) δ 142.1, 133.5, 130.3, 128.4, 127.7, 126.0, 88.5, 79.9, 76.3, 52.4, 33.0, 31.9, 28.8, 18.3, 18.1. IR (film) $\tilde{\nu}$ 2988, 2934, 1488, 1451, 1232, 1001, 1014, 965, 866, 827, 606 cm^{-1}. HRMS (ESI$^+$) for $C_{17}H_{21}ClO$ [M+Na]$^+$: calcd. 299.11731; found: 299.11693.

(*E*)-1-(2-Methoxydec-8-en-3-yn-2-yl)-4-(trifluoromethyl)benzene (S22)

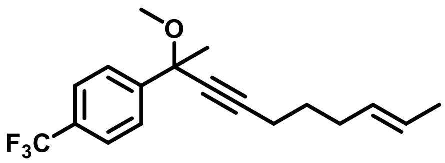

Prepared analogously from 1-(4-(trifluoromethyl)phenyl)-ethan-1-one as colorless oil (335 mg, 87%). ^{1}H NMR (400 MHz, $CDCl_3$ δ 7.75 – 7.67 (m, 2H), 7.65 – 7.57 (m, 2H), 5.56 – 5.34 (m, 2H), 3.21 (s, 3H), 2.34 (t, *J* = 7.1 Hz, 2H), 2.19 –

2.08 (m, 2H), 1.70 – 1.61 (m, 8H). ^{13}C NMR (101 MHz, $CDCl_3$) δ 147.7, 130.3, 129.9 (q, *J* = 32.1 Hz), 126.6, 126.1, 125.3 (q, *J* = 3.8 Hz), 123.0, 88.9, 79.6, 76.4, 52.6, 33.0, 31.9, 28.7, 18.3, 18.1. ^{19}F NMR (282 MHz, $CDCl_3$) δ −62.5. IR (film) $\tilde{\nu}$ 2936, 1619, 1409, 1323, 1163, 1124, 1097, 1078, 1066, 968, 622 cm^{-1}. HRMS (ESI$^+$) for $C_{18}H_{21}F_3O$ [M+Na]$^+$: calcd. 333.14367; found: 333.14350.

Methyl (*E*)-4-(2-methoxydec-8-en-3-yn-2-yl)benzoate (S23)

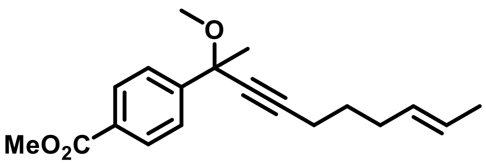

Prepared analogously from methyl 4-acetylbenzoate as colorless oil (66.5 mg, 36%). ^{1}H NMR (400 MHz, $CDCl_3$) δ 8.06 – 7.98 (m, 2H), 7.73 – 7.58 (m, 2H), 5.53 – 5.36 (m, 2H), 3.92 (s, 3H), 3.20 (s, 3H), 2.34 (t, *J* = 7.1 Hz, 2H), 2.21 – 2.08 (m, 2H), 1.70 – 1.61 (m, 8H). ^{13}C NMR (101 MHz, $CDCl_3$) δ 167.1, 148.8, 130.3, 129.7, 129.6, 126.3, 126.0, 88.7, 79.7, 76.6, 52.6, 52.3, 32.9, 31.9, 28.7, 18.3, 18.1. IR (film) 2989, 2935, 1722, 1610, 1435, 1274, 1234, 1094, 965, 773, 708 cm^{-1}. HRMS (ESI$^+$) for $C_{19}H_{24}O_3$ [M+Na]$^+$: calcd. 323.16176; found: 323.16216.

(*E*)-Oct-6-en-1-yn-1-ylferrocene (S24)

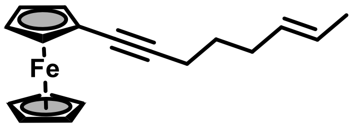

n-BuLi (1.57 M in hexanes, 906 µL, 1.42 mmol) was added dropwise to a solution of ethynylferrocene (249 mg, 1.19 mmol) in THF (5 mL) at 0 °C. The mixture was stirred at this temperature for 20 min before a solution of bromide **S5** (232 mg, 1.42 mmol) in DMPU (716 µL) and THF (3 mL) was added dropwise. After stirring overnight at ambient temperature, the reaction was quenched with sat. aq. NH_4Cl (2 mL), *tert*-butyl methyl ether (10 mL) and water (2 mL). The aqueous layer was extracted with *tert*-butyl methyl ether (3 × 20 mL) and the combined organic phases were washed with brine and dried over Na_2SO_4. The solvent was removed under reduced pressure and the residue purified by flash chromatography (silica, pentane/*tert*-butyl methyl ether 50:1) to provide the title compound as orange oil (254 mg, 73%). ^{1}H NMR (400 MHz, C_6D_6) δ 5.52 – 5.23 (m, 2H), 4.40 (s, 2H), 4.10 (s, 5H), 3.89 (s, 2H), 2.21 (t, *J* = 7.1 Hz, 2H), 2.12 – 2.05 (m, 2H), 1.65 – 1.49 (m, 5H). ^{13}C NMR (101 MHz, C_6D_6) δ 130.8, 125.8, 86.4, 79.2, 71.6, 70.1, 68.5, 67.4, 32.0, 29.2, 19.3, 18.0. IR (film) $\tilde{\nu}$ 3096, 2931, 2856, 1452, 1436, 1412, 1326, 1257, 1106, 1001, 965, 817, 488 cm^{-1}. HRMS (ESI$^+$) for $C_{18}H_{20}Fe$ [M]$^+$: calcd. 292.09089; found: 292.09091.

(*E*)-1-Methoxy-1-(non-7-en-1-yn-1-yl)cyclohexane (S25)

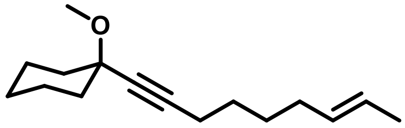

n-BuLi (1.55 M in hexanes, 390 µL, 605 µmol) was added dropwise to a solution of alkyne **S1** (83.7 mg, 606 µmol) in THF (2 mL) at 0 °C. The mixture was stirred at this temperature for

30 min before a solution of bromide **S6** (123 mg, 696 µmol) in DMPU (300 µL) and THF (1.7 mL) was added dropwise. After warming to ambient temperature, the mixture was stirred overnight. Sat. aq. NH_4Cl (2 mL), *tert*-butyl methyl ether (20 mL) and water (10 mL) were added, the aqueous layer was extracted with *tert*-butyl methyl ether (3 × 20 mL) and the combined organic phases were washed with brine and dried over Na_2SO_4. The solvent was removed under reduced pressure and the residue was purified by flash chromatography (silica, pentane/*tert*-butyl methyl ether 30:1) to provide the title compound as colorless oil (109 mg, 77%). ^{1}H NMR (400 MHz, $CDCl_3$) δ 5.60 – 5.25 (m, 2H), 3.35 (s, 3H), 2.23 (t, *J* = 6.8 Hz, 2H), 1.99 (tdt, *J* = 6.7, 5.3, 1.6 Hz, 2H), 1.92 – 1.77 (m, 2H), 1.66 – 1.62 (m, 5H), 1.55 – 1.41 (m, 9H), 1.34 – 1.12 (m, 1H). ^{13}C NMR (101 MHz, $CDCl_3$) δ 131.1, 124.9, 86.3, 81.0, 74.0, 37.0, 32.0, 28.7, 28.3, 25.5, 22.9, 18.5, 17.9. IR (film) $\tilde{\nu}$ 2932, 2856, 1447, 1292, 1184, 1092, 1081, 965, 925, 906 cm^{-1}. HRMS (GC-CI) for $C_{16}H_{26}O$ $[M+H]^+$: calcd. 235.20564; found: 235.20532.

(*E*)-1-(Methoxymethoxy)-1-(non-7-en-1-yn-1-yl)cyclohexane (S26)

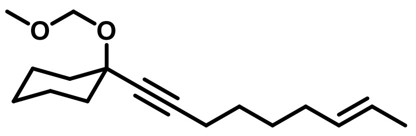

Prepared analogously from alkyne **S2** and bromide **S6** as a colorless oil (109 mg, 82%). ^{1}H NMR (400 MHz, $CDCl_3$) δ 5.48 – 5.35 (m, 2H), 4.93 (s, 2H), 3.39 (s, 3H), 2.23 (t, *J* = 6.9 Hz, 2H), 2.04 – 1.95 (m, 2H), 1.91 (dt, *J* = 11.0, 3.7 Hz, 2H), 1.75 – 1.41 (m, 14H), 1.37 – 1.18 (m, 1H). ^{13}C NMR (101 MHz, $CDCl_3$) δ 131.2, 125.1, 92.8, 87.4, 80.9, 75.4, 55.8, 39.1, 32.1, 28.8, 28.3, 25.5, 23.3, 18.7, 18.0. IR (film) $\tilde{\nu}$ 2931, 2857, 1448, 1294, 1177, 1149, 1097, 1068, 1026, 965 cm^{-1}. HRMS (ESI$^+$) for $C_{17}H_{28}O_2$ $[M+Na]^+$: calcd. 287.19815; found: 287.19781.

1-(4-(But-2-en-1-yloxy)but-1-yn-1-yl)-1-methoxycyclohexane (S27)

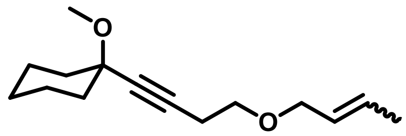

NaH (98.8 mg, 4.12 mmol) was added in portions to a solution of 4-(1-methoxycyclohexyl)but-3-yn-1-ol (500 mg, 2.74 mmol)[17] in DMF (14 mL) at 0 °C and the resulting suspension was stirred for 30 min. Crotyl bromide (mixture of *(E/Z)*-isomers, 423 µL, 4.12 mmol). was added and stirring continued at ambient temperature overnight. Water (20 mL) was introduced and the mixture was extracted with *tert*-butyl methyl ether (3 × 50 mL). The combined organic phases were washed with brine and dried over Na_2SO_4. The solvent was removed under reduced pressure and the residue purified by flash chromatography (silica, pentane/*tert*-butyl methyl ether 25:1) to provide the title compound as colorless oil (mixture of *(E/Z)*-isomers, 453 mg, 70%). ^{1}H NMR (400 MHz, $CDCl_3$) δ 5.78 – 5.61 (m, 1H), 5.56 (dddd, *J* = 15.2, 7.9, 4.6, 1.6 Hz, 1H), 4.19 – 3.85 (m, 2H), 3.66 – 3.45 (m, 2H), 3.33 (s, 3H), 2.58 – 2.45 (m, 2H), 1.95 – 1.78 (m, 2H), 1.76 – 1.41 (m, 10H), 1.26 (tt, *J* = 9.4, 4.9 Hz, 1H). ^{13}C NMR (101 MHz, $CDCl_3$) δ 129.8, 128.1, 127.5, 126.8, 83.1, 82.2, 74.1,

71.7, 68.9, 68.6, 66.3, 50.6, 37.0, 25.7, 23.0, 20.3, 17.9, 13.3. IR (film) $\tilde{\nu}$ 2933, 2856, 1447, 1293, 1184, 1135, 1092, 1086, 1027, 966, 925, 819 cm^{-1}. HRMS (ESI^+) for $C_{15}H_{24}O_2$ $[M+Na]^+$: calcd. 259.16685; found: 259.16676.

Dimethyl (*E*)-2-(but-2-en-1-yl)-2-(4-(1-hydroxycyclohexyl)but-3-yn-1-yl)malonate (S28)

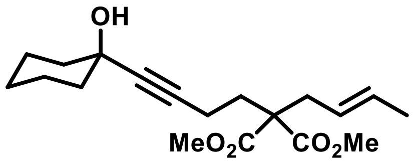

A solution of LiHMDS (674 mg, 4.03 mmol) in THF (4 mL) was added dropwise at –78 °C to a solution of dimethyl (*E*)-2-(but-2-en-1-yl)-2-(but-3-yn-1-yl)malonate (mixture of *(E/Z)*-isomers, 800 mg, 3.36 mmol)[18] in THF (30 mL). After stirring for 30 min at this temperature, cyclohexanone (522 µL, 5.04 mmol) was added dropwise and the mixture was warmed to ambient temperature. The reaction was quenched with sat. aq. NH_4Cl (2 mL), EtOAc (50 mL) and water (20 mL), the aqueous phase was extracted with EtOAc (3 × 50 mL) and the combined organic layers were dried over Na_2SO_4. The solvent was removed under reduced pressure and the residue purified by flash chromatography (silica, pentane/*tert*-butyl methyl ether 15:1 – 10:1 – 3:1) to provide the title compound as colorless oil (mixture of *(E/Z)*-isomers, 439 mg, 39%). ^1H NMR (400 MHz, $CDCl_3$) δ 5.68 – 5.48 (m, 1H), 5.25 (dddd, *J* = 16.5, 8.9, 4.4, 2.1 Hz, 1H), 3.72 (s, 6H), 2.73 – 2.54 (m, 2H), 2.25 – 2.07 (m, 4H), 1.87 – 1.77 (m, 3H), 1.71 – 1.59 (m, 5H), 1.59 – 1.44 (m, 5H), 1.33 – 1.17 (m, 1H). ^{13}C NMR (101 MHz, $CDCl_3$) δ 171.6, 130.2, 128.4, 124.4, 123.4, 84.6, 83.2, 68.8, 57.5, 57.2, 52.7, 52.6, 40.3, 36.2, 31.8, 31.8, 30.3, 25.4, 23.5, 18.2, 14.4, 14.3, 13.0. IR (film) $\tilde{\nu}$ 2933, 1720, 1444, 1270, 1236, 1199, 1178, 1132, 1066, 964, 904 cm^{-1}. HRMS (ESI^+) for $C_{19}H_{28}O_5$ $[M+Na]^+$: calcd. 359.18289; found: 359.18247.

Dimethyl (*E*)-2-(but-2-en-1-yl)-2-(4-(1-methoxycyclohexyl)but-3-yn-1-yl)malonate (S29)

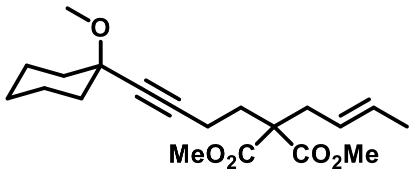

A solution of **S28** (mixture of *(E/Z)*-isomers, 389 mg, 1.16 mmol) in THF (2 mL) was added dropwise to a suspension of NaH (85.1 mg, 3.55 mmol) in THF (4.5 mL) at 0 °C. After 5 min, the mixture was warmed to ambient temperature and stirred for additional 10 min. It was then cooled to 0°C before MeI (370 µL, 5.95 mmol) was added dropwise. The mixture was stirred at ambient temperature overnight. Water (20 mL) was introduced and the aqueous layer was extracted with *tert*-butyl methyl ether (3 × 50 mL). The combined organic phases were washed with brine and dried over Na_2SO_4. The solvent was removed under reduced pressure and the residue purified by flash chromatography (silica, pentane/*tert*-butyl methyl ether 20:1) to provide the title compound as colorless oil (mixture of *(E/Z)*-isomers, 318 mg, 79%). ^1H NMR (400 MHz, $CDCl_3$) δ 5.69 – 5.46 (m, 1H), 5.26 (ddddt, *J* = 14.7, 7.5, 5.6, 3.9, 1.9 Hz, 1H), 3.72 (s, 6H), 3.33 (s, 3H), 2.76 – 2.52 (m, 2H), 2.26 – 2.06 (m,

4H), 1.91 – 1.77 (m, 2H), 1.68 – 1.43 (m, 10H), 1.35 – 1.23 (m, 1H). ^{13}C NMR (101 MHz, $CDCl_3$) δ 171.5, 171.5, 130.2, 128.4, 124.4, 123.4, 85.0, 81.7, 74.0, 57.6, 57.2, 52.6, 52.6, 50.6, 37.0, 36.3, 32.2, 32.1, 30.3, 25.7, 22.9, 18., 14., 14.3, 13.0. IR (film) $\tilde{\nu}$ 3372, 2971, 2936, 1734, 1444, 1083, 1047, 969, 924, 880 cm^{-1}. HRMS (ESI^+) for $C_{20}H_{30}O_5$ $[M+Na]^+$: calcd. 373.19854; found: 373.19819.

(*E*)-*N*-(But-2-en-1-yl)-*N*-(4-(1-methoxycyclohexyl)but-3-yn-1-yl)-4-methylbenzenesulfonamide (S30)

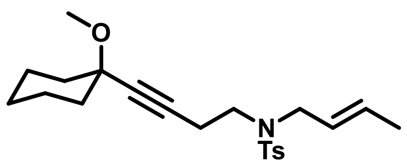

N-(4-(1-Methoxycyclohexyl)but-3-yn-1-yl)-4-methylbenzenesulfonamide (**S3**) (270 mg, 805 µmol) and K_2CO_3 (412 mg, 2.98 mmol) were suspended in MeCN (3 mL). Crotyl bromide (mixture of (*E/Z*)-isomers, 300 µL, 2.92 mmol) was added and the mixture was stirred at 60 °C overnight. After cooling to room temperature, the mixture was filtered through a pad of cotton wool and the residue in the filter was washed with MeCN (15 mL). The combined filtrates was concentrated and the crude material was purified by flash chromatography (silica, pentane/*tert*-butyl methyl ether 10:1 – 3:1) to provide the title compound as colorless oil (mixture of (*E*/*Z)*-isomers, 269 mg, 86%). ^{1}H NMR (400 MHz, $CDCl_3$) δ 7.77 – 7.68 (m, 2H), 7.34 – 7.28 (m, 2H), 5.68 – 5.55 (m, 1H), 5.33 – 5.23 (m, 1H), 3.83 (ddt, *J* = 46.8, 6.6, 1.1 Hz, 2H), 3.31 (s, 3H), 3.30 – 3.24 (m, 2H), 2.57 – 2.46 (m, 2H), 2.43 (s, 3H), 1.88 – 1.77 (m, 2H), 1.71 – 1.43 (m, 10H), 1.35 – 1.22 (m, 1H). ^{13}C NMR (101 MHz, $CDCl_3$) δ 143.3, 143.2, 137.2, 137.1, 130.6, 129.7, 129.7, 128.8, 127.1, 125.6, 124.8, 82.9, 82.6, 73.9, 50.5, 50.5, 46.5, 46.1, 44.8, 36.8, 25.5, 22.7, 21.5, 19.7, 19.6, 17.7, 12.9. IR (film) $\tilde{\nu}$ 2935, 1449, 1341, 1202, 1157, 1081, 928, 816, 743, 655, 548 cm^{-1}. HRMS (ESI^+) for $C_{22}H_{31}NO_3S$ $[M+H]^+$: calcd. 412.19169; found: 412.19197.

3.2 Synthesis of the Precatalysts

Since preliminary results showed that the purity of the catalyst has a significant impact on the outcome of the catalytic transformation, a modified and optimized synthesis procedure for [(IPr)(cymene)$RuCl_2$] was developed. In our hands, literature protocols for the synthesis of this complex did not provide material of acceptable quality.[19]

[(IPr)(η^6-cymene)$RuCl_2$] (3c)

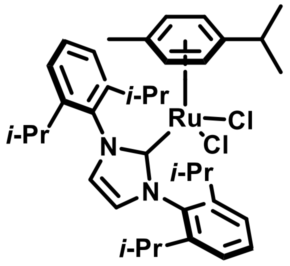

[(*p*-Cymene)$RuCl_2$]$_2$ (487 mg, 795 µmol) was suspended in THF (15 mL) and a solution of IPr (607 mg, 1.55 mmol) in THF (15 mL) was added dropwise within 5 min. The resulting mixture was stirred for 1 h at ambient temperature in the dark. All volatile components were removed under high vacuum. The residue was redissolved in a minimum amount of CH_2Cl_2 and the turbid solution was filtered through a pad of neutral aluminium oxide. Eluting with CH_2Cl_2/MeOH (50:1, 50 mL) provided an orange solution, which was concentrated under high vacuum. The residue was redissolved in a minimum amount of benzene and filtered through a short pad of Celite, which was eluted with benzene (80 mL). The orange solution was concentrated under high vacuum until the product started to precipitate. At this point, the solution was cooled to 0 °C before pentane (150 mL) was added until an orange solid precipitated from the mixture. The supernatant was siphoned off and the resulting solid was washed with pentane (2 × 25 mL) and dried under high vacuum. The title compound was obtained as orange solid (715 mg, 66%). ^{1}H NMR (400 MHz, C_6D_6) δ 7.24 (dd, *J* = 8.4, 7.0 Hz, 2H), 7.15 – 7.11 (m, 4H), 6.49 (s, 2H), 4.86 (d, *J* = 5.8 Hz, 2H), 4.52 (d, *J* = 5.8 Hz, 2H), 3.38 (hept, *J* = 6.7 Hz, 4H), 2.65 (hept, *J* = 6.8 Hz, 1H), 1.77 (s, 3H), 1.49 (d, *J* = 6.7 Hz, 12H), 1.07 (d, *J* = 7.0 Hz, 6H), 1.02 (d, *J* = 6.8 Hz, 12H). ^{13}C NMR (101 MHz, C_6D_6) δ 173.7, 146.4, 140.2, 129.8, 126.5, 123.8, 105.4, 96.9, 86.5, 84.3, 30.4, 28.9, 26.4, 23.5, 22.9, 18.8. The spectroscopic data are consistent with those reported in the literature.[19]

During this study, the authors used several batches of **3c** and did not notice any significant decomposition or change in the catalytic performance when the material was stored in the dark at –20 °C.

Complexes of the type [(NHC)(p-cymene)$RuCl_2$] are moderately light-sensitive in solution. Solutions of such complexes should therefore be handled in the dark; the light of the fume-hood should be switched off during any manipulations.

[(IMes)(η⁶-cymene)RuCl₂] (3b). Prepared analogously; the spectroscopic data are consistent with those reported in the literature.[19]

4. Hydrogenative Metathesis Reactions

General Procedure: Intramolecular Hydrogenative Enyne Metathesis

A flame-dried quartz Schlenk tube was charged with [(IPr)(*p*-cymene)$RuCl_2$] (13.8 mg, 0.02 mmol, 10 mol%), the substrate (0.2 mmol) and toluene (2 mL, 0.1 M). The Schlenk tube was closed with a septum and then transferred into the photolysis apparatus. A hydrogen-filled balloon was connected to a needle which was pierced through the septum and the Schlenk tube was flushed with hydrogen for 2 min through an outlet cannula (the cannula did not reach into the solution to make sure that only the head space of the tube was flushed). After the first 10 seconds of flushing with hydrogen, the light source was switched on and the reaction mixture was stirred for 60 min under hydrogen atmosphere. The reaction mixture was diluted with pentane (5 mL) and then filtered through a short pad of silica. The filtrate was evaporated and the residue purified by flash chromatography.

1-(Cyclopent-1-en-1-ylmethyl)-1-methoxycyclohexane (2)

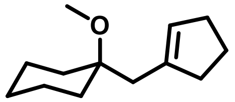

Prepared according to the General Procedure from enyne **1b** (44.1 mg, 200 µmol); colorless oil (36.9 mg, 95%). When the reaction was carried out on a 1 mmol scale (220 mg of **1b**), a yield of 88% (172 mg) was obtained.

When the same reaction was performed in ordinary laboratory glassware (instead of the quartz Schlenk-tube), a yield of 75% was obtained.

The analytical data of **2** matched those previously reported in the literature.[21]

The following compounds were prepared analogously:

2-((1-Methoxycyclohexyl)methyl)cyclopent-2-en-1-ol (5)

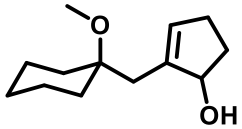

Prepared from substrate **S18**; colorless oil (29.5 mg, 70%). ^{1}H NMR (400 MHz, $CDCl_3$) δ 5.60 (s, 1H), 4.57 – 4.49 (m, 1H), 4.06 (s, 1H), 3.25 (s, 3H), 2.59 – 2.43 (m, 1H), 2.42 – 2.30 (m, 2H), 2.25 – 2.06 (m, 2H), 1.91 – 1.65 (m, 4H), 1.61 – 1.22 (m, 7H). ^{13}C NMR (101 MHz, $CDCl_3$) δ 141.9, 132.9, 78.4, 76.3, 48.2, 36.3, 34.4, 33.6, 33.5, 30.1, 25.8, 22.5, 22.1. IR (film) $\tilde{\nu}$ 3423, 2930, 2853, 1456, 1146, 1131, 1079, 951 cm^{-1}. HRMS (ESI$^+$) for $C_{13}H_{22}O_2$ $[M+Na]^+$: calcd. 233.15120; found: 233.15141.

2-(Cyclopent-1-en-1-ylmethyl)-2-methoxyadamantane (6)

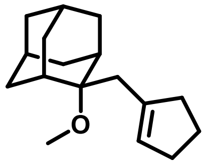

Prepared from substrate **S15**; colorless oil (37.1 mg, 75%). ^{1}H NMR (400 MHz, $CDCl_3$) δ 5.44 (td, *J* = 2.3, 1.2 Hz, 1H), 3.19 (s, 3H), 2.51 (s, 2H), 2.41 – 2.24 (m, 4H), 2.11 (dd, *J* = 12.7, 3.2 Hz, 2H), 1.94 – 1.76 (m, 8H), 1.74 – 1.62 (m, 4H), 1.50 – 1.41 (m, 2H). ^{13}C NMR (101 MHz, $CDCl_3$) δ 140.5, 127.3, 79.4, 47.2, 38.6, 36.6, 34.6, 34.0, 32.9, 32.4, 31.5, 27.8, 27.4, 23.8. IR (film) $\tilde{\nu}$ 2905, 2852, 1455, 1115, 1083, 1061, 993 cm^{-1}. HRMS (ESI$^+$) for $C_{17}H_{26}ONa$ [M]$^+$: calcd. 269.18767; found: 269.18758.

3-(2-Methoxy-2-methylpropyl)-1*H*-indene (7)

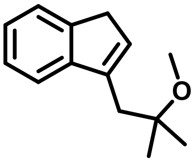

Prepared from substrate **S11**; colorless oil (27.0 mg, 66%). ^{1}H NMR (400 MHz, $CDCl_3$) δ 7.46 (dt, *J* = 7.4, 1.0 Hz, 1H), 7.42 (dt, *J* = 7.7, 0.9 Hz, 1H), 7.33 – 7.28 (m, 1H), 7.19 (td, *J* = 7.4, 1.2 Hz, 1H), 6.40 – 6.35 (m, 1H), 3.39 – 3.34 (m, 2H), 3.31 (s, 3H), 2.77 (d, *J* = 1.4 Hz, 2H), 1.24 (s, 6H). ^{13}C NMR (101 MHz, $CDCl_3$) δ 146.4, 144.1, 140.7, 131.7, 126.0, 124.4, 123.7, 119.6, 75.5, 49.5, 38.1, 37.5, 25.3. IR (film) $\tilde{\nu}$ 2972, 2937, 1717, 1462, 1383, 1366, 1241, 1161, 1070, 1020, 879, 764 cm^{-1}. HRMS (GC-EI) for $C_{14}H_{18}O$ [M]$^+$: calcd. 202.13490; found: 202.13522.

(1-(Cyclopent-1-en-1-yl)-2-methoxypropan-2-yl)benzene (8a)

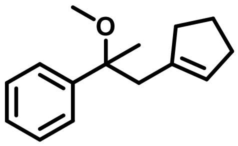

Prepared from substrate **S20**; colorless oil (36.8 mg, 85%). ^{1}H NMR (400 MHz, $CDCl_3$) δ 7.40 – 7.23 (m, 4H), 7.23 – 7.09 (m, 1H), 5.27 – 5.22 (m, 1H), 3.00 (s, 3H), 2.60 – 2.43 (m, 2H), 2.23 – 2.11 (m, 2H), 2.06 – 1.94 (m, 1H), 1.83 – 1.49 (m, 3H), 1.47 (s, 3H). ^{13}C NMR (101 MHz, $CDCl_3$) δ 145.2, 140.7, 128.4, 128.0, 126.9, 126.5, 79.3, 50.4, 45.0, 36.4, 32.5, 23.9, 22.4. IR (film) $\tilde{\nu}$ 2977, 2934, 2846, 2824, 1445, 1371, 1155, 1098, 1072, 764 cm^{-1}. HRMS (ESI$^+$) for $C_{15}H_{21}O$ [M+Na]$^+$: calcd. 217.15869; found: 217.15847.

1-Chloro-4-(1-(cyclopent-1-en-1-yl)-2-methoxypropan-2-yl)benzene (8b)

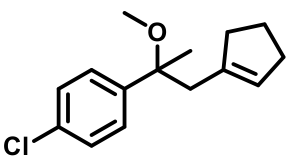

Prepared from substrate **S21**; colorless oil (42.0 mg, 85%). ^{1}H NMR (400 MHz, $CDCl_3$) δ 7.29 (s, 4H), 5.34 – 5.26 (m, 1H), 3.06 (s, 3H), 2.60 – 2.46 (m, 2H), 2.26 – 2.16 (m, 2H), 2.12 – 2.00 (m, 1H), 1.89 – 1.78 (m, 1H), 1.77 – 1.65 (m, 2H), 1.51 (s, 3H). ^{13}C NMR (101 MHz, $CDCl_3$) δ 143.9, 140.3, 132.7, 128.8, 128.2, 128.0, 79.0, 50.5, 45.0, 36.5, 32.5, 24.0, 22.5. IR (film) $\tilde{\nu}$ 2932, 1842, 1489, 1398, 1153, 1114, 1085, 1055, 1013, 829 cm^{-1}. HRMS (ESI$^+$) for $C_{15}H_{19}ClO$ [M+Na]$^+$: calcd. 273.10166; found: 273.10150.

1-(1-(Cyclopent-1-en-1-yl)-2-methoxypropan-2-yl)-4-(trifluoromethyl)benzene (8c)

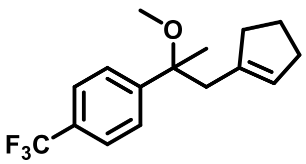

Prepared from substrate **S22**; colorless oil (48.0 mg, 85%). ^{1}H NMR (400 MHz, $CDCl_3$) δ 7.62 – 7.55 (m, 2H), 7.53 – 7.43 (m, 2H), 5.35 – 5.23 (m, 1H), 3.09 (s, 3H), 2.63 – 2.50 (m, 2H), 2.26 – 2.18 (m, 2H), 2.14 – 2.02 (m, 1H), 1.92 – 1.80 (m, 1H), 1.79 – 1.65 (m, 2H), 1.55 (s, 3H). ^{13}C NMR (101 MHz, $CDCl_3$) δ 149.7, 140.1, 129.0, 126.8, 129.2 (q, *J* = 32.2 Hz), 125.1 (q, *J* = 3.7 Hz), 124.4 (q, *J* = 272.2 Hz), 79.2, 50.6, 44.8, 36.5, 32.5, 24.0, 22.6. ^{19}F NMR (282 MHz, $CDCl_3$) δ –62.4. IR (film) $\tilde{\nu}$ 2937, 1619, 1410, 1324, 1163, 1119, 1075, 1016, 844 cm^{-1}. HRMS (APPI$^+$) for $C_{16}H_{19}F_3O$ $[M+Na]^+$: calcd. 284.13825; found: 284.13788.

Methyl 4-(1-(cyclopent-1-en-1-yl)-2-methoxypropan-2-yl)benzoate (8d)

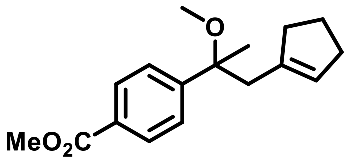

Prepared from substrate **S23**; colorless oil (42.6 mg, 80%). ^{1}H NMR (400 MHz, $CDCl_3$) δ 8.04 – 7.94 (m, 2H), 7.49 – 7.41 (m, 2H), 5.37 – 5.21 (m, 1H), 3.91 (s, 3H), 3.08 (s, 3H), 2.64 – 2.49 (m, 2H), 2.27 – 2.13 (m, 2H), 2.09 – 1.96 (m, 1H), 1.86 – 1.75 (m, 1H), 1.75 – 1.61 (m, 2H), 1.55 (s, 3H). ^{13}C NMR (101 MHz, $CDCl_3$) δ 167.2, 150.8, 140.2, 129.5, 128.9, 128.8, 126.6, 79.4, 52.2, 50.6, 44.9, 36.5, 32.5, 24.0, 22.5. IR (film) $\tilde{\nu}$ 2948, 1723, 1435, 1277, 1109, 1087, 1056, 775, 711 cm^{-1}. HRMS (GC-CI) for $C_{17}H_{22}O_3$ $[M+H]^+$: calcd. 275.16417; found: 275.16440.

2-((1-(Cyclopent-1-en-1-yl)propan-2-yl)oxy)tetrahydro-2*H*-pyran (9)

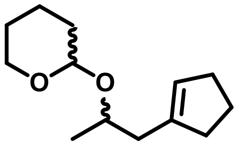

Prepared from substrate **S17**; colorless oil (mixture of diastereoisomers, 25.4 mg, 60%). ^{1}H NMR (400 MHz, $CDCl_3$) δ 5.45 – 5.36 (m, 1H), 4.77 – 4.61 (m, 1H), 4.00 – 3.81 (m, 2H), 3.48 (dddd, *J* = 9.7, 8.3, 4.2, 2.9 Hz, 1H), 2.50 – 2.09 (m, 6H), 1.90 – 1.78 (m, 3H), 1.74 – 1.45 (m, 5H), 1.22 – 1.06 (m, 3H). ^{13}C NMR (101 MHz, $CDCl_3$) δ 141.7, 141.4, 126.2, 126.1, 98.3, 95.8, 72.2, 70.1, 62.9, 62.2, 39.5, 38.5, 35.7, 35.6, 32.6, 32.6, 31.3, 31.3, 25.8, 25.7, 23.8, 23.7, 21.8, 20.1, 19.7, 19.2. IR (film) $\tilde{\nu}$ 2928, 2846, 1372, 1121, 1076, 1020, 995, 869 cm^{-1}. HRMS (GC-CI) for $C_{13}H_{22}O_2$ $[M+H]^+$: calcd. 211.16926; found: 211.16921.

Compound 10

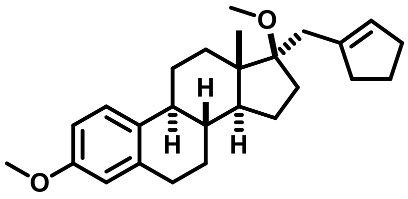

Prepared from substrate **S16**; white solid (52.0 mg, 68%). ^{1}H NMR (400 MHz, $CDCl_3$) δ 7.20 (dd, *J* = 8.7, 1.1 Hz, 1H), 6.71 (dd, *J* = 8.6, 2.8 Hz, 1H), 6.63 (d, *J* = 2.7 Hz, 1H), 5.61 – 5.50 (m, 1H), 3.78 (s, 3H), 3.26 (s, 3H), 2.89 – 2.83 (m, 2H), 2.75 (d, *J* = 15.9 Hz, 1H), 2.48 – 2.23 (m, 4H), 2.22 – 2.08 (m, 2H), 2.08 – 1.93 (m, 1H), 1.93 – 1.79 (m, 3H), 1.74 (td, *J* = 12.6, 4.0 Hz, 1H), 1.69 – 1.52 (m, 3H), 1.52 – 1.21 (m, 6H), 0.97 (s, 3H). ^{13}C NMR (101 MHz, $CDCl_3$) δ 157.5, 140.8, 138.1, 132.8, 127.2, 126.3, 113.9, 111.6, 88.0, 55.3, 51.5, 50.9, 47.5, 43.7, 39.6, 36.7, 34.5, 34.0, 32.6, 31.8, 30.0, 27.5, 26.7, 23.6, 23.4, 13.9. IR (film) $\tilde{\nu}$ 2933, 1504, 1245, 1158, 1093, 1081, 1063, 1036, 843, 451 cm^{-1}. HRMS (GC-EI) for $C_{26}H_{36}O_2$ $[M+Na]^+$: calcd. 403.26099; found: 403.26075.

(Cyclopent-1-en-1-ylmethyl)triisopropylsilane (11)

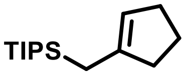

Prepared from substrate **S13**; colorless oil (39.5 mg, 84%). ^{1}H NMR (400 MHz, $CDCl_3$) δ 5.22 (q, *J* = 1.5 Hz, 1H), 2.26 (t, *J* = 7.4 Hz, 4H), 1.82 (p, *J* = 7.4 Hz, 2H), 1.66 (s, 2H), 1.05 (s, 21H). ^{13}C NMR (101 MHz, $CDCl_3$) δ 142.0, 122.6, 38.0, 32.6, 24.1, 18.8, 13.5, 11.6. IR (film) $\tilde{\nu}$ 2940, 2891, 2865, 1463, 1015, 998, 882, 745, 663 cm^{-1}. HRMS (GC-EI) for $C_{15}H_{30}Si$ $[M]^+$: calcd. 238.21074; found: 238.21113.

1-(Cyclopent-1-en-1-ylmethyl)adamantine (12)

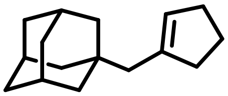

Prepared from substrate **S14**; colorless oil (42.1 mg, 95%). ^{1}H NMR (400 MHz, $CDCl_3$) δ 5.29 (tt, *J* = 2.1, 1.0 Hz, 1H), 2.34 – 2.22 (m, 4H), 1.96 – 1.90 (m, 3H), 1.88 – 1.79 (m, 4H), 1.73 – 1.58 (m, 6H), 1.48 (d, *J* = 2.8 Hz, 6H). ^{13}C NMR (101 MHz, $CDCl_3$) δ 141.7, 127.2, 46.0, 43.0, 38.3, 37.2, 33.4, 32.6, 29.0, 24.3. IR (film) $\tilde{\nu}$ 2896, 2844, 1449, 1345, 1315, 1101, 1036, 956, 820, 802, 467 cm^{-1}. HRMS (GC-EI) for $C_{16}H_{24}$ $[M]^+$: calcd. 216.18734; found: 216.18725.

(Cyclopent-1-en-1-ylmethyl)ferrocene (13)

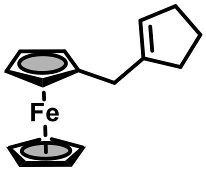

Prepared from substrate **S24**; orange oil (41.5 mg, 84%). The isolated product is only modestly stable and decomposes upon storage. ^{1}H NMR (400 MHz, $CDCl_3$) δ 5.26 – 5.22 (m, 1H), 4.10 (s, 5H), 4.07 (t, *J* = 1.8 Hz, 2H), 4.06 – 4.04 (m, 2H), 3.12 (q, *J* = 1.3 Hz, 4H), 2.31 – 2.17 (m, 2H). ^{13}C NMR (101 MHz, $CDCl_3$) δ 144.5, 124.1, 68.7, 68.6, 67.2, 35.1, 32.3, 31.9, 23.4. IR (film) $\tilde{\nu}$ 2922, 2843, 1105, 1038, 1022, 1009, 959, 814, 480 cm^{-1}. HRMS (EI) for $C_{16}H_{18}Fe$ $[M]^+$: calcd. 266.07579; found: 266.07570.

(Cyclopent-1-en-1-ylmethyl)benzene (14a) and 1-**Phenylcyclohexene (14'a)**

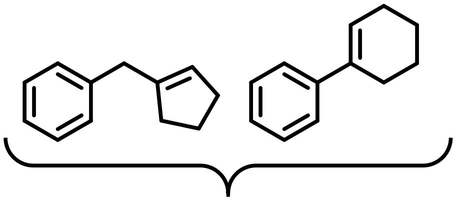

2:1

Prepared from substrate **S12a**; colorless oil (**14a**:**14'a** = 2:1, 21.0 mg, 66%). The analytical data of **14b** matched those reported in the literature,[20] as well as those of a commercial sample. Spectral data of **14a**: ^{1}H NMR (400 MHz, $CDCl_3$) δ 7.41 – 7.37 (m, 2H), 7.35 – 7.27 (m, 2H), 7.23 – 7.17 (m, 1H), 5.38 – 5.31 (m, 1H), 3.40 (d, *J* = 2.2 Hz, 2H), 2.36 – 2.29 (m, 2H) 2.26 – 2.17 (m, 2H), 1.93 – 1.84 (m, 2H). ^{13}C NMR (101 MHz, $CDCl_3$) δ 143.9, 140.3, 128.9, 125.9, 125.6, 125.0, 38.1, 34.9, 32.5, 23.6. HRMS (GC-EI) for $C_{12}H_{15}$ $[M+H]^+$: calcd. 159.11663; found: 159.11683.

1-(Cyclopent-1-en-1-ylmethyl)-4-methylbenzene (14b) and 4'-Methyl-2,3,4,5-tetrahydro-1,1'-biphenyl (14'b)

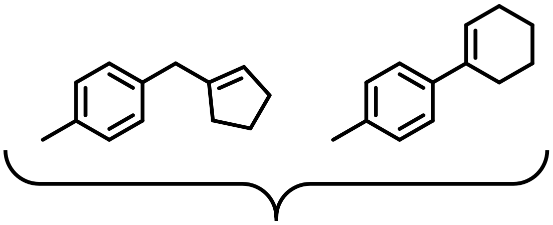

2:1

Prepared from substrate **S12b**; colorless oil (**14b**:**14'b** = 2:1, 27.0 mg, 78%). The analytical data of the compounds matched those reported in the literature.[21] ^{1}H NMR (400 MHz, $CDCl_3$) δ 7.09 (d, *J* = 3.2 Hz, 4H), 5.34 (hept, *J* = 1.9 Hz, 1H), 3.36 (s, 2H), 2.33 (s, 3H), 2.31 – 2.28 (m, 2H), 2.20 (tdd, *J* = 7.1, 3.6, 1.8 Hz, 2H), 1.90 – 1.83 (m, 2H).

1-(Cyclopent-1-en-1-ylmethyl)-4-methoxybenzene (14c) and 4'-Methoxy-2,3,4,5-tetrahydro-1,1'-biphenyl (14'c)

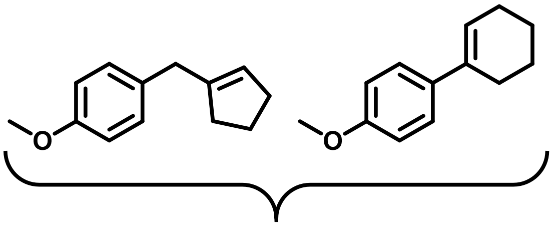

2:1

Prepared from substrate **S12c**; colorless oil (**14c**:**14'c** = 2:1, 23.0 mg, 61%). The analytical data matched those reported in the literature.[22] ^{1}H NMR (400 MHz, $CDCl_3$) δ 7.14 – 7.05 (m, 2H), 6.91 – 6.79 (m, 2H), 5.35 – 5.30 (m, *J* = 2.3 Hz, 1H), 3.79 (s, 3H), 3.33 (s, 2H), 2.31 (tp, *J* = 6.6, 2.2 Hz, 2H), 2.19 (tq, *J* = 5.8, 1.9 Hz, 2H), 1.86 (tt, *J* = 8.2, 6.7 Hz, 2H).

1-Chloro-4-(cyclopent-1-en-1-ylmethyl)benzene (14d) and 4'-Chloro-2,3,4,5-tetrahydro-1,1'-biphenyl (14'd)

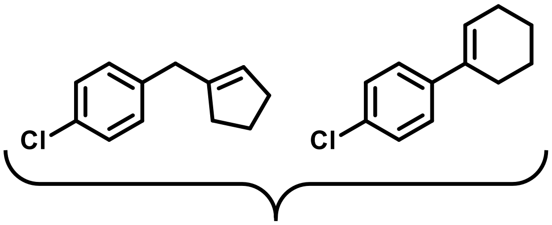

2:1

Prepared from substrate **S12d**; colorless oil (**14d**:**14'd** = 2:1, 31.7 mg, 41%; NMR yield with tetramethylbenzene as internal standard). In this case, flash chromatography did not allow trace amounts of by-products to be separated; however, the product structure could be confirmed by comparison of the NMR data with those reported in the literature.[23] ^{1}H NMR (400 MHz, $CDCl_3$) (characteristic signals only): δ 7.26 – 7.24 (m, 2H), 7.13 – 7.09 (m, 2H), 5.35 – 5.32 (m, 1H), 3.35 (s, 2H), 2.35 – 2.28 (m, 2H), 2.24 – 2.15 (m, 2H), 1.92 – 1.82 (m, 2H).

1-((1-Methoxycyclohexyl)methyl)cyclohex-1-ene (15a)

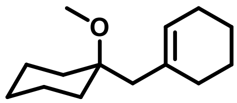

Prepared from substrate **S25**; colorless oil (32.0 mg, 77%). ^{1}H NMR (400 MHz, $CDCl_3$) δ 5.44 – 5.34 (m, 1H), 3.17 (s, 3H), 2.08 – 1.94 (m, 5H), 1.73 – 1.63 (m, 2H), 1.62 – 1.36 (m, 9H), 1.33 – 1.15 (m, 4H). ^{13}C NMR (101 MHz, $CDCl_3$) δ 134.6, 125.0, 75.8, 48.2, 44.1, 34.4, 30.3, 26.0, 25.6, 23.3, 22.4, 22.1. IR (film) $\tilde{\nu}$ 2925, 2854, 1454, 1146, 1123, 1079, 917, 811, 707 cm^{-1}. HRMS (ESI^+) for $C_{14}H_{24}O$ $[M+Na]^+$: calcd. 231.17193; found: 231.17224.

1-((1-(Methoxymethoxy)cyclohexyl)methyl)cyclohex-1-ene (15b)

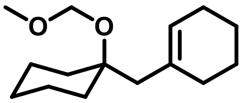

Prepared from substrate **S26** using 20 mol% of catalyst; colorless oil (24.6 mg, 79%). ^{1}H NMR (400 MHz, $CDCl_3$) δ 5.44 – 5.37 (m, 1H), 4.73 (s, 2H), 3.41 (s, 3H), 2.18 – 2.10 (m, 2H), 2.03 – 1.98 (m, 4H), 1.75 – 1.67 (m, 2H), 1.65 – 1.50 (m, 5H), 1.48 – 1.36 (m, 5H), 1.33 – 1.19 (m, 2H). ^{13}C NMR (101 MHz, $CDCl_3$) δ 134.4, 125.5, 90.7, 78.1, 55.8, 46.5, 35.4, 30.7, 25.8, 25.6, 23.3, 22.4. IR (film) $\tilde{\nu}$ 2926, 2856, 1448, 1157, 1140, 1085, 1032, 922 cm^{-1}. HRMS (ESI^+) for $C_{15}H_{26}O_2$ $[M]^+$: calcd. 261.18250; found: 261.18268.

4-((1-Methoxycyclohexyl)methyl)-1-tosyl-1,2,3,6-tetrahydropyridine (16)

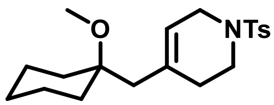

Prepared from substrate **S30**; colorless oil (49.0 mg, 75%). ^{1}H NMR (400 MHz, $CDCl_3$) δ 7.68 – 7.64 (m, 2H), 7.33 – 7.28 (m, 2H), 5.32 (tdd, J = 3.4, 1.7, 0.8 Hz, 1H), 3.56 (dt, J = 3.5, 1.3 Hz, 2H), 3.15 – 3.11 (m, 5H), 2.42 (s, 3H), 2.24 (tq, J = 4.6, 2.1 Hz, 2H), 2.06 (d, J = 1.5 Hz, 2H), 1.67 – 1.55 (m, 2H), 1.55 – 1.31

(m, 5H), 1.25 – 1.09 (m, 3H). ^{13}C NMR (101 MHz, $CDCl_3$) δ 143.5, 133.5, 133.4, 129.6, 127.8, 119.9, 75.6, 48.1, 45.0, 43.1, 42.6, 34.3, 29.9, 25.8, 21.9, 21.6. IR (film) $\tilde{\nu}$ 2931, 1685, 1342, 1292, 1162, 1132, 1071, 949, 814, 708, 687, 644 cm^{-1}. HRMS (ESI$^+$) for $C_{20}H_{29}NO_3S$ [M+Na]$^+$: calcd. 386.17604; found: 386.17615.

4-((1-Methoxycyclohexyl)methyl)-3,6-dihydro-2*H*-pyran (17)

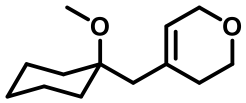

Prepared from substrate **S27**; colorless oil (31.6 mg, 76%). ^{1}H NMR (400 MHz, $CDCl_3$) δ 5.43 (tt, *J* = 2.9, 1.4 Hz, 1H), 4.13 (tt, *J* = 2.7, 1.4 Hz, 2H), 3.75 (t, *J* = 5.5 Hz, 2H), 3.18 (s, 3H), 2.20 – 2.15 (m, 2H), 2.12 (d, *J* = 1.6 Hz, 2H), 1.74 – 1.66 (m, 2H), 1.61 – 1.37 (m, 4H), 1.33 – 1.15 (m, 4H). ^{13}C NMR (101 MHz, $CDCl_3$) δ 132.6, 123.6, 75.8, 65.7, 64.7, 48.2, 43.1, 34.5, 30.3, 26.0, 22.1. IR (film) 2932, 2854, 2826, 1725, 1456, 1166, 1135, 1080, 912, 732 cm^{-1}. HRMS (ESI$^+$) for $C_{13}H_{22}O_2$ [M+Na]$^+$: calcd. 233.15120; found: 233.15108.

Dimethyl 4-((1-methoxycyclohexyl)methyl)cyclohex-3-ene-1,1-dicarboxylate (18)

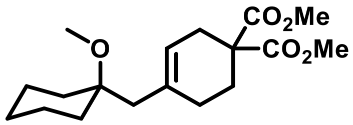

Prepared from substrate **S29** using 20 mol% of the catalyst; colorless oil (48.5 mg, 79%). ^{1}H NMR (400 MHz, $CDCl_3$) δ 5.39 – 5.33 (m, 1H), 3.69 (s, 6H), 3.13 (s, 3H), 2.63 – 2.45 (m, 2H), 2.11 (s, 4H), 2.04 (s, 2H), 1.64 – 1.56 (m, 2H), 1.55 – 1.33 (m, 4H), 1.27 – 1.13 (m, 4H). ^{13}C NMR (101 MHz, $CDCl_3$) δ 172.3, 134.1, 121.6, 75.8, 52.9, 52.7, 48.1, 43.1, 34.3, 31.1, 28.3, 26.9, 25.9, 22.0. IR (film) $\tilde{\nu}$ 2932, 1731, 1434, 1253, 1072, 911, 729 cm^{-1}. HRMS (ESI$^+$) for $C_{18}H_{28}O_5$ [M+Na]$^+$: calcd. 347.18289; found: 347.18293.

5. Copies of Spectra

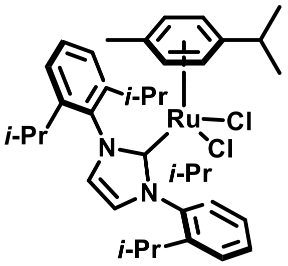
i-Pr
Ru
Cl
Cl
i-Pr
N
N
i-Pr
i-Pr

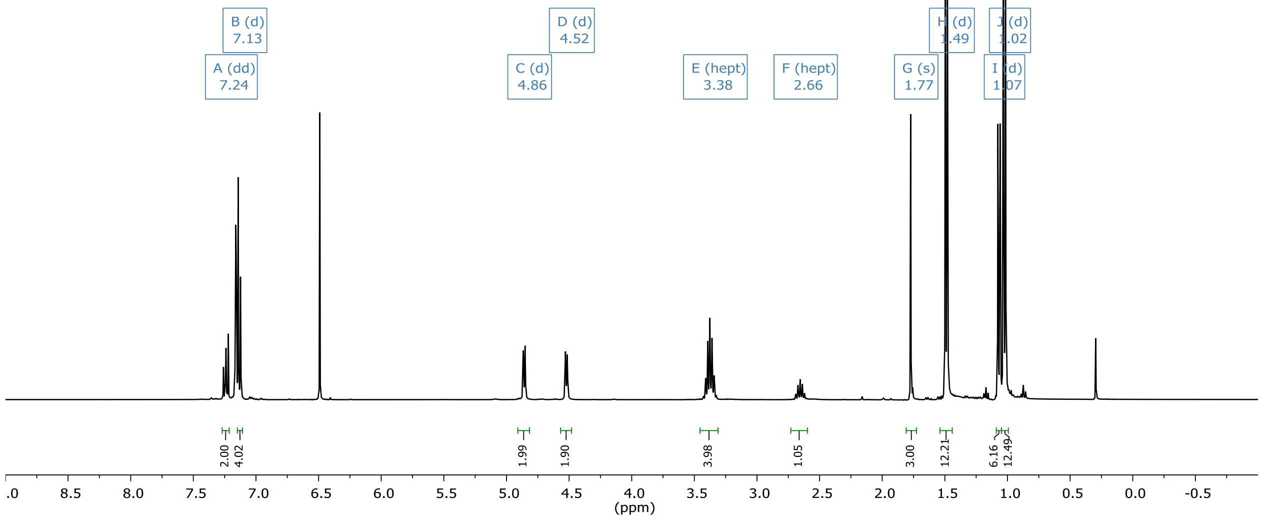
B (d) 7.13
A (dd) 7.24
D (d) 4.52
C (d) 4.86
E (hept) 3.38
F (hept) 2.66
G (s) 1.77
H (d) 1.49
J (d) 1.02
I (d) 1.07
2.00
4.02
1.99
1.90
3.98
1.05
3.00
12.21
6.16
12.49
(ppm)

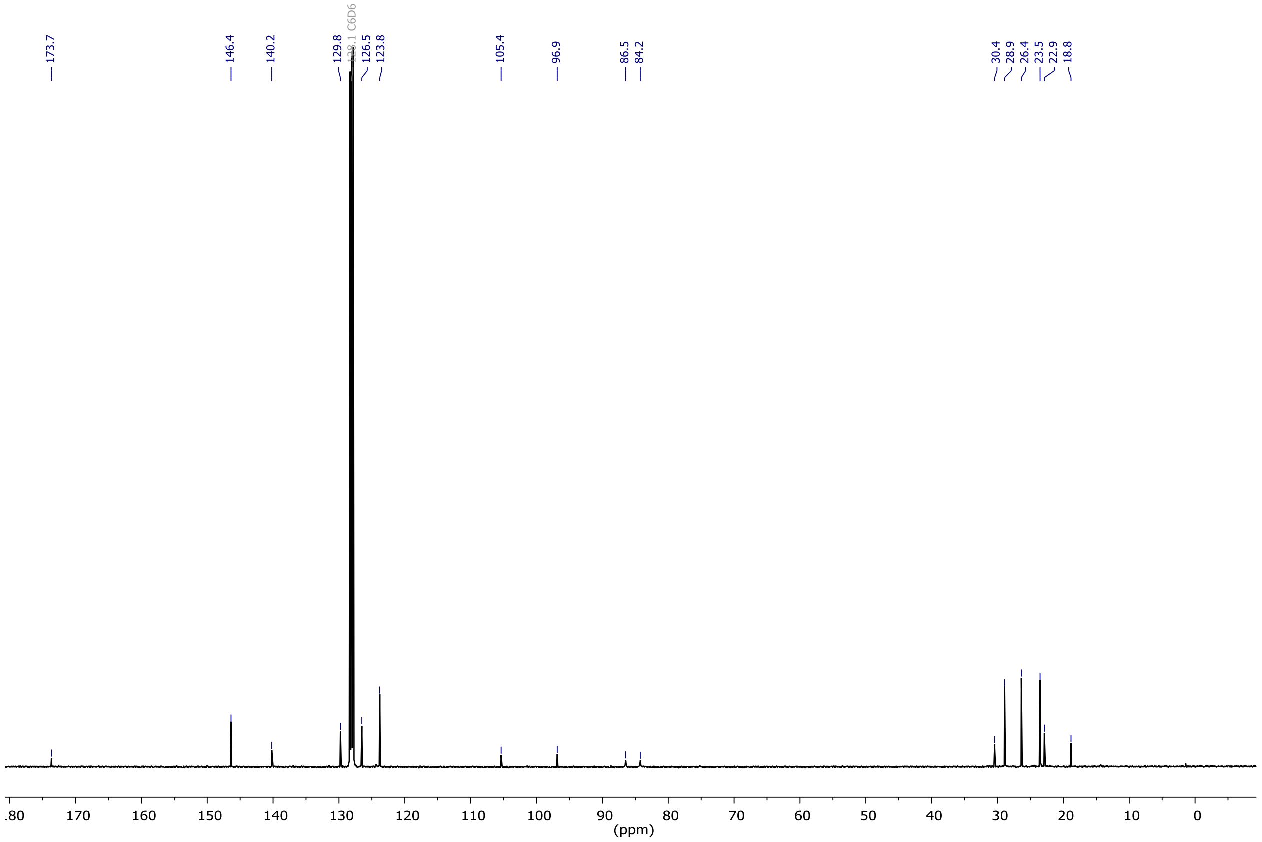
173.7
146.4
140.2
129.8
128.1 C6D6
126.5
123.8
105.4
96.9
86.5
84.2
30.4
28.9
26.4
23.5
22.9
18.8
(ppm)

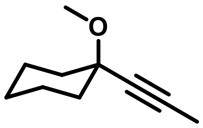

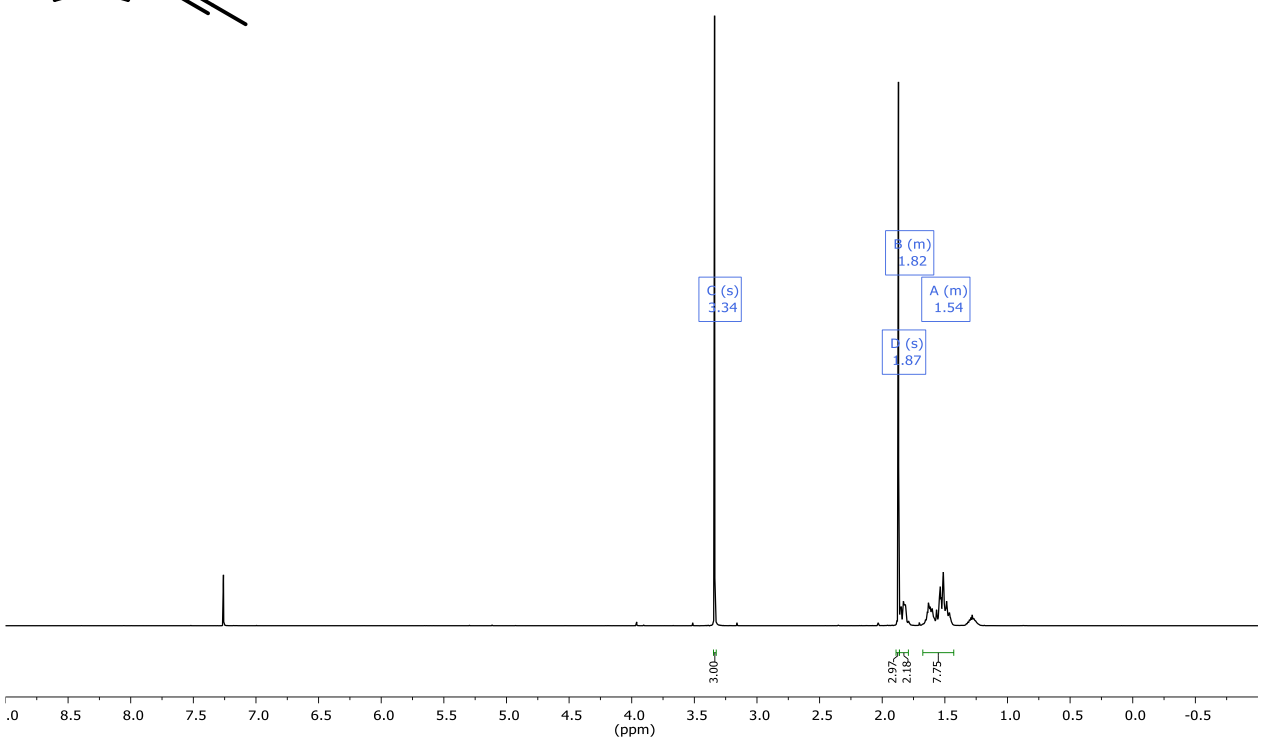
B (m)
1.82
C (s)
3.34
A (m)
1.54
D (s)
1.87
3.00
2.97
2.18
7.75
(ppm)

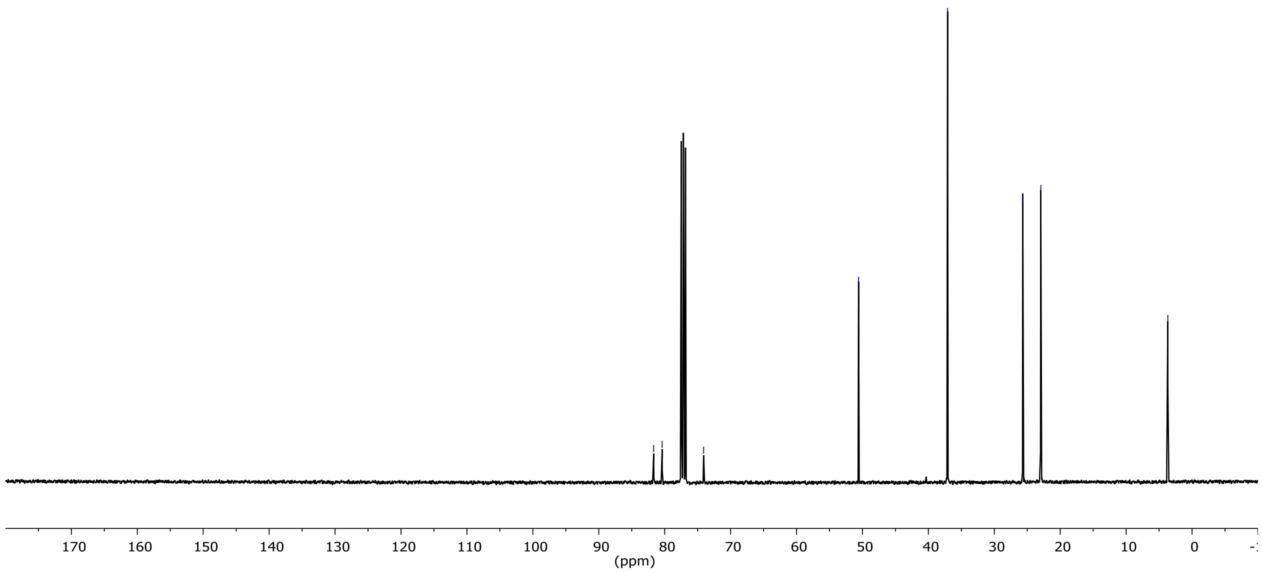
81.7
80.4
74.1
50.6
37.1
25.7
22.9
3.7
(ppm)

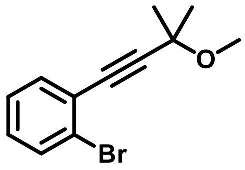
Br
O

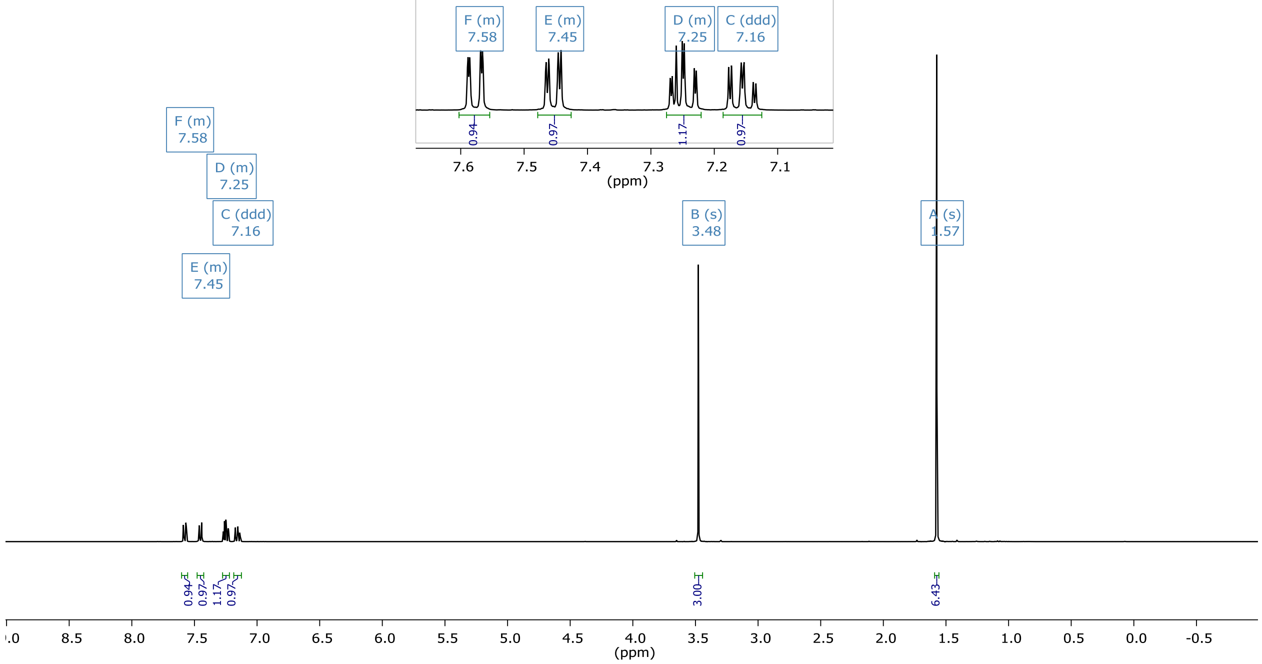
F (m) 7.58
E (m) 7.45
D (m) 7.25
C (ddd) 7.16
B (s) 3.48
A (s) 1.57
0.94
0.97
1.17
0.97
3.00
6.43
(ppm)

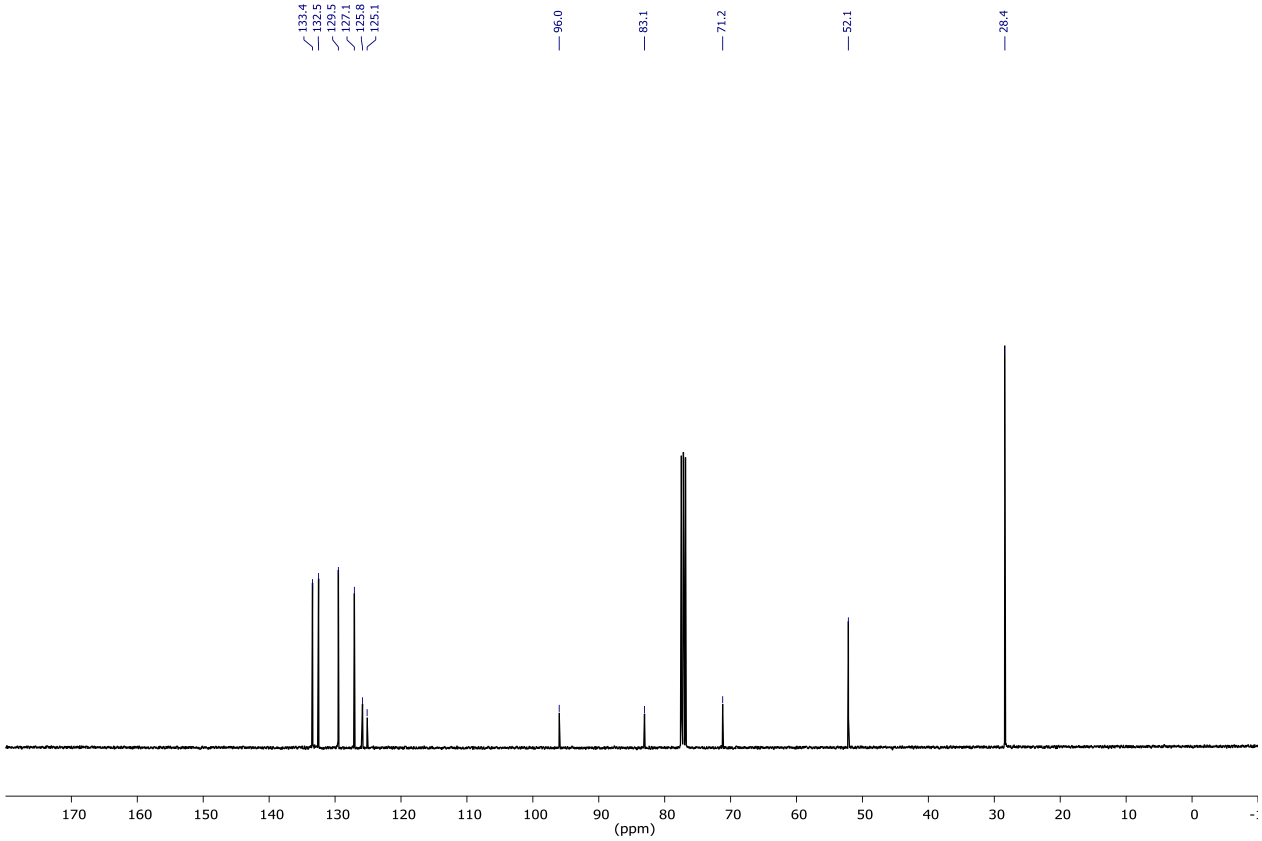
133.4
132.5
129.5
127.1
125.8
125.1
96.0
83.1
71.2
52.1
28.4
(ppm)

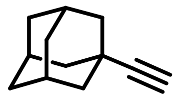

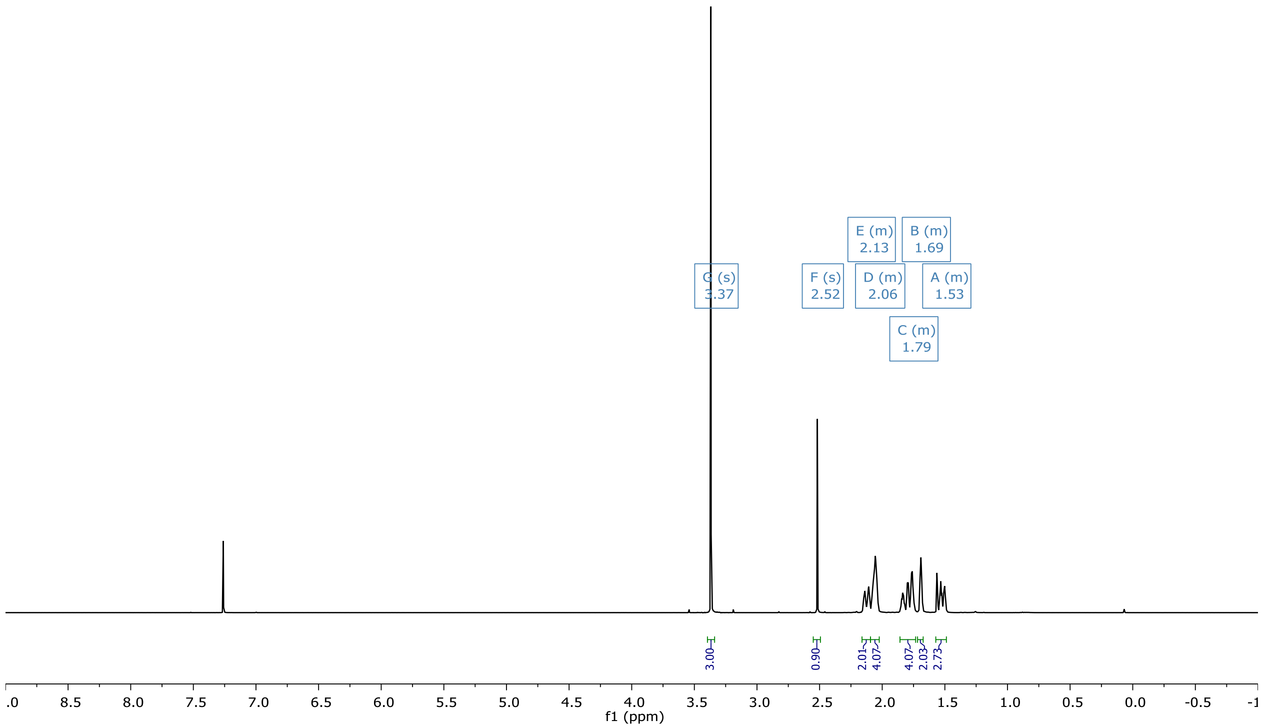
G (s)
3.37
F (s)
2.52
E (m)
2.13
D (m)
2.06
C (m)
1.79
B (m)
1.69
A (m)
1.53
3.00
0.90
2.01
4.07
4.07
2.03
2.73
f1 (ppm)

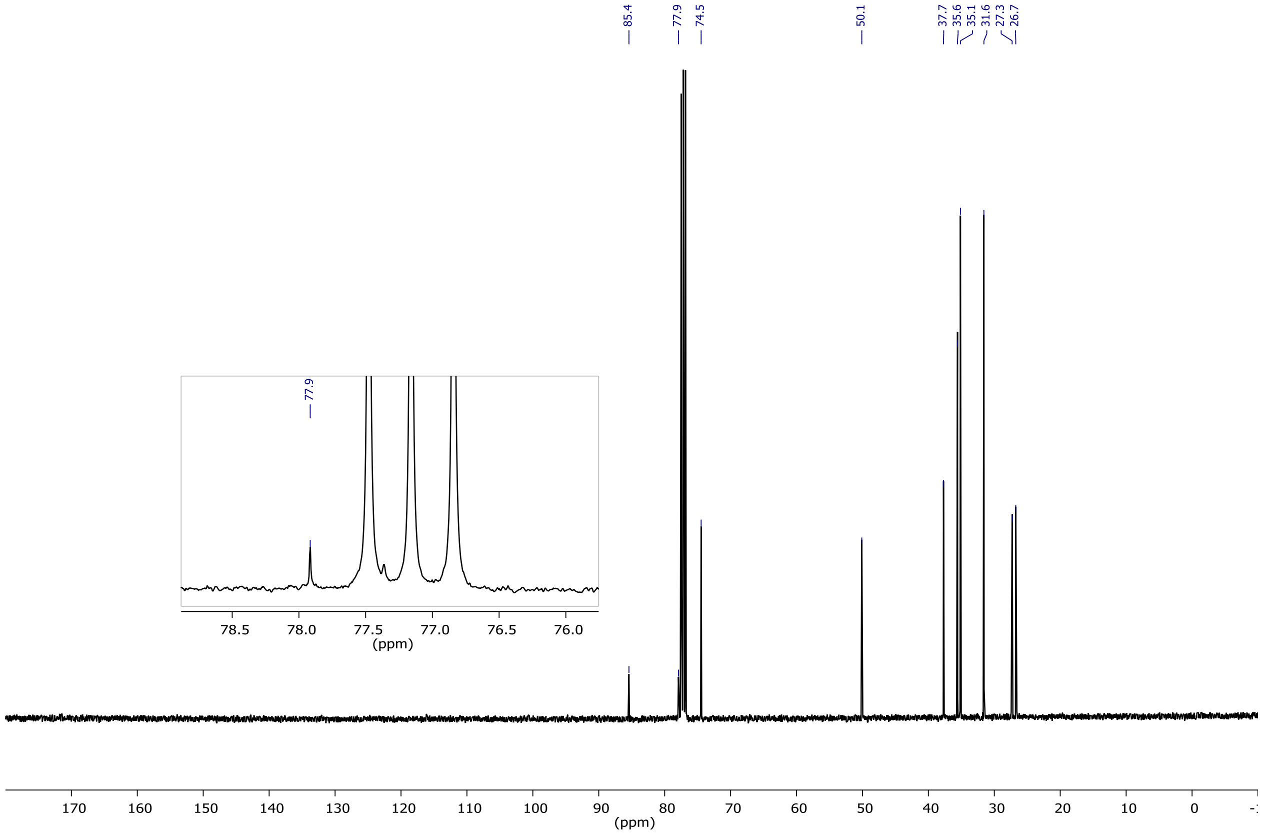
85.4
77.9
74.5
50.1
37.7
35.6
35.1
31.6
27.3
26.7
77.9
(ppm)
(ppm)

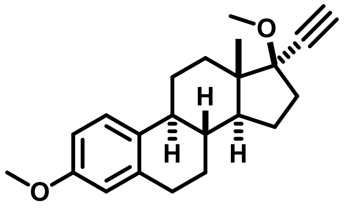

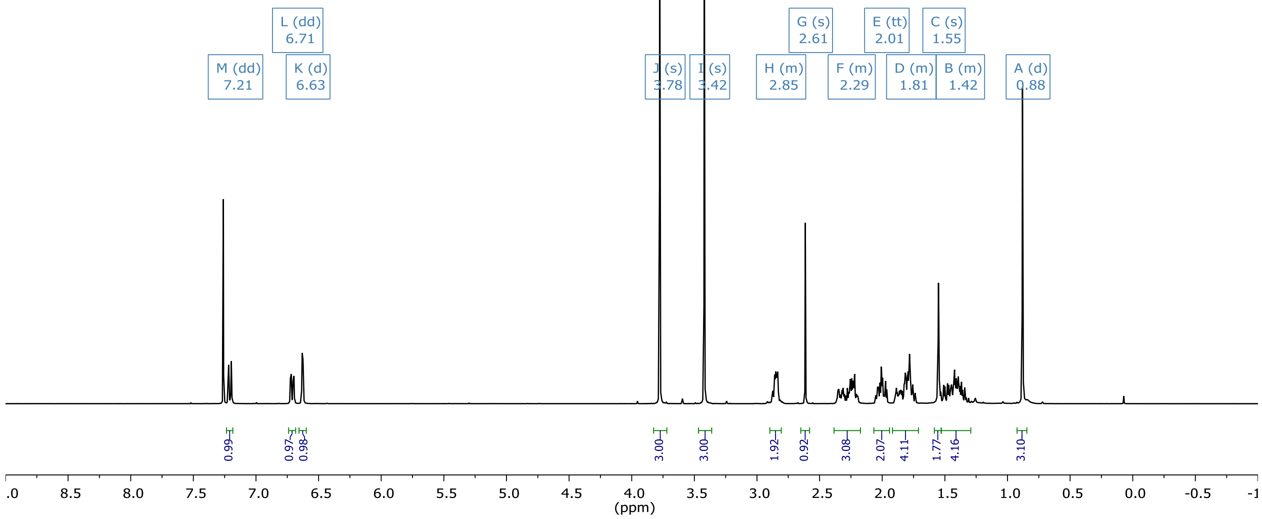

L (dd) 6.71
G (s) 2.61
E (tt) 2.01
C (s) 1.55
M (dd) 7.21
K (d) 6.63
J (s) 3.78
I (s) 3.42
H (m) 2.85
F (m) 2.29
D (m) 1.81
B (m) 1.42
A (d) 0.88
0.99
0.97
0.98
3.00
3.00
1.92
0.92
3.08
2.07
4.11
1.77
4.16
3.10
(ppm)

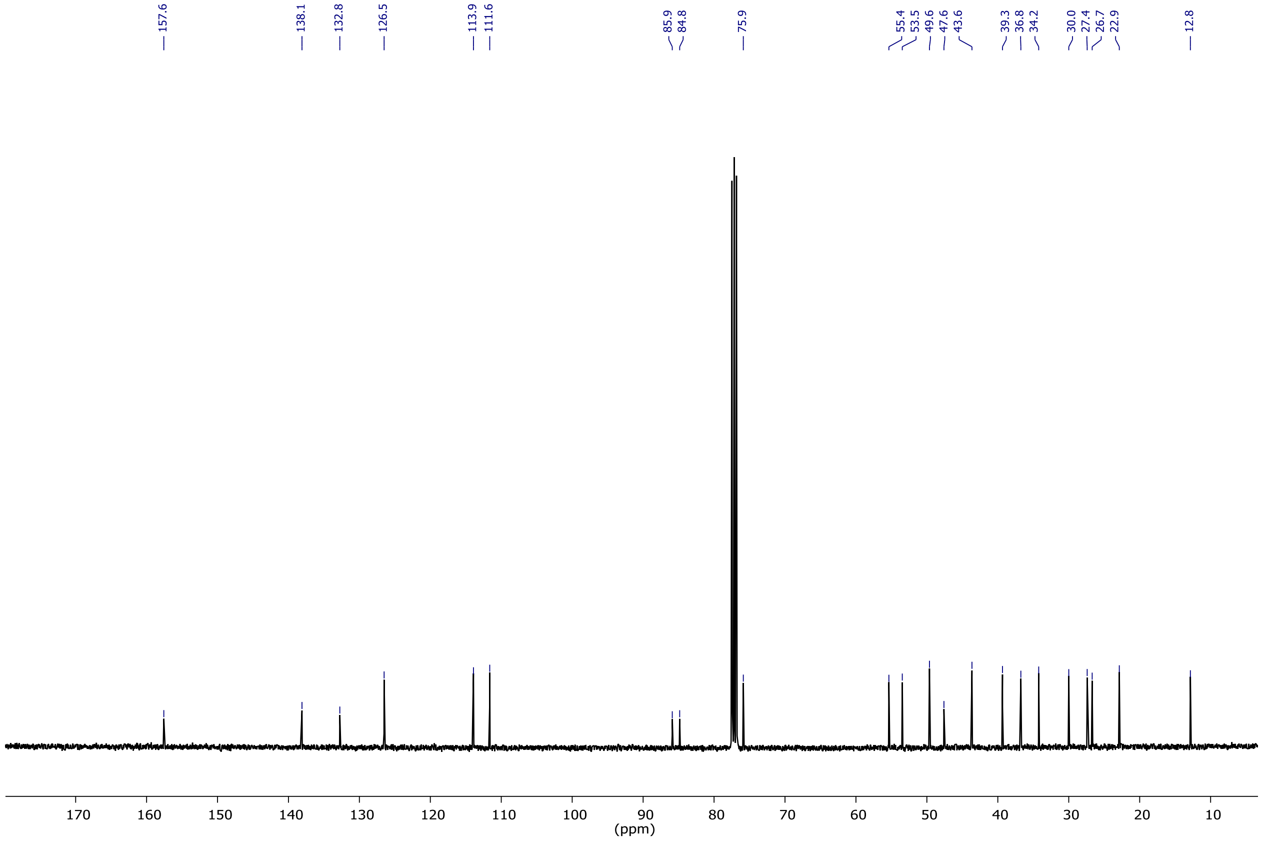

157.6
138.1
132.8
126.5
113.9
111.6
85.9
84.8
75.9
55.4
53.5
49.6
47.6
43.6
39.3
36.8
34.2
30.0
27.4
26.7
22.9
12.8
(ppm)

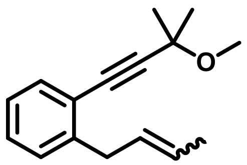

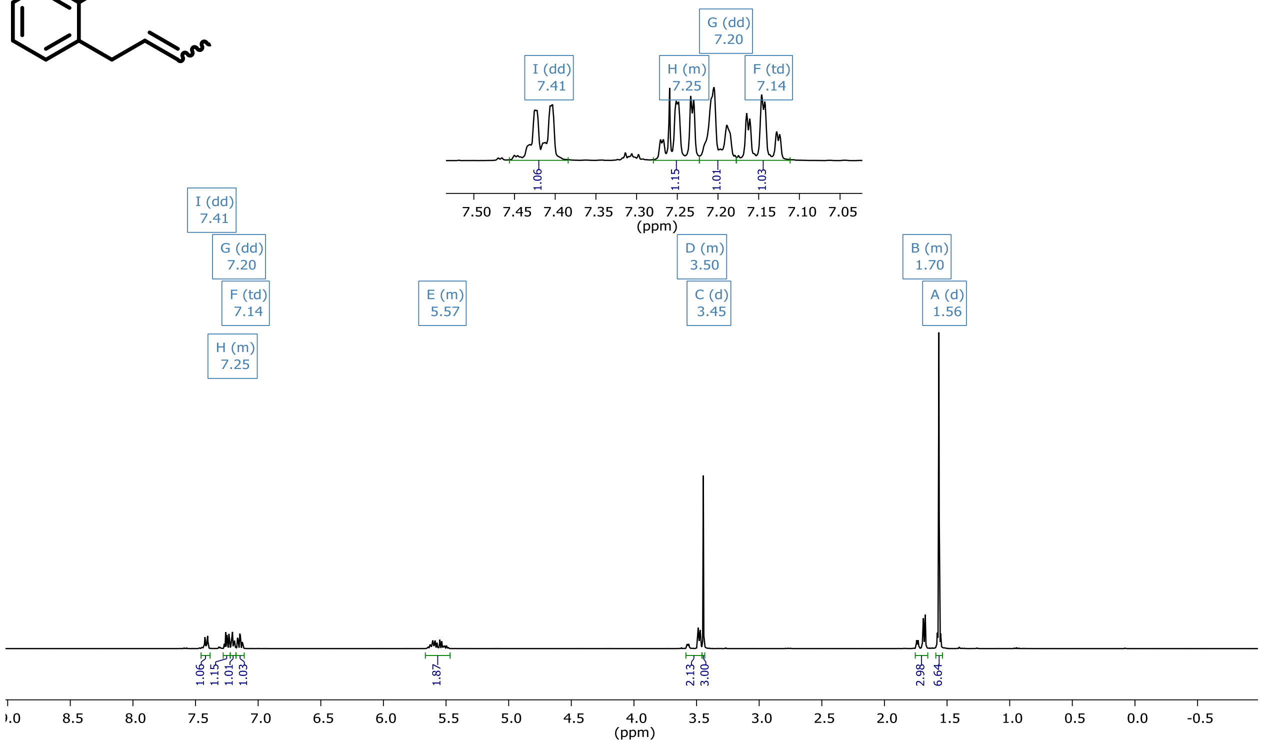
I (dd)
7.41
G (dd)
7.20
H (m)
7.25
F (td)
7.14
E (m)
5.57
D (m)
3.50
C (d)
3.45
B (m)
1.70
A (d)
1.56
1.06
1.15
1.01
1.03
1.87
2.13
3.00
2.98
6.64
(ppm)

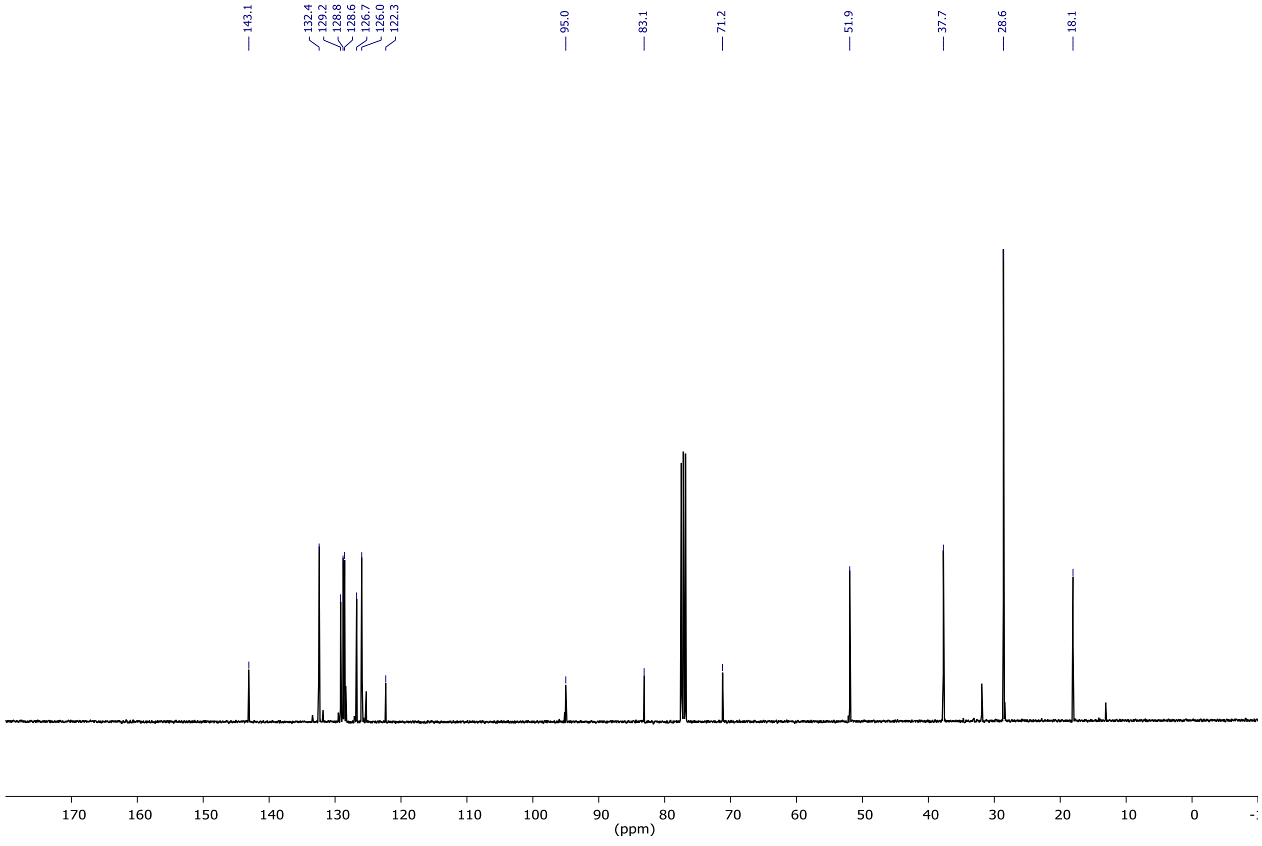
143.1
132.4
129.2
128.8
128.6
126.7
126.0
122.3
95.0
83.1
71.2
51.9
37.7
28.6
18.1
(ppm)

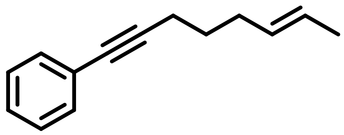

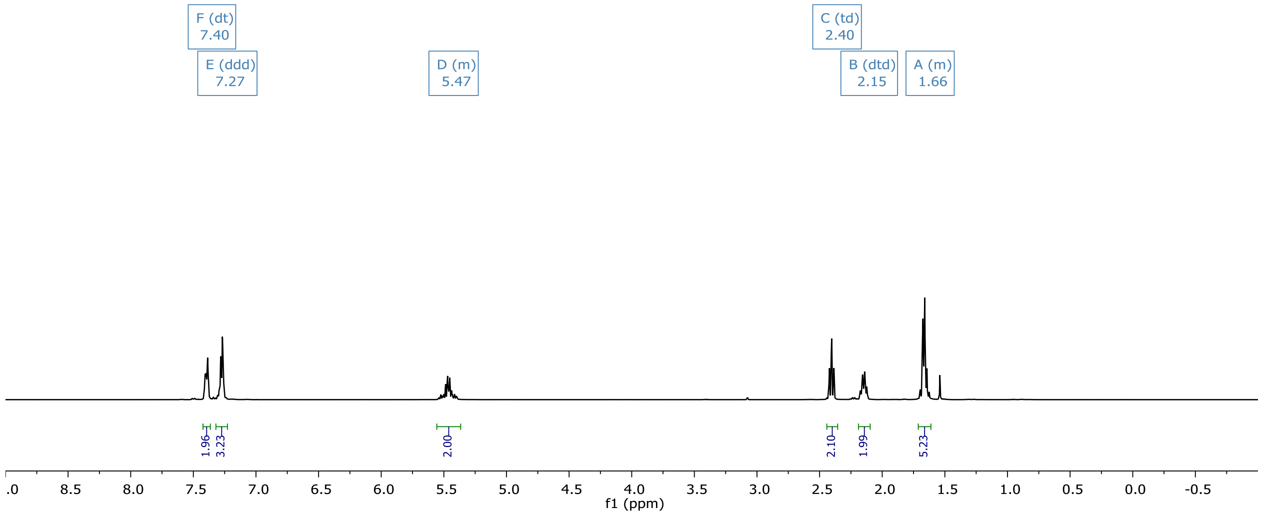
F (dt)
7.40
E (ddd)
7.27
D (m)
5.47
C (td)
2.40
B (dtd)
2.15
A (m)
1.66
1.96
3.23
2.00
2.10
1.99
5.23
f1 (ppm)

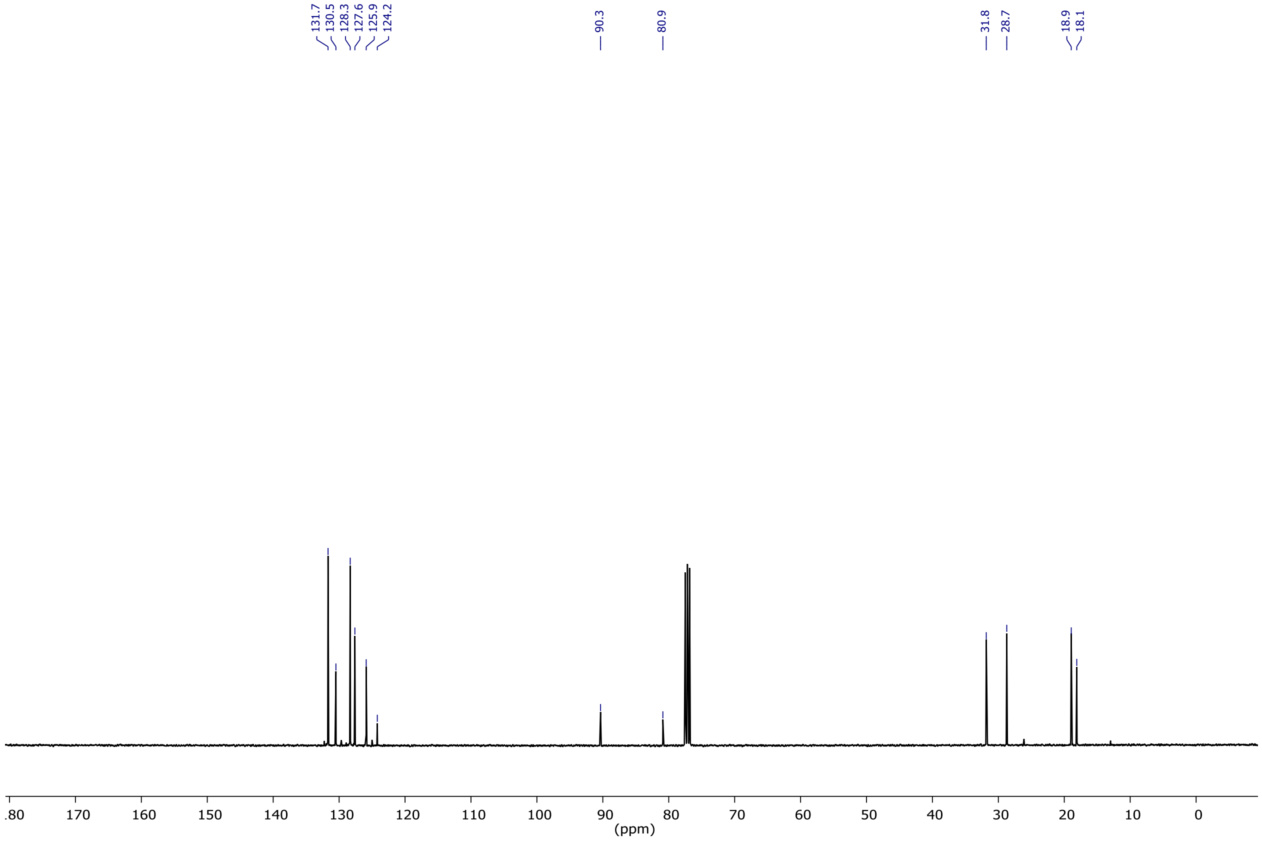
131.7
130.5
128.3
127.6
125.9
124.2
90.3
80.9
31.8
28.7
18.9
18.1
(ppm)

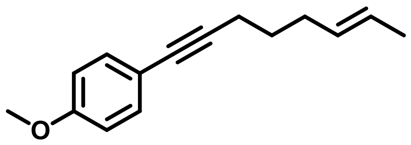

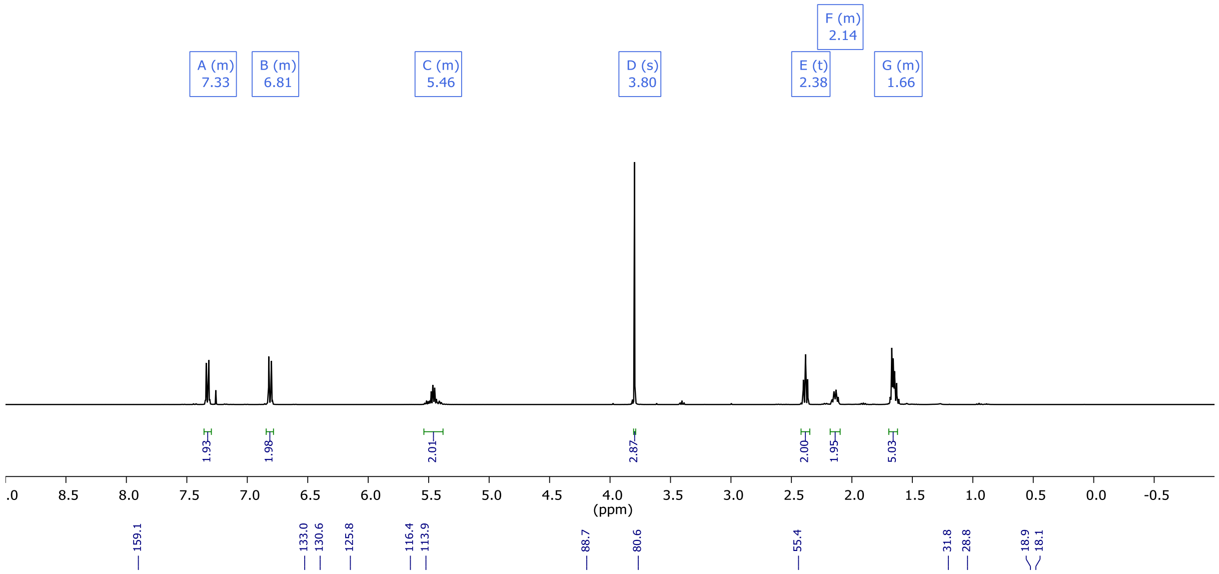
A (m)
7.33
B (m)
6.81
C (m)
5.46
D (s)
3.80
E (t)
2.38
F (m)
2.14
G (m)
1.66
1.93
1.98
2.01
2.87
2.00
1.95
5.03
(ppm)

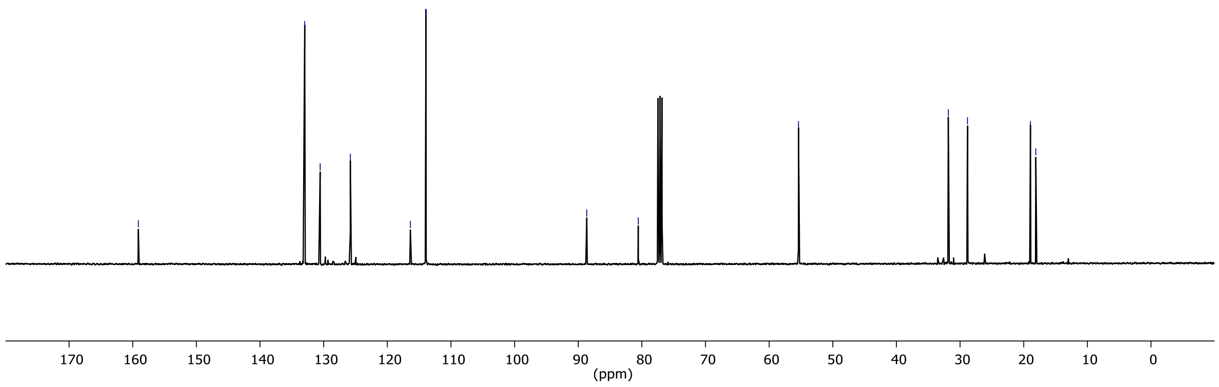
159.1
133.0
130.6
125.8
116.4
113.9
88.7
80.6
55.4
31.8
28.8
18.9
18.1
(ppm)

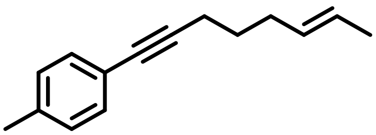

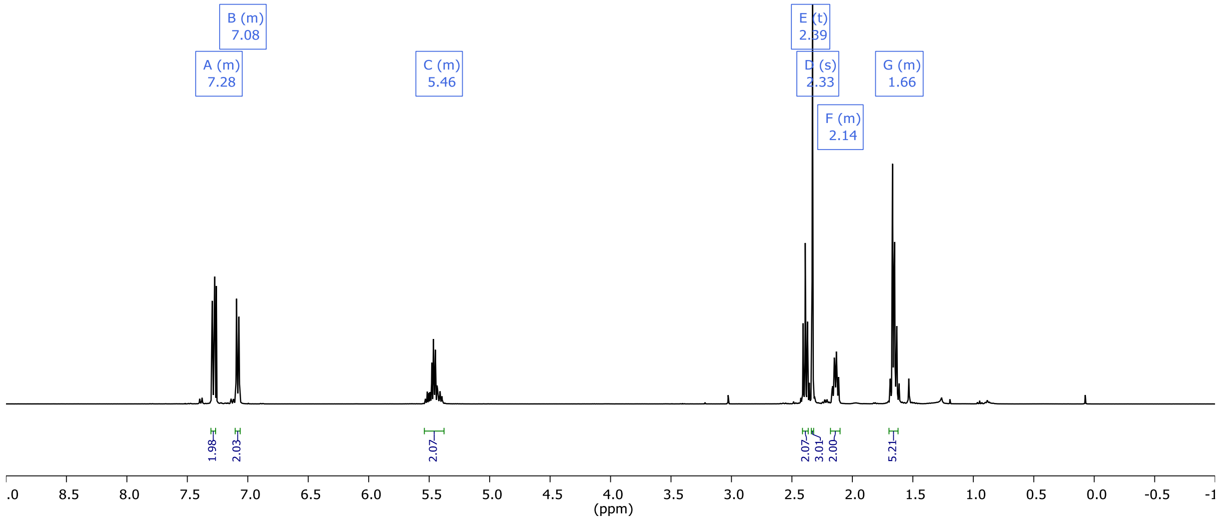
B (m)
7.08
A (m)
7.28
C (m)
5.46
E (t)
2.39
D (s)
2.33
G (m)
1.66
F (m)
2.14
1.98
2.03
2.07
2.07
3.01
2.00
5.21
(ppm)

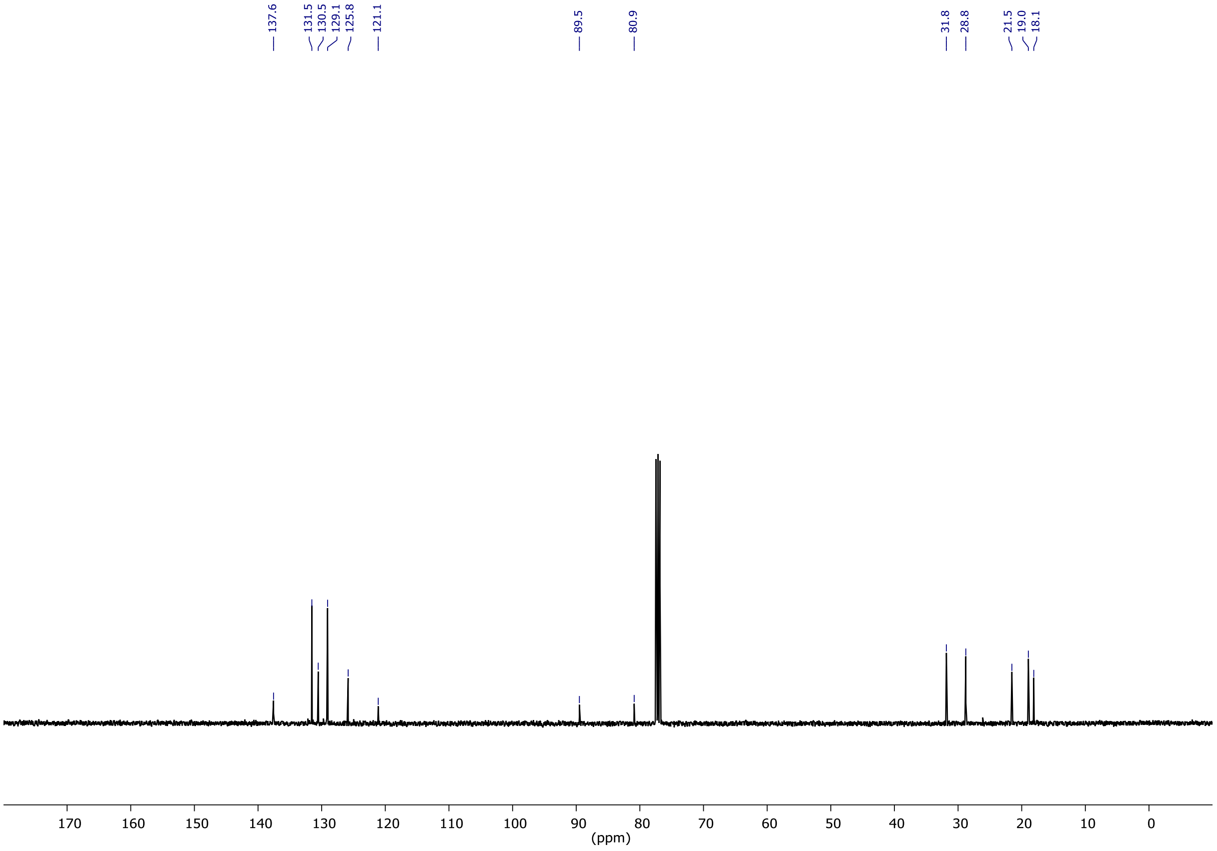
137.6
131.5
130.5
129.1
125.8
121.1
89.5
80.9
31.8
28.8
21.5
19.0
18.1
(ppm)

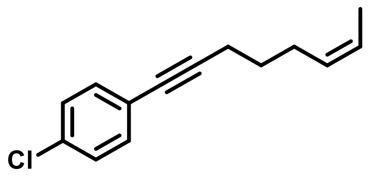
Cl

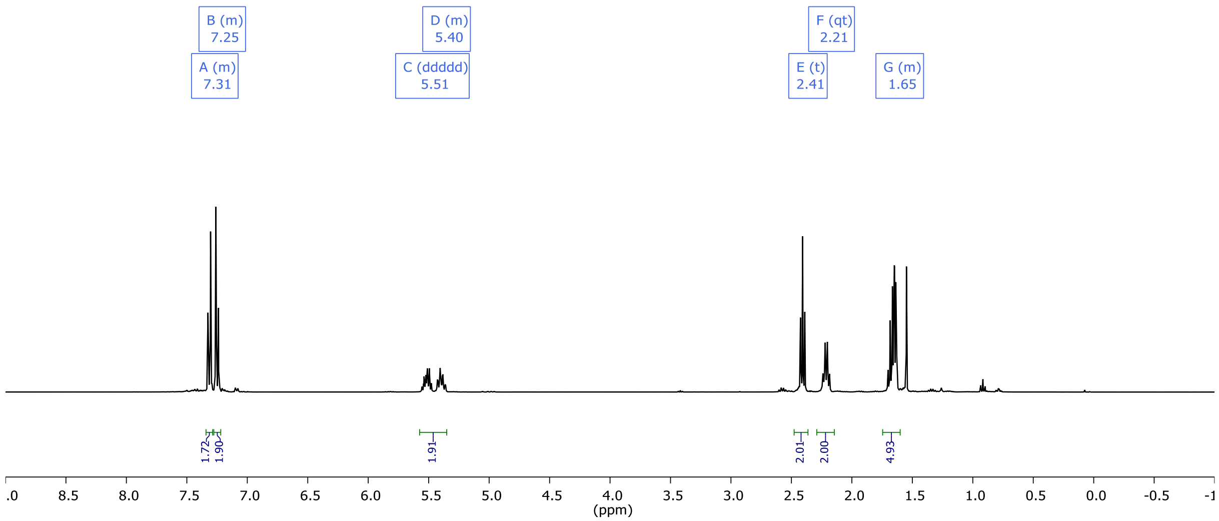
B (m)
7.25
A (m)
7.31
D (m)
5.40
C (ddddd)
5.51
F (qt)
2.21
E (t)
2.41
G (m)
1.65
1.72
1.90
1.91
2.01
2.00
4.93
(ppm)

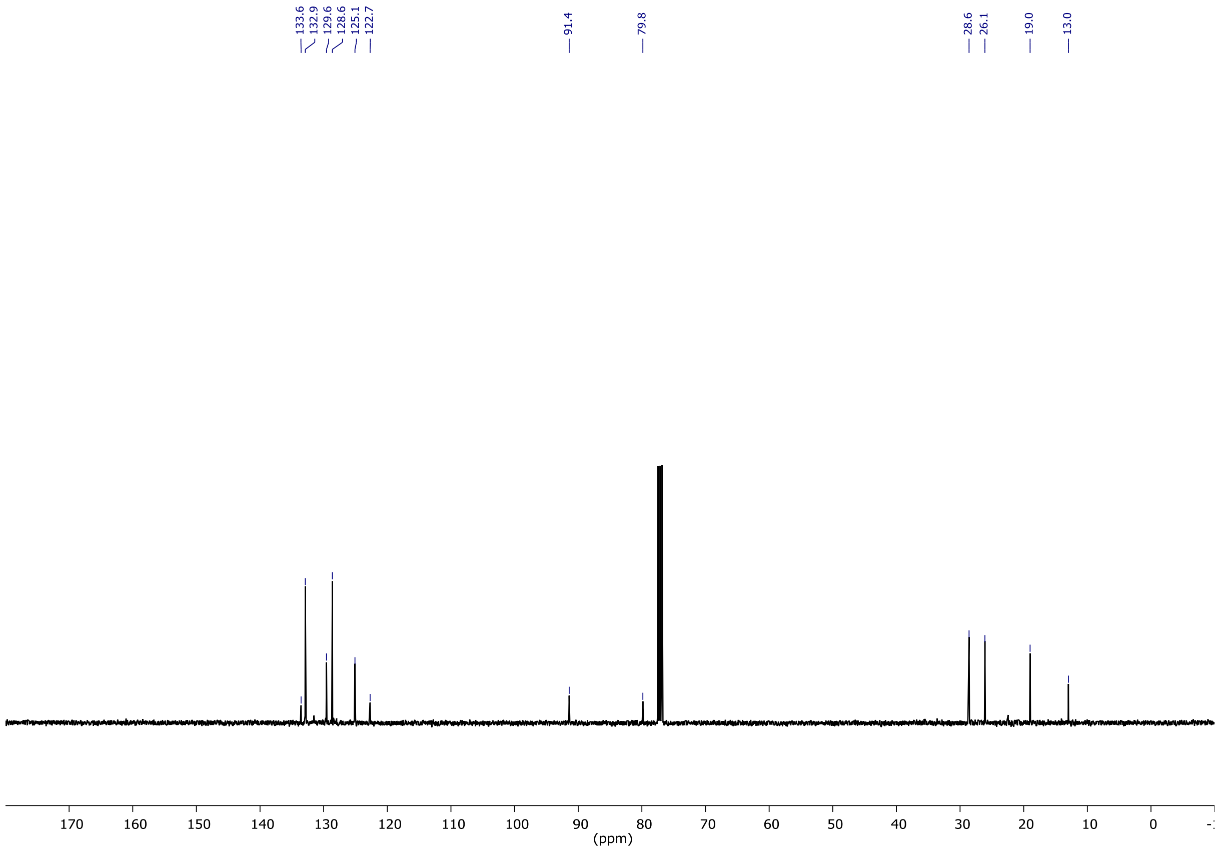
133.6
132.9
129.6
128.6
125.1
122.7
91.4
79.8
28.6
26.1
19.0
13.0
(ppm)

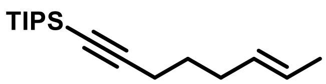
TIPS

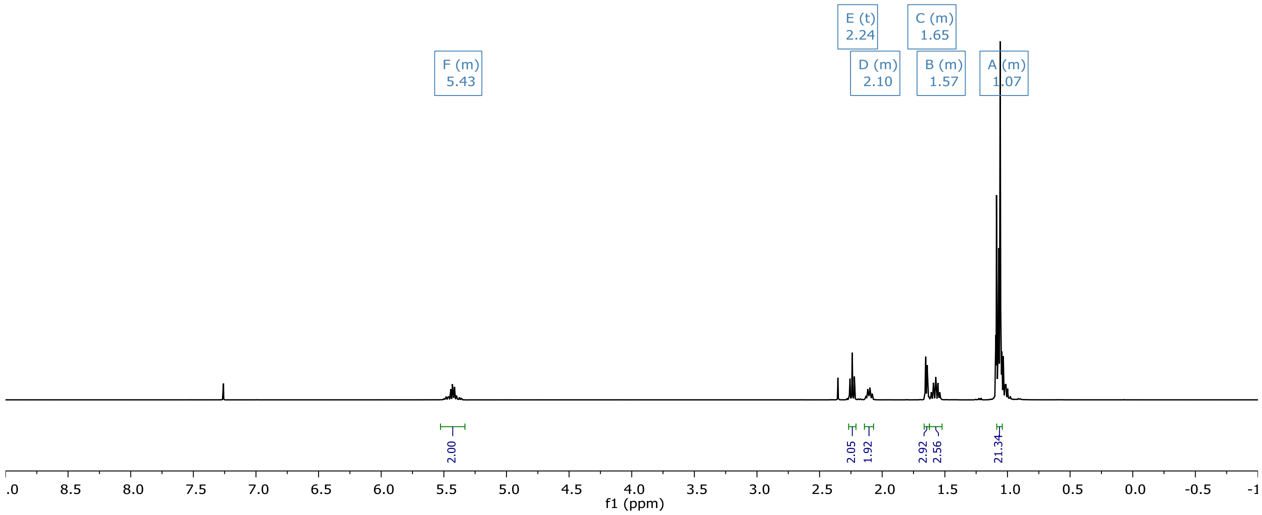
F (m)
5.43
E (t)
2.24
D (m)
2.10
C (m)
1.65
B (m)
1.57
A (m)
1.07
2.00
2.05
1.92
2.92
2.56
21.34
f1 (ppm)

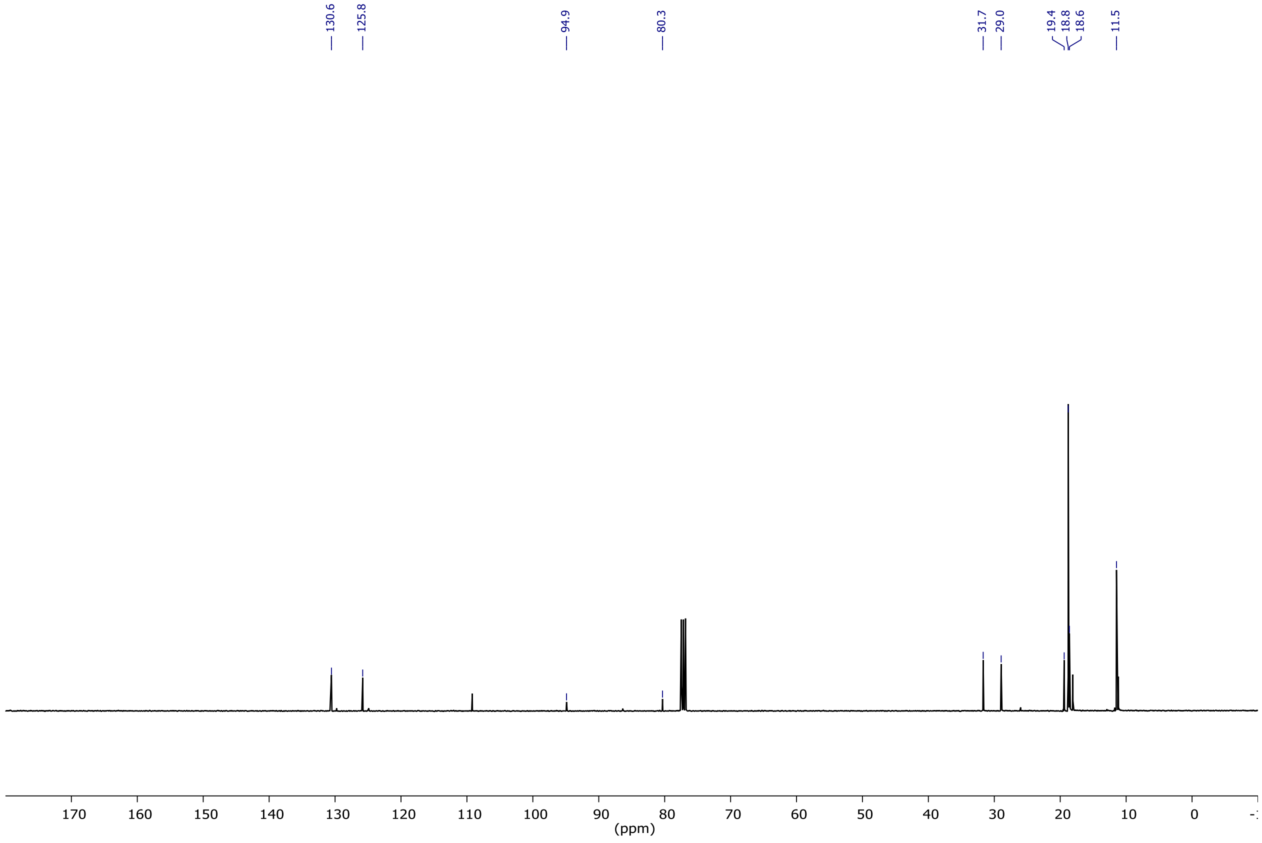
130.6
125.8
94.9
80.3
31.7
29.0
19.4
18.8
18.6
11.5
(ppm)

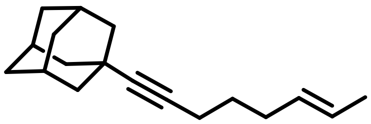

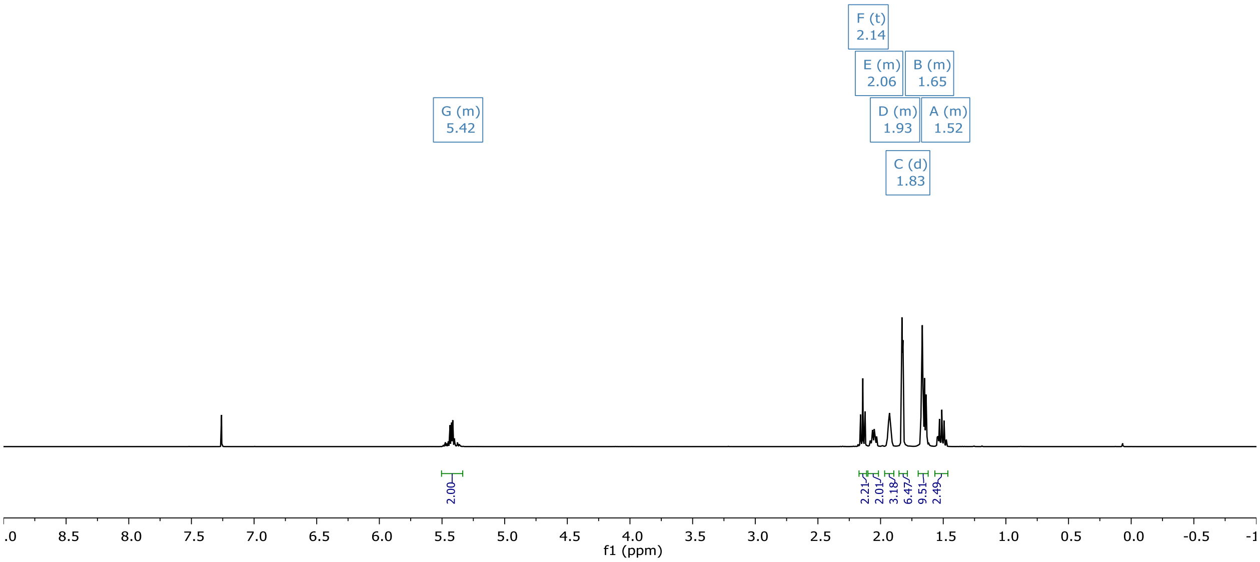
F (t)
2.14
E (m)
2.06
B (m)
1.65
G (m)
5.42
D (m)
1.93
A (m)
1.52
C (d)
1.83
2.00
2.21
2.01
3.18
6.47
9.51
2.49
f1 (ppm)

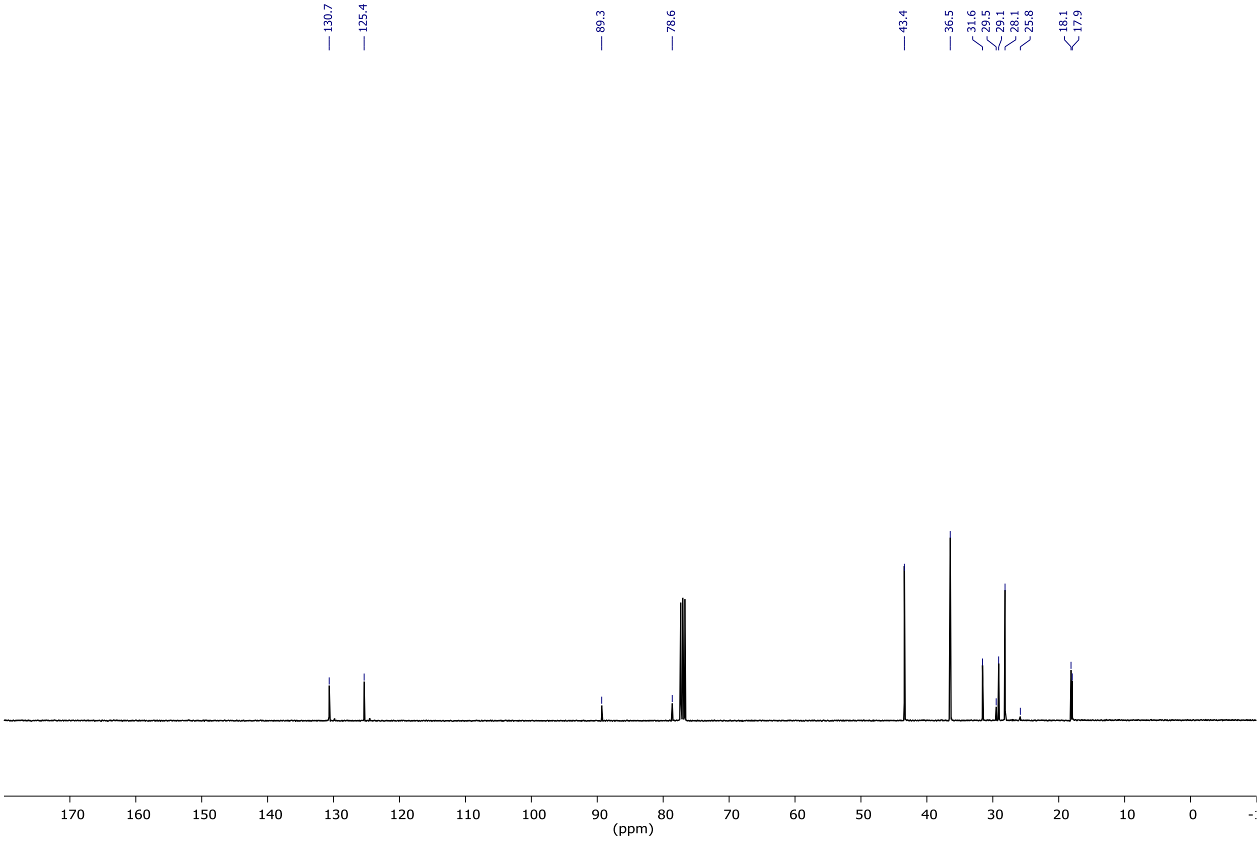
130.7
125.4
89.3
78.6
43.4
36.5
31.6
29.5
29.1
28.1
25.8
18.1
17.9
(ppm)

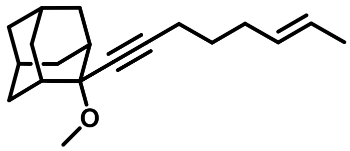

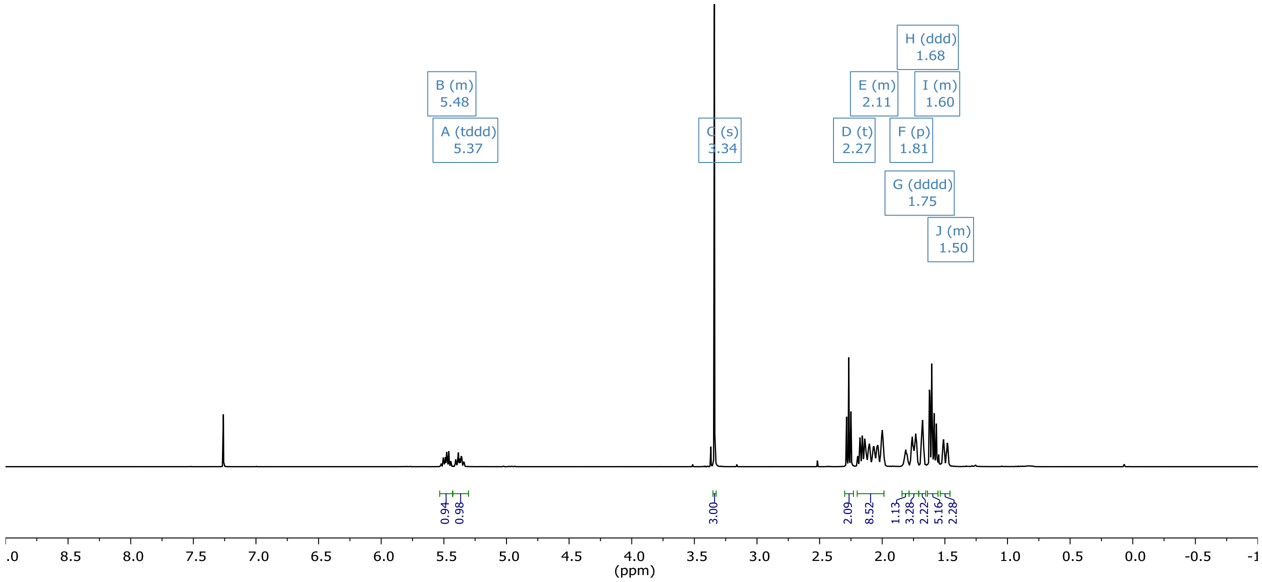

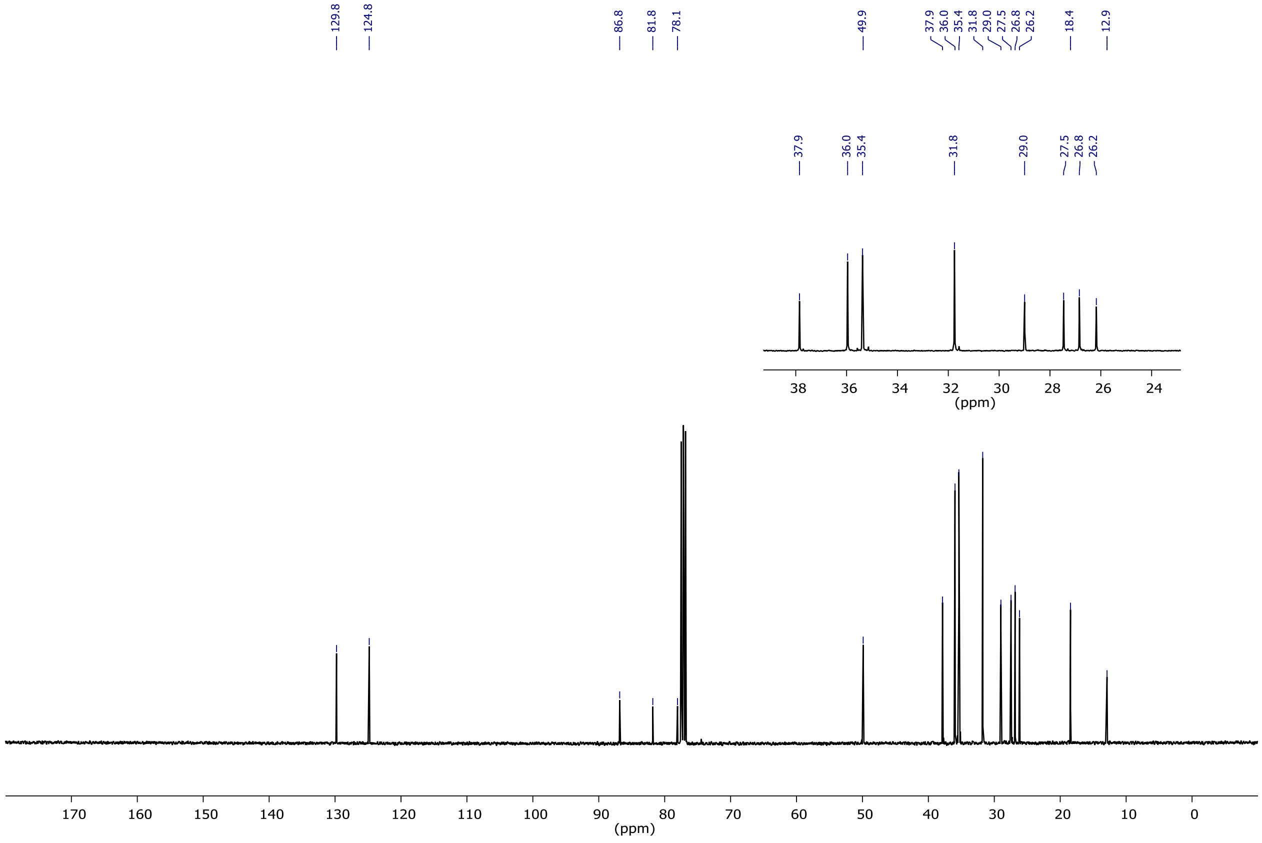

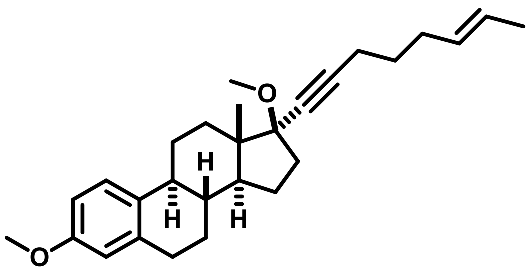

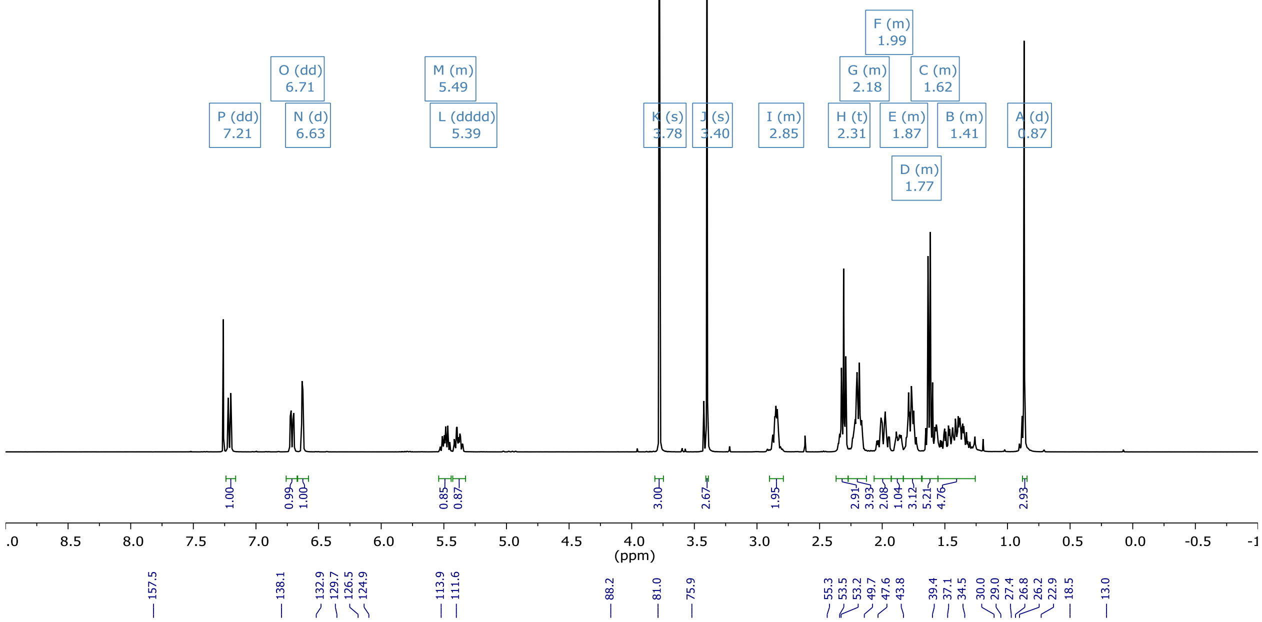
P (dd) 7.21
O (dd) 6.71
N (d) 6.63
M (m) 5.49
L (dddd) 5.39
K (s) 3.78
J (s) 3.40
I (m) 2.85
H (t) 2.31
G (m) 2.18
F (m) 1.99
E (m) 1.87
D (m) 1.77
C (m) 1.62
B (m) 1.41
A (d) 0.87
1.00 0.99 1.00 0.85 0.87 3.00 2.67 1.95 2.91 3.93 2.08 1.04 3.12 5.21 4.76 2.93
(ppm)

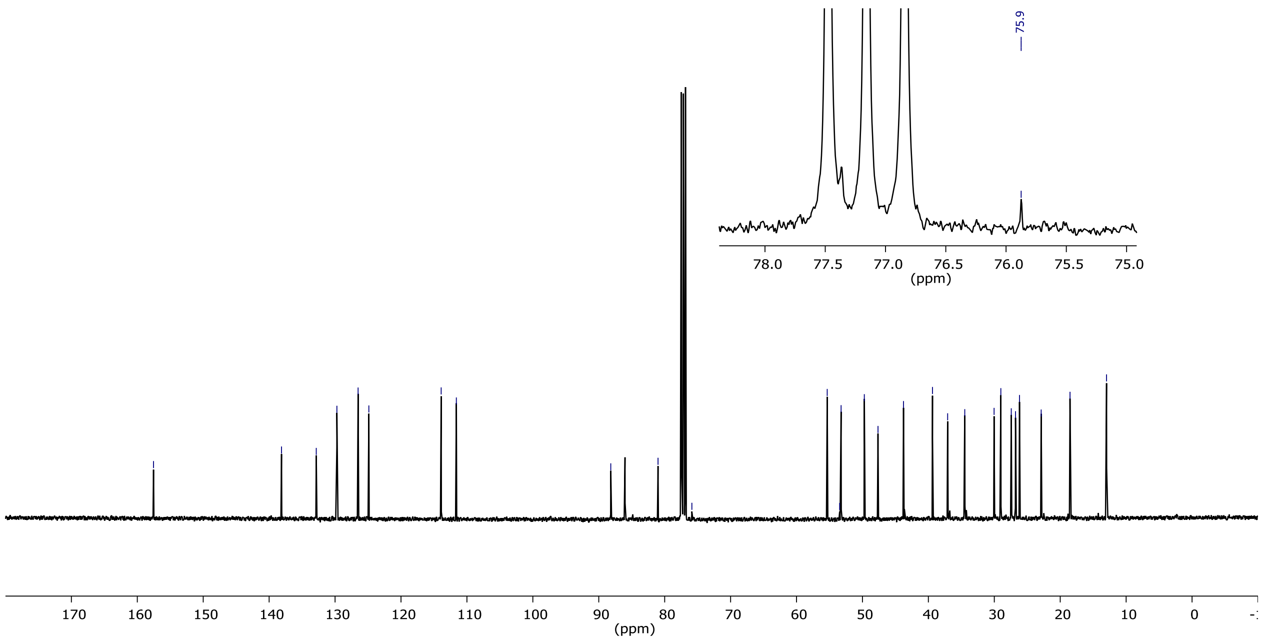
157.5 138.1 132.9 129.7 126.5 124.9 113.9 111.6 88.2 81.0 75.9 55.3 53.5 53.2 49.7 47.6 43.8 39.4 37.1 34.5 30.0 29.0 27.4 26.8 26.2 22.9 18.5 13.0
75.9
(ppm)
(ppm)

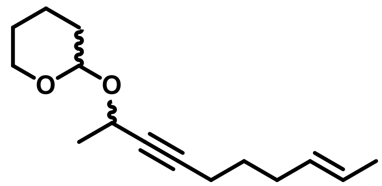

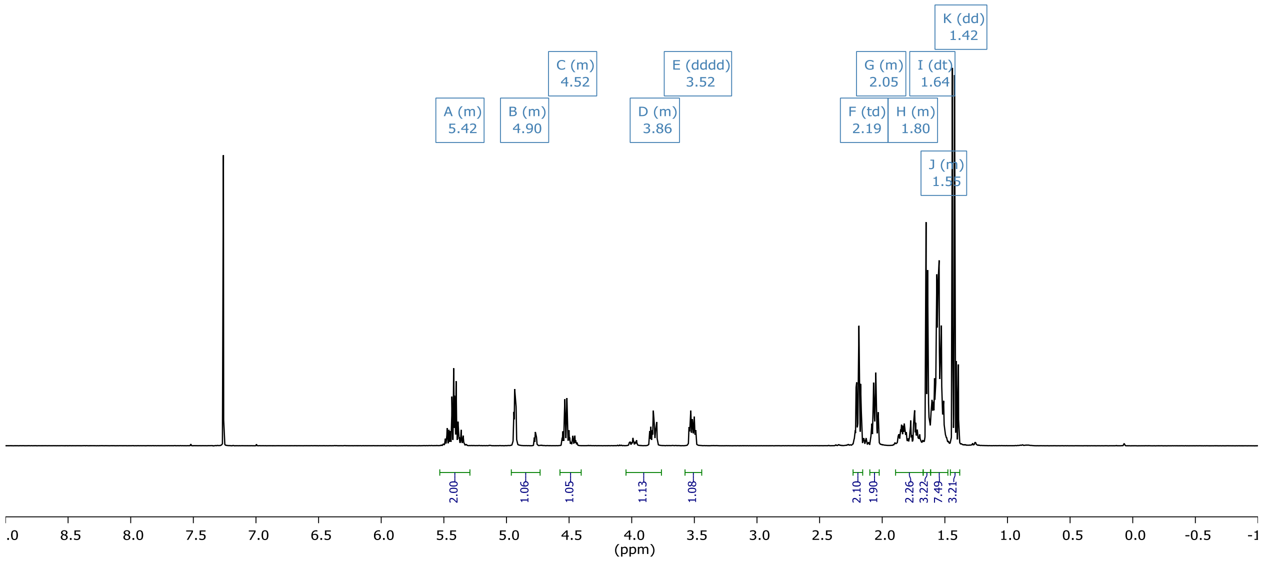
A (m) 5.42
B (m) 4.90
C (m) 4.52
D (m) 3.86
E (dddd) 3.52
F (td) 2.19
G (m) 2.05
H (m) 1.80
I (dt) 1.64
J (m) 1.56
K (dd) 1.42
2.00 1.06 1.05 1.13 1.08 2.10 1.90 2.26 3.22 7.49 3.21
(ppm)

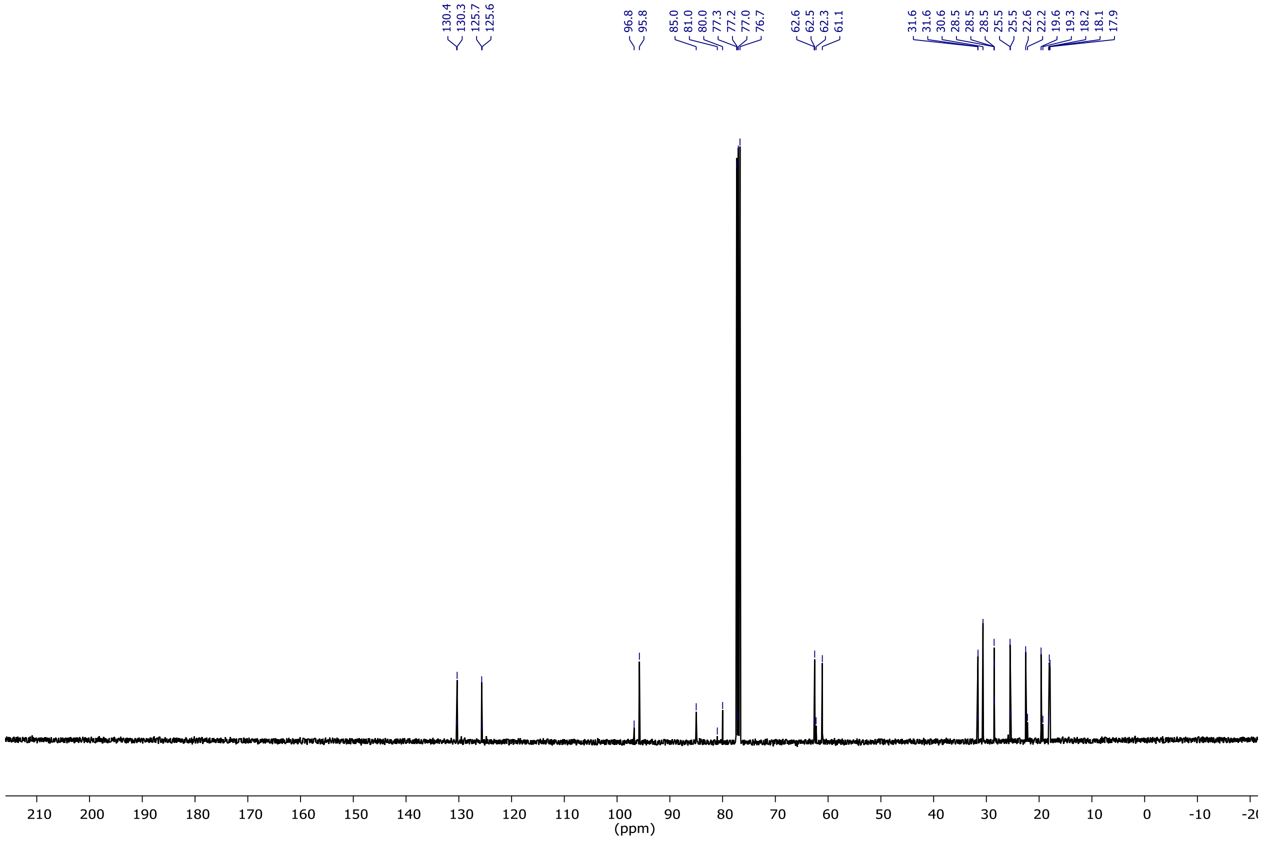
130.4 130.3 125.7 125.6
96.8 95.8
85.0 81.0 80.0 77.3 77.2 77.0 76.7
62.6 62.5 62.3 61.1
31.6 31.6 30.6 28.5 28.5 28.5 25.5 25.5 22.6 22.2 19.6 19.3 18.2 18.1 17.9
(ppm)

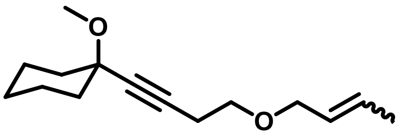
O
O

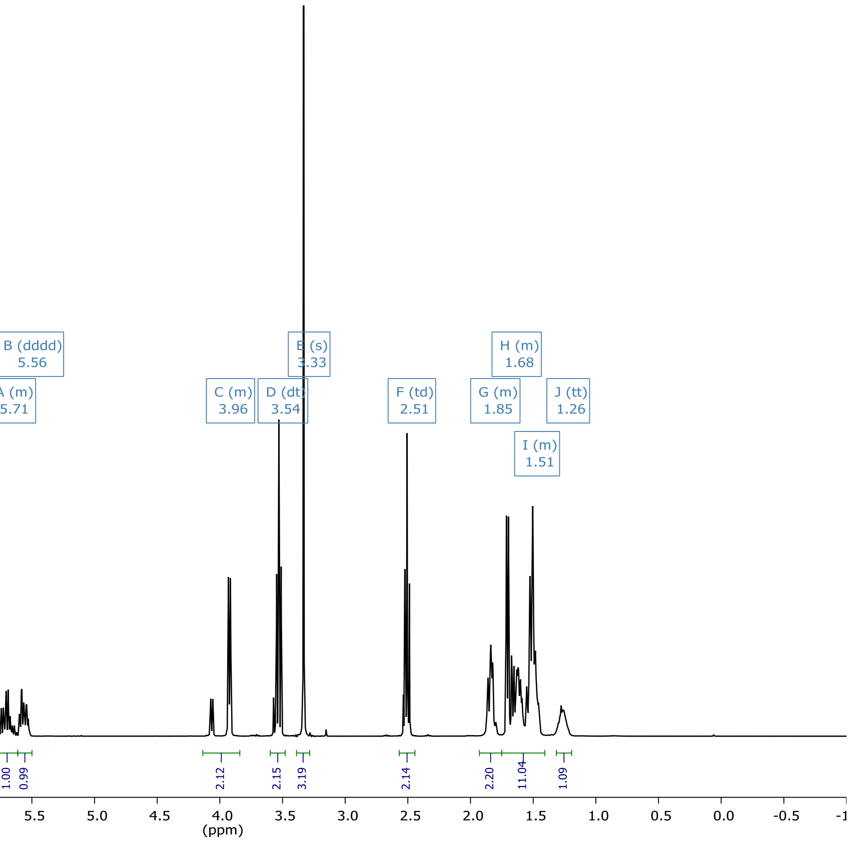
B (dddd) 5.56
A (m) 5.71
C (m) 3.96
D (dt) 3.54
E (s) 3.33
F (td) 2.51
G (m) 1.85
H (m) 1.68
J (tt) 1.26
I (m) 1.51
1.00
0.99
2.12
2.15
3.19
2.14
2.20
11.04
1.09
(ppm)

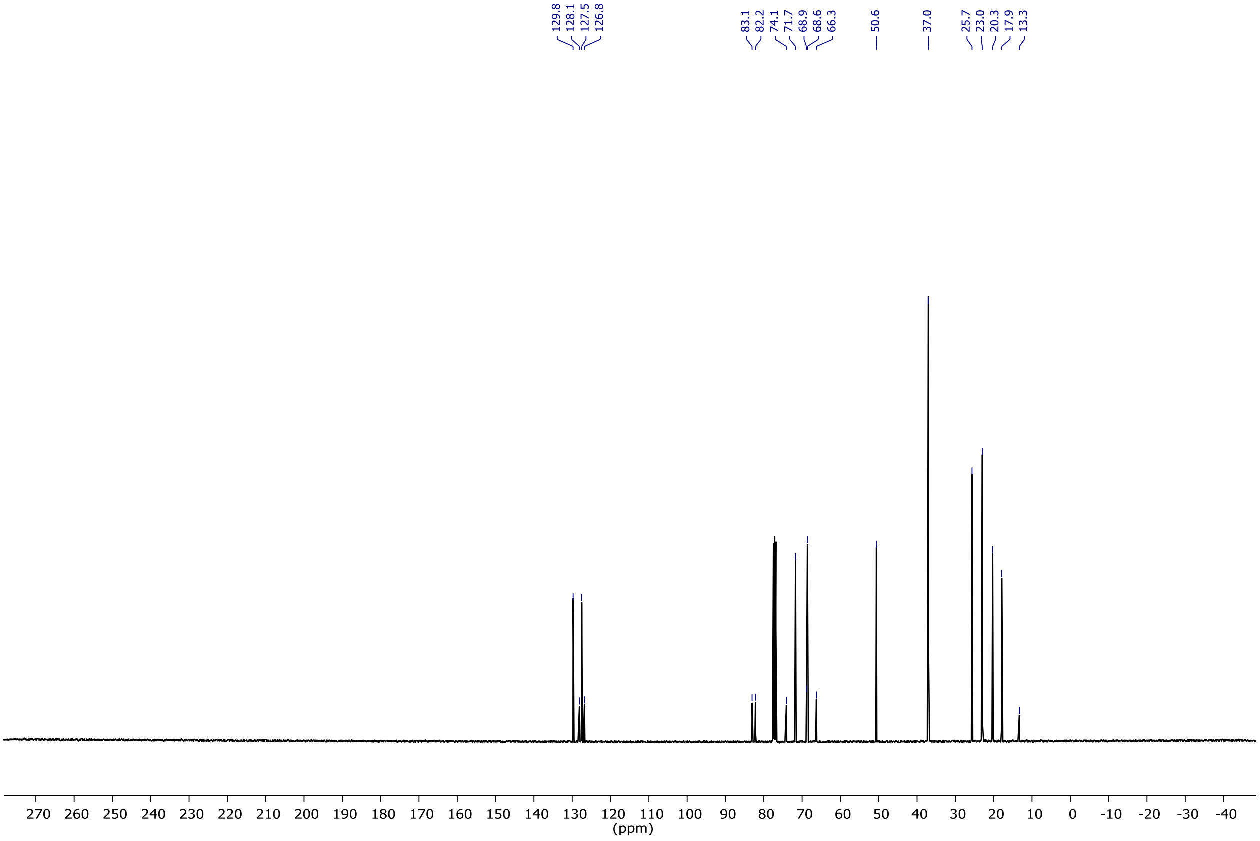
129.8
128.1
127.5
126.8
83.1
82.2
74.1
71.7
68.9
68.6
66.3
50.6
37.0
25.7
23.0
20.3
17.9
13.3
(ppm)

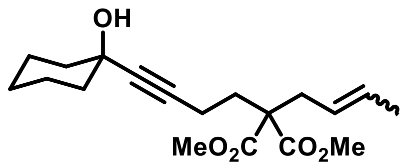
OH
MeO2C
CO2Me

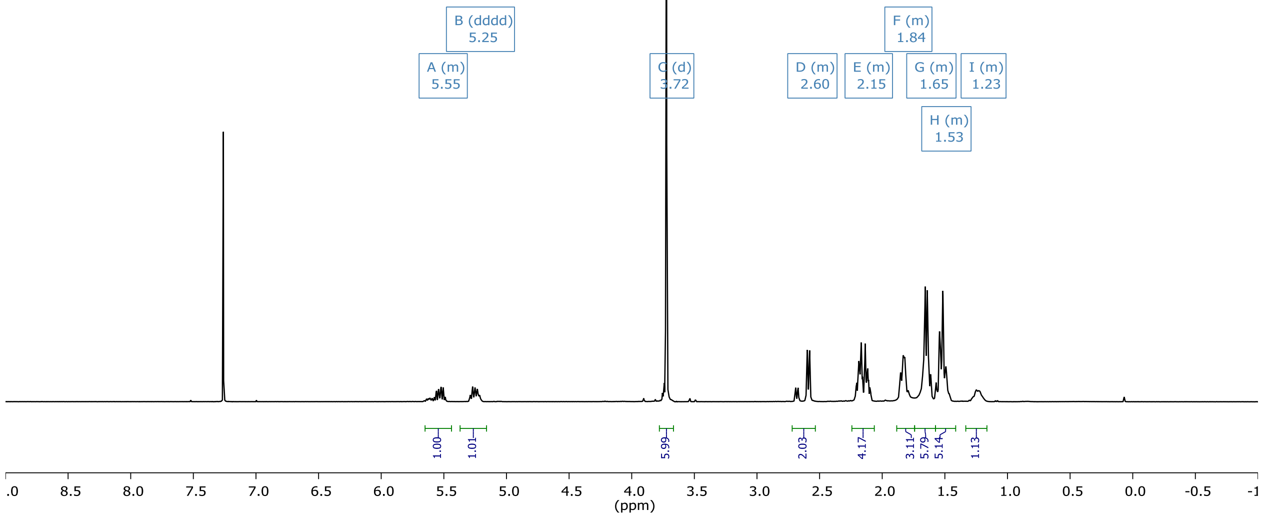
B (dddd)
5.25
A (m)
5.55
C (d)
3.72
D (m)
2.60
E (m)
2.15
F (m)
1.84
G (m)
1.65
I (m)
1.23
H (m)
1.53
1.00
1.01
5.99
2.03
4.17
3.11
5.79
5.14
1.13
(ppm)

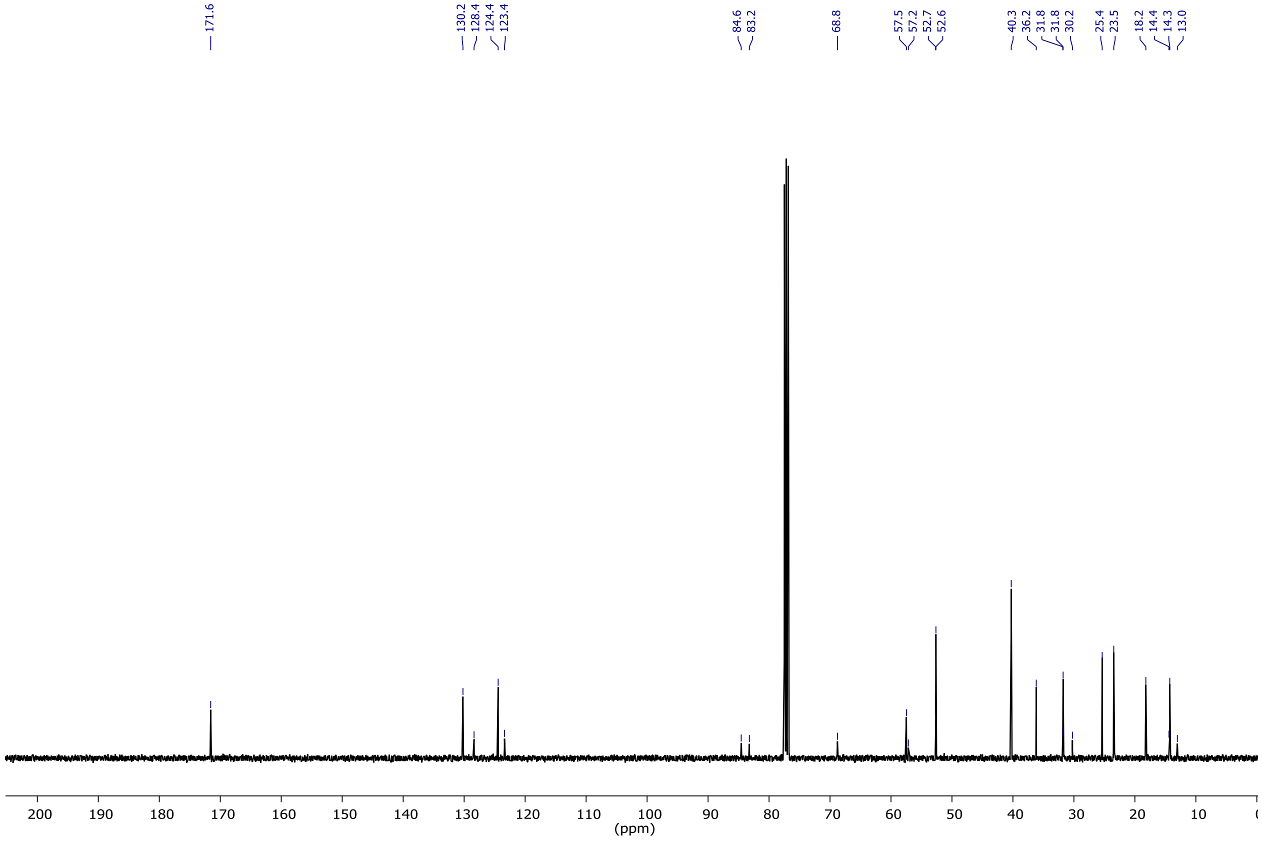
171.6
130.2
128.4
124.4
123.4
84.6
83.2
68.8
57.5
57.2
52.7
52.6
40.3
36.2
31.8
31.8
30.2
25.4
23.5
18.2
14.4
14.3
13.0
(ppm)

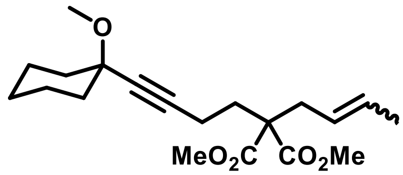
O
MeO2C CO2Me

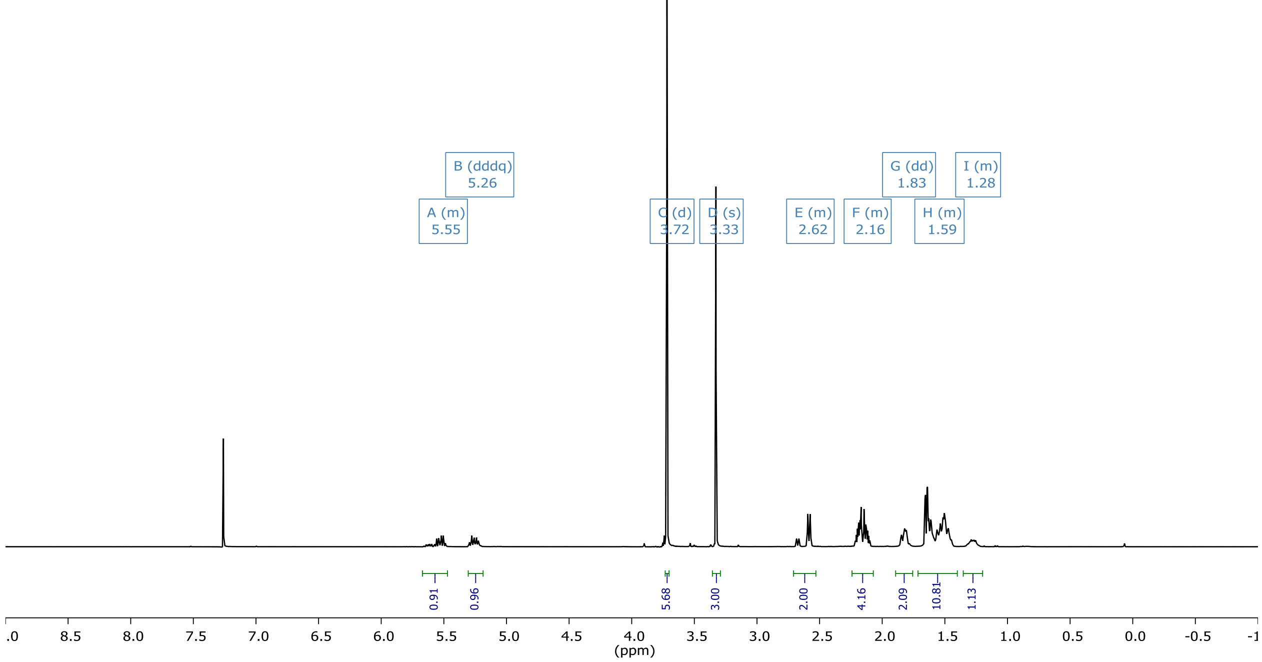
B (dddq)
5.26
A (m)
5.55
C (d)
3.72
D (s)
3.33
E (m)
2.62
F (m)
2.16
G (dd)
1.83
H (m)
1.59
I (m)
1.28
0.91
0.96
5.68
3.00
2.00
4.16
2.09
10.81
1.13
(ppm)

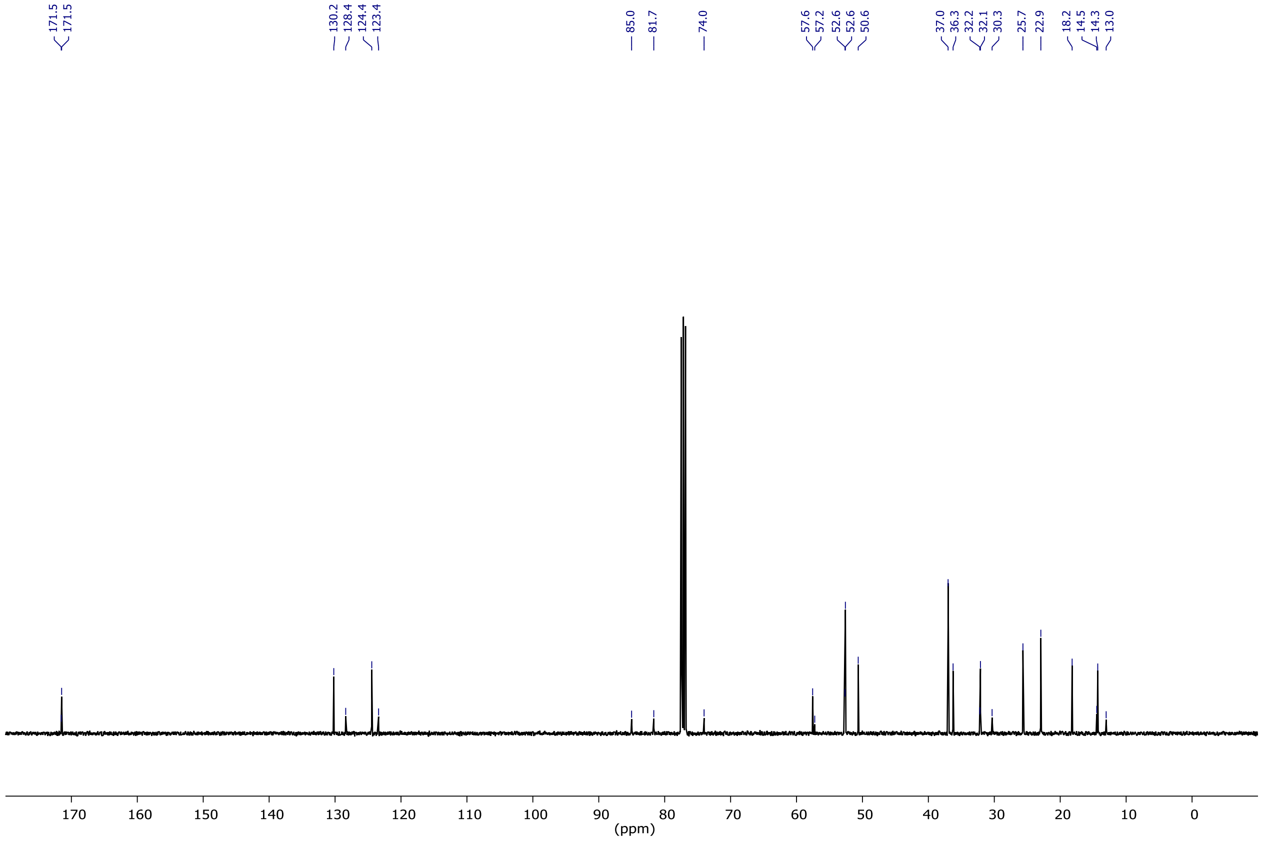
171.5
171.5
130.2
128.4
124.4
123.4
85.0
81.7
74.0
57.6
57.2
52.6
52.6
50.6
37.0
36.3
32.2
32.1
30.3
25.7
22.9
18.2
14.5
14.3
13.0
(ppm)

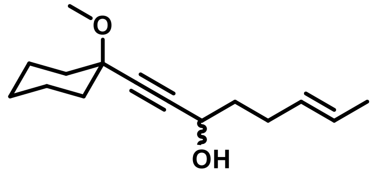
O
OH

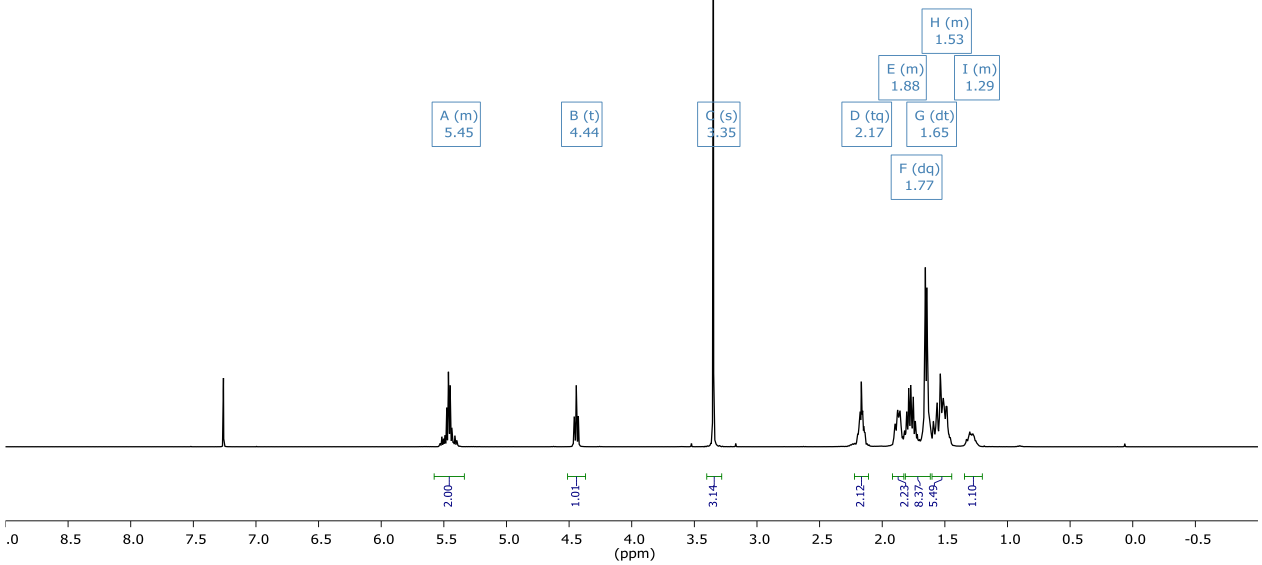
H (m)
1.53
E (m)
1.88
I (m)
1.29
A (m)
5.45
B (t)
4.44
C (s)
3.35
D (tq)
2.17
G (dt)
1.65
F (dq)
1.77
2.00
1.01
3.14
2.12
2.23
8.37
5.49
1.10
(ppm)

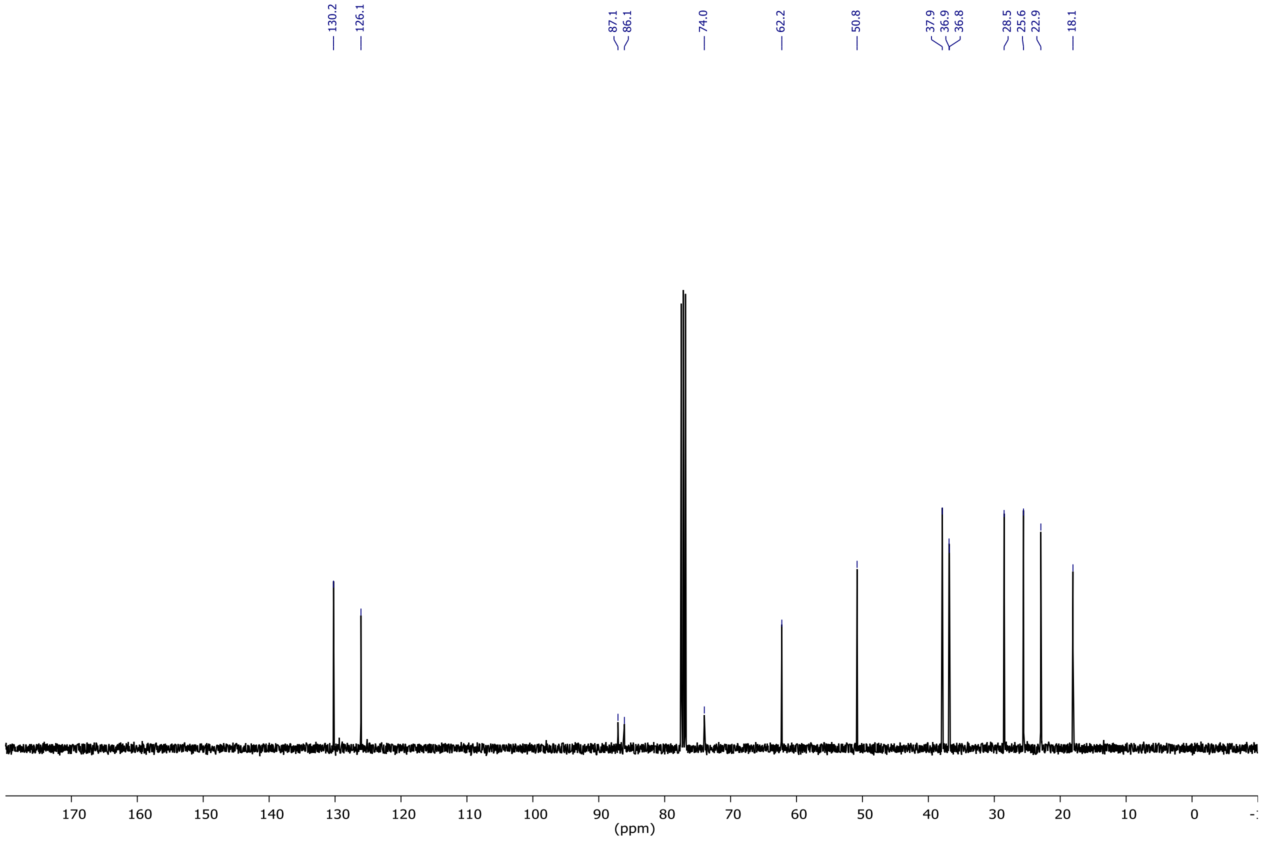
130.2
126.1
87.1
86.1
74.0
62.2
50.8
37.9
36.9
36.8
28.5
25.6
22.9
18.1
(ppm)

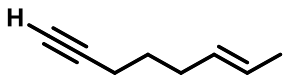

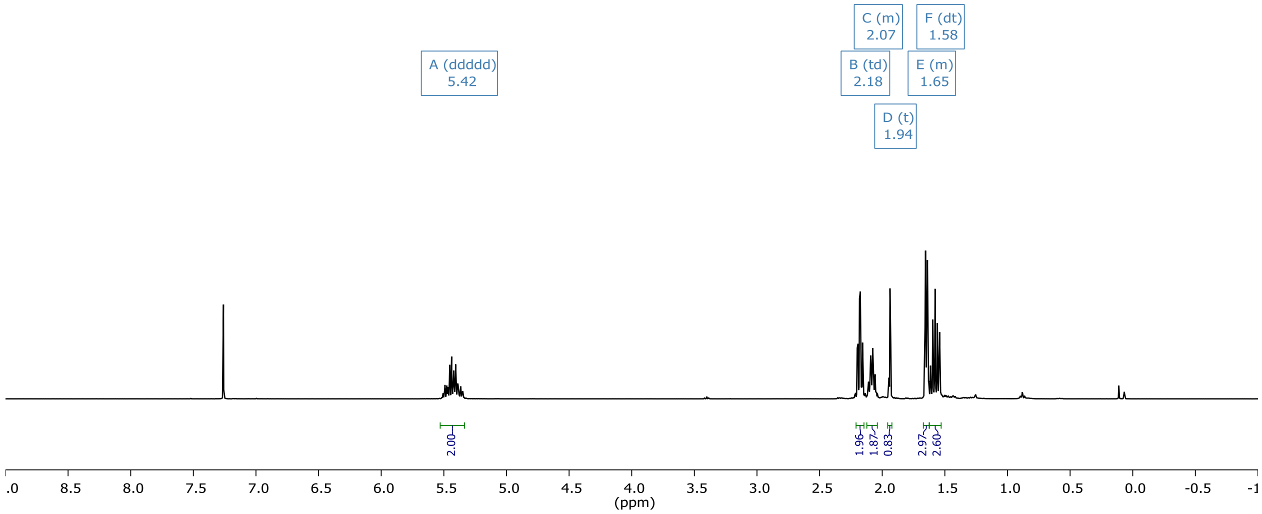
A (ddddd)
5.42
B (td)
2.18
C (m)
2.07
D (t)
1.94
E (m)
1.65
F (dt)
1.58
2.00
1.96
1.87
0.83
2.97
2.60
(ppm)

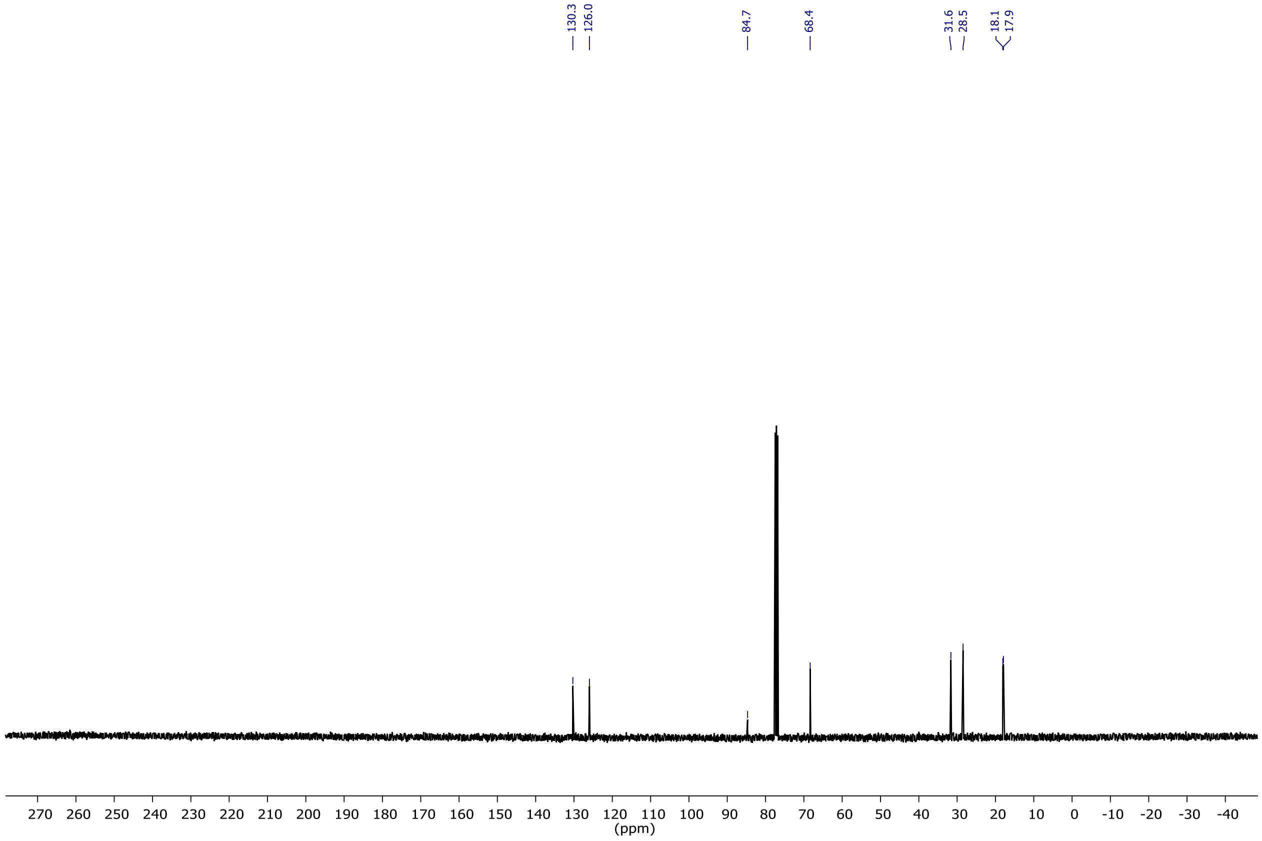
130.3
126.0
84.7
68.4
31.6
28.5
18.1
17.9
(ppm)

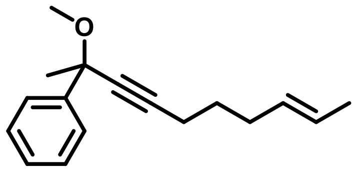

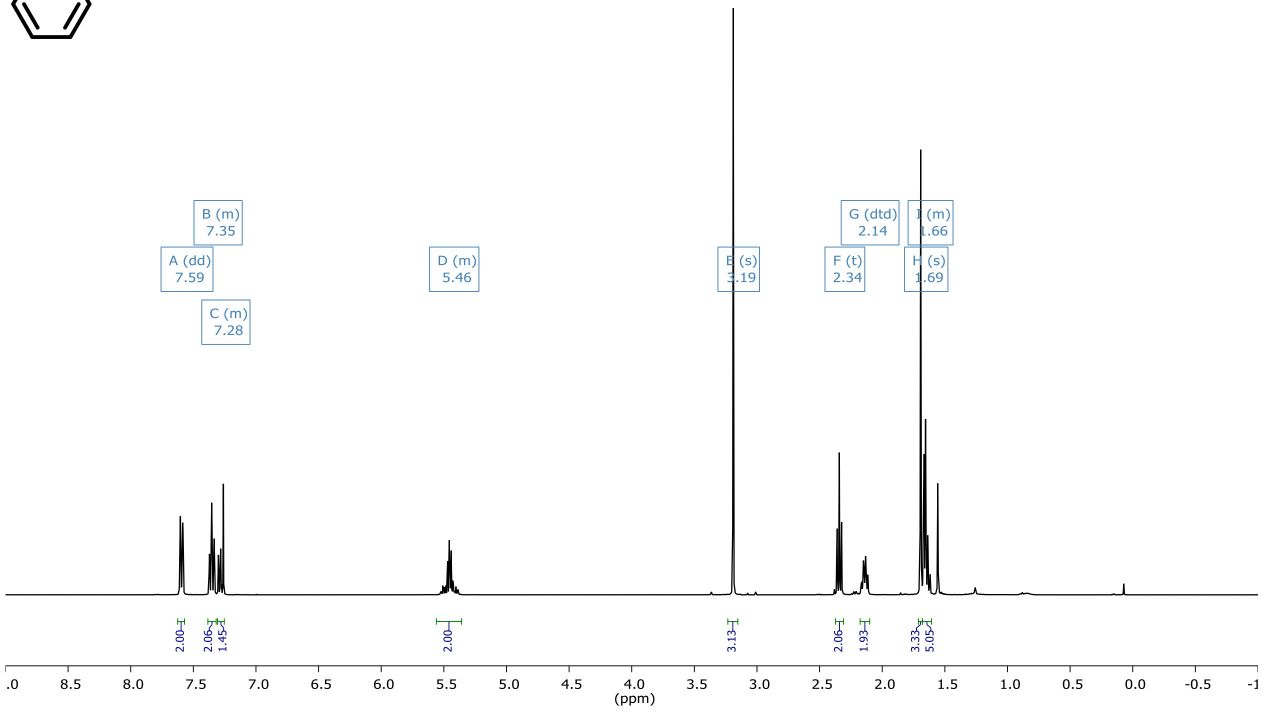
B (m)
7.35
A (dd)
7.59
C (m)
7.28
D (m)
5.46
E (s)
3.19
G (dtd)
2.14
F (t)
2.34
I (m)
1.66
H (s)
1.69
2.00
2.06
1.45
2.00
3.13
2.06
1.93
3.33
5.05
(ppm)

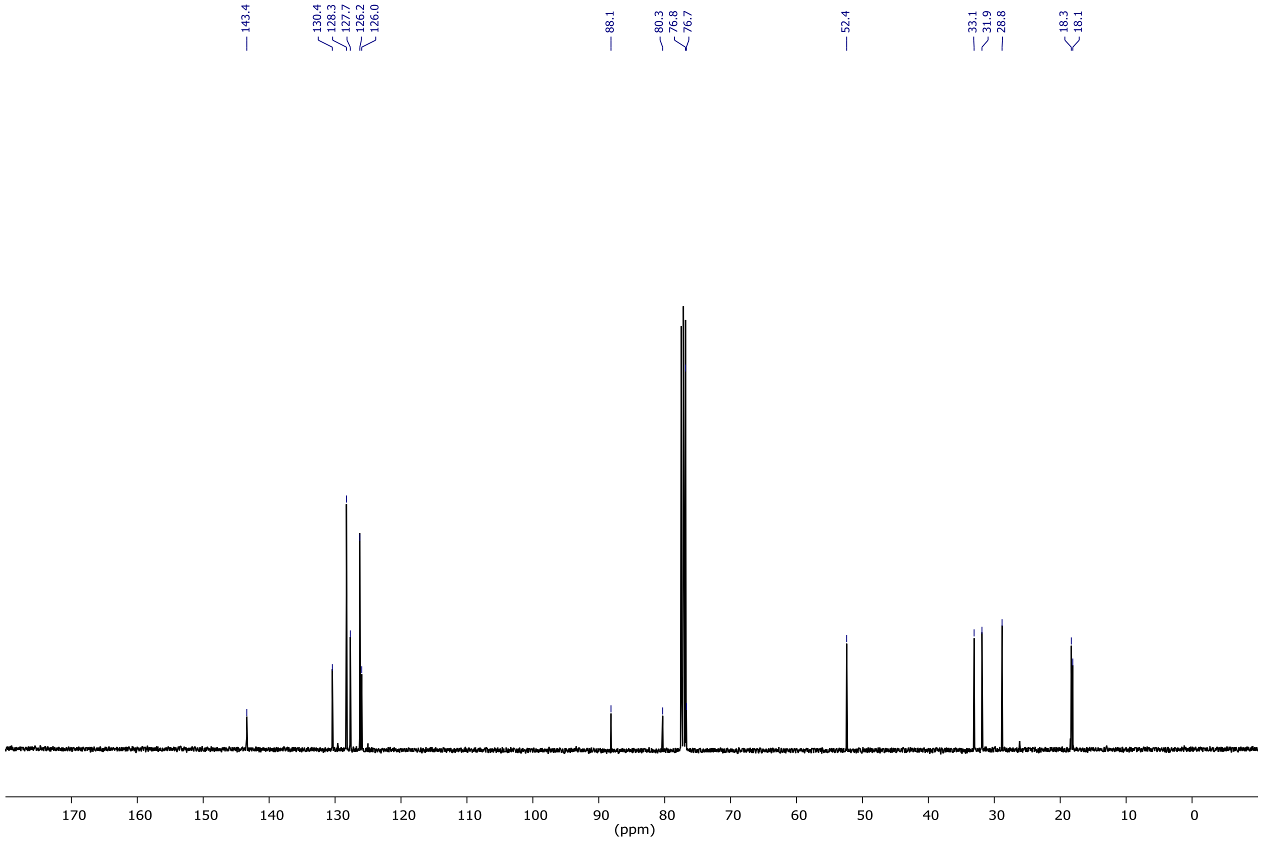
143.4
130.4
128.3
127.7
126.2
126.0
88.1
80.3
76.8
76.7
52.4
33.1
31.9
28.8
18.3
18.1
(ppm)

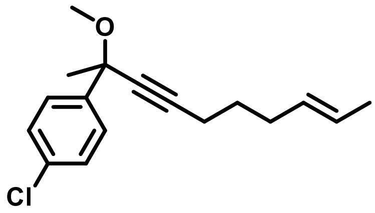

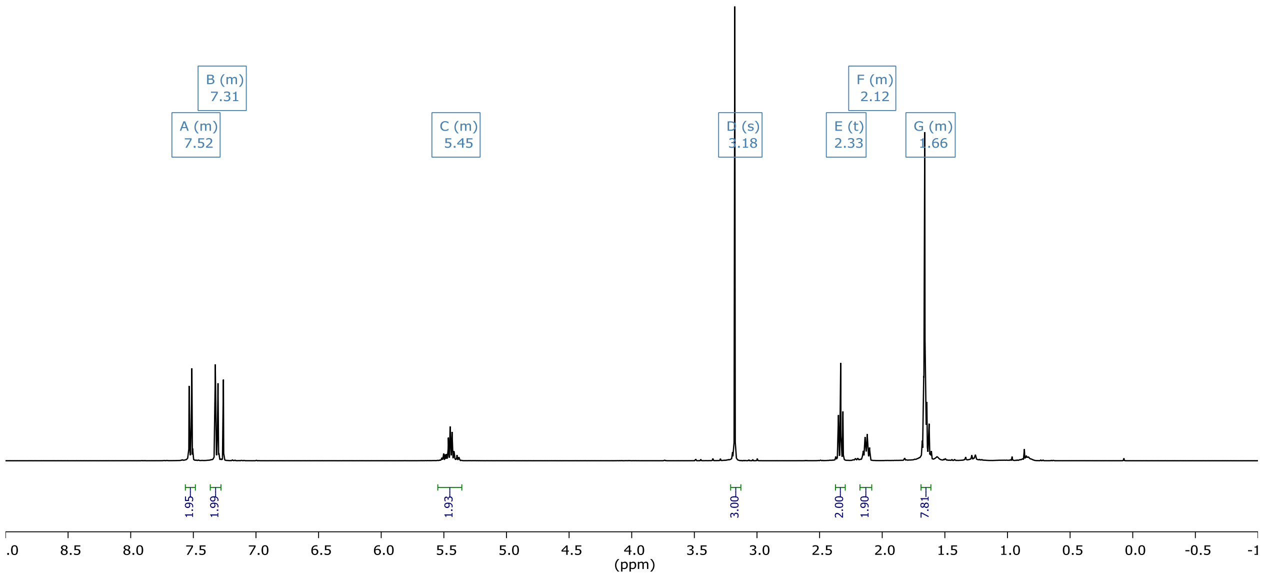

B (m)
7.31
A (m)
7.52
C (m)
5.45
D (s)
3.18
F (m)
2.12
E (t)
2.33
G (m)
1.66
1.95
1.99
1.93
3.00
2.00
1.90
7.81
(ppm)

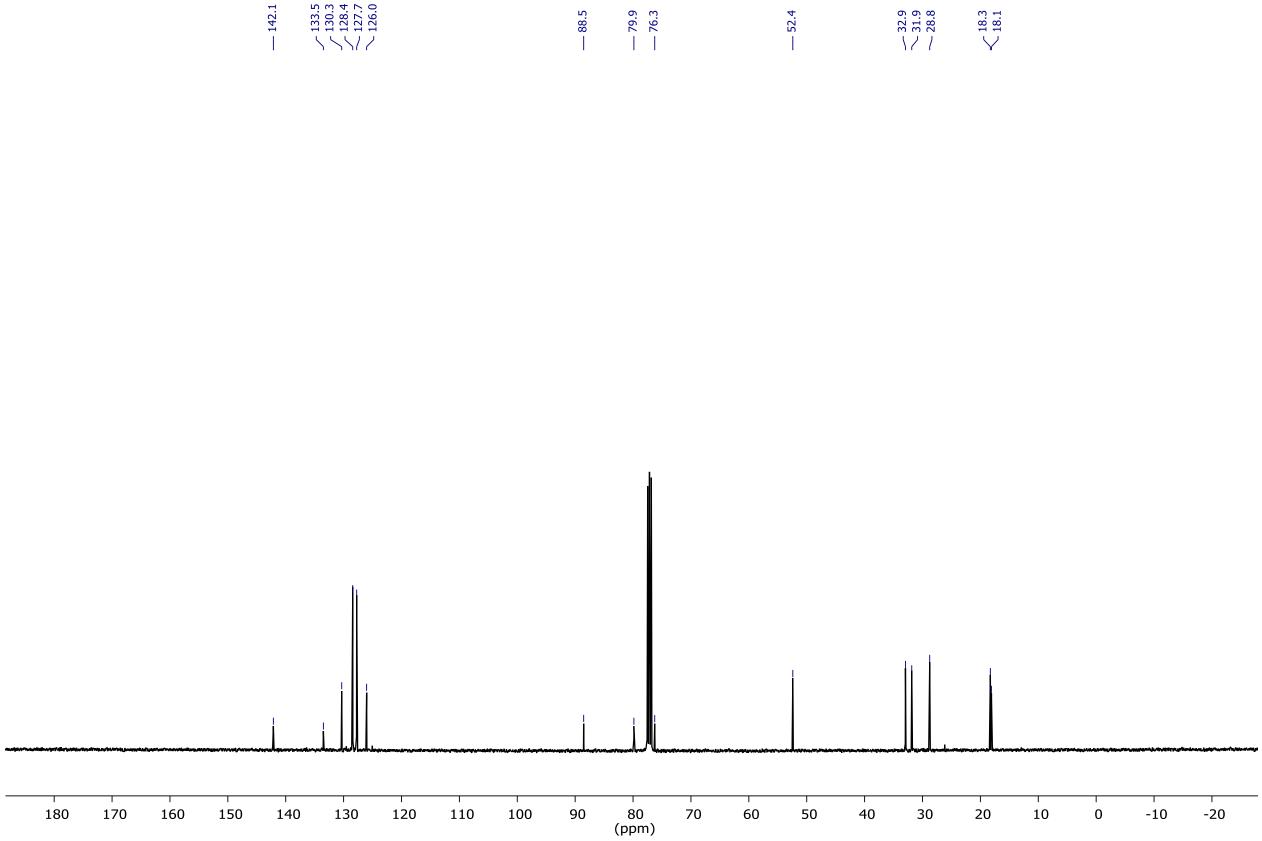

142.1
133.5
130.3
128.4
127.7
126.0
88.5
79.9
76.3
52.4
32.9
31.9
28.8
18.3
18.1
(ppm)

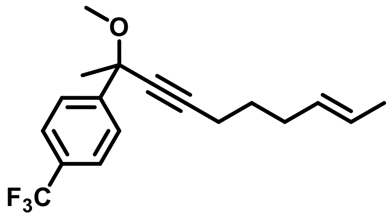

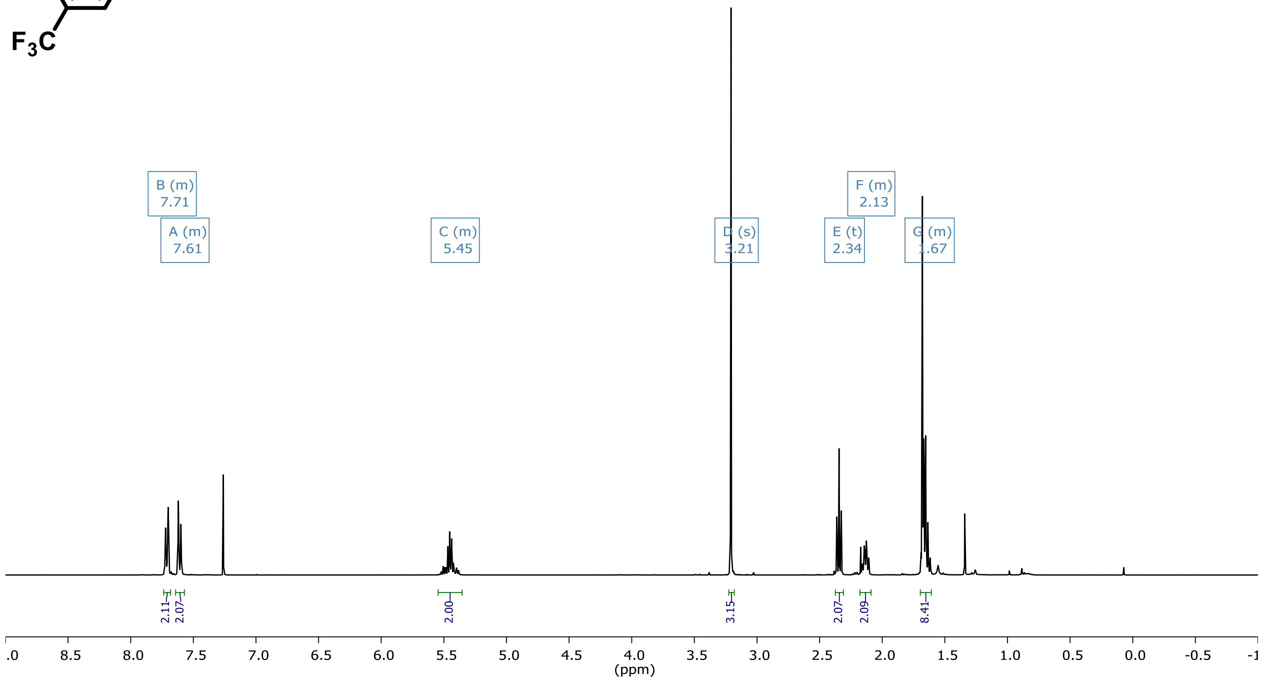
B (m)
7.71
A (m)
7.61
C (m)
5.45
D (s)
3.21
E (t)
2.34
F (m)
2.13
G (m)
1.67
2.11
2.07
2.00
3.15
2.07
2.09
8.41
(ppm)

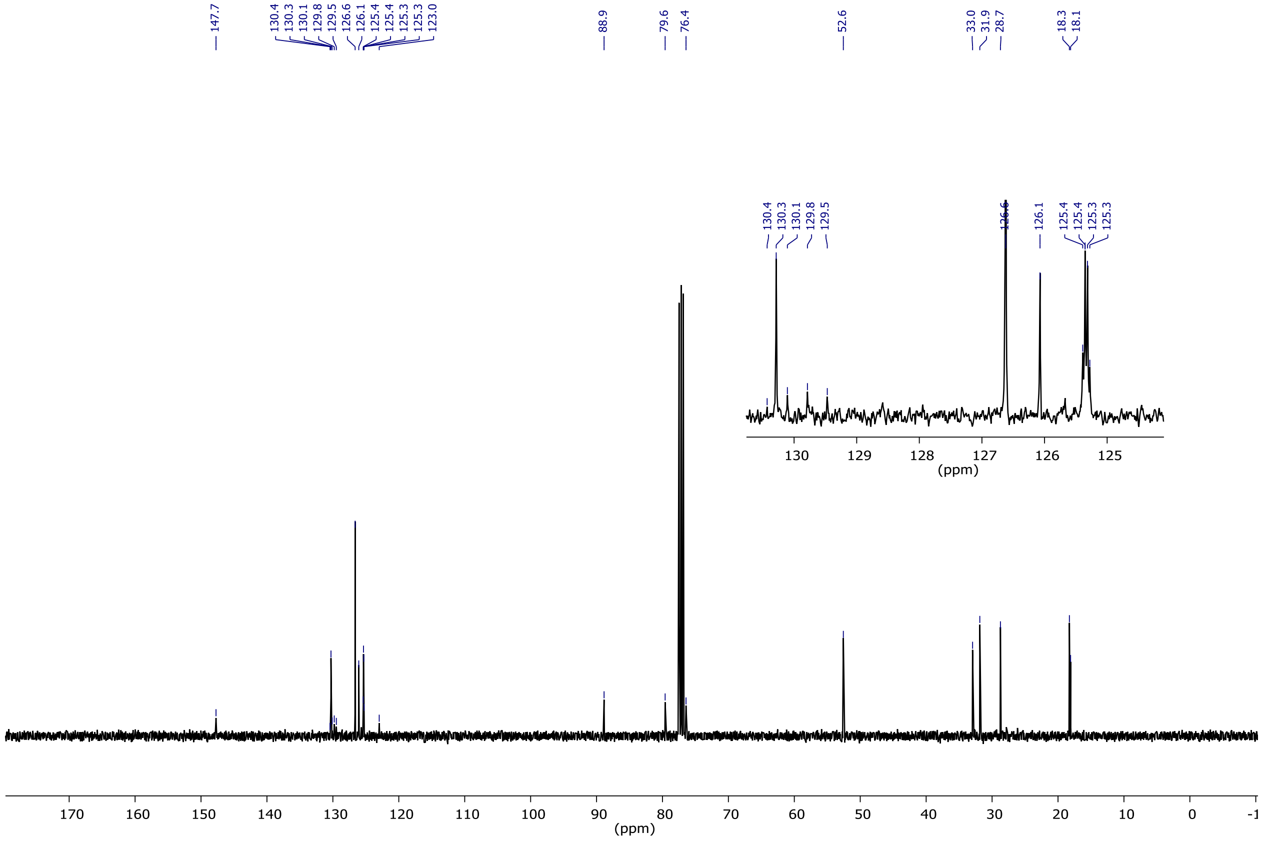
147.7
130.4
130.3
130.1
129.8
129.5
126.6
126.1
125.4
125.4
125.3
125.3
123.0
88.9
79.6
76.4
52.6
33.0
31.9
28.7
18.3
18.1
(ppm)

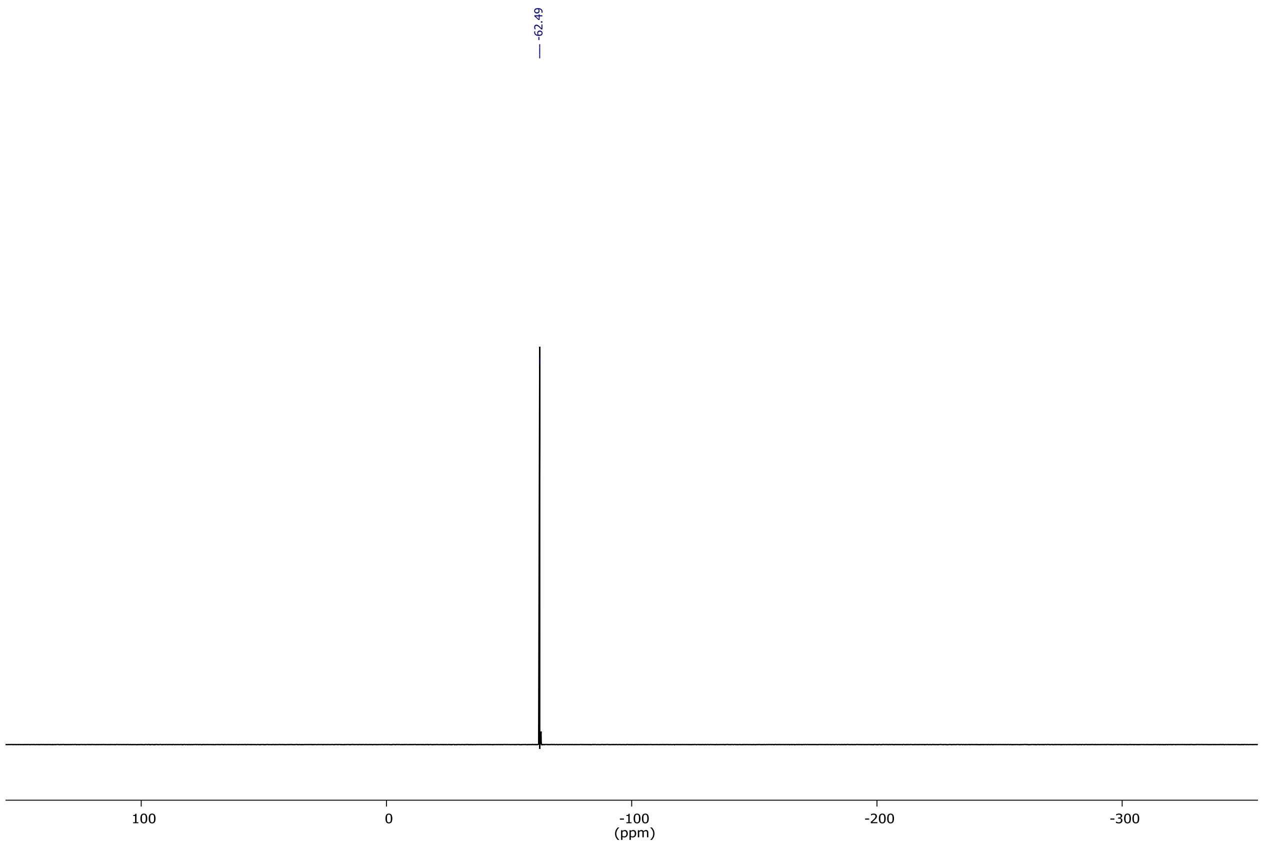
-62.49
100
0
-100
(ppm)
-200
-300

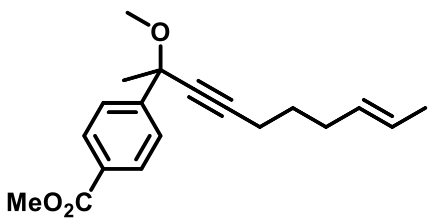
MeO2C
O

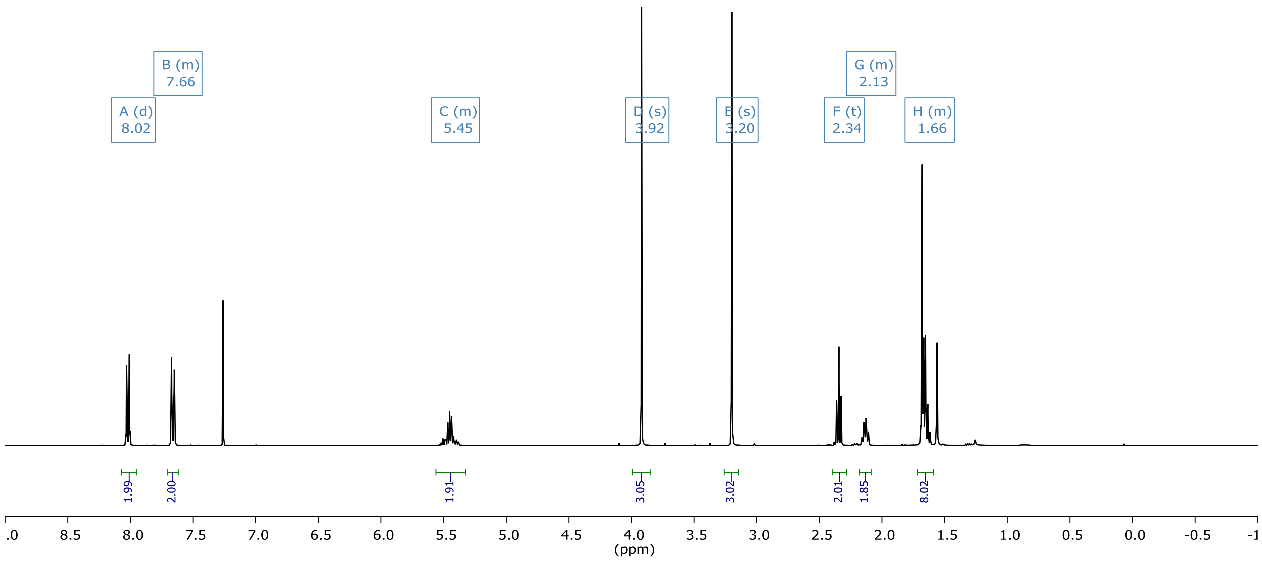

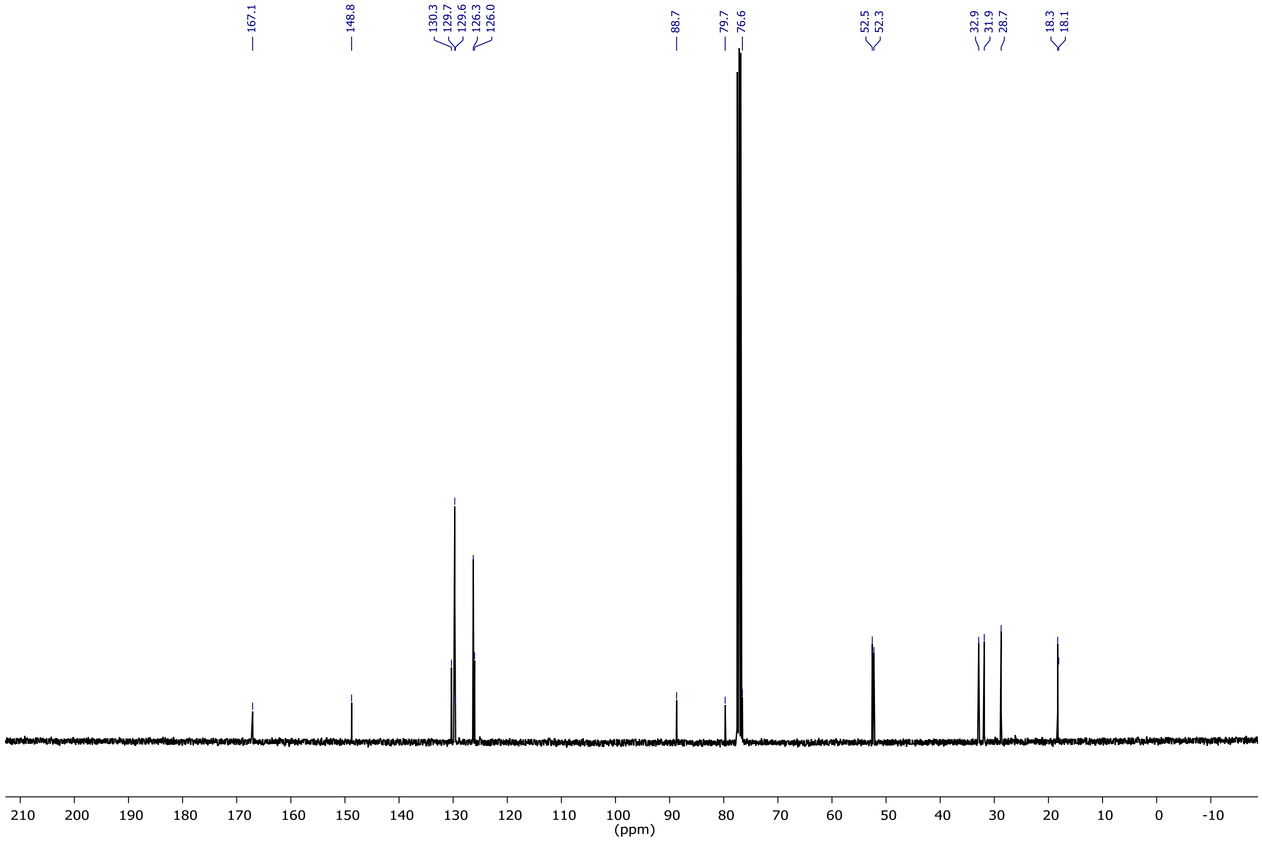

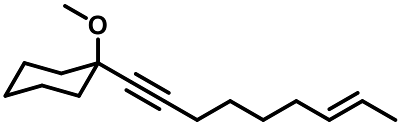

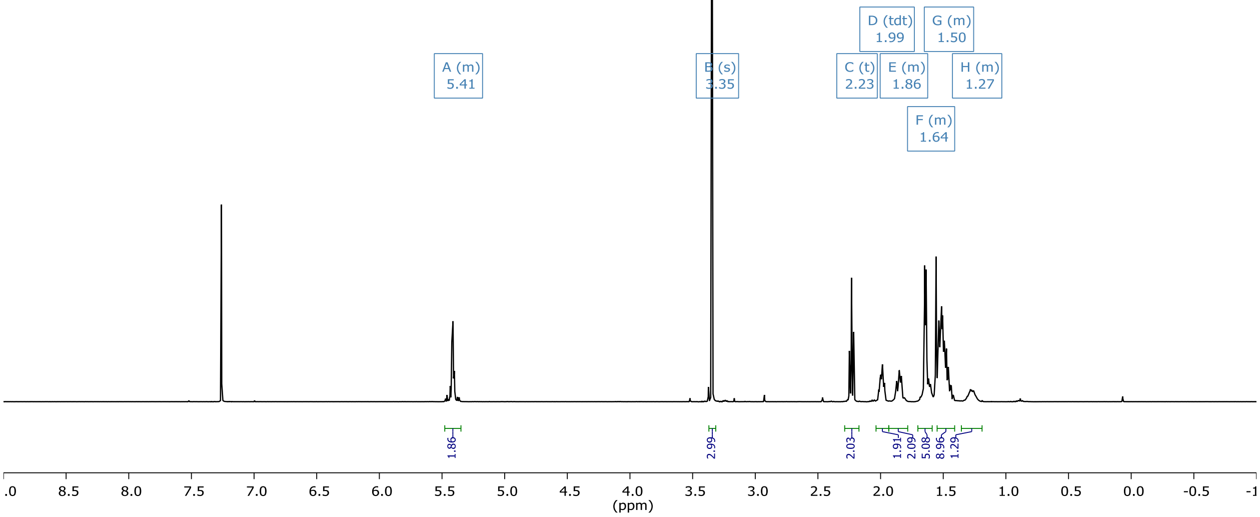
A (m)
5.41
B (s)
3.35
C (t)
2.23
D (tdt)
1.99
E (m)
1.86
F (m)
1.64
G (m)
1.50
H (m)
1.27
1.86
2.99
2.03
1.91
2.09
5.08
8.96
1.29
(ppm)

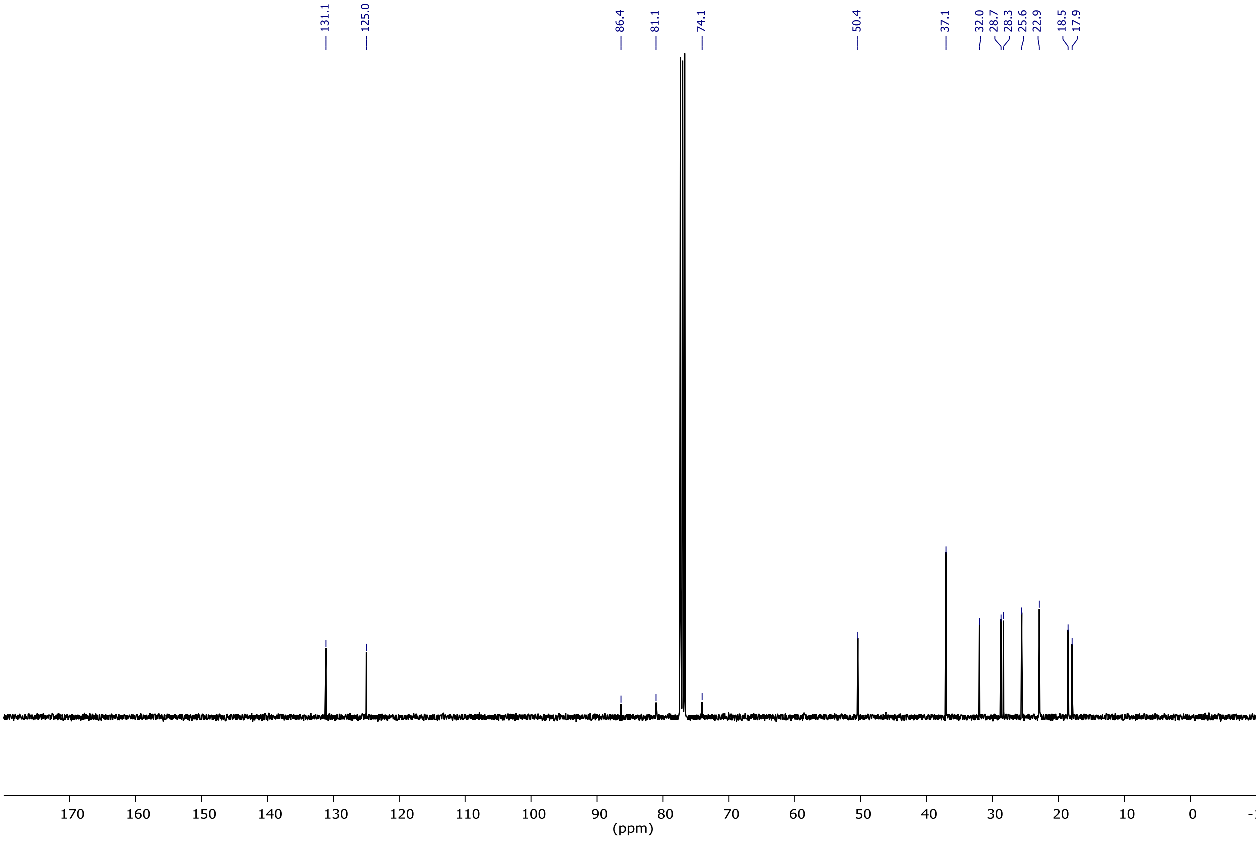
131.1
125.0
86.4
81.1
74.1
50.4
37.1
32.0
28.7
28.3
25.6
22.9
18.5
17.9
(ppm)

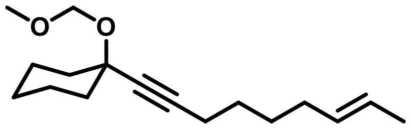

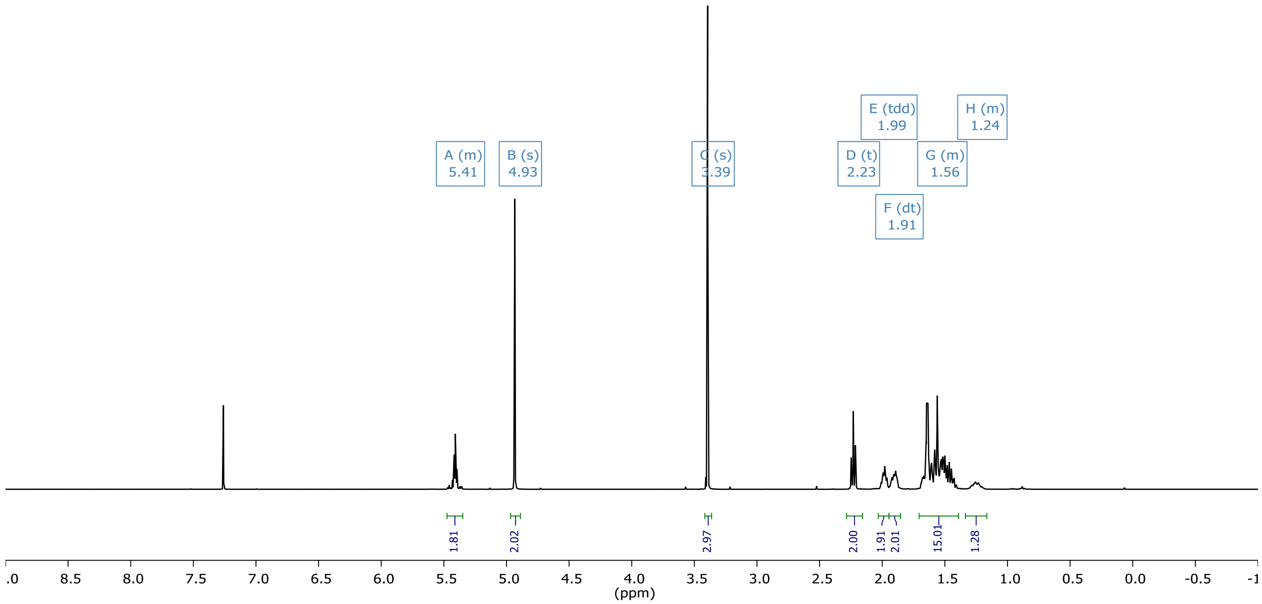
A (m) 5.41
B (s) 4.93
C (s) 3.39
D (t) 2.23
E (tdd) 1.99
F (dt) 1.91
G (m) 1.56
H (m) 1.24
1.81
2.02
2.97
2.00
1.91
2.01
15.01
1.28
(ppm)

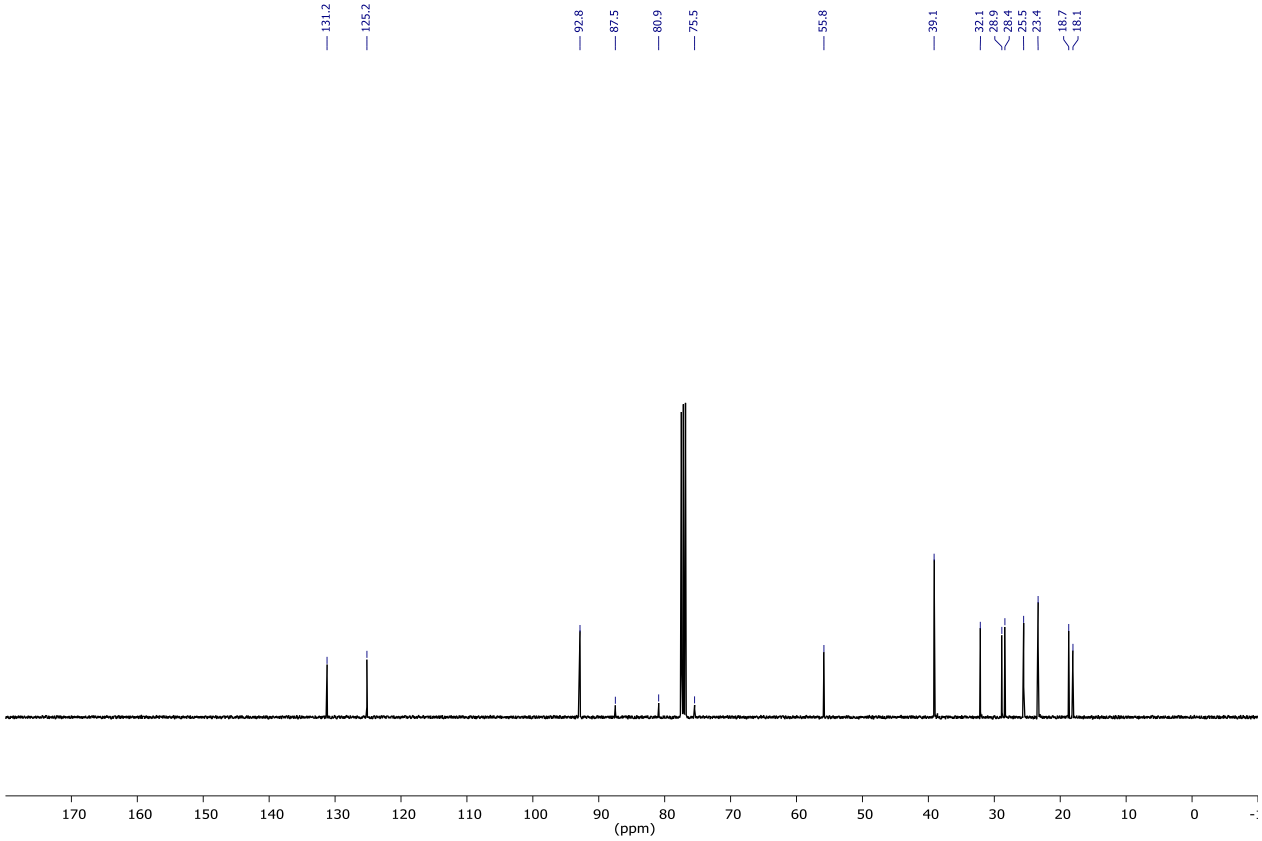
131.2
125.2
92.8
87.5
80.9
75.5
55.8
39.1
32.1
28.9
28.4
25.5
23.4
18.7
18.1
(ppm)

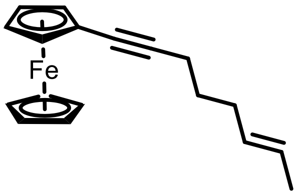

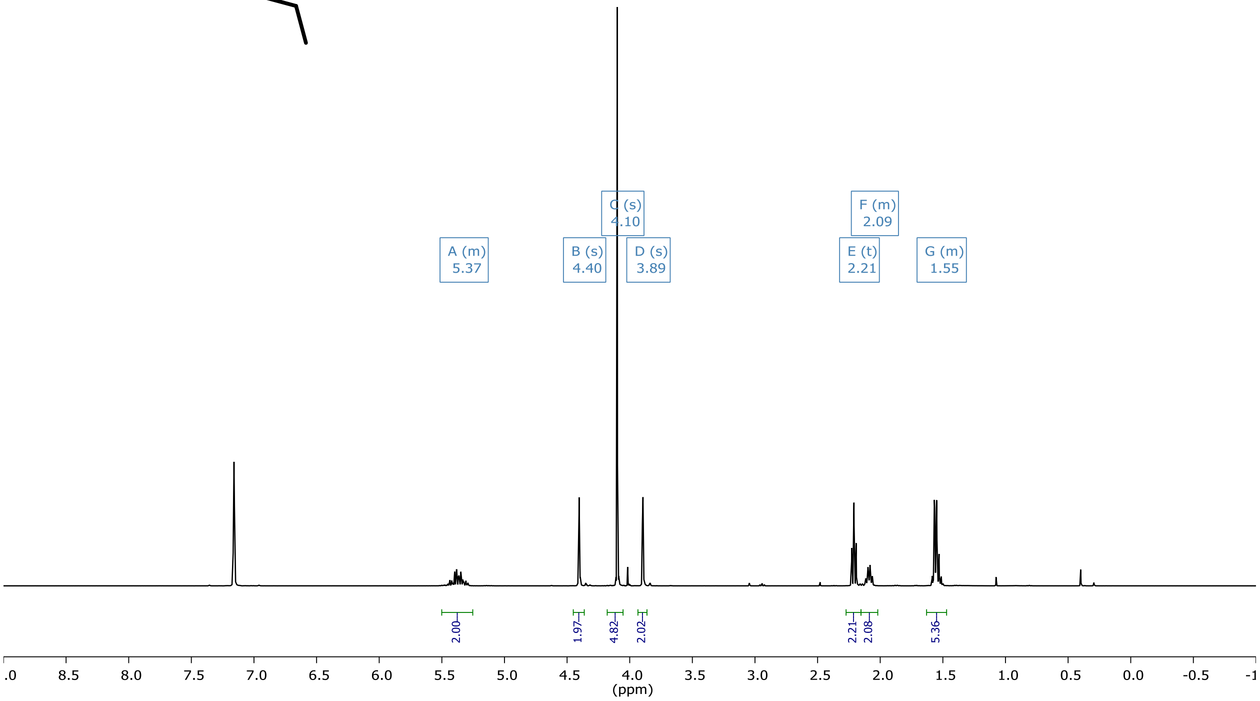
A (m)
5.37
B (s)
4.40
C (s)
4.10
D (s)
3.89
E (t)
2.21
F (m)
2.09
G (m)
1.55
2.00
1.97
4.82
2.02
2.21
2.08
5.36
(ppm)

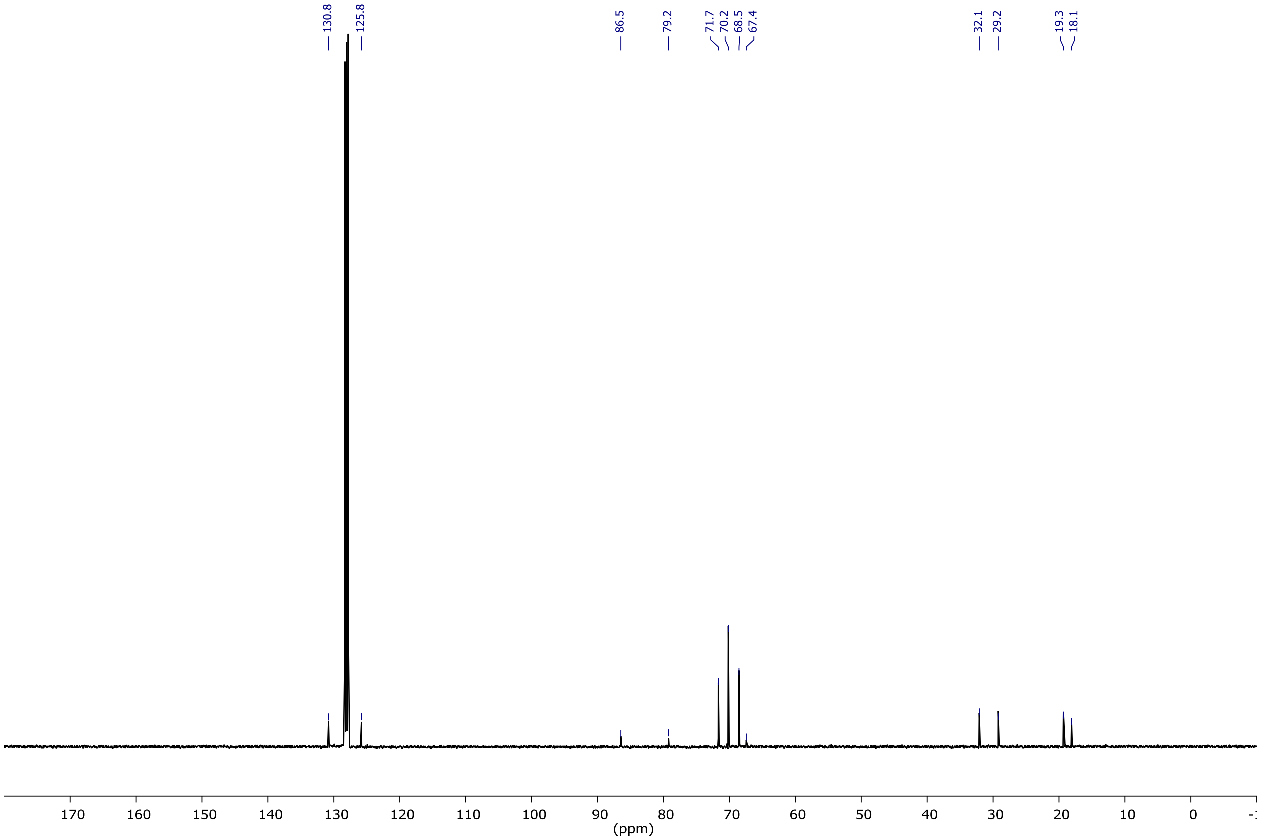
130.8
125.8
86.5
79.2
71.7
70.2
68.5
67.4
32.1
29.2
19.3
18.1
(ppm)

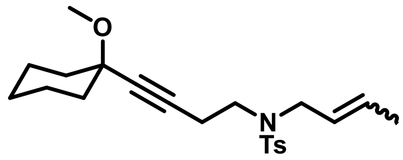
O
N
Ts

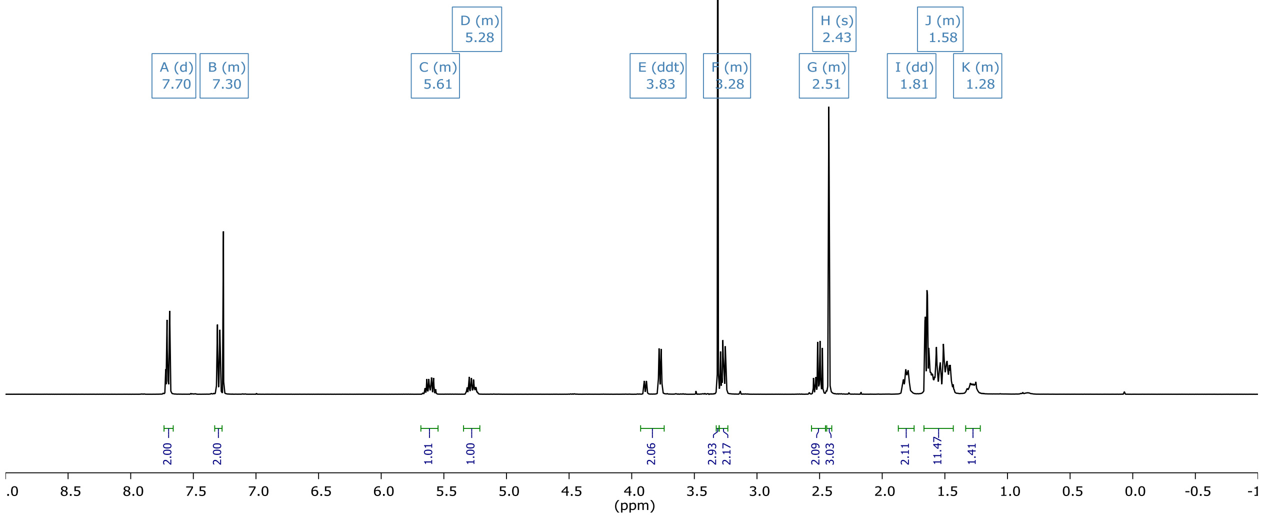
A (d)
7.70
B (m)
7.30
C (m)
5.61
D (m)
5.28
E (ddt)
3.83
F (m)
3.28
G (m)
2.51
H (s)
2.43
I (dd)
1.81
J (m)
1.58
K (m)
1.28
2.00
2.00
1.01
1.00
2.06
2.93
2.17
2.09
3.03
2.11
11.47
1.41
(ppm)

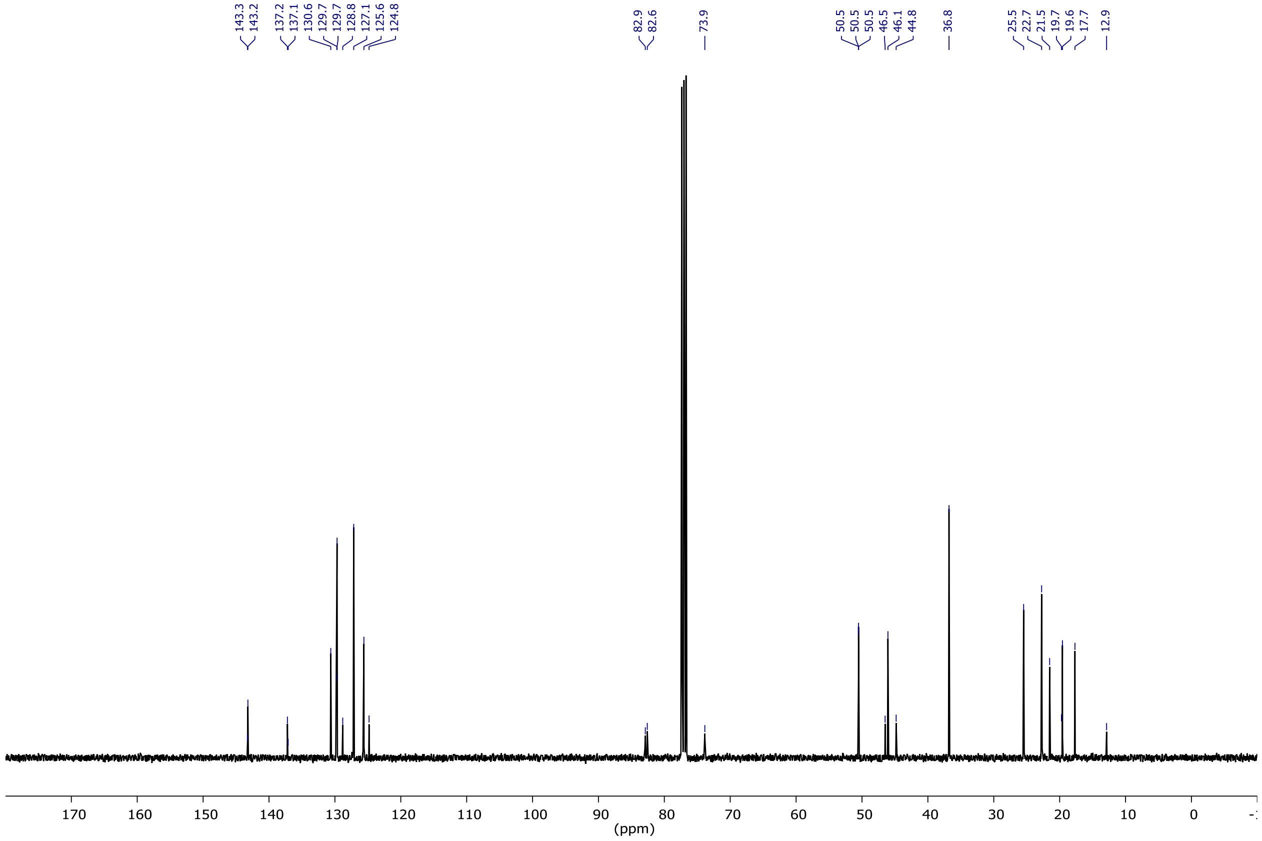
143.3
143.2
137.2
137.1
130.6
129.7
129.7
128.8
127.1
125.6
124.8
82.9
82.6
73.9
50.5
50.5
50.5
46.5
46.1
44.8
36.8
25.5
22.7
21.5
19.7
19.6
17.7
12.9
(ppm)

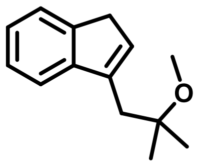
O

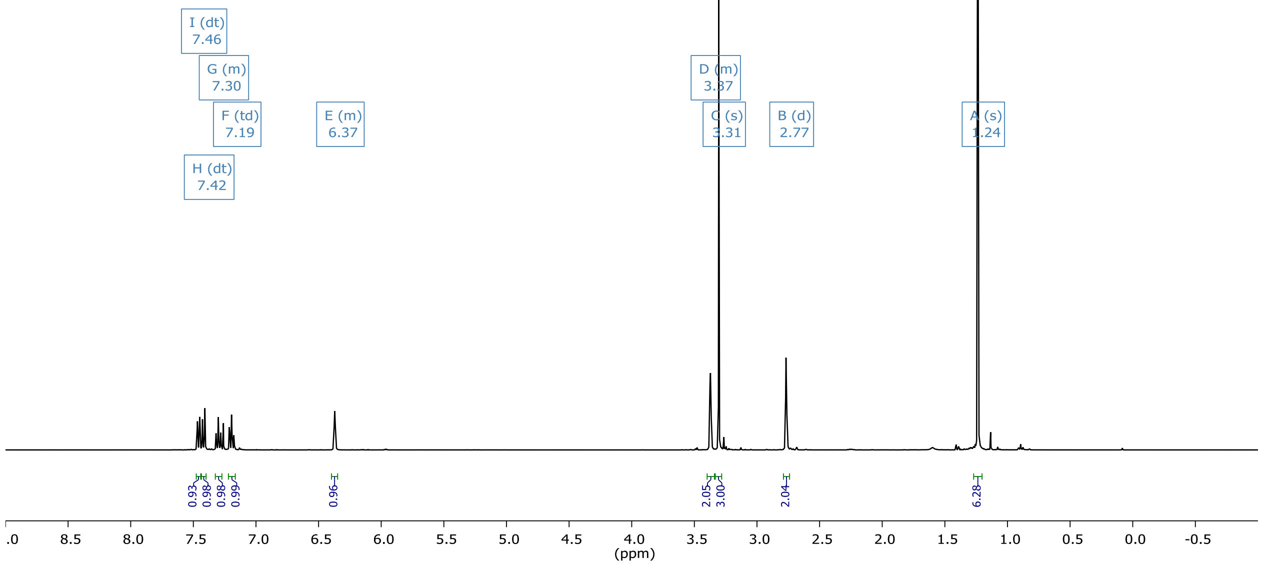
I (dt)
7.46
G (m)
7.30
F (td)
7.19
H (dt)
7.42
E (m)
6.37
D (m)
3.37
C (s)
3.31
B (d)
2.77
A (s)
1.24
0.93
0.98
0.98
0.99
0.96
2.05
3.00
2.04
6.28
.0 8.5 8.0 7.5 7.0 6.5 6.0 5.5 5.0 4.5 4.0 3.5 3.0 2.5 2.0 1.5 1.0 0.5 0.0 -0.5
(ppm)

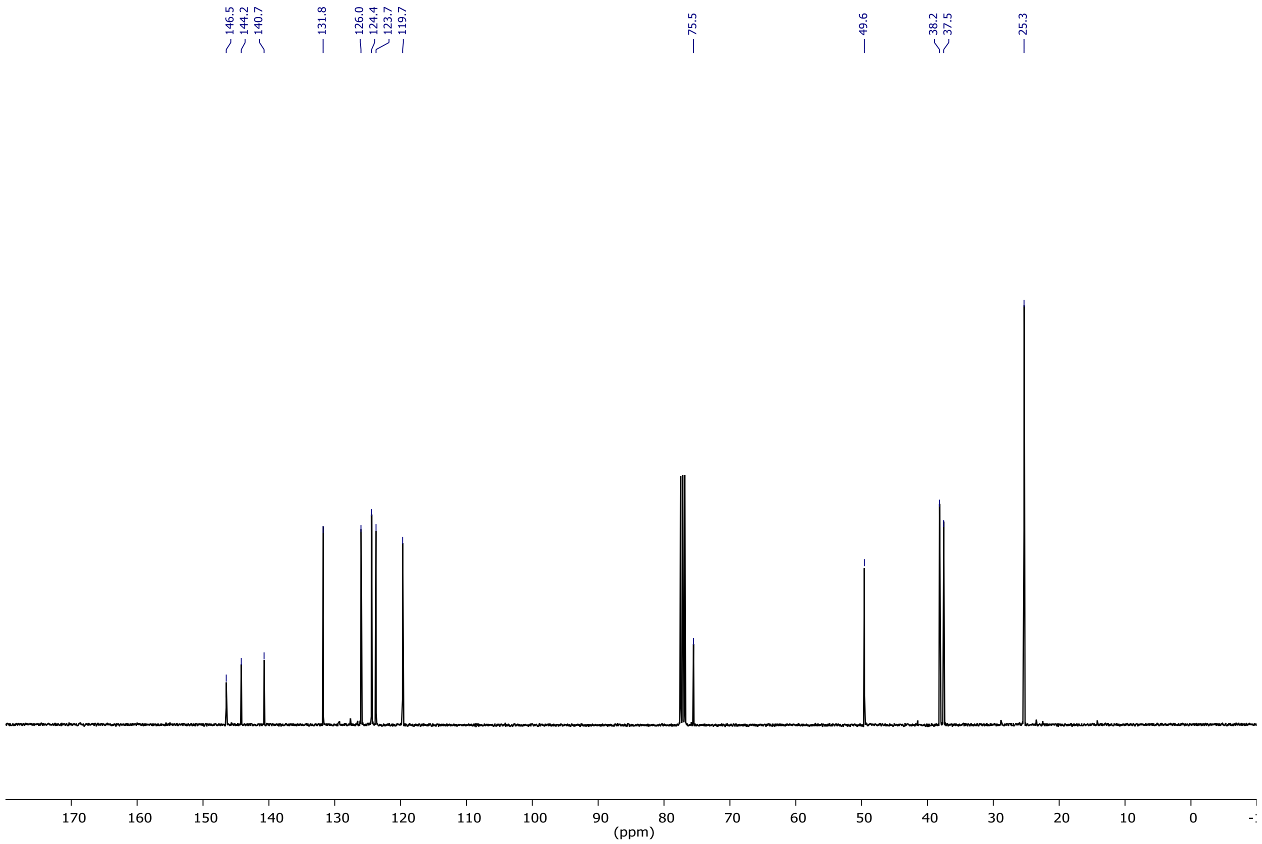
146.5
144.2
140.7
131.8
126.0
124.4
123.7
119.7
75.5
49.6
38.2
37.5
25.3
170 160 150 140 130 120 110 100 90 80 70 60 50 40 30 20 10 0
(ppm)

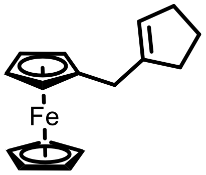

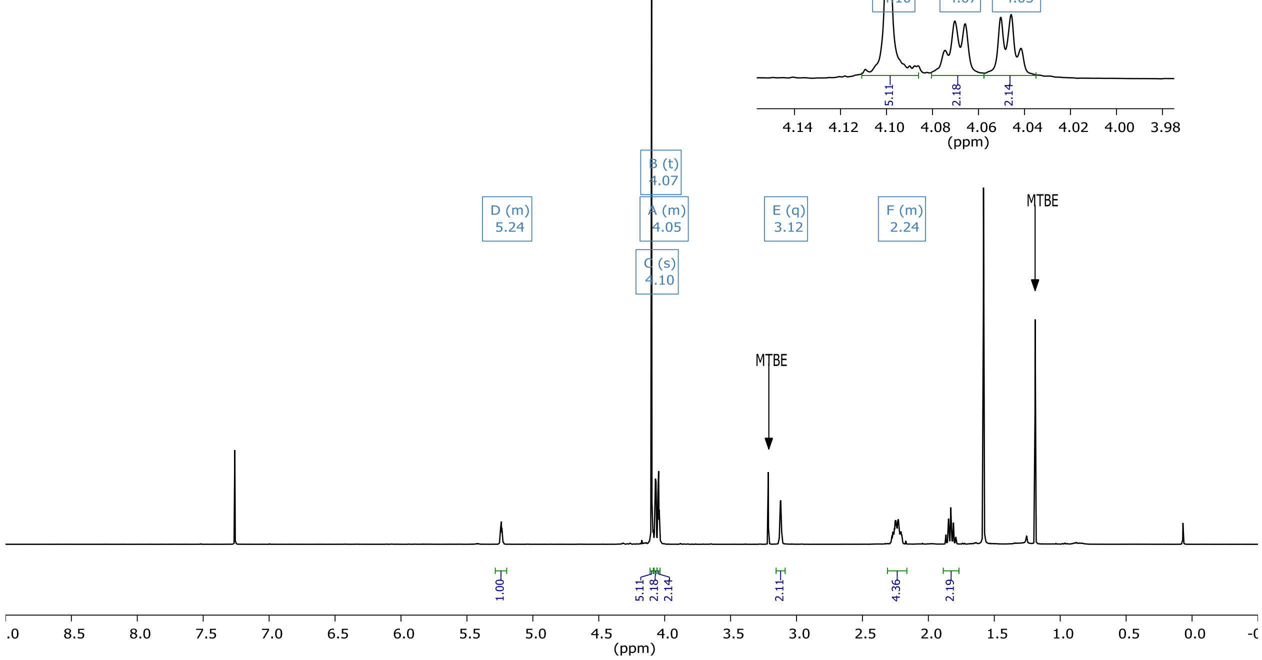
C (s)
4.10
B (t)
4.07
A (m)
4.05
5.11
2.18
2.14
4.14 4.12 4.10 4.08 4.06 4.04 4.02 4.00 3.98
(ppm)
D (m)
5.24
E (q)
3.12
F (m)
2.24
MTBE
MTBE
1.00
2.11
4.36
2.19
8.5 8.0 7.5 7.0 6.5 6.0 5.5 5.0 4.5 4.0 3.5 3.0 2.5 2.0 1.5 1.0 0.5 0.0
(ppm)

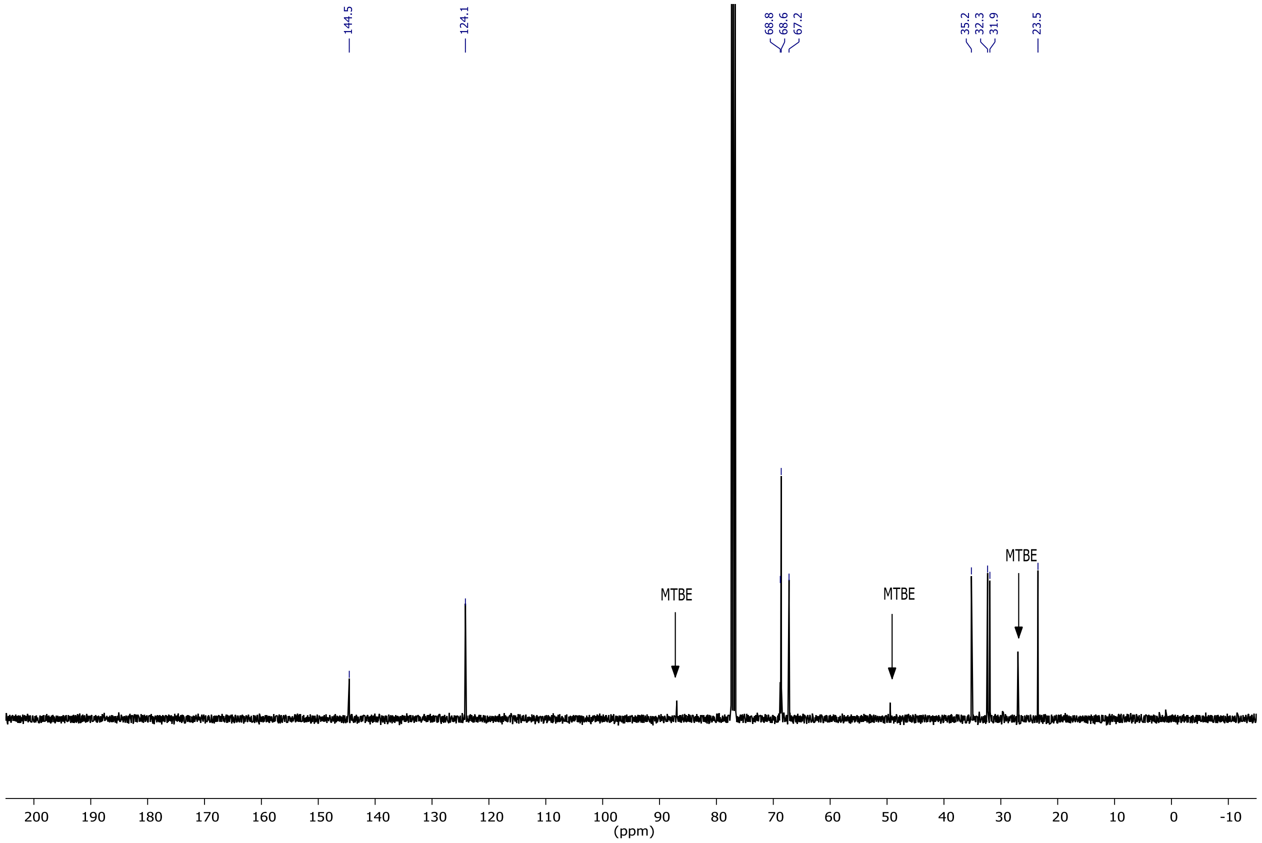
144.5
124.1
68.8
68.6
67.2
35.2
32.3
31.9
23.5
MTBE
MTBE
MTBE
200 190 180 170 160 150 140 130 120 110 100 90 80 70 60 50 40 30 20 10 0 -10
(ppm)

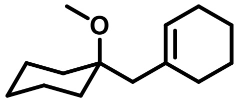

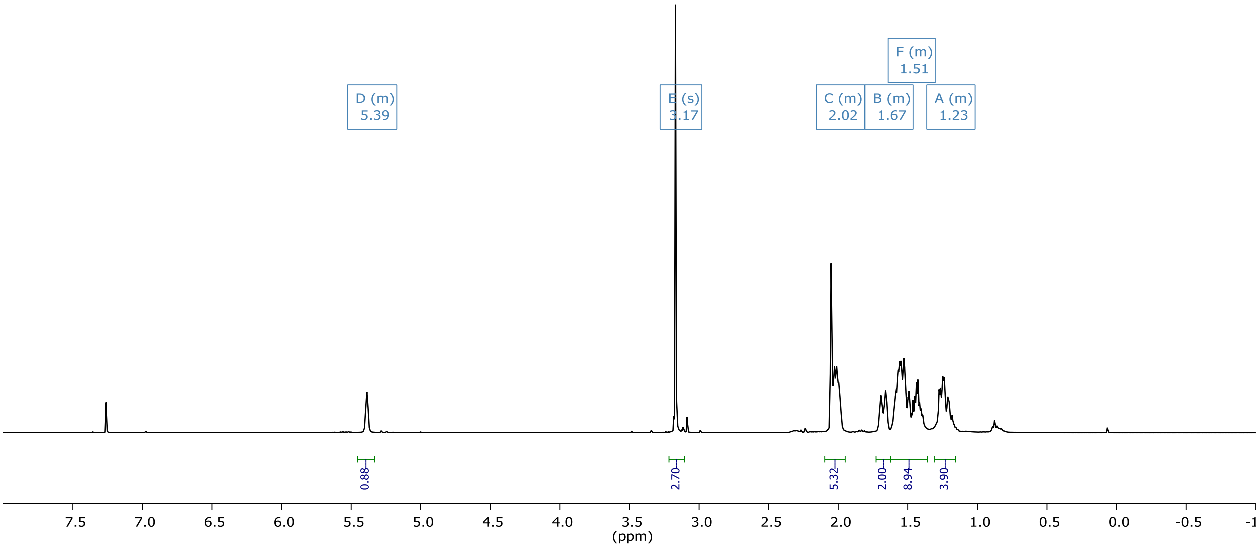
D (m)
5.39
E (s)
3.17
C (m)
2.02
B (m)
1.67
F (m)
1.51
A (m)
1.23
0.88
2.70
5.32
2.00
8.94
3.90
(ppm)

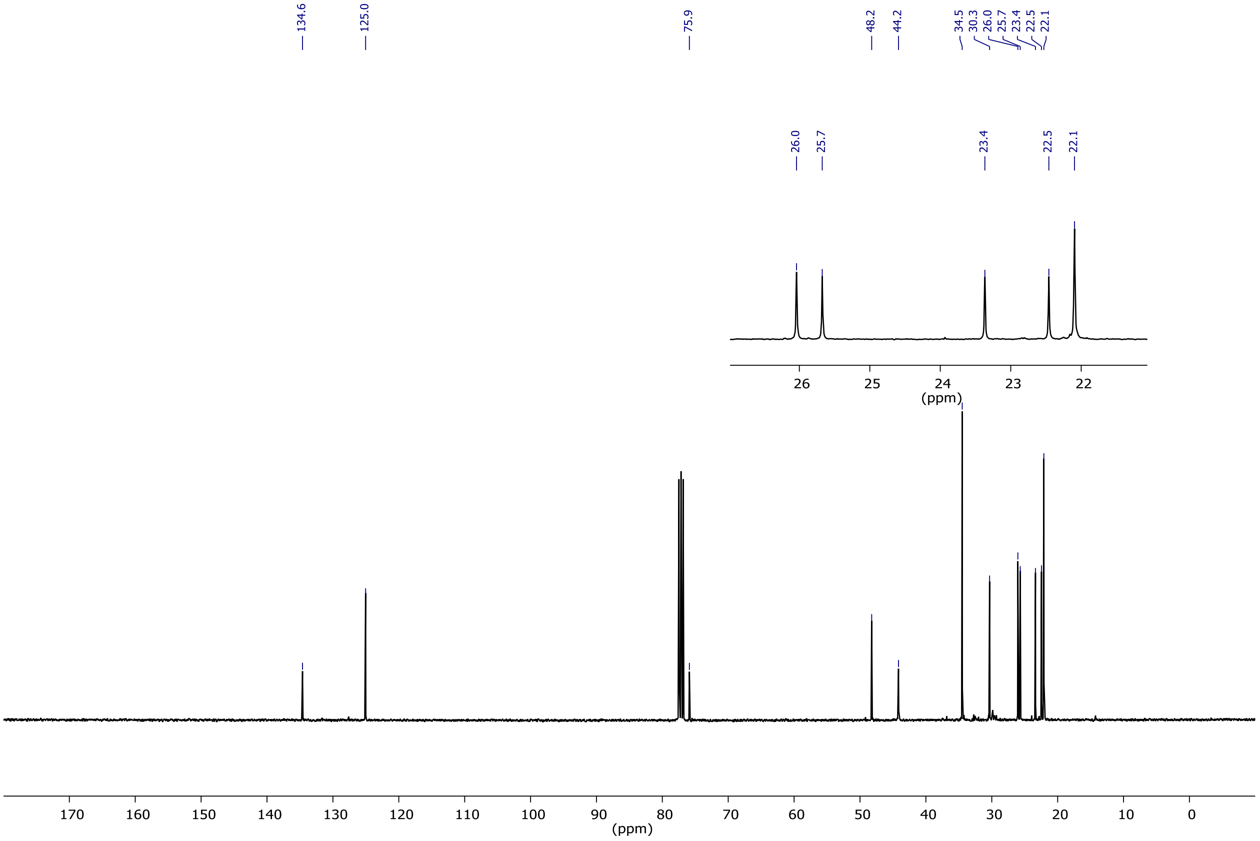
134.6
125.0
75.9
48.2
44.2
34.5
30.3
26.0
25.7
23.4
22.5
22.1
(ppm)

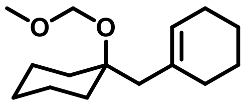

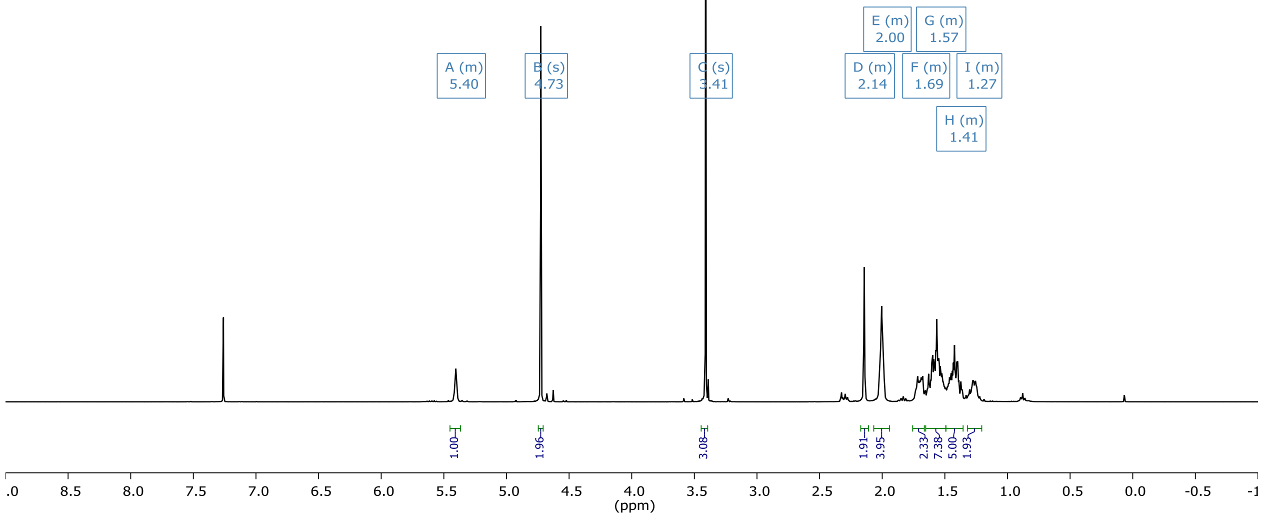
A (m)
5.40
B (s)
4.73
C (s)
3.41
E (m)
2.00
G (m)
1.57
D (m)
2.14
F (m)
1.69
I (m)
1.27
H (m)
1.41
1.00
1.96
3.08
1.91
3.95
2.33
7.38
5.00
1.93
(ppm)

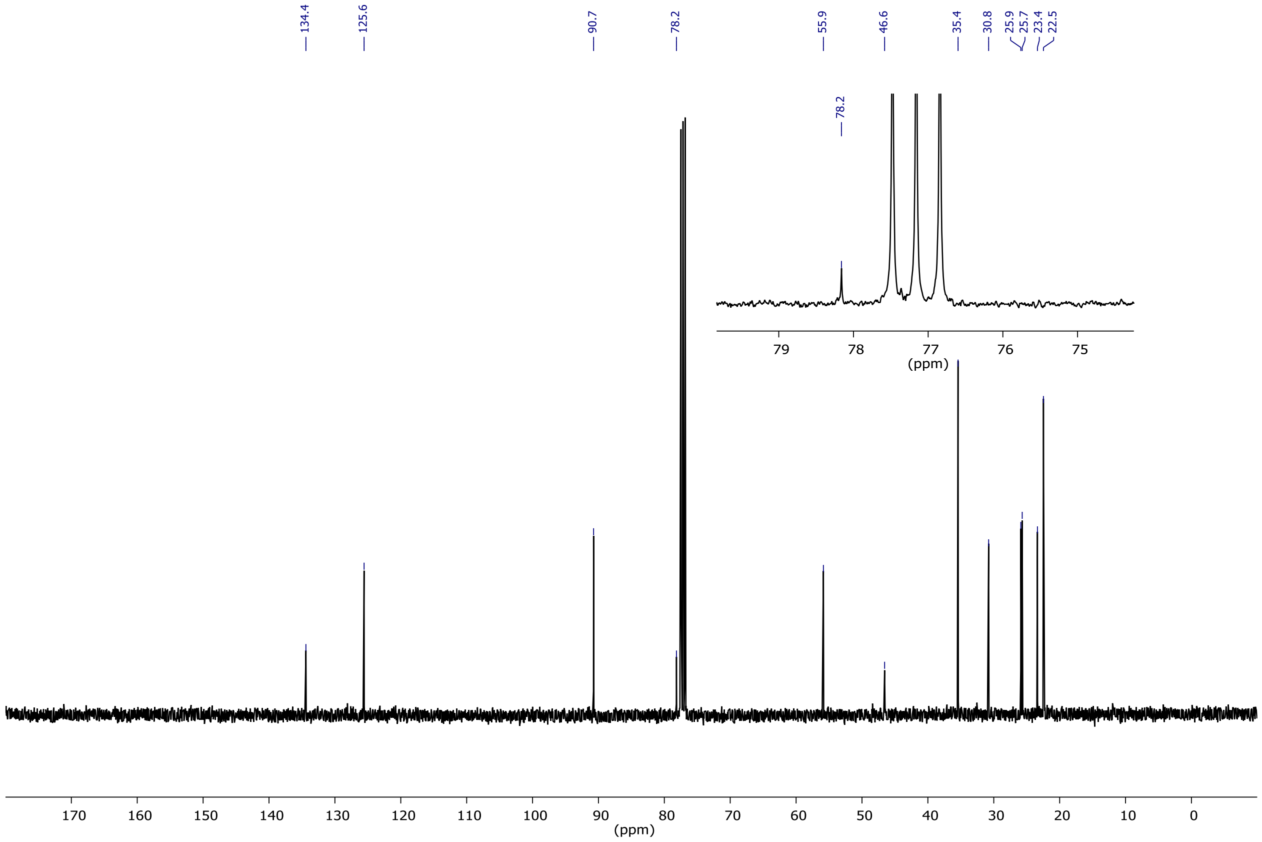
134.4
125.6
90.7
78.2
55.9
46.6
35.4
30.8
25.9
25.7
23.4
22.5
(ppm)

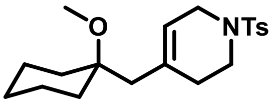
O
NTs

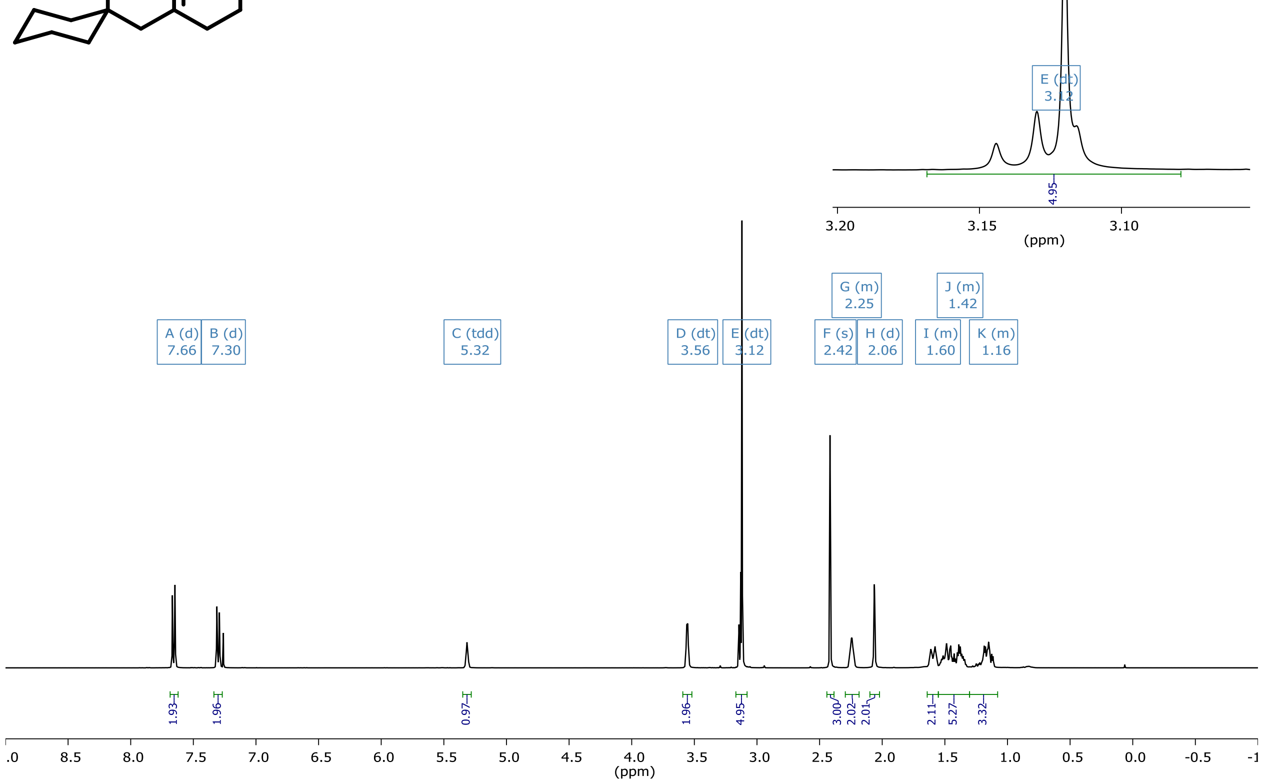
E (dt)
3.12
4.95
3.20
3.15
3.10
(ppm)
A (d)
7.66
B (d)
7.30
C (tdd)
5.32
D (dt)
3.56
E (dt)
3.12
F (s)
2.42
G (m)
2.25
H (d)
2.06
I (m)
1.60
J (m)
1.42
K (m)
1.16
1.93
1.96
0.97
1.96
4.95
3.00
2.02
2.01
2.11
5.27
3.32
(ppm)

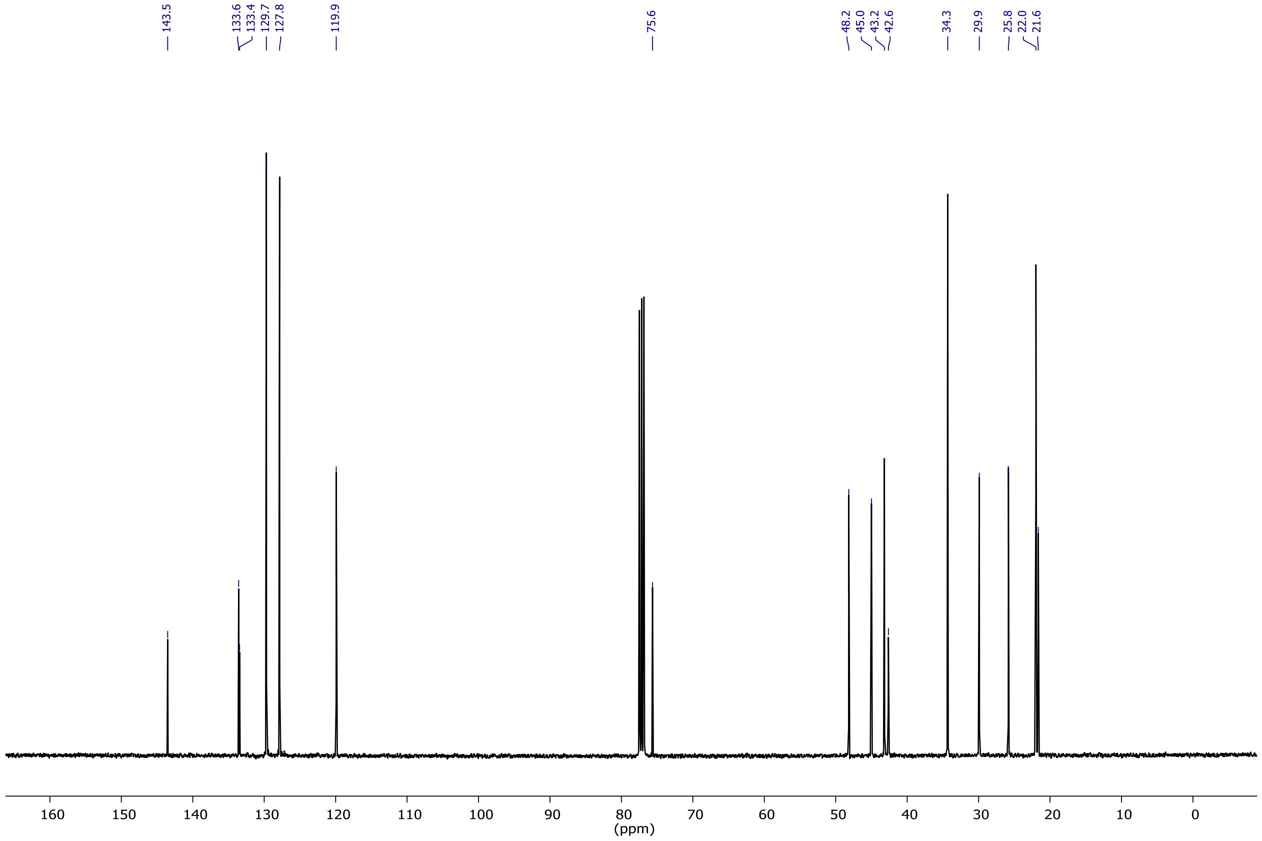
143.5
133.6
133.4
129.7
127.8
119.9
75.6
48.2
45.0
43.2
42.6
34.3
29.9
25.8
22.0
21.6
(ppm)

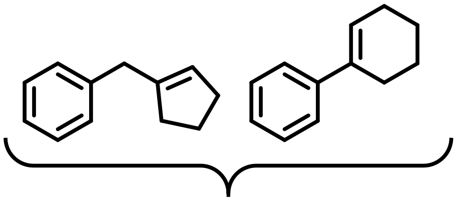

2:1

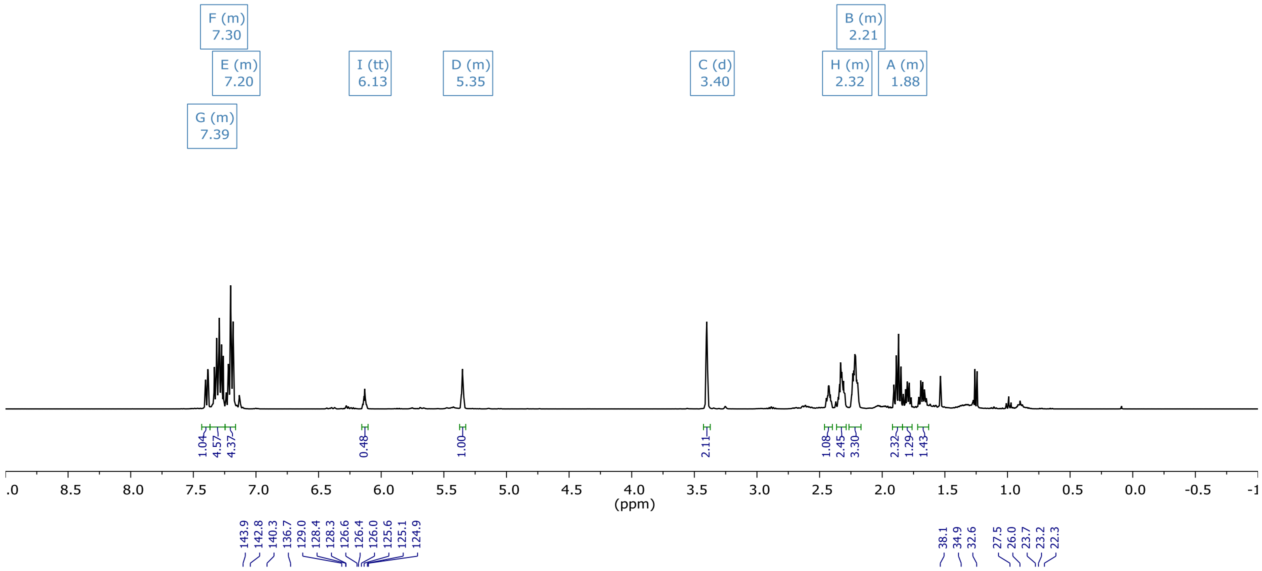
F (m)
7.30
E (m)
7.20
G (m)
7.39
I (tt)
6.13
D (m)
5.35
C (d)
3.40
B (m)
2.21
H (m)
2.32
A (m)
1.88
1.04
4.57
4.37
0.48
1.00
2.11
1.08
2.45
3.30
2.32
1.29
1.43
(ppm)
143.9
142.8
140.3
136.7
129.0
128.4
128.3
126.6
126.4
126.0
125.6
125.1
124.9
38.1
34.9
32.6
27.5
26.0
23.7
23.2
22.3

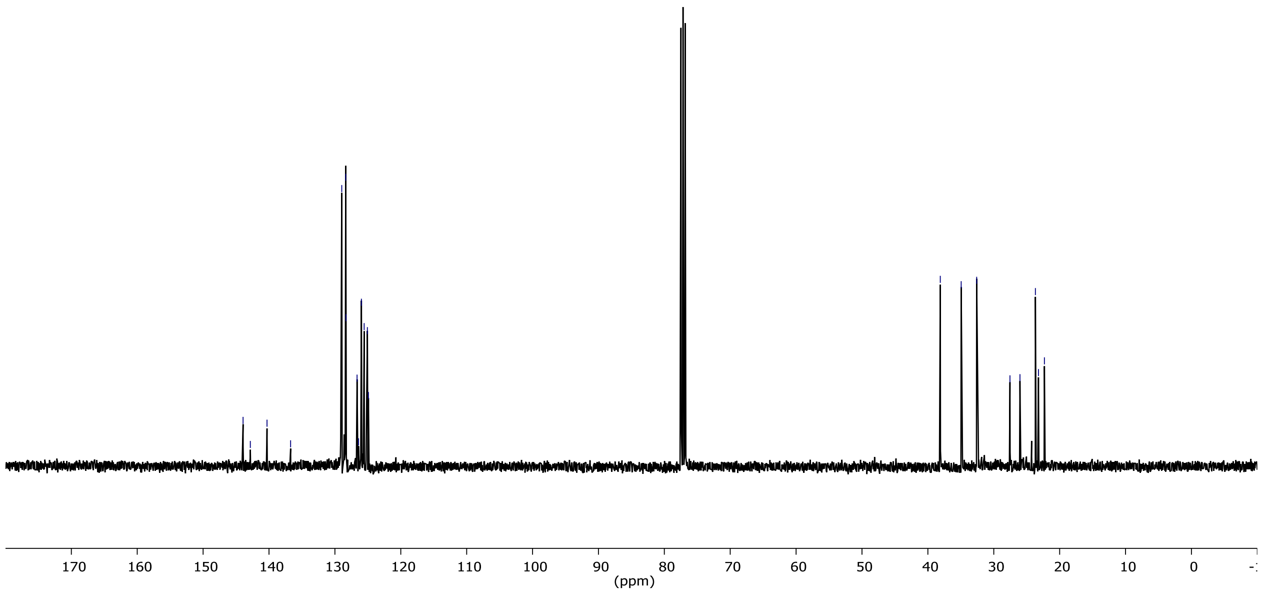
(ppm)

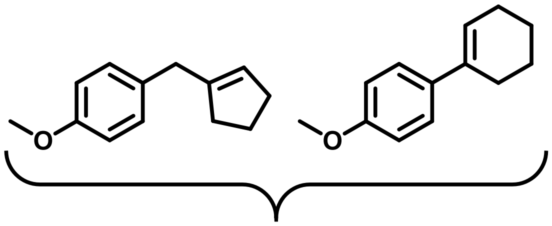

2:1

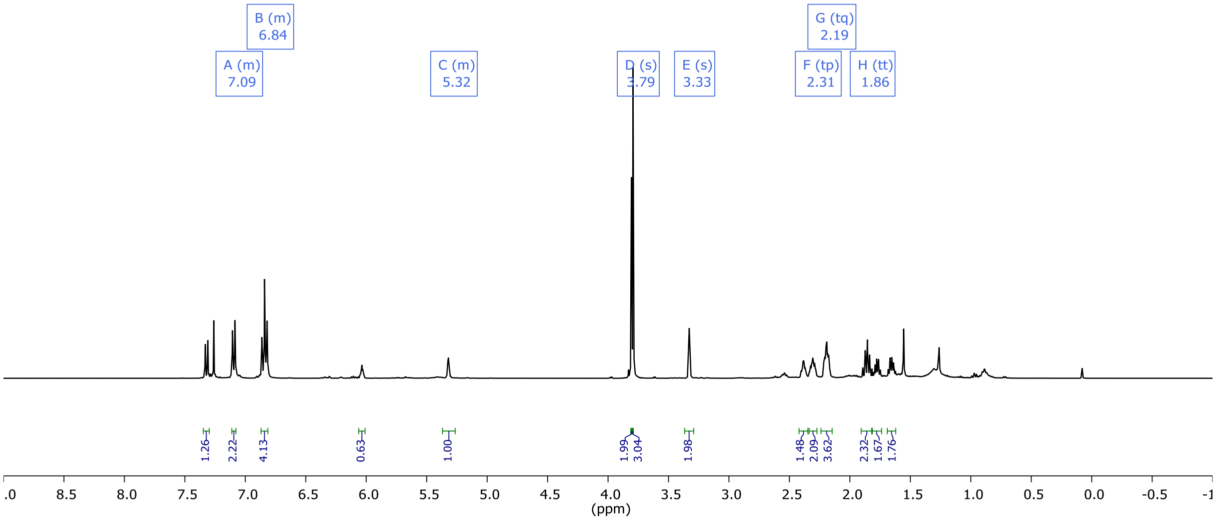
B (m)
6.84
A (m)
7.09
C (m)
5.32
D (s)
3.79
E (s)
3.33
G (tq)
2.19
F (tp)
2.31
H (tt)
1.86
1.26
2.22
4.13
0.63
1.00
1.99
3.04
1.98
1.48
2.09
3.62
2.32
1.67
1.76
(ppm)

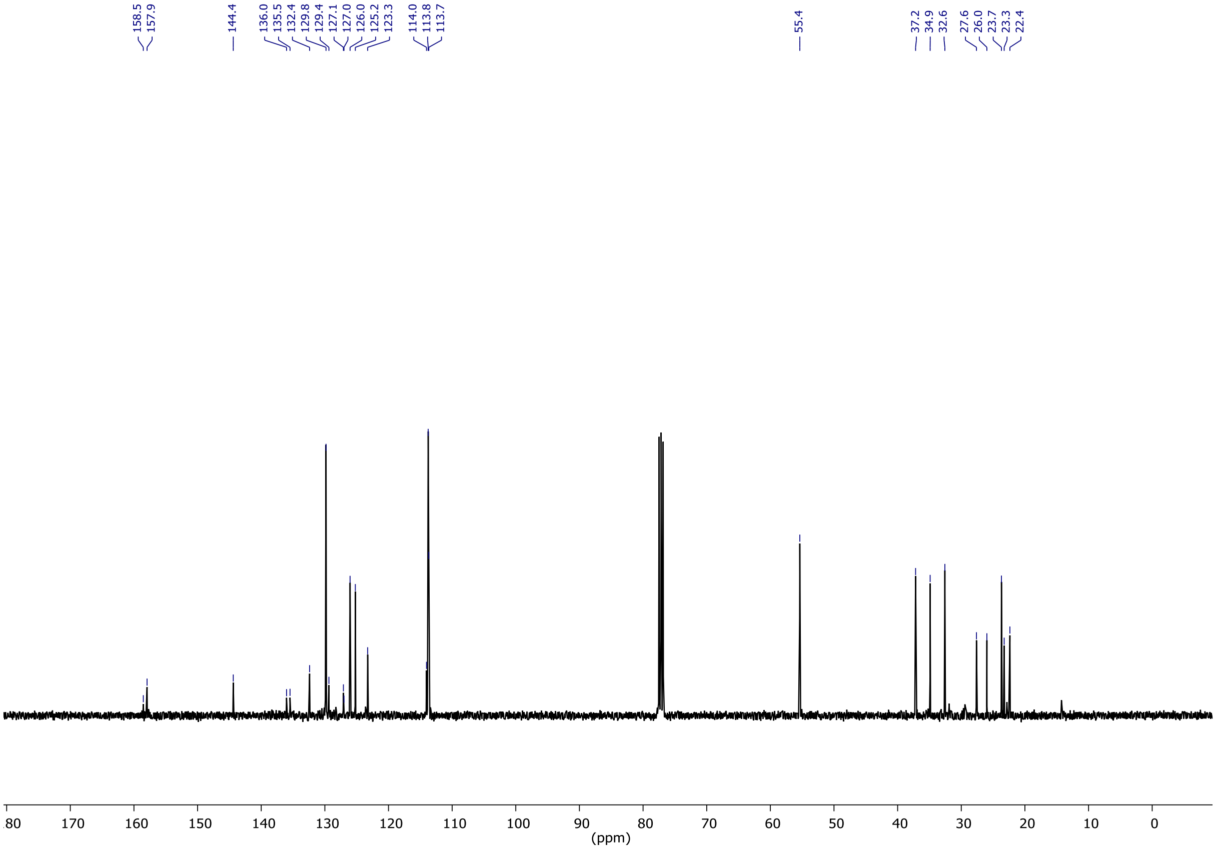
158.5
157.9
144.4
136.0
135.5
132.4
129.8
129.4
127.1
127.0
126.0
125.2
123.3
114.0
113.8
113.7
55.4
37.2
34.9
32.6
27.6
26.0
23.7
23.3
22.4
(ppm)

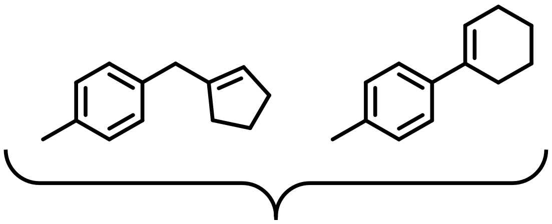

2:1

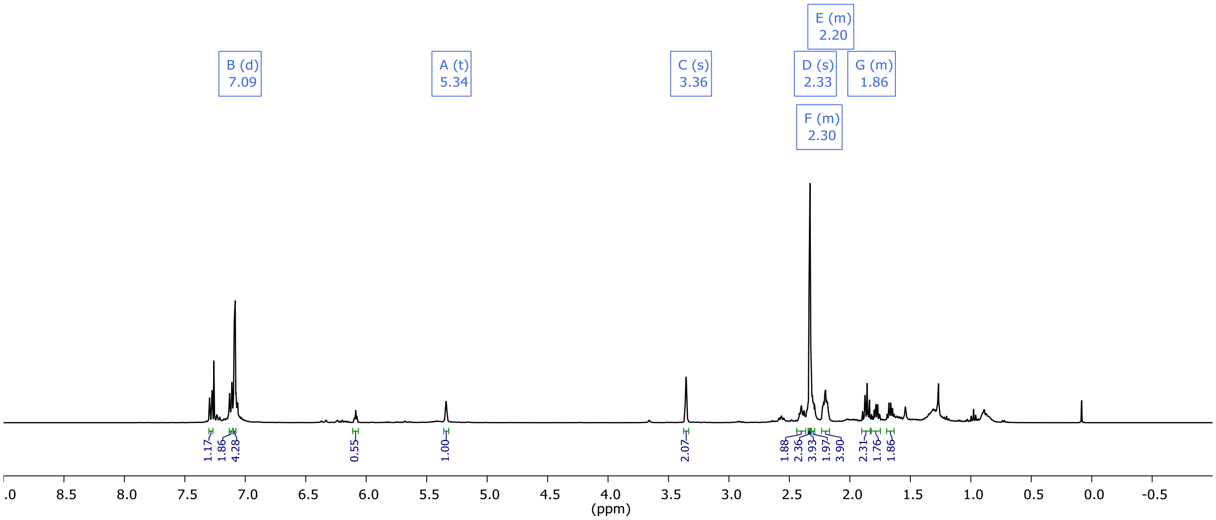
B (d)
7.09
A (t)
5.34
C (s)
3.36
E (m)
2.20
D (s)
2.33
G (m)
1.86
F (m)
2.30
1.17
1.86
4.28
0.55
1.00
2.07
1.88
2.36
3.93
1.97
3.90
2.31
1.76
1.86
(ppm)

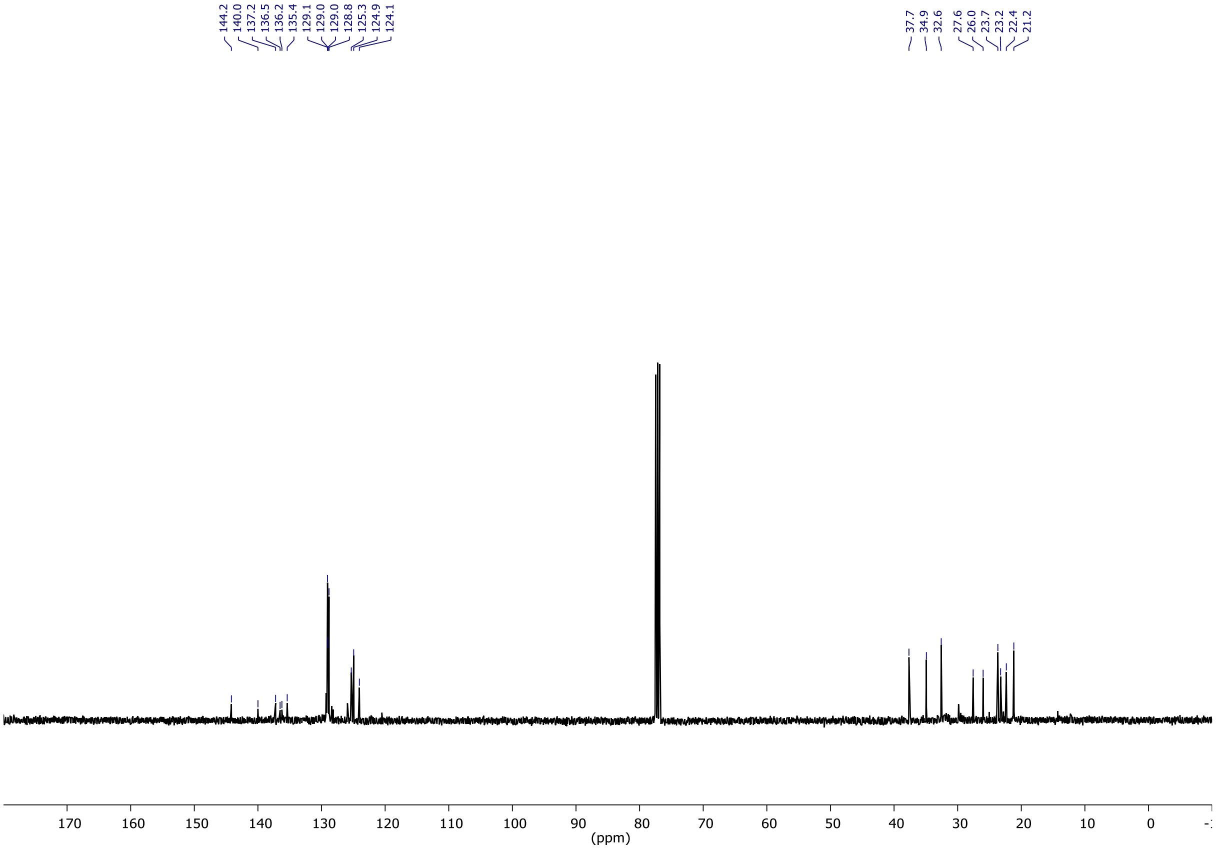
144.2
140.0
137.2
136.5
136.2
135.4
129.1
129.0
129.0
128.8
125.3
124.9
124.1
37.7
34.9
32.6
27.6
26.0
23.7
23.2
22.4
21.2
(ppm)

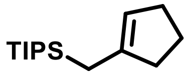
TIPS

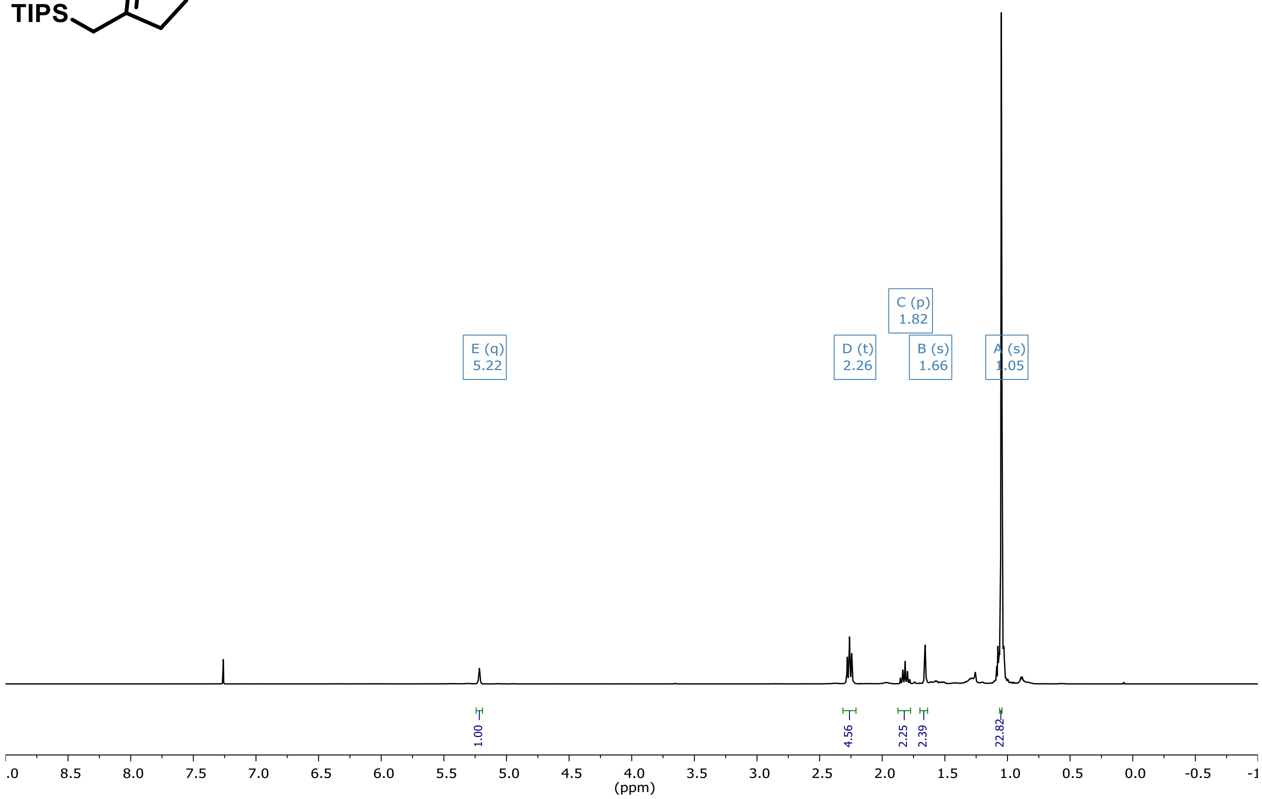
E (q)
5.22
D (t)
2.26
C (p)
1.82
B (s)
1.66
A (s)
1.05
1.00
4.56
2.25
2.39
22.82
(ppm)

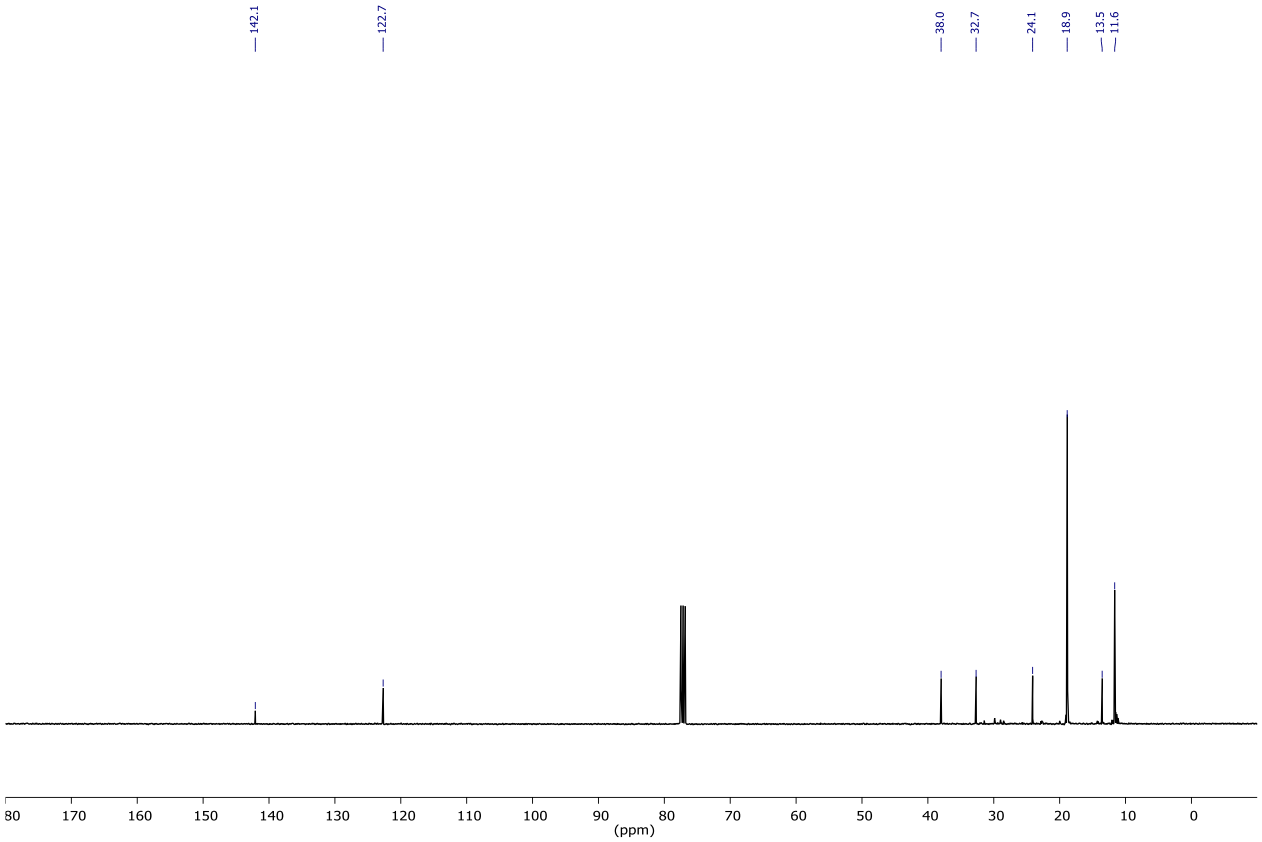
142.1
122.7
38.0
32.7
24.1
18.9
13.5
11.6
(ppm)

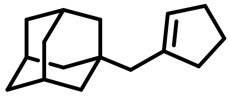

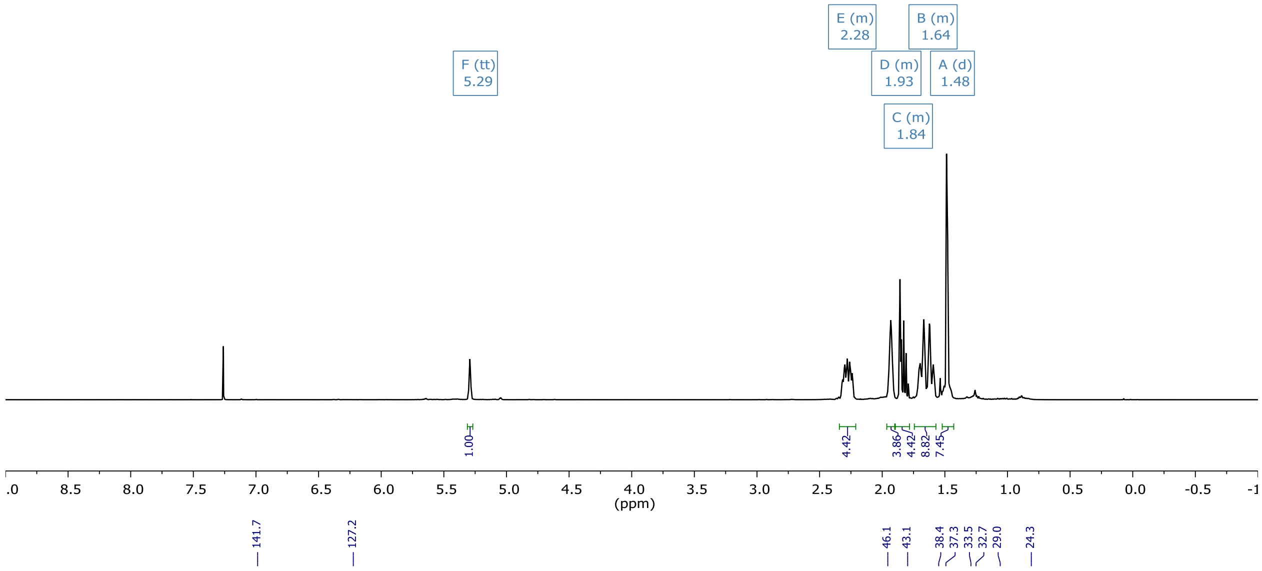

E (m)
2.28
B (m)
1.64
F (tt)
5.29
D (m)
1.93
A (d)
1.48
C (m)
1.84
1.00
4.42
3.86
4.42
8.82
7.45
(ppm)

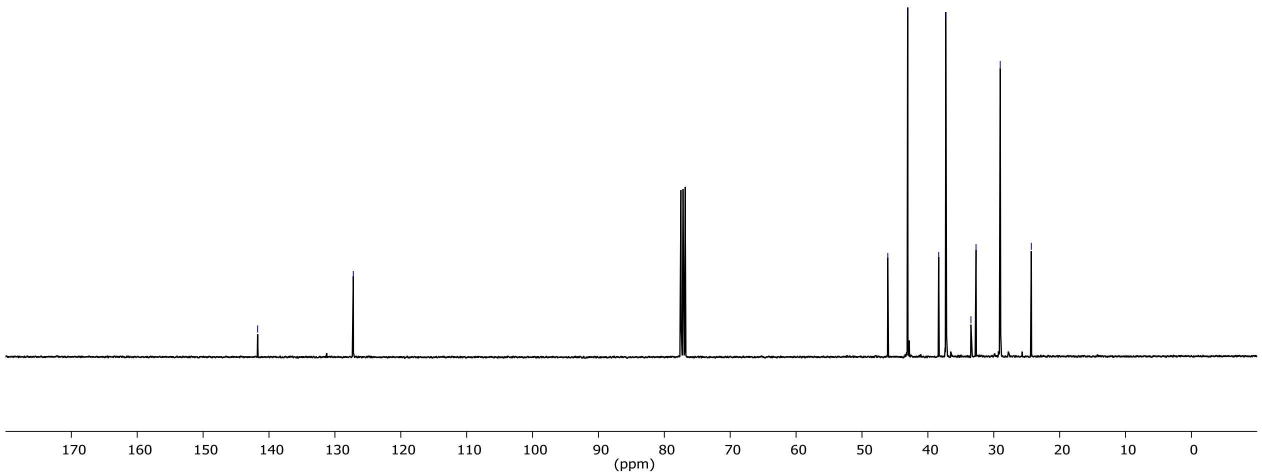

141.7
127.2
46.1
43.1
38.4
37.3
33.5
32.7
29.0
24.3
(ppm)

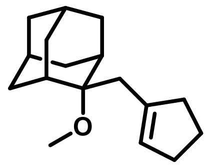

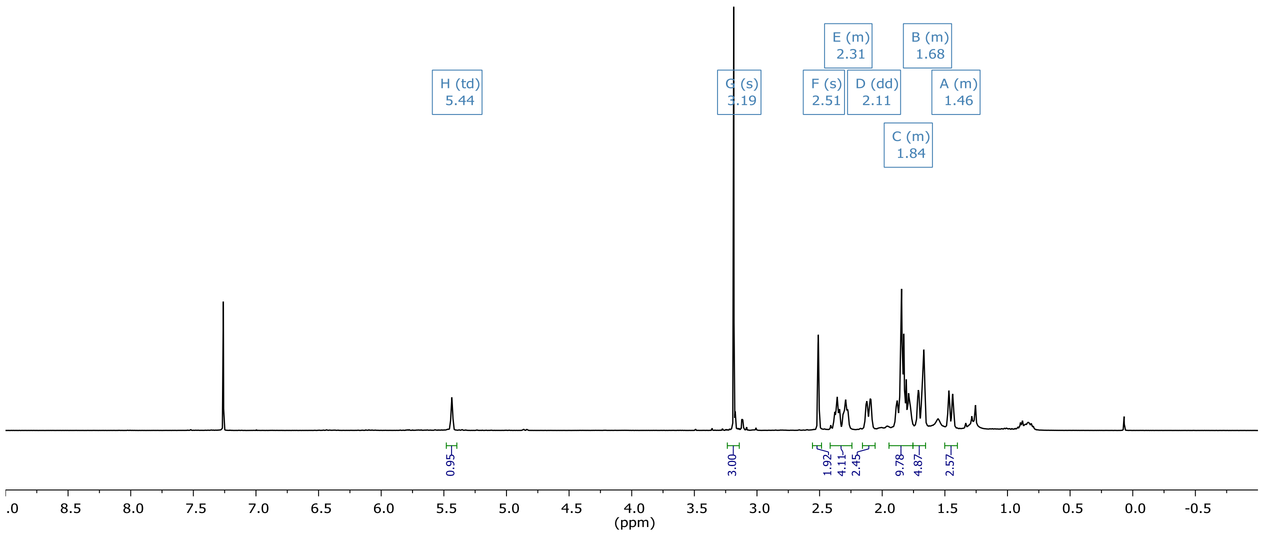
H (td)
5.44
G (s)
3.19
F (s)
2.51
E (m)
2.31
D (dd)
2.11
C (m)
1.84
B (m)
1.68
A (m)
1.46
0.95
3.00
1.92
4.11
2.45
9.78
4.87
2.57
(ppm)

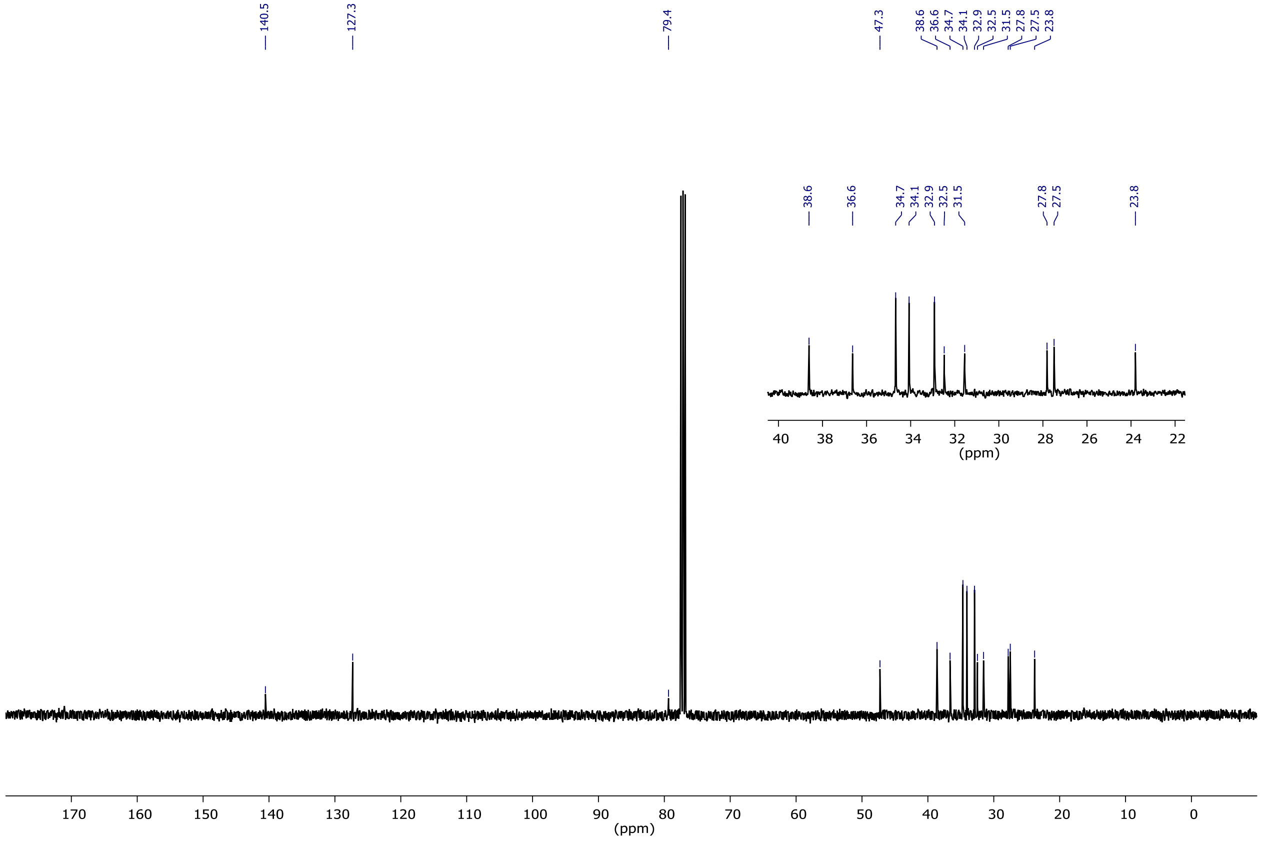
140.5
127.3
79.4
47.3
38.6
36.6
34.7
34.1
32.9
32.5
31.5
27.8
27.5
23.8
(ppm)

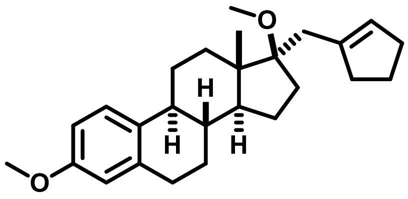

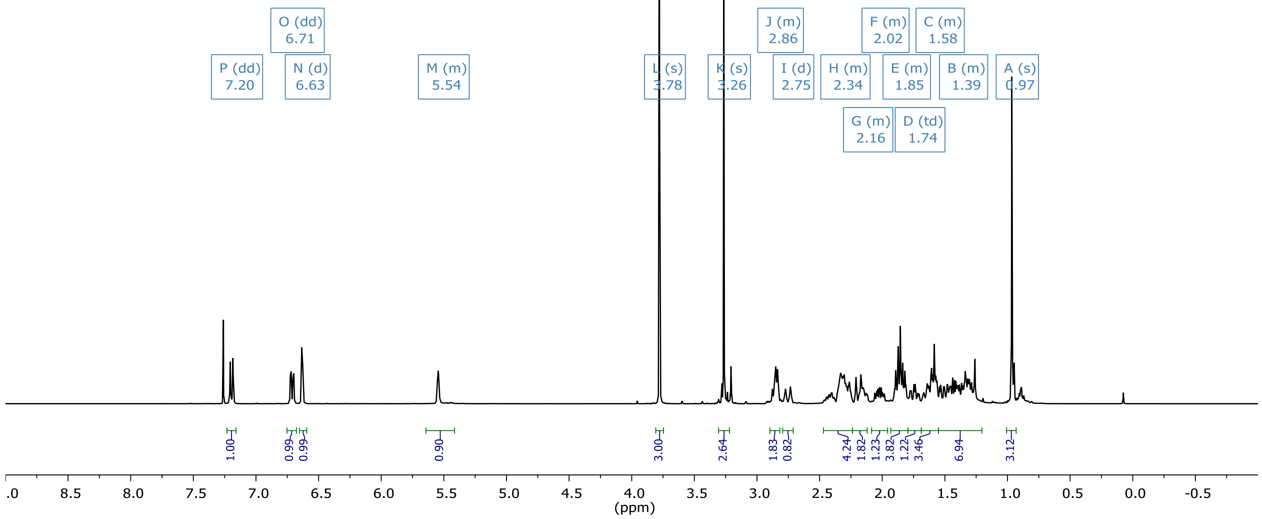
O (dd)
6.71
P (dd)
7.20
N (d)
6.63
M (m)
5.54
L (s)
3.78
K (s)
3.26
J (m)
2.86
I (d)
2.75
H (m)
2.34
G (m)
2.16
F (m)
2.02
E (m)
1.85
D (td)
1.74
C (m)
1.58
B (m)
1.39
A (s)
0.97
1.00
0.99
0.99
0.90
3.00
2.64
1.83
0.82
4.24
1.82
1.23
3.82
1.22
3.46
6.94
3.12
(ppm)

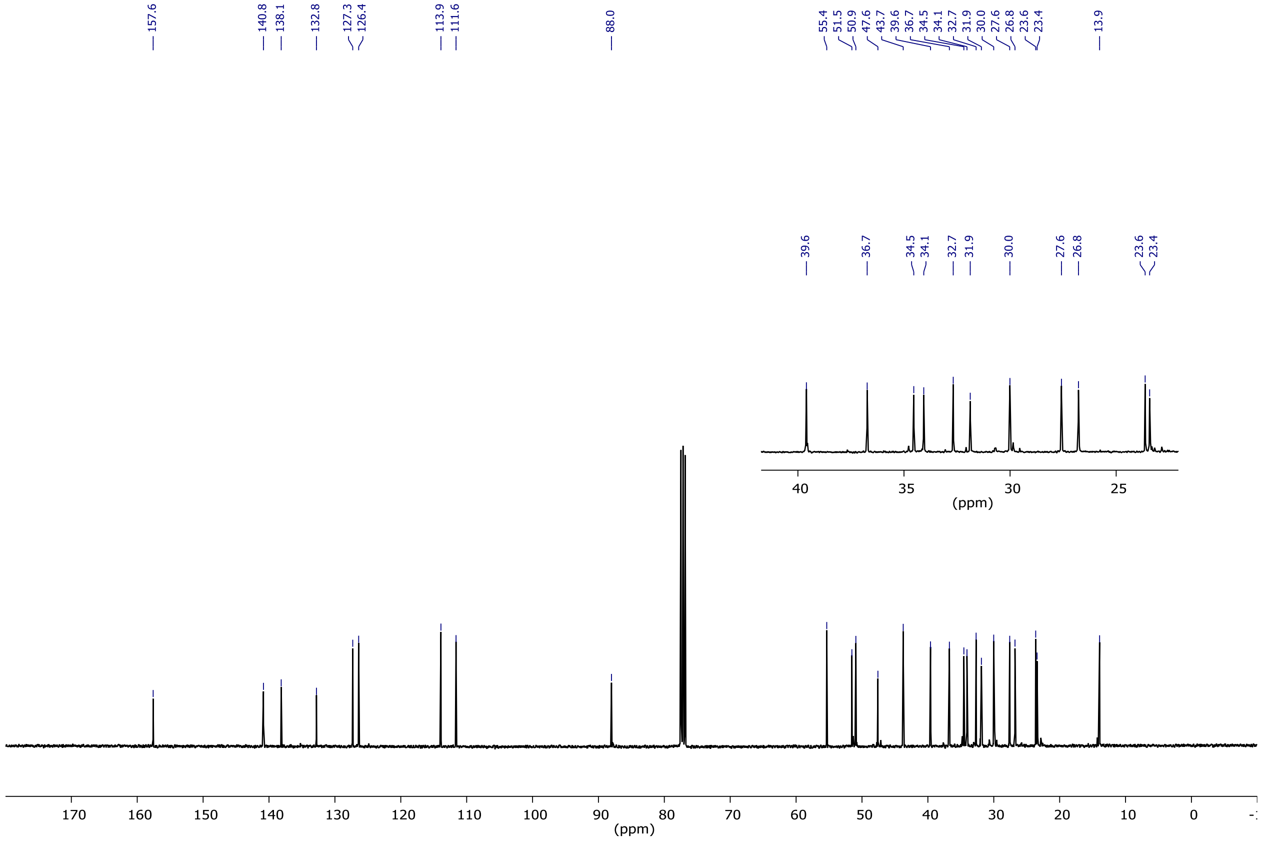
157.6
140.8
138.1
132.8
127.3
126.4
113.9
111.6
88.0
55.4
51.5
50.9
47.6
43.7
39.6
36.7
34.5
34.1
32.7
31.9
30.0
27.6
26.8
23.6
23.4
13.9
(ppm)

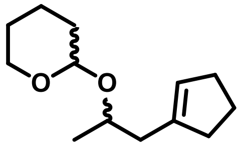

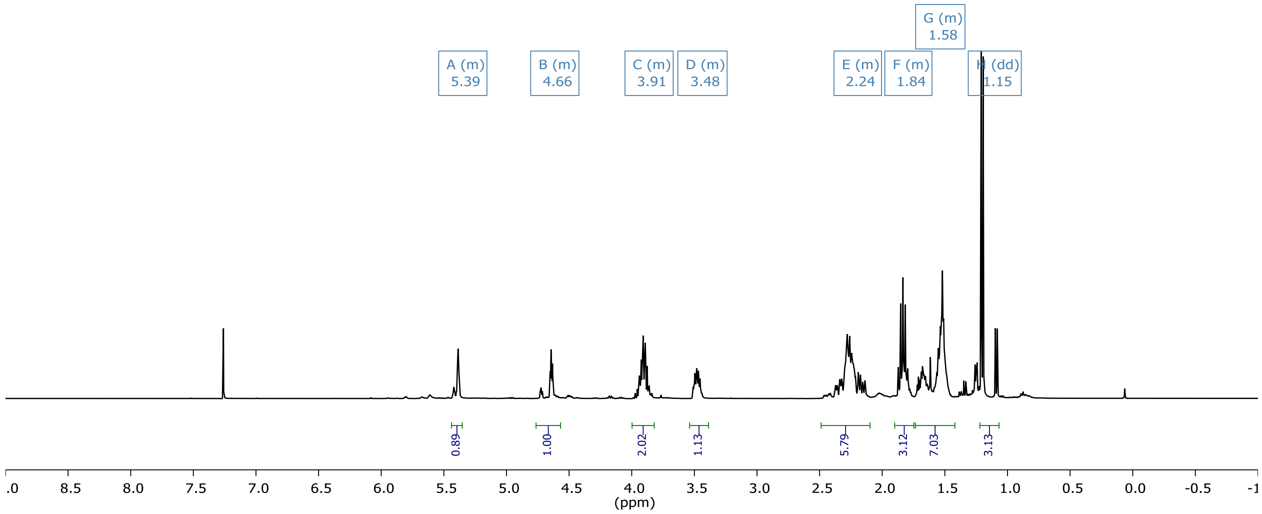
A (m)
5.39
B (m)
4.66
C (m)
3.91
D (m)
3.48
E (m)
2.24
F (m)
1.84
G (m)
1.58
H (dd)
1.15
0.89
1.00
2.02
1.13
5.79
3.12
7.03
3.13
(ppm)

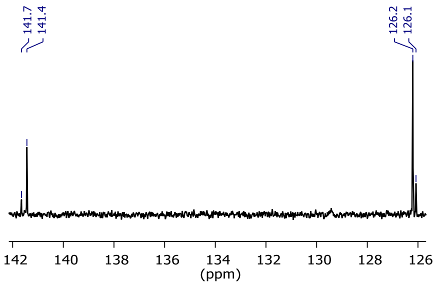
141.7
141.4
126.2
126.1
(ppm)

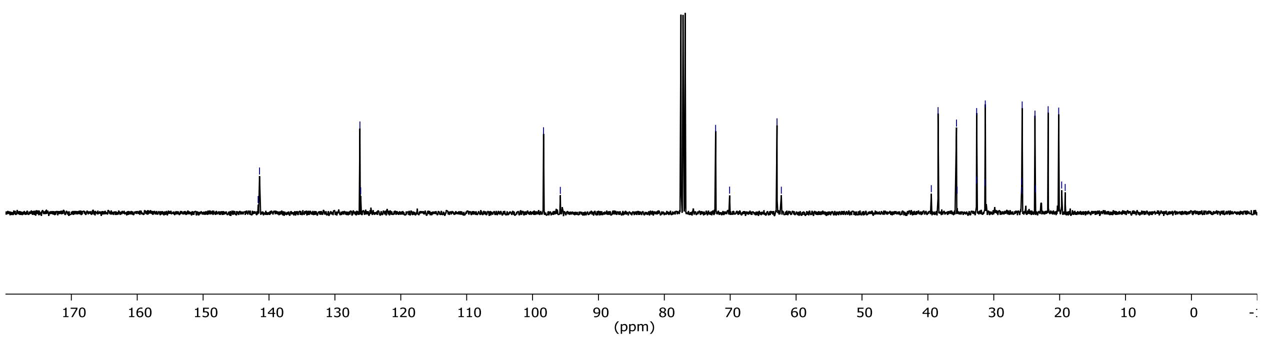
141.7
141.4
126.2
126.1
98.3
95.8
72.2
70.1
62.9
62.2
39.5
38.5
35.7
35.6
32.6
32.6
31.3
31.3
25.8
25.7
25.7
23.7
21.7
20.1
19.7
19.2
(ppm)

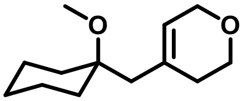

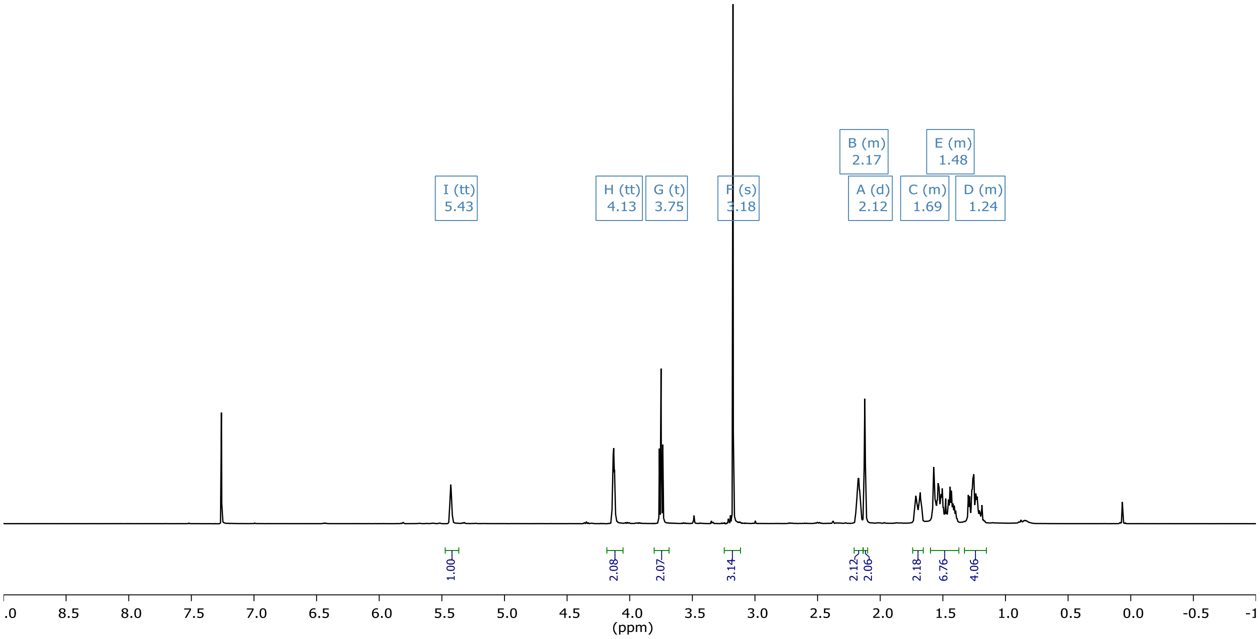
I (tt)
5.43
H (tt)
4.13
G (t)
3.75
F (s)
3.18
B (m)
2.17
A (d)
2.12
C (m)
1.69
E (m)
1.48
D (m)
1.24
1.00
2.08
2.07
3.14
2.12
2.06
2.18
6.76
4.06
(ppm)

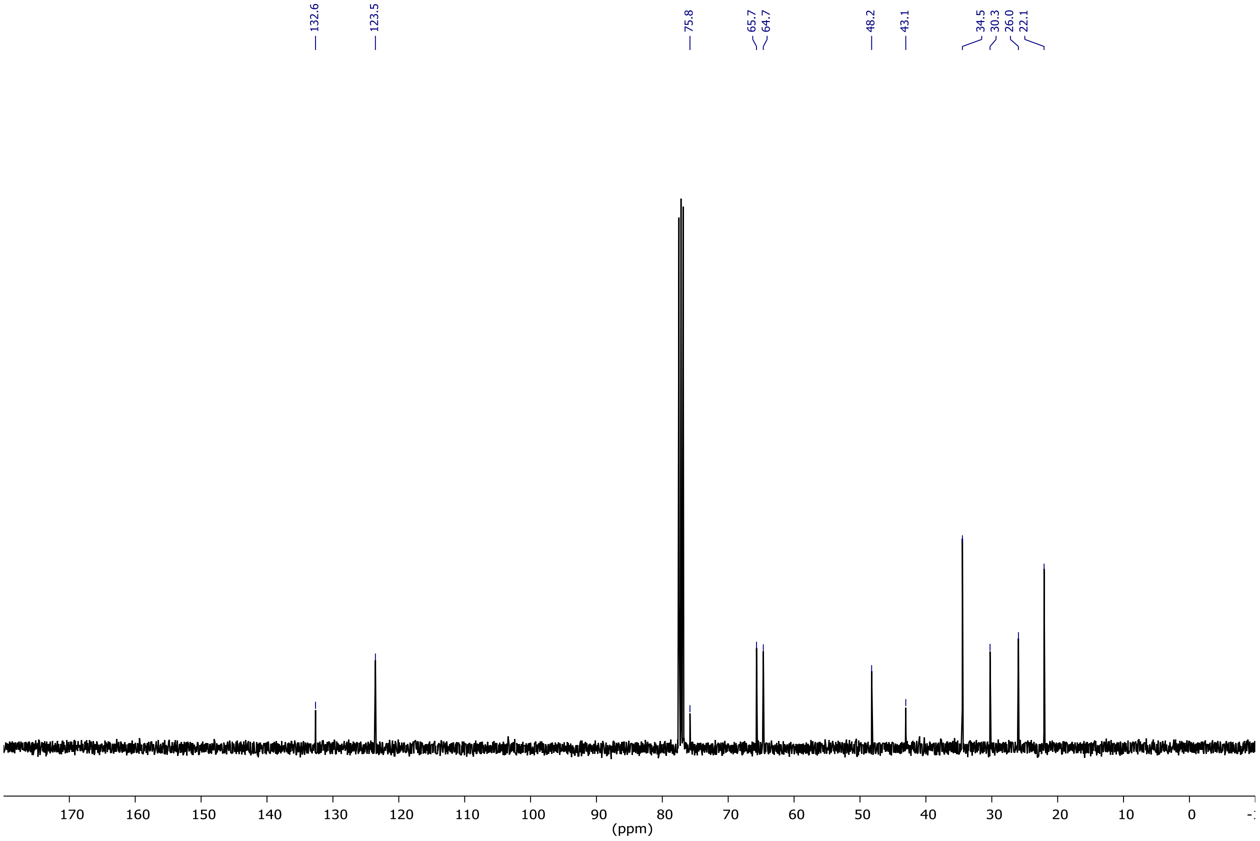
132.6
123.5
75.8
65.7
64.7
48.2
43.1
34.5
30.3
26.0
22.1
(ppm)

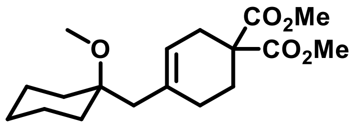
CO2Me
CO2Me
O

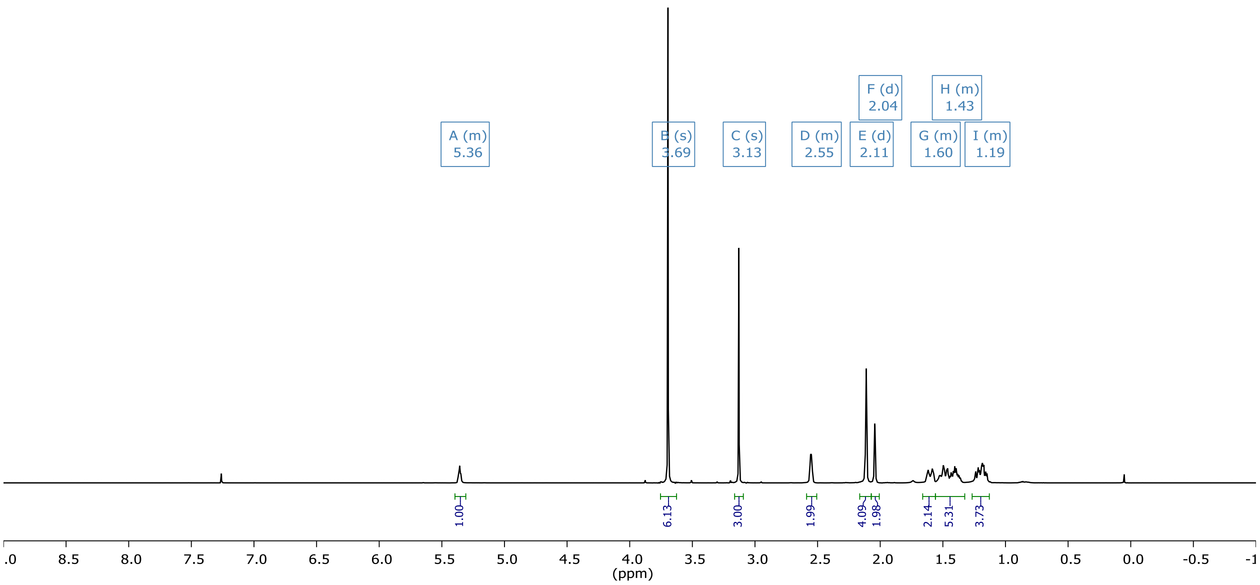
A (m) 5.36
B (s) 3.69
C (s) 3.13
D (m) 2.55
E (d) 2.11
F (d) 2.04
G (m) 1.60
H (m) 1.43
I (m) 1.19
1.00
6.13
3.00
1.99
4.09
1.98
2.14
5.31
3.73
(ppm)

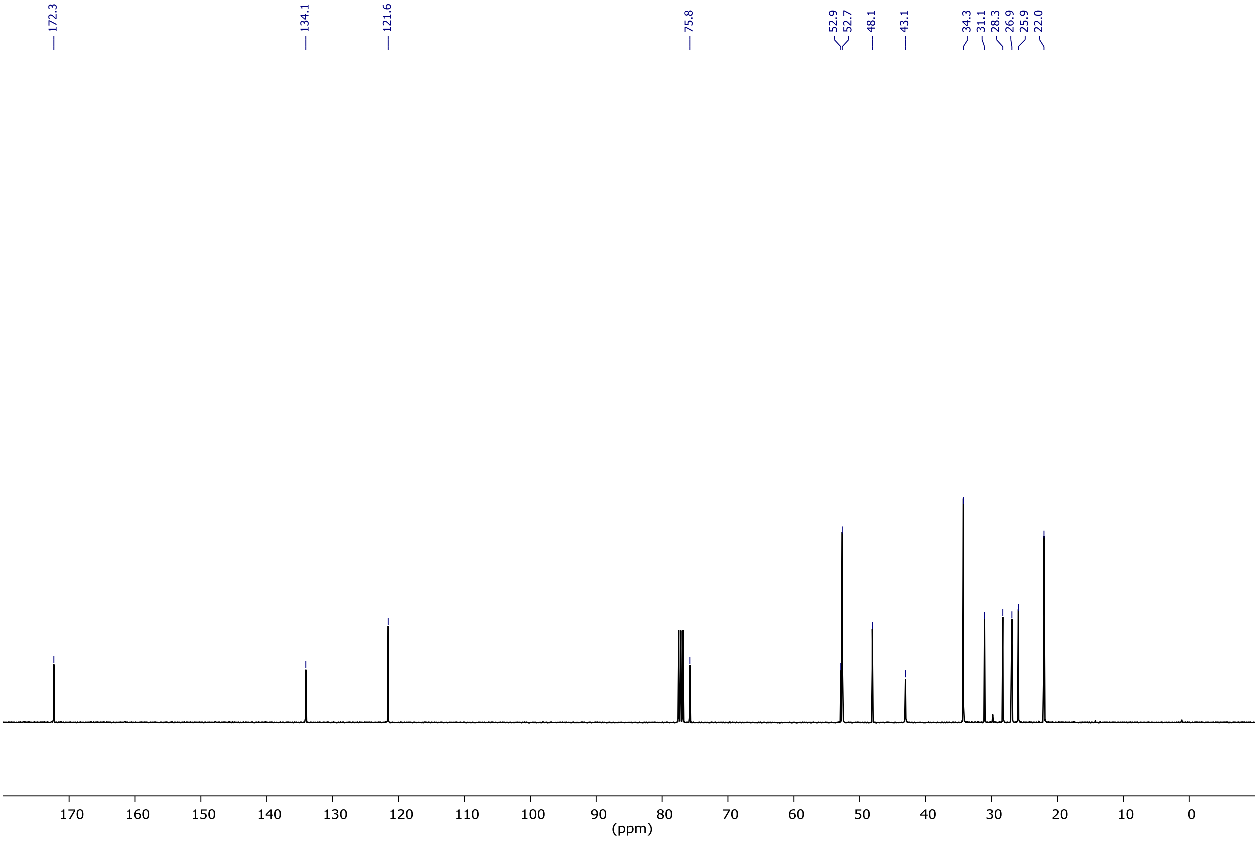
172.3
134.1
121.6
75.8
52.9
52.7
48.1
43.1
34.3
31.1
28.3
26.9
25.9
22.0
(ppm)

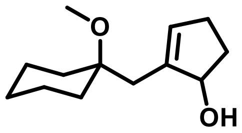
O
OH

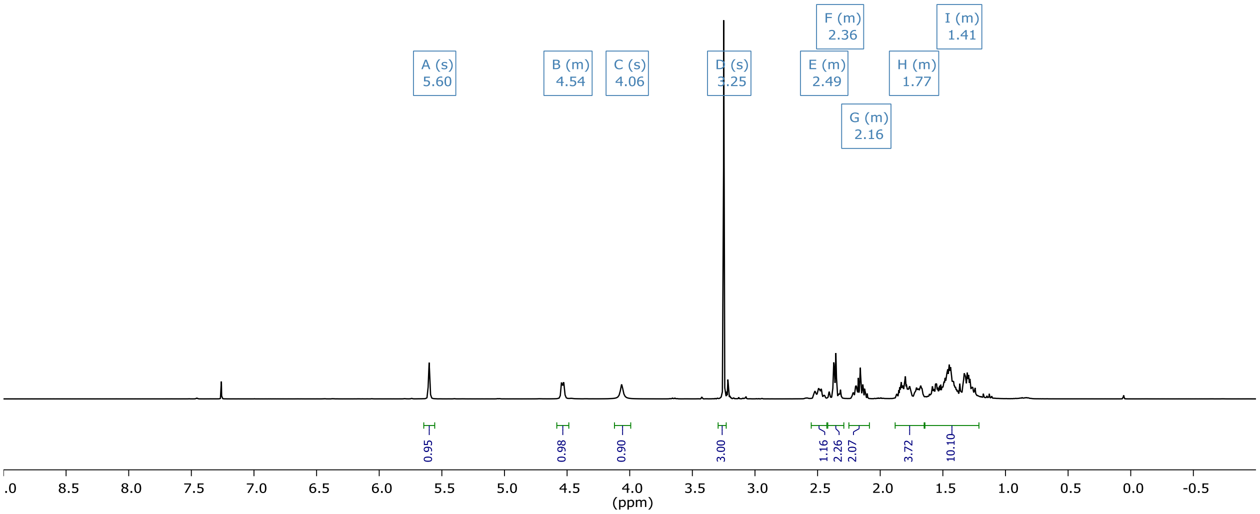
A (s)
5.60
B (m)
4.54
C (s)
4.06
D (s)
3.25
E (m)
2.49
F (m)
2.36
G (m)
2.16
H (m)
1.77
I (m)
1.41
0.95
0.98
0.90
3.00
1.16
2.26
2.07
3.72
10.10
(ppm)

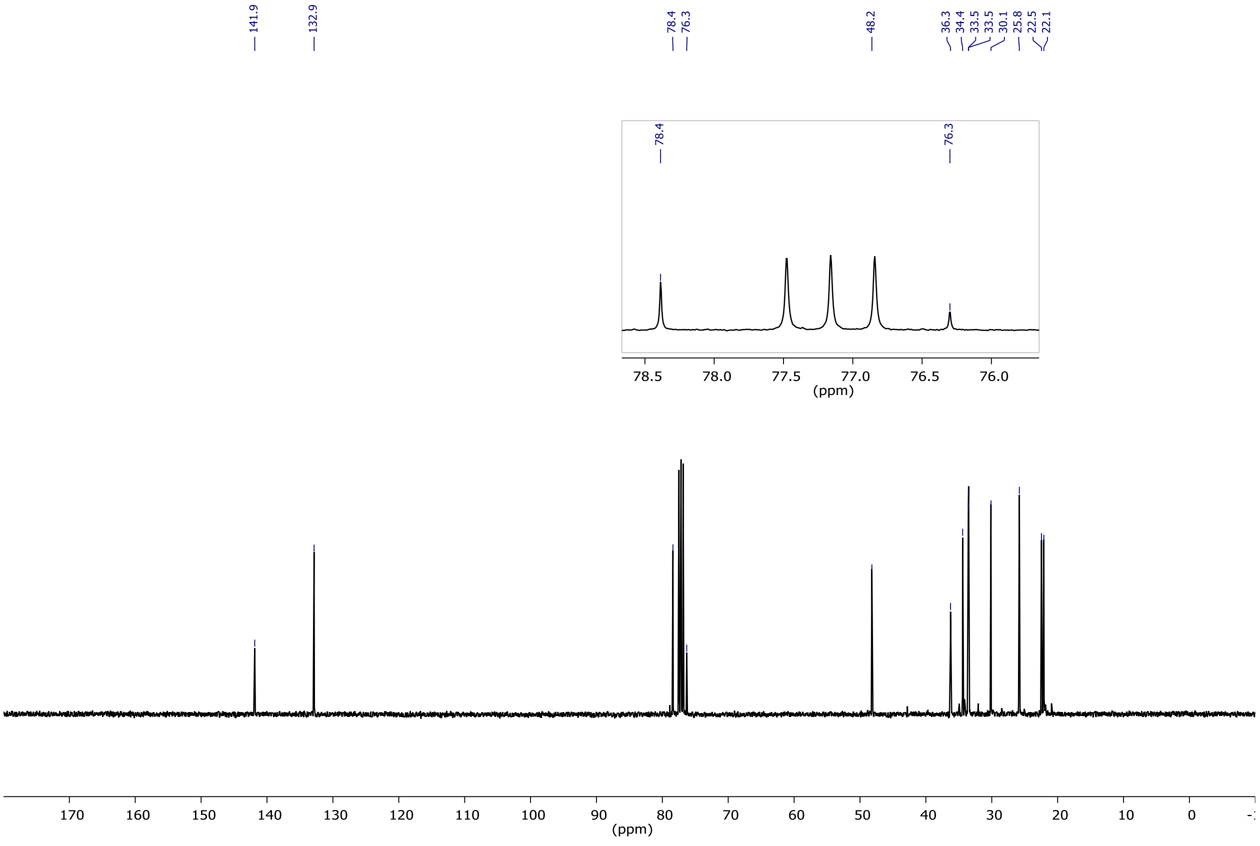
141.9
132.9
78.4
76.3
48.2
36.3
34.4
33.5
30.1
25.8
22.5
22.1
(ppm)

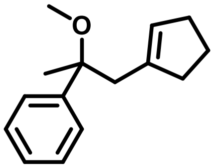

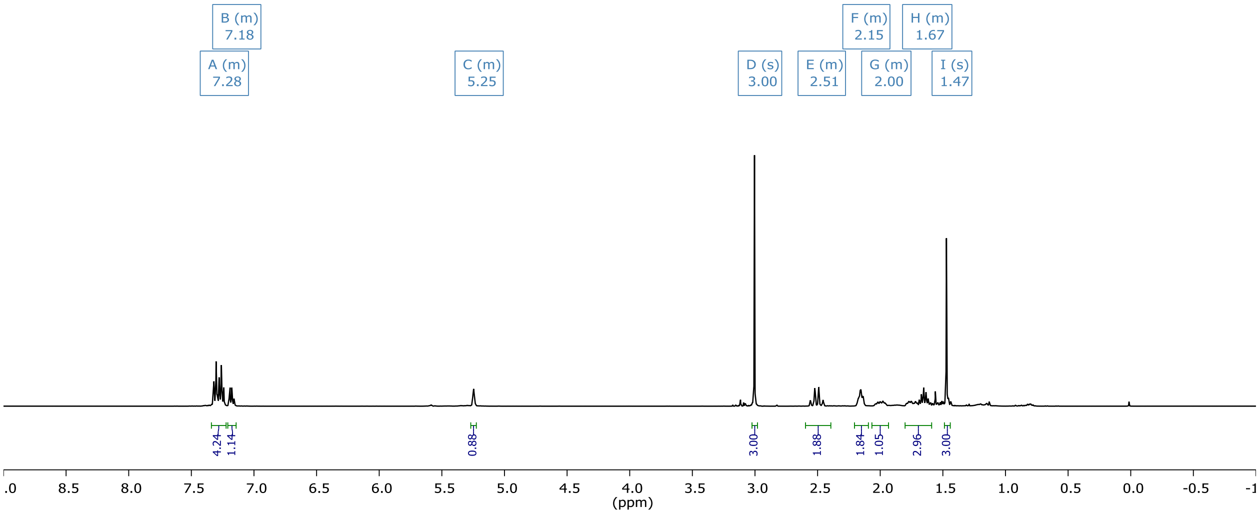
B (m)
7.18
A (m)
7.28
C (m)
5.25
D (s)
3.00
E (m)
2.51
F (m)
2.15
G (m)
2.00
H (m)
1.67
I (s)
1.47
4.24
1.14
0.88
3.00
1.88
1.84
1.05
2.96
3.00
(ppm)

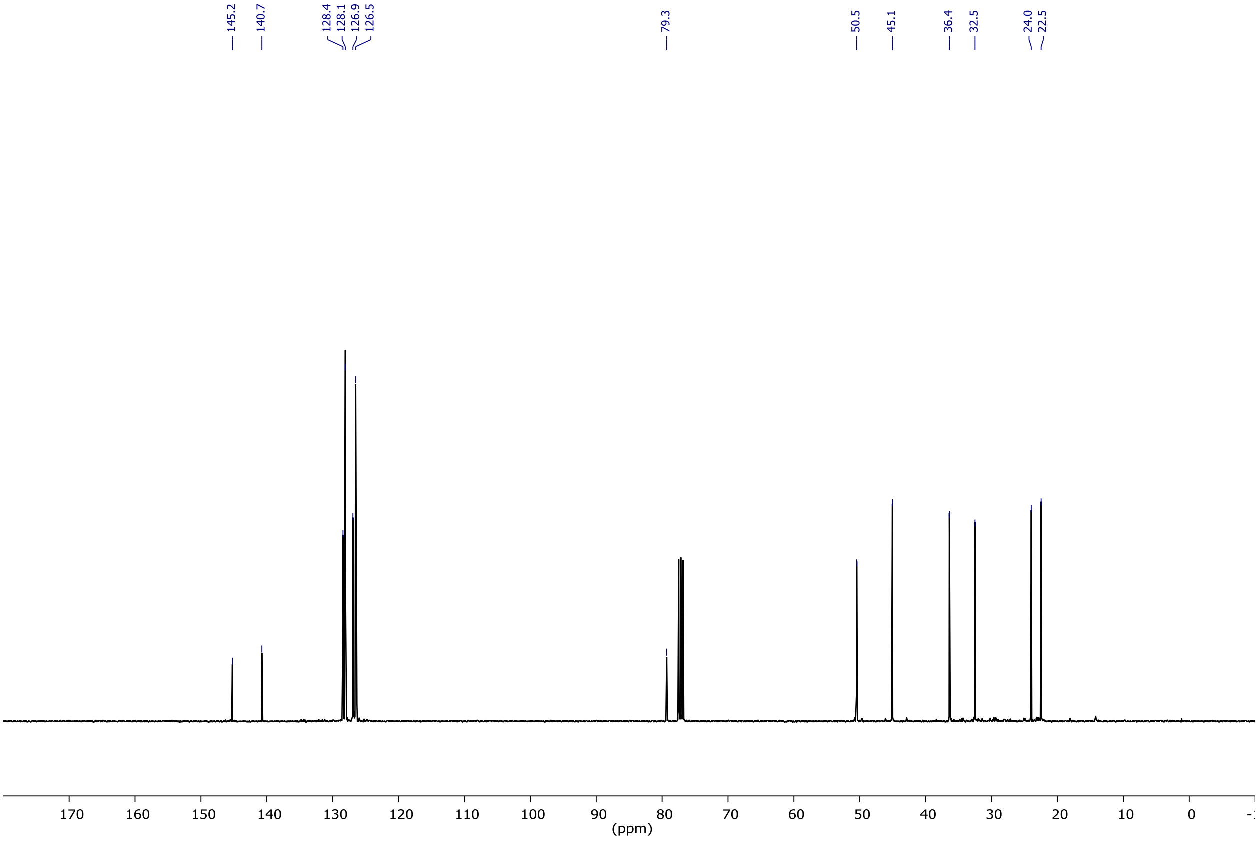
145.2
140.7
128.4
128.1
126.9
126.5
79.3
50.5
45.1
36.4
32.5
24.0
22.5
(ppm)

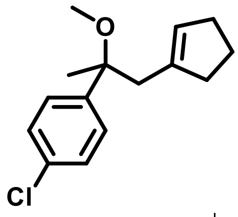
O
Cl

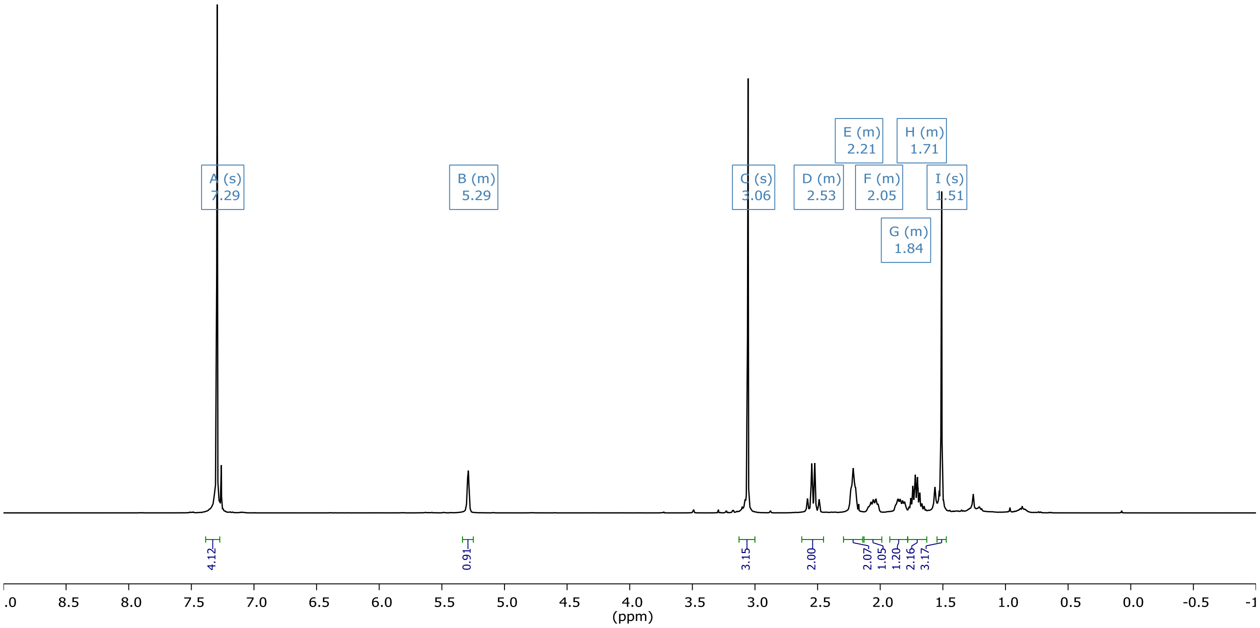
A (s)
7.29
B (m)
5.29
C (s)
3.06
D (m)
2.53
E (m)
2.21
F (m)
2.05
G (m)
1.84
H (m)
1.71
I (s)
1.51
4.12
0.91
3.15
2.00
2.07
1.05
1.20
2.16
3.17
8.5
8.0
7.5
7.0
6.5
6.0
5.5
5.0
4.5
4.0
3.5
3.0
2.5
2.0
1.5
1.0
0.5
0.0
-0.5
(ppm)

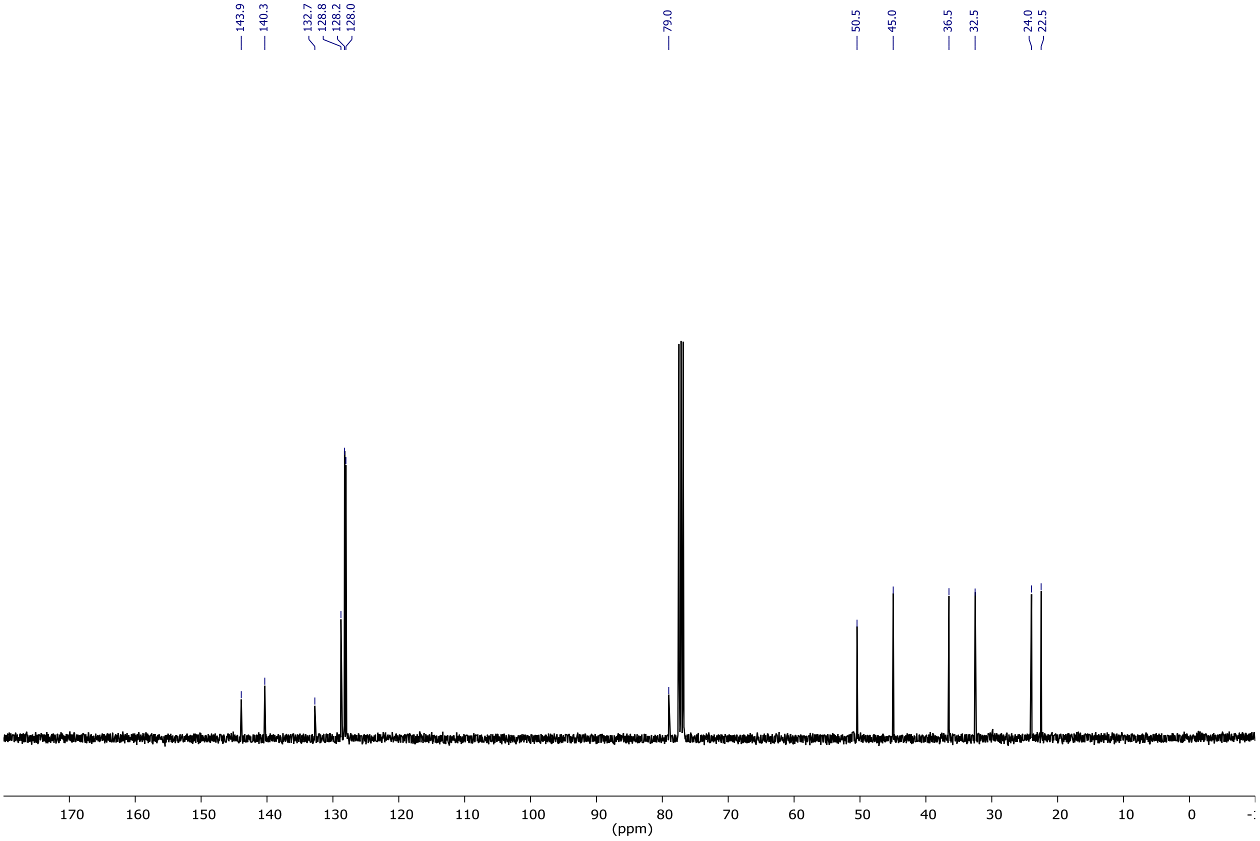
143.9
140.3
132.7
128.8
128.2
128.0
79.0
50.5
45.0
36.5
32.5
24.0
22.5
170
160
150
140
130
120
110
100
90
80
70
60
50
40
30
20
10
0
(ppm)

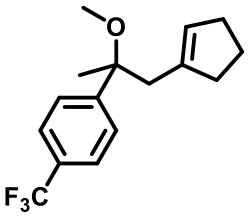
F3C
O

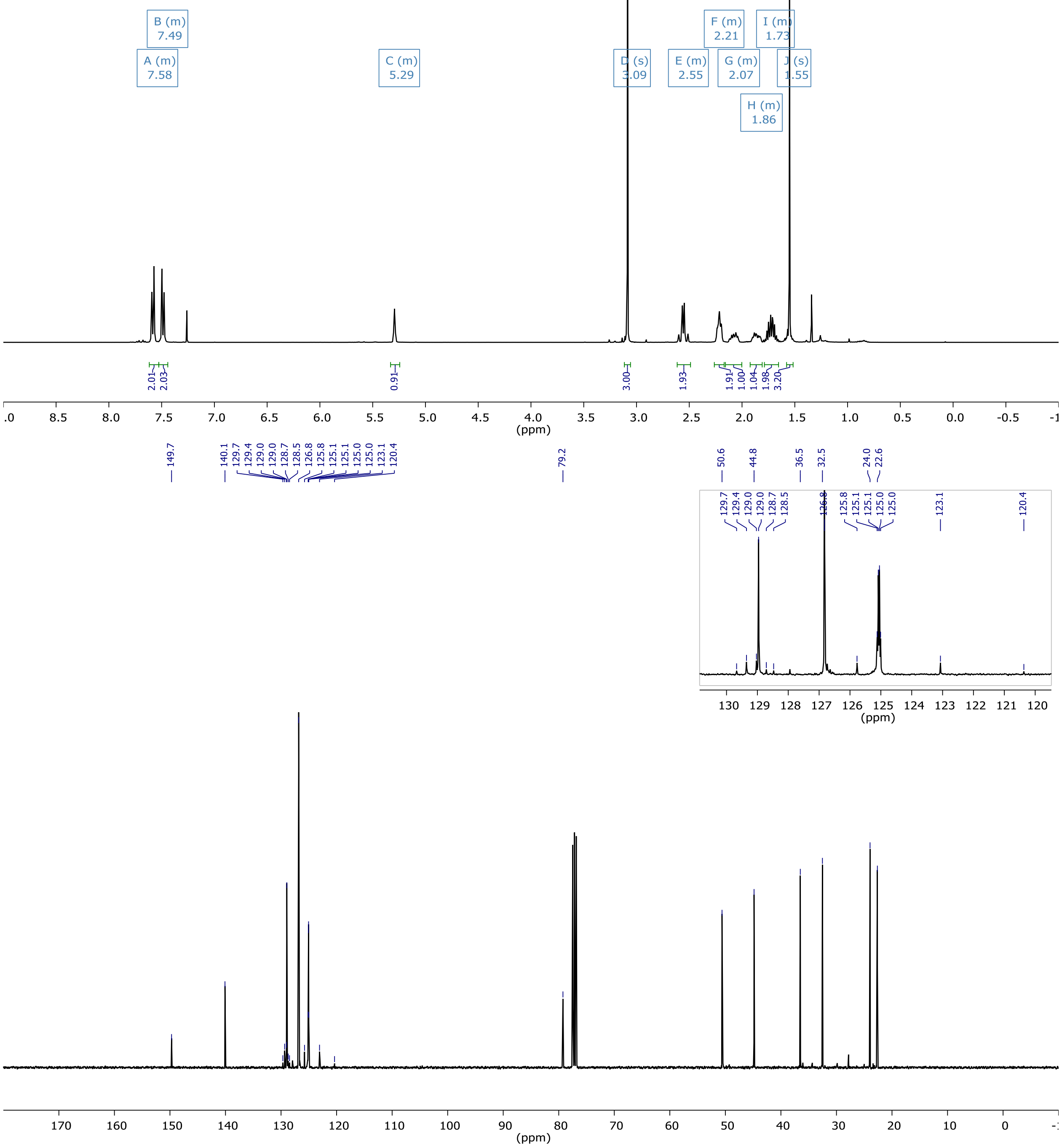
A (m) 7.58
B (m) 7.49
C (m) 5.29
D (s) 3.09
E (m) 2.55
F (m) 2.21
G (m) 2.07
H (m) 1.86
I (m) 1.73
J (s) 1.55
(ppm)
149.7
140.1
79.2
50.6
44.8
36.5
32.5
24.0
22.6
(ppm)

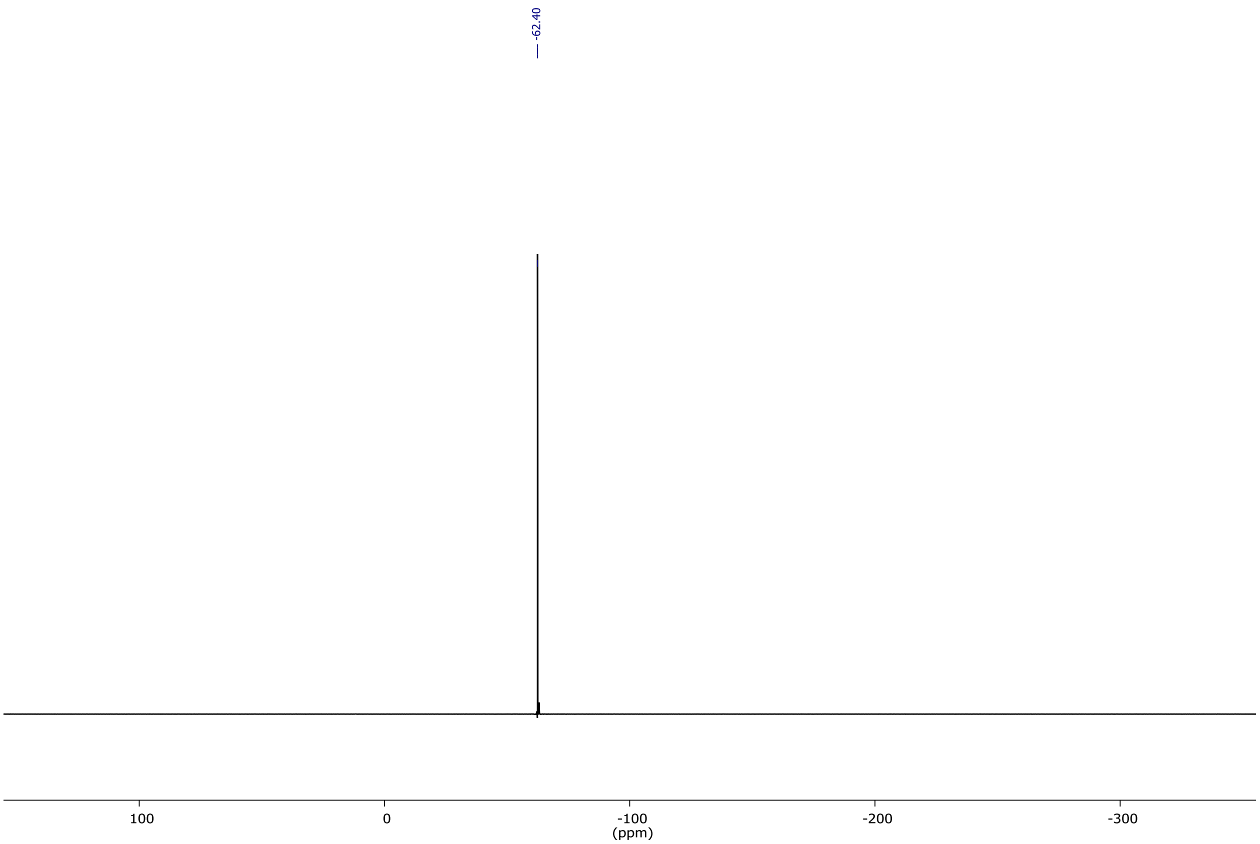
-62.40
100
0
-100
(ppm)
-200
-300

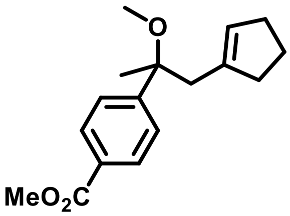

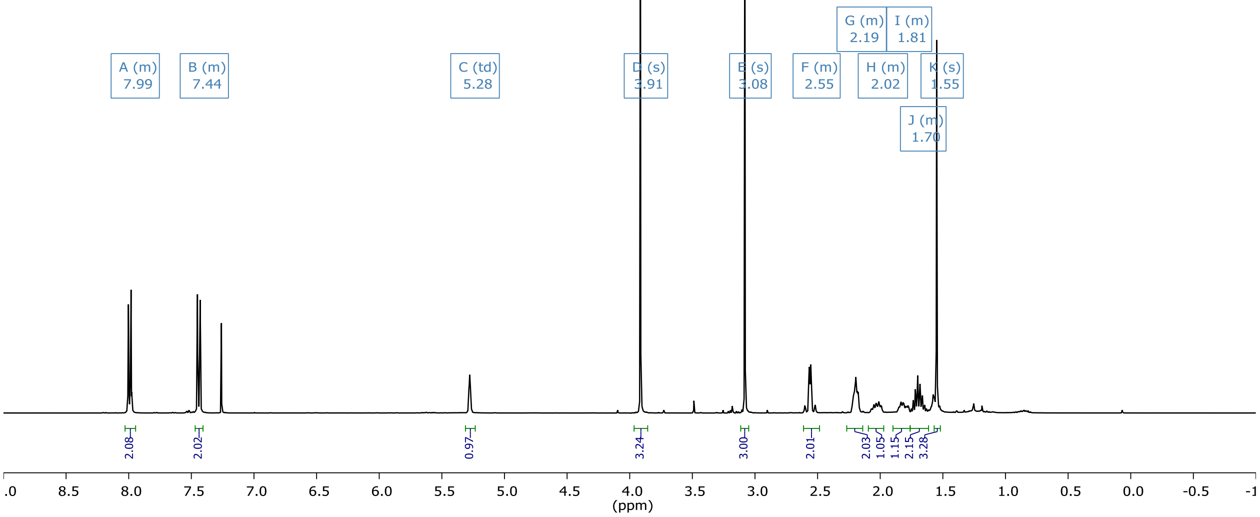

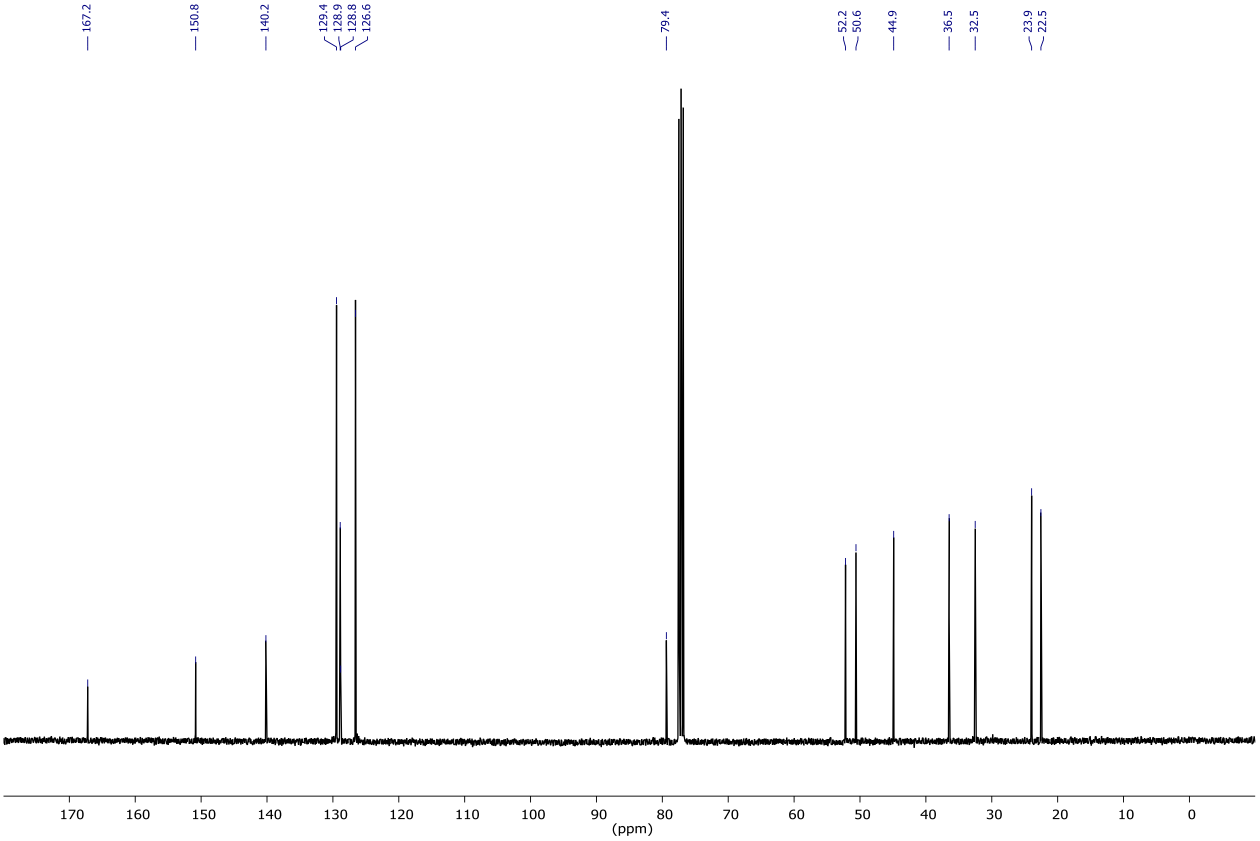

6. References

[1] G. R. Fulmer, A. J. M. Miller, N. H. Sherden, H. E. Gottlieb, A. Nudelman, B. M. Stoltz, J. E. Bercaw, K. I. Goldberg, *Organometallics* **2010**, *29*, 2176–2179

[2] A. Fürstner, P. W. Davies, C. W. Lehmann, *Organometallics* **2005**, *24*, 4065-4071.

[3] T. Ritter, A. Hejl, A. G. Wenzel, T. W. Funk, R. H. Grubbs, *Organometallics* **2006**, *25*, 5740-5745.

[4] J. Engel, W. Smit, M. Foscato, G. Occhipinti, K. W. Törnroos, V. R. Jensen, *J. Am. Chem. Soc* **2017**, *139*, 16609-16619.

[5] G. Bailey, M. Foscato, C. S. Higman, C. S. Day, V. R. Jensen, D. E. Fogg. *J. Am. Chem. Soc.* **2018**, *140*, 6931-6944.

[6] A. Guthertz, M. Leutzsch, L. M. Wolf, P. Gupta, S. M. Rummelt, R. Goddard, C. Farès, W. Thiel, A. Fürstner, *J. Am. Chem. Soc.* **2018**, *140*, 3156-3169.

[7] The procedure follows the preparation of an analogous complex carrying a saturated H_2IMes ligand described in ref. 5

[8] S. Barlow, J.-L. Brédas, Y. A. Getmanenko, R. L. Gieseking, J. M. Hales, H. Kim, S. R. Marder, J. W. Perry, C. Risko, Y. Zhang, *Mater. Horiz.* **2014**, *1*, 577.

[9] N. Z. Burns, M. R. Witten, E. N. Jacobsen, *J. Am. Chem. Soc.* **2011**, *133,* 14578-14581.

[10] T. S. Ahmed, T. P. Montgomery, R. H. Grubbs, *Chem. Sci.* **2018**, *9*, 3580–3583.

[11] L. M. Bannwart, L. Jundt, T. Müntener, M. Neuburger, D. Häussinger, M. Mayor, *Eur. J. Org. Chem.* **2018**, 3391.

[12] C. Battilocchio, I. R., Baxendale, M. Biava, M. O. Kitching, S. V. Ley, *Org. Process Res. Dev.* **2012**, *16*, 798.

[13] S. Peil, A. Guthertz, T. Biberger, *Angew. Chem. Int. Ed.* **2019**, *58*, 8851.

[14] S. Nicolai, P. Swallow, J. Waser *Tetrahedron* **2015**, *71*, 5959-5964.

[15] G.C. Tsui, K. Villeneuve, E. Carlson, W. Tam, *Organometallics* **2014**, *33*, 3847-3856.

[16] J.-S. Ryu, T. J. Marks, F. E. McDonald, *Org. Lett.* **2001**, 3, 3091-3094.

[17] P. A. Allegretti, E. M. Ferreira, *Org. Lett.* **2011**, 13, 5924-5927.

[18] B. M. Trost, F. D. Toste, *J. Am. Chem. Soc.* **2002**, *124*, 5025-5036.

[19] L. Jafarpour, J. Huang, E. D. Stevens, S. P. Nolan, *Organometallics* **1999**, *18*, 3760-3763.

[20] X. Wu, Y. Lu, H. Hirao, J. Zhou, *Chem. Eur. J.* **2013**, *19*, 6014-6020.

[21] a) Compound **14b**: D.-H. Lee, K.-H. Kwon, C. S. Yi. *Science* **2011**, *333*, 1613 -1616; b) compound **14’b**: L. J. Rono, H. G. Yayla, D. Y. Wang, M. F. Armstrong, R. R. Knowles, *J. Am. Chem. Soc.* **2013**, *135*, 17735–17738.

[22] a) Compound **14c**: H. Luo, G. Hu, P. Li, *J. Org. Chem.* **2019**, *84*, 10569–10578; b) compound **14’c**: J. Tallineau, G. Bashiardes, J.-M. Coustard, F. Lecornué, *Synlett* **2009**,*17*, 2761–2764.

[23] V. J. Olsson, K. J. Szabó *Angew. Chem. Int. Ed.* **2007***, 46*, 6891-6893.
